# Supplementary material for: Gastrin ameliorates heart failure and suppresses myocardial remodeling via the JAK2/STAT3 and ERK1/2 pathways
Source: PLoS One. 2026 Mar 5;21(3):e0343403. doi: 10.1371/journal.pone.0343403 (PMC12962455; doi:10.1371/journal.pone.0343403)
Supplement: S1 File — (DOCX) [file pone.0343403.s001.docx]

**Fig 1.(A) Gastrin level of ISO and control group**

According to the manufacturer's instructions for the Mouse GT(Gastrin) ELISA Kit(E-EL-M2669,elabscience,Wuhan,China), standard substances were first used to perform preliminary experiments and generate a standard curve(Table 1). Subsequently, test samples were added following the specified procedural steps, the optical density (OD) values were measured, and the concentrations of the samples were determined based on the standard curve(Table 2).

Table1 The standard curve of the Mouse GT(Gastrin) ELISA Kit

| Standard curve |  | 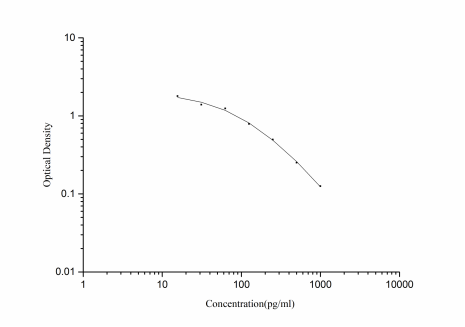 |  |  |
| --- | --- | --- | --- | --- |
| Concentration（pg/ml） | OD |  |  |  |
| 1000 | 0.126 |  |  |  |
| 500 | 0.252 |  |  |  |
| 250 | 0.498 |  |  |  |
| 125 | 0.789 |  |  |  |
| 62.5 | 1.246 |  |  |  |
| 31.25 | 1.391 |  |  |  |
| 15.63 | 1.794 |  |  |  |
| 0 | 1.993 |  |  |  |
|  |  |  |  |  |
|  |  |  |  |  |

Table2 The concentration of gastrin

| Samples | OD | Concentration（pg/ml） |
| --- | --- | --- |
| Control1 | 0.98 | 91.58 |
| Control2 | 1.24 | 55.32 |
| Control3 | 1.13 | 68.01 |
| Control4 | 1.09 | 74.22 |
| Control5 | 0.96 | 94.54 |
| Control6 | 0.97 | 92.10 |
| Control7 | 0.94 | 98.16 |
| ISO1 | 0.98 | 90.39 |
| ISO2 | 0.81 | 125.41 |
| ISO3 | 0.93 | 99.64 |
| ISO4 | 0.94 | 97.79 |
| ISO5 | 0.96 | 94.54 |
| ISO6 | 0.92 | 101.53 |
| ISO7 | 0.98 | 91.24 |

Table 3 The comparision of gastrin level between ISO and control group

| Characteristic | ISO group | Control group | t-value | P-value |
| --- | --- | --- | --- | --- |
| Gastrin concentration  （pg/ml） | 100.08±11.91 | 81.99±16.23 | -2.377 | 0.35 |

**
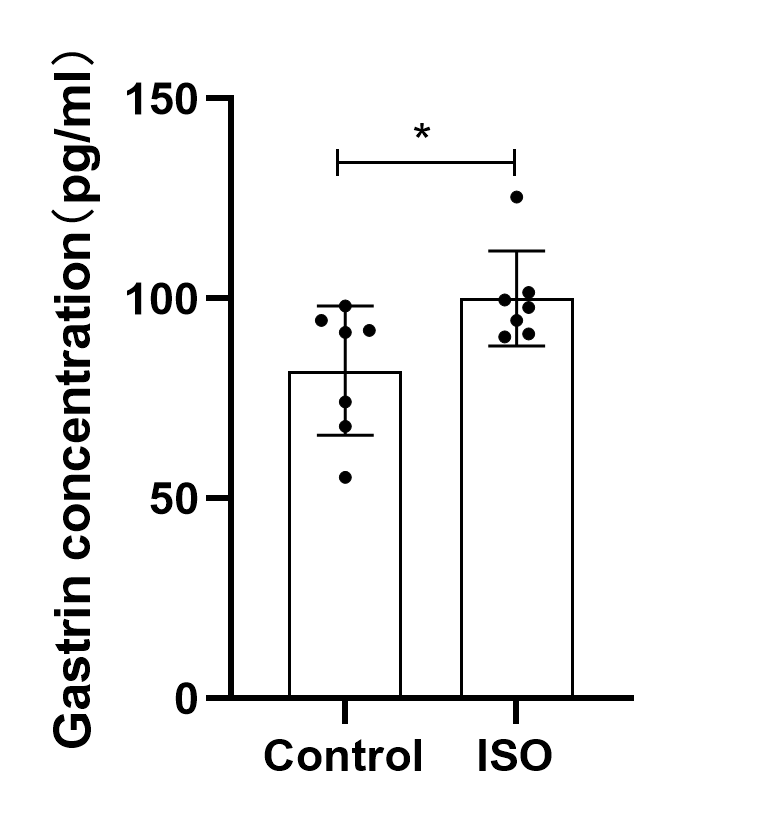
**

**Gastrin level of ISO and control group**

**Fig 1.(D) Cardiac Output(CO) of five groups**

Table 4 The CO of the mice

| CO(L/min) | Control | Gastrin | ISO | ISO+gastrin | ISO+gastrin+CI988 |
| --- | --- | --- | --- | --- | --- |
| 1 | 17.62 | 15.20 | 5.98 | 9.56 | 6.48 |
| 2 | 18.34 | 10.82 | 11.08 | 16.67 | 8.36 |
| 3 | 13.34 | 19.41 | 9.52 | 9.76 | 5.02 |
| 4 | 12.85 | 18.21 | 3.94 | 14.03 | 8.93 |
| 5 | 16.25 | 13.55 | 8.41 | 10.47 | 6.05 |
| 6 | 15.34 | 13.31 | 7.09 | 14.70 | 12.44 |
| 7 | 17.09 | 20.01 | 6.69 | 10.44 | 7.04 |

Table 5 The comparison of CO across each group

|  | groups | CO(L/min)  Mean±SD | F-value | P-value |
| --- | --- | --- | --- | --- |
| ANOVA |  |  | 14.08 | ＜0.001 |
| Multiple comparisons | Control | 15.83±2.11 |  | 0.999 |
|  | Gastrin | 15.50±4.00 |  |  |
|  | Control | 15.83±2.11 |  | ＜0.001 |
|  | ISO | 7.53±2.36 |  |  |
|  | Control | 15.83±2.11 |  | 0.15 |
|  | ISO+gatrin | 12.23±2.85 |  |  |
|  | Control | 15.83±2.11 |  | ＜0.001 |
|  | ISO+gatrin+CI988 | 7.76±2.46 |  |  |
|  | ISO | 7.53±2.36 |  | 0.031 |
|  | ISO+gastrin | 12.23±2.85 |  |  |
|  | ISO+gastrin | 12.23±2.85 |  | 0.045 |
|  | ISO+gastrin+CI988 | 7.76±2.46 |  |  |

**
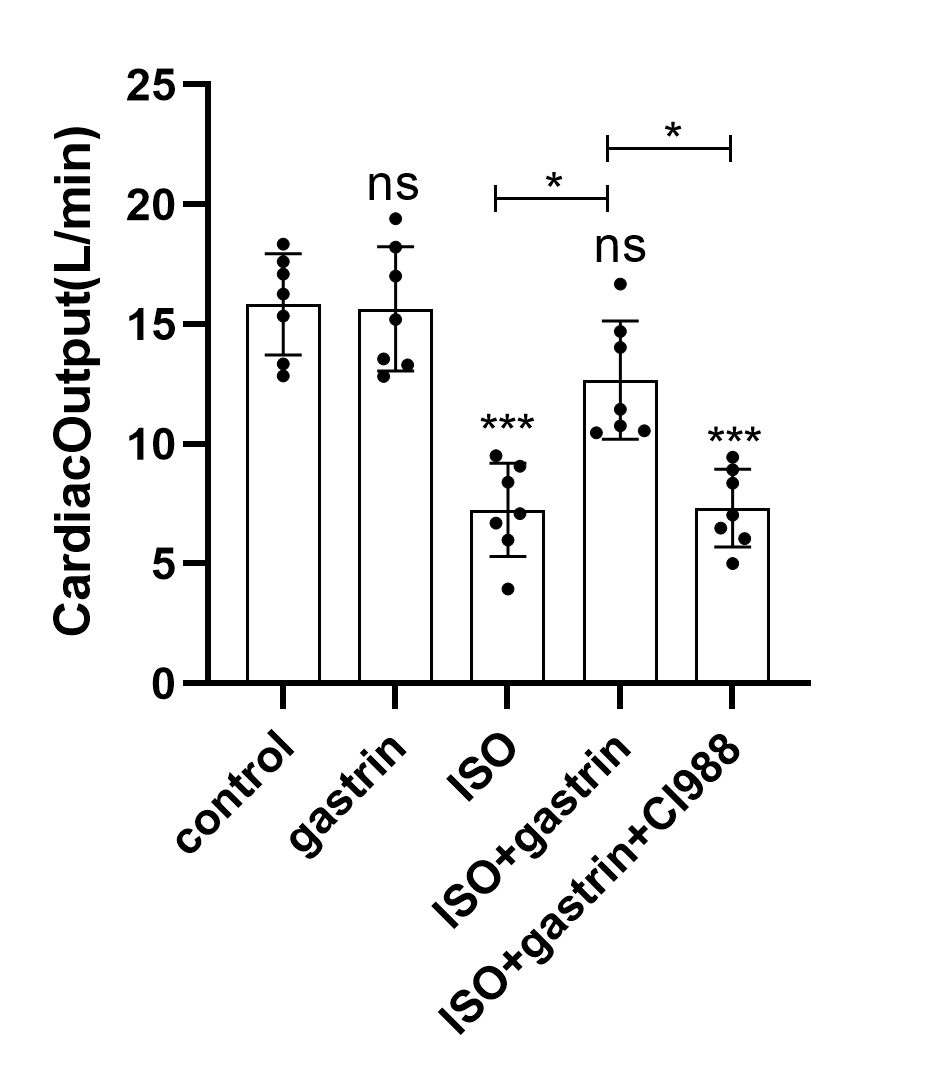
**

**The CO of five groups**

**Fig 1.(E) Left ventricular ejection fraction (EF) of five groups**

Table 6 The EF of the mice

| EF(%) | Control | Gastrin | ISO | ISO+gastrin | ISO+gastrin+CI988 |
| --- | --- | --- | --- | --- | --- |
| 1 | 58.94 | 55.52 | 41.24 | 49.22 | 41.16 |
| 2 | 68.45 | 59.40 | 42.60 | 49.23 | 26.73 |
| 3 | 58.83 | 57.93 | 38.71 | 56.32 | 33.96 |
| 4 | 76.57 | 59.07 | 36.28 | 57.62 | 22.60 |
| 5 | 58.04 | 69.28 | 40.35 | 51.47 | 52.27 |
| 6 | 59.31 | 62.17 | 30.31 | 50.34 | 56.02 |
| 7 | 59.53 | 63.06 | 36.57 | 47.63 | 30.30 |

Table 7 The comparison of EF across each group

|  | groups | EF(%)  Mean±SD | F-value | P-value |
| --- | --- | --- | --- | --- |
| ANOVA |  |  | 19.37 | ＜0.001 |
| Multiple comparisons | Control | 62.81±7.05 |  | 0.988 |
|  | Gastrin | 60.92±4.47 |  |  |
|  | Control | 62.81±7.05 |  | ＜0.001 |
|  | ISO | 38.01±4.12 |  |  |
|  | Control | 62.81±7.05 |  | 0.054 |
|  | ISO+gatrin | 51.69±3.81 |  |  |
|  | Control | 62.81±7.05 |  | ＜0.001 |
|  | ISO+gatrin+CI988 | 37.58±12.76 |  |  |
|  | ISO | 38.01±4.12 |  | 0.011 |
|  | ISO+gastrin | 51.69±3.81 |  |  |
|  | ISO+gastrin | 51.69±3.81 |  | 0.008 |
|  | ISO+gastrin+CI988 | 37.58±12.76 |  |  |

**
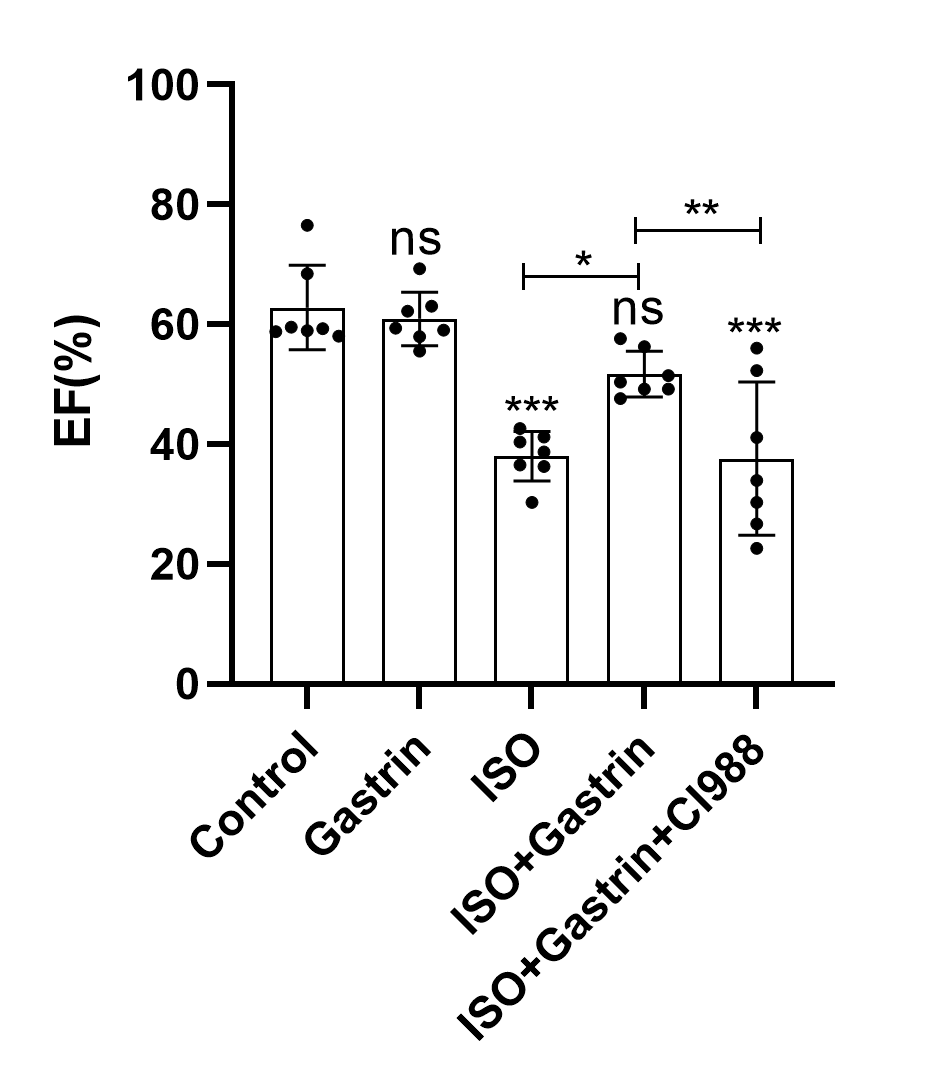
**

**The EF of five groups**

**Fig 1. (F)Left ventricular fraction shortening(FS) of five groups**

Table 8 The FS of the mice

| FS(%) | Control | Gastrin | ISO | ISO+gastrin | ISO+gastrin+CI988 |
| --- | --- | --- | --- | --- | --- |
| 1 | 30.55 | 27.81 | 19.40 | 24.07 | 19.85 |
| 2 | 37.04 | 30.86 | 20.53 | 24.78 | 12.18 |
| 3 | 30.81 | 29.96 | 18.41 | 28.40 | 15.90 |
| 4 | 43.70 | 30.48 | 16.69 | 29.72 | 10.12 |
| 5 | 29.85 | 38.15 | 19.19 | 25.92 | 25.91 |
| 6 | 30.71 | 32.68 | 13.94 | 24.23 | 28.46 |
| 7 | 30.78 | 33.62 | 17.05 | 23.17 | 13.96 |

Table9 The comparison of FS across each group

|  | groups | FS(%)  Mean±SD | F-value | P-value |
| --- | --- | --- | --- | --- |
| ANOVA |  |  | 19.484 | ＜0.001 |
| Multiple comparisons | Control | 33.35±5.18 |  | 0.974 |
|  | Gastrin | 31.94±3.32 |  |  |
|  | Control | 33.35±5.18 |  | ＜0.001 |
|  | ISO | 17.89±2.20 |  |  |
|  | Control | 33.35±5.18 |  | 0.024 |
|  | ISO+gatrin | 25.76±2.43 |  |  |
|  | Control | 33.35±5.18 |  | ＜0.001 |
|  | ISO+gatrin+CI988 | 18.06±6.97 |  |  |
|  | ISO | 17.89±2.20 |  | 0.018 |
|  | ISO+gastrin | 25.76±2.43 |  |  |
|  | ISO+gastrin | 25.76±2.43 |  | 0.021 |
|  | ISO+gastrin+CI988 | 18.06±6.97 |  |  |

**
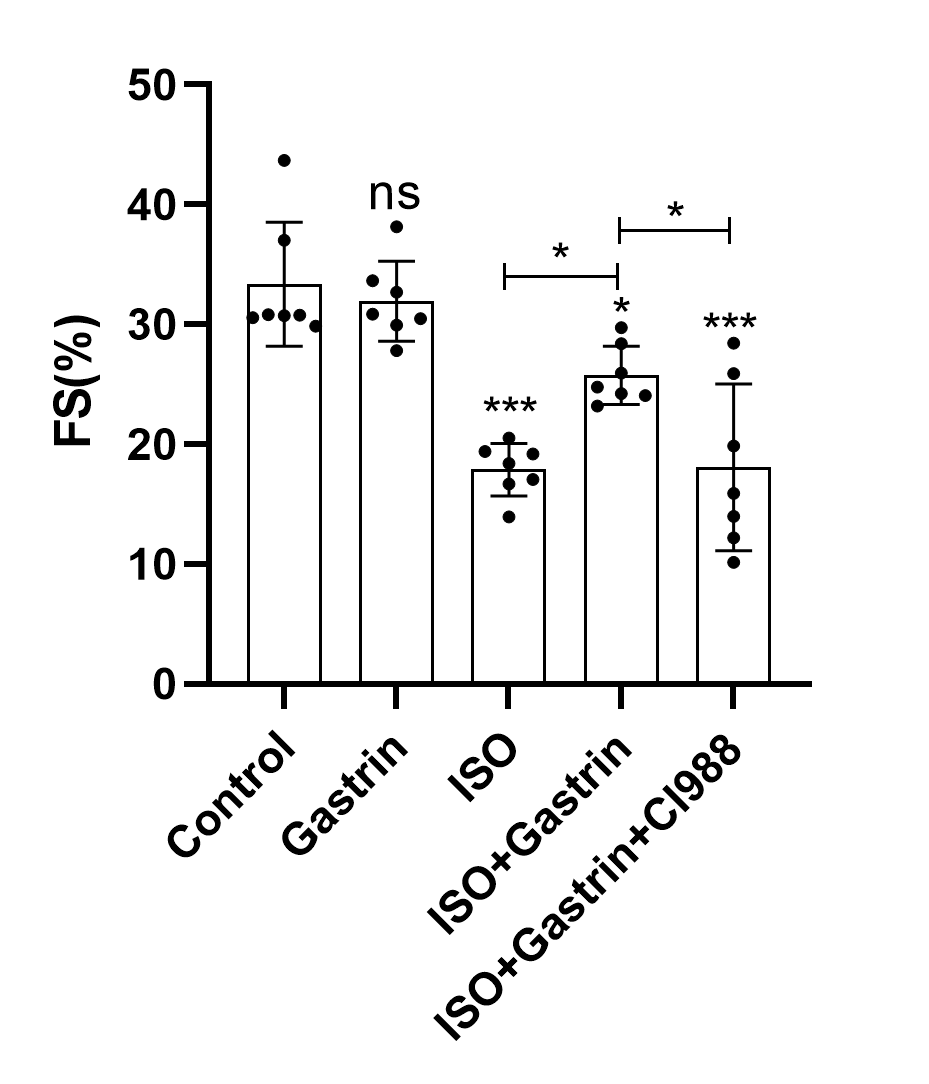
**

**The FS of five groups**

**Fig 2.(A)Heart weight/Body weight(HW/ BW) of five groups; and (B)Heart weight/Tibial length(HW/TL) of five groups**

Table10 HW/BW and HW/TL of the mice

| Groups | BW | HW | TL | HW/TL | HW/BW |
| --- | --- | --- | --- | --- | --- |
| Control | 23.5 | 0.108 | 19.415 | 0.005563 | 0.004596 |
|  | 24.3 | 0.093 | 19.68 | 0.004726 | 0.003827 |
|  | 22.6 | 0.101 | 17.585 | 0.005744 | 0.004469 |
|  | 23.7 | 0.095 | 17.045 | 0.005573 | 0.004008 |
|  | 23.1 | 0.102 | 17.6 | 0.005795 | 0.004416 |
|  | 24.1 | 0.105 | 17.38 | 0.006041 | 0.004357 |
|  | 24 | 0.107 | 19.59 | 0.005462 | 0.004458 |
| Gastrin | 24.4 | 0.105 | 17.66 | 0.005946 | 0.004303 |
|  | 21.3 | 0.089 | 17.245 | 0.005161 | 0.004178 |
|  | 22.4 | 0.095 | 18.205 | 0.005218 | 0.004241 |
|  | 25.5 | 0.105 | 18.4 | 0.005707 | 0.004118 |
|  | 24.2 | 0.101 | 19.695 | 0.005128 | 0.004174 |
|  | 24.8 | 0.102 | 16.47 | 0.006193 | 0.004113 |
|  | 22.4 | 0.103 | 16.73 | 0.006157 | 0.004598 |
| ISO | 22.7 | 0.119 | 17.35 | 0.006859 | 0.005242 |
|  | 24.2 | 0.124 | 17.115 | 0.007245 | 0.005124 |
|  | 23.9 | 0.123 | 16.715 | 0.007359 | 0.005146 |
|  | 24 | 0.123 | 17.215 | 0.007145 | 0.005125 |
|  | 24.8 | 0.131 | 17.67 | 0.007414 | 0.005282 |
|  | 22.2 | 0.121 | 17.33 | 0.006982 | 0.00545 |
|  | 22.9 | 0.13 | 14.755 | 0.008811 | 0.005677 |
| ISO+  Gastrin | 25.3 | 0.116 | 17.955 | 0.006461 | 0.004585 |
|  | 23.3 | 0.111 | 17.555 | 0.006323 | 0.004764 |
|  | 24.1 | 0.115 | 16.93 | 0.006793 | 0.004772 |
|  | 22.8 | 0.108 | 17.195 | 0.006281 | 0.004737 |
|  | 22.1 | 0.106 | 17.295 | 0.006129 | 0.004796 |
|  | 23.7 | 0.113 | 17.31 | 0.006528 | 0.004768 |
|  | 25.1 | 0.12 | 17.53 | 0.006845 | 0.004781 |
| ISO+Gastrin  +CI988 | 23.6 | 0.12 | 16.76 | 0.007160 | 0.005085 |
|  | 24.4 | 0.119 | 17.17 | 0.006931 | 0.004877 |
|  | 23.7 | 0.118 | 16.19 | 0.007288 | 0.004979 |
|  | 22.5 | 0.122 | 17.36 | 0.007028 | 0.005422 |
|  | 22.9 | 0.119 | 17 | 0.007000 | 0.005197 |
|  | 22.4 | 0.126 | 16.725 | 0.007534 | 0.005625 |
|  | 23.4 | 0.121 | 16.865 | 0.007175 | 0.005171 |

Table11 The comparison of HW/BW across each group

|  | groups | HW/BW(g/g)  Mean±SD | F-value | P-value |
| --- | --- | --- | --- | --- |
| ANOVA |  |  | 37.523 | ＜0.001 |
| Multiple comparisons | Control | 0.0043±0.0003 |  | 0.985 |
|  | Gastrin | 0.0042±0.0002 |  |  |
|  | Control | 0.0043±0.0003 |  | ＜0.001 |
|  | ISO | 0.0053±0.0002 |  |  |
|  | Control | 0.0043±0.0003 |  | 0.004 |
|  | ISO+gatrin | 0.0047±0.0007 |  |  |
|  | Control | 0.0043±0.0003 |  | ＜0.001 |
|  | ISO+gatrin+CI988 | 0.0052±0.0003 |  |  |
|  | ISO | 0.0053±0.0002 |  | ＜0.001 |
|  | ISO+gastrin | 0.0047±0.0007 |  |  |
|  | ISO+gastrin | 0.0047±0.0007 |  | 0.003 |
|  | ISO+gastrin+CI988 | 0.0052±0.0003 |  |  |


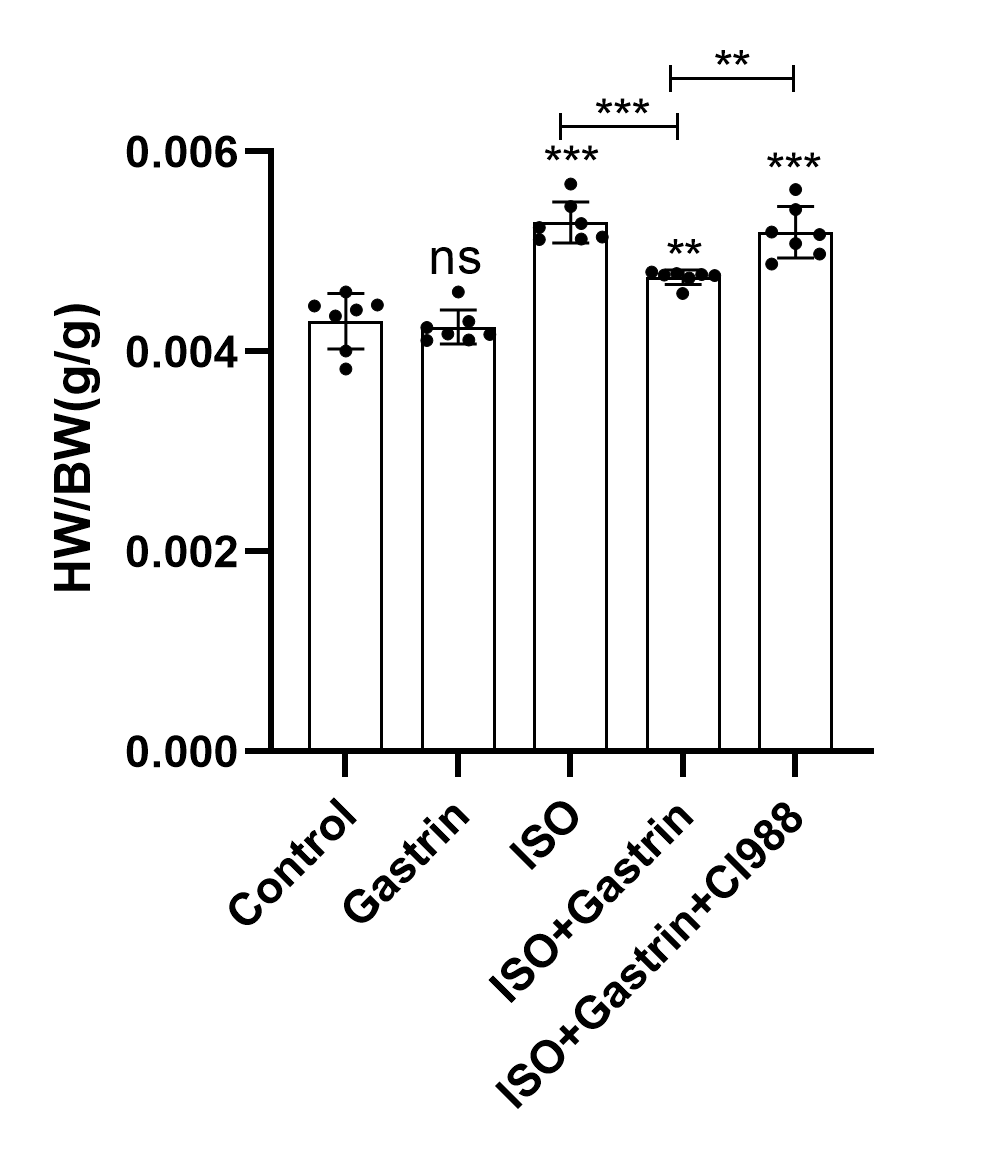


**The HW/ BW of five groups**

Table12 The comparison of HW/TL across each group

|  | groups | HW/TL(g/mm)  Mean±SD | F-value | P-value |
| --- | --- | --- | --- | --- |
| ANOVA |  |  | 26.826 | ＜0.001 |
| Multiple comparisons | Control | 0.0056±0.0004 |  | 0.996 |
|  | Gastrin | 0.0056±0.0005 |  |  |
|  | Control | 0.0056±0.0004 |  | ＜0.001 |
|  | ISO | 0.0074±0.0006 |  |  |
|  | Control | 0.0056±0.0004 |  | 0.003 |
|  | ISO+gatrin | 0.0065±0.0003 |  |  |
|  | Control | 0.0056±0.0004 |  | ＜0.001 |
|  | ISO+gatrin+CI988 | 0.0072±0.0002 |  |  |
|  | ISO | 0.0074±0.0006 |  | 0.003 |
|  | ISO+gastrin | 0.0065±0.0003 |  |  |
|  | ISO+gastrin | 0.0065±0.0003 |  | 0.045 |
|  | ISO+gastrin+CI988 | 0.0072±0.0002 |  |  |


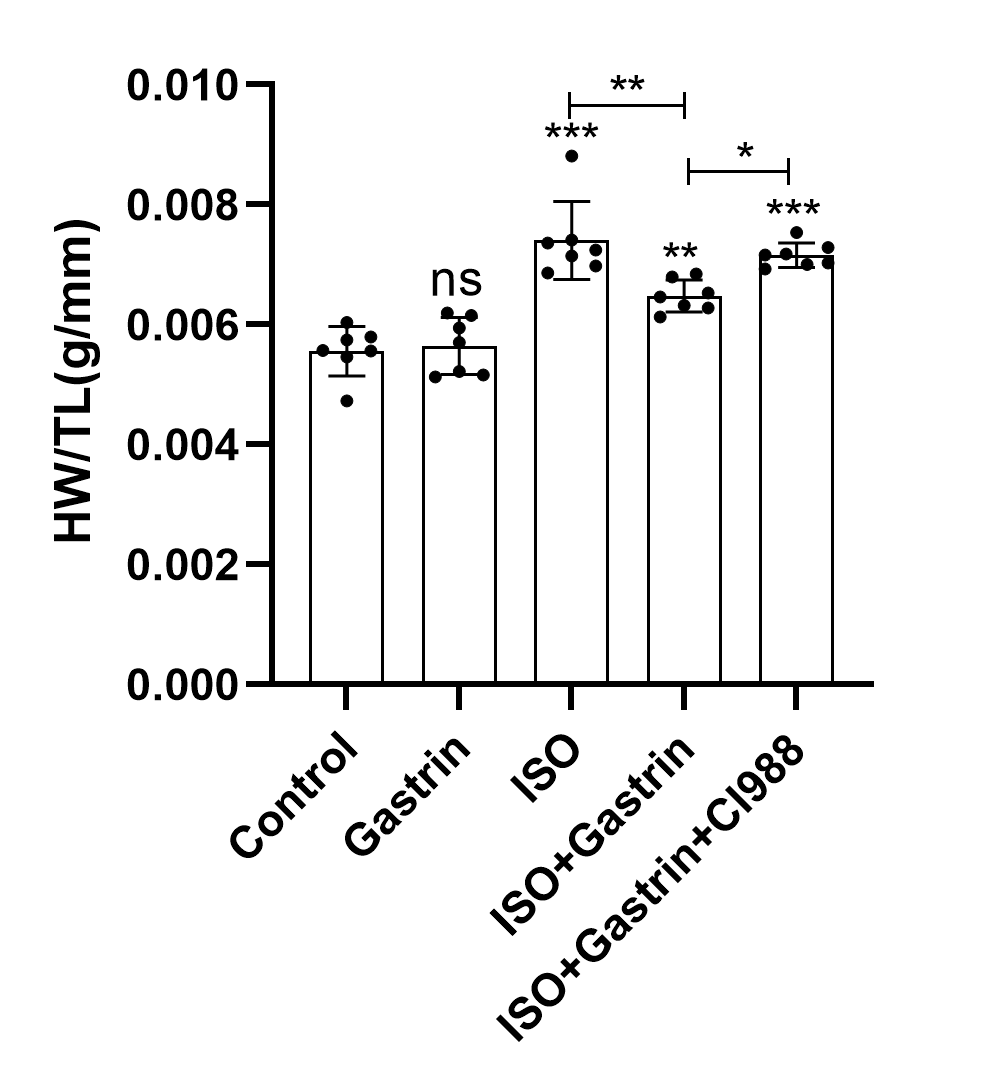


**The HW/TL of five groups**

**Fig 2.(C)Posterior wall of left ventricle(LVPWs) of five groups**

Table 13 The LVPWs of the mice

|  | Control  (mm) | Gastrin  (mm) | ISO  (mm) | ISO+Gastrin  (mm) | ISO+Gastrin+CI988  (mm) |
| --- | --- | --- | --- | --- | --- |
| 1 | 0.98 | 0.88 | 1.05 | 0.95 | 1.19 |
| 2 | 0.92 | 1.04 | 1.13 | 0.93 | 1.18 |
| 3 | 0.97 | 1.09 | 1.31 | 1.00 | 0.98 |
| 4 | 0.98 | 0.99 | 1.20 | 0.84 | 0.92 |
| 5 | 1.04 | 0.85 | 1.29 | 0.92 | 1.13 |
| 6 | 1.00 | 1.02 | 1.18 | 0.81 | 1.31 |
| 7 | 0.84 | 1.04 | .91 | 0.98 | 1.14 |

Table14 The comparison of LVPWs across each group

|  | groups | LVPWs(mm)  Mean±SD | F-value | P-value |
| --- | --- | --- | --- | --- |
| ANOVA |  |  | 6.895 | ＜0.001 |
| Multiple comparisons | Control | 0.96±0.64 |  | 0.991 |
|  | Gastrin | 0.99±0.86 |  |  |
|  | Control | 0.96±0.64 |  | 0.015 |
|  | ISO | 1.15±0.14 |  |  |
|  | Control | 0.96±0.64 |  | 0.916 |
|  | ISO+gatrin | 0.92±0.07 |  |  |
|  | Control | 0.96±0.64 |  | 0.057 |
|  | ISO+gatrin+CI988 | 1.12±0.13 |  |  |
|  | ISO | 1.15±0.14 |  | 0.002 |
|  | ISO+gastrin | 0.92±0.07 |  |  |
|  | ISO+gastrin | 0.92±0.07 |  | 0.007 |
|  | ISO+gastrin+CI988 | 1.12±0.13 |  |  |


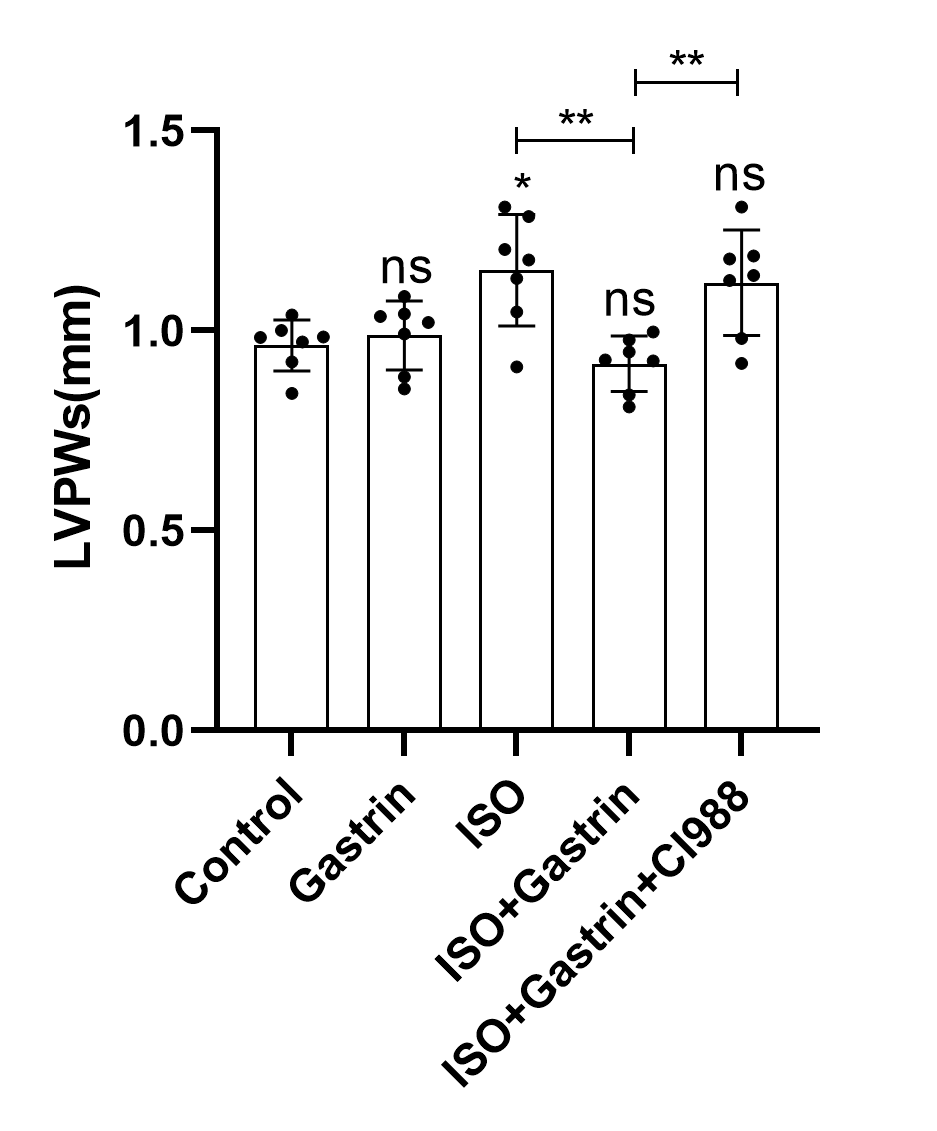


**The LVPWs of five groups**

**Fig 2.(D) Cardiomyocyte cross-sectional areas** **(CSA) of five groups**

Five distinct fields of view were selected from each sample, and ten cross-sectional cells were measured in each field to determine the cell surface area. The average surface area was then calculated based on these 50 individual cell measurements. The values listed below represent the mean cell surface area for each sample.

Table 15 The CSA of five groups

|  | Control  (um^2^) | Gastrin  (um^2^) | ISO  (um^2^) | ISO+Gastrin  (um^2^) | ISO+Gastrin+CI988  (um^2^) |
| --- | --- | --- | --- | --- | --- |
| 1 | 268.43 | 305.11 | 296.21 | 207.45 | 272.31 |
| 2 | 280.46 | 255.86 | 303.92 | 221.02 | 293.08 |
| 3 | 275.04 | 240.40 | 352.22 | 214.75 | 315.94 |
| 4 | 241.54 | 207.15 | 392.58 | 235.13 | 257.10 |
| 5 | 236.57 | 199.40 | 342.09 | 253.42 | 267.24 |
| 6 | 280.85 | 208.34 | 318.18 | 257.10 | 255.61 |
| 7 | 257.50 | 202.93 | 319.17 | 251.83 | 279.86 |

Table16 The comparison of CSA across each group

|  | groups | CSA(um^2^)  Mean±SD | F-value | P-value |
| --- | --- | --- | --- | --- |
| ANOVA |  |  | 15.348 | ＜0.001 |
| Multiple comparisons | Control | 262.91±18.20 |  | 0.23 |
|  | Gastrin | 231.31±38.87 |  |  |
|  | Control | 262.91±18.20 |  | ＜0.001 |
|  | ISO | 332.05±33.18 |  |  |
|  | Control | 262.91±18.20 |  | 0.323 |
|  | ISO+gatrin | 234.39±20.30 |  |  |
|  | Control | 262.91±18.20 |  | 0.864 |
|  | ISO+gatrin+CI988 | 277.30±21.42 |  |  |
|  | ISO | 332.05±33.18 |  | ＜0.001 |
|  | ISO+gastrin | 234.39±20.30 |  |  |
|  | ISO+gastrin | 234.39±20.30 |  | 0.049 |
|  | ISO+gastrin+CI988 | 277.30±21.42 |  |  |


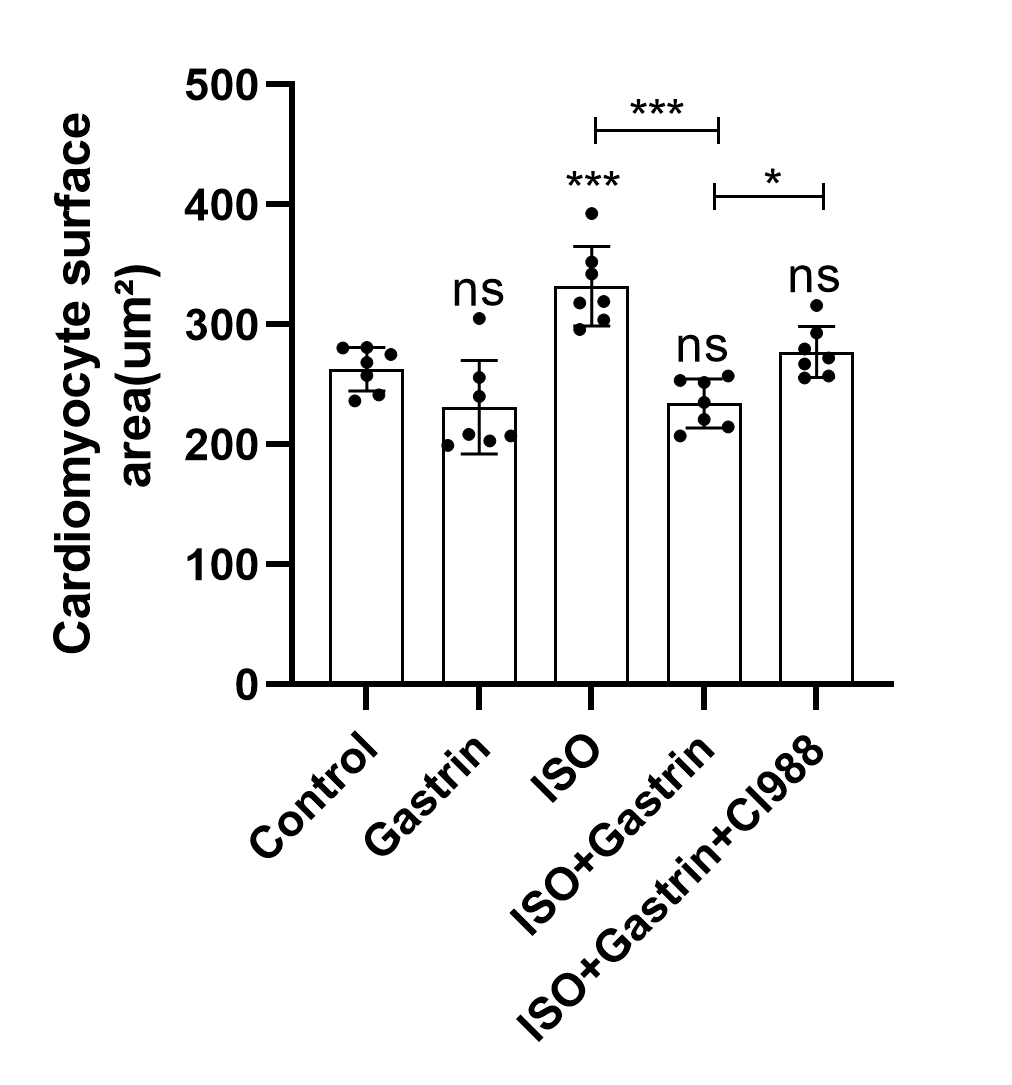


**The CSA of five groups**

**Fig 2.(F)Expression of ANP in the hearts of mice across Control, Gastrin, ISO, and ISO+Gastrin Groups**

Table 17 Quantitative analysis of ANP expression in mice heart tissues using the 2^-ΔΔCT^ method

| ANP/18S  (2^-ΔΔCT^) | Control | Gastrin | ISO | ISO+Gastrin |
| --- | --- | --- | --- | --- |
| Group1 | 1 | 2.44 | 5.50 | 3.08 |
| Group2 | 1 | 1.82 | 3.55 | 1.79 |
| Group3 | 1 | 1.09 | 4.55 | 1.84 |
| Group4 | 1 | 2.08 | 4.18 | 2.25 |
| Group5 | 1 | 1.28 | 5.87 | 1.73 |
| Group6 | 1 | 1.24 | 2.84 | 1.68 |

Table18 The comparison of ANP across each group

|  | groups | ANP(2^-ΔΔCT^)  Mean±SD | F-value | P-value |
| --- | --- | --- | --- | --- |
| ANOVA |  |  | 27.912 | ＜0.001 |
| Multiple comparisons | Control | 1 |  | 0.374 |
|  | Gastrin | 1.66±0.54 |  |  |
|  | Control | 1 |  | ＜0.001 |
|  | ISO | 4.42±1.15 |  |  |
|  | Control | 1 |  | 0.065 |
|  | ISO+gatrin | 2.06±0.54 |  |  |
|  | ISO | 4.42±1.15 |  | ＜0.001 |
|  | ISO+gastrin | 2.06±0.54 |  |  |


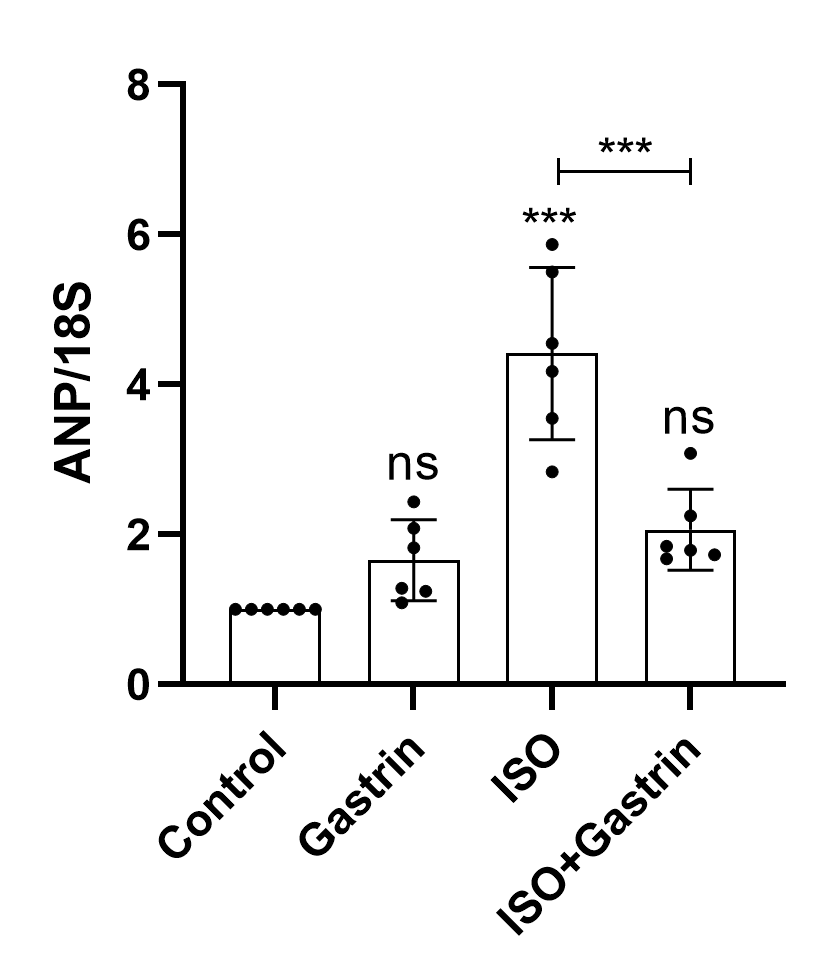


**The ANP expression of the heart tissues**

**Fig 2.(G)Expression of BNP in the hearts of mice across Control, Gastrin, ISO, and ISO+Gastrin Groups**

Table 19 Quantitative analysis of BNP expression in mouse hearts using the 2^-ΔΔCT^ method

| BNP/18S  (2^-ΔΔCT^) | Control | Gastrin | ISO | ISO+Gastrin |
| --- | --- | --- | --- | --- |
| Group1 | 1 | 1.32 | 3.69 | 1.85 |
| Group2 | 1 | 1.29 | 3.60 | 1.27 |
| Group3 | 1 | 1.48 | 5.41 | 2.89 |
| Group4 | 1 | 1.11 | 4.40 | 1.49 |
| Group5 | 1 | 1.32 | 4.87 | 1.35 |
| Group6 | 1 | 1.56 | 4.21 | 1.64 |

Table20 The comparison of BNP across each group

|  | groups | BNP(2^-ΔΔCT^)  Mean±SD | F-value | P-value |
| --- | --- | --- | --- | --- |
| ANOVA |  |  | 65.206 | ＜0.001 |
| Multiple comparisons | Control | 1 |  | 0.576 |
|  | Gastrin | 1.35±1.59 |  |  |
|  | Control | 1 |  | ＜0.001 |
|  | ISO | 4.36±0.69 |  |  |
|  | Control | 1 |  | 0.052 |
|  | ISO+gatrin | 1.75±0.60 |  |  |
|  | ISO | 4.36±0.69 |  | ＜0.001 |
|  | ISO+gastrin | 1.75±0.60 |  |  |


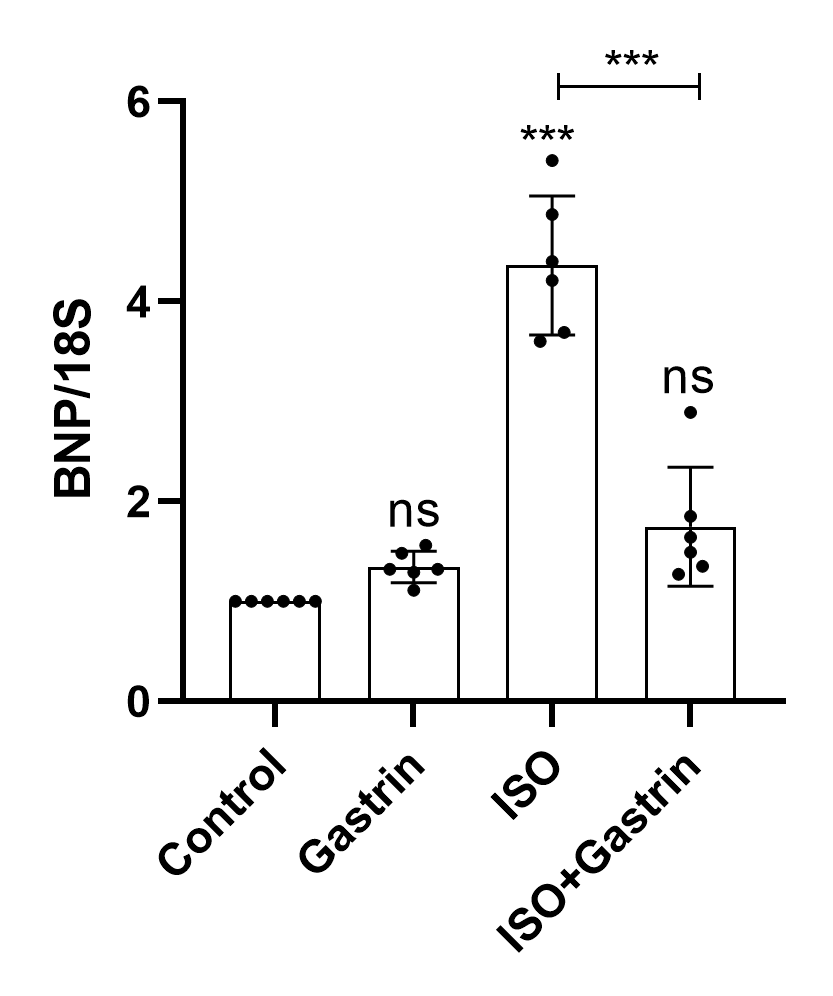


**The BNP expression of the heart tissues**

**Fig 2.(H)Expression of β-MHC in the hearts of mice across Control, Gastrin, ISO, and ISO+Gastrin Groups**

Table 21 Quantitative analysis of β-MHC expression in mouse hearts using the 2^-ΔΔCT^ method

| β-MHC/18S  (2^-ΔΔCT^) | Control | Gastrin | ISO | ISO+Gastrin |
| --- | --- | --- | --- | --- |
| Group1 | 1 | 1.23 | 7.41 | 1.58 |
| Group2 | 1 | 1.20 | 4.66 | 1.40 |
| Group3 | 1 | 1.60 | 4.79 | 1.92 |
| Group4 | 1 | 1.24 | 6.55 | 1.29 |
| Group5 | 1 | 1.14 | 6.15 | 2.90 |
| Group6 | 1 | 1.10 | 6.19 | 1.17 |

Table22 The comparison of β-MHC across each group

|  | groups | β-MHC(2^-ΔΔCT^)  Mean±SD | F-value | P-value |
| --- | --- | --- | --- | --- |
| ANOVA |  |  | 83.914 | ＜0.001 |
| Multiple comparisons | Control | 1 |  | 0.898 |
|  | Gastrin | 1.25±0.18 |  |  |
|  | Control | 1 |  | ＜0.001 |
|  | ISO | 5.96±1.06 |  |  |
|  | Control | 1 |  | 0.236 |
|  | ISO+gatrin | 1.71±0.64 |  |  |
|  | ISO | 5.96±1.06 |  | ＜0.001 |
|  | ISO+gastrin | 1.71±0.64 |  |  |


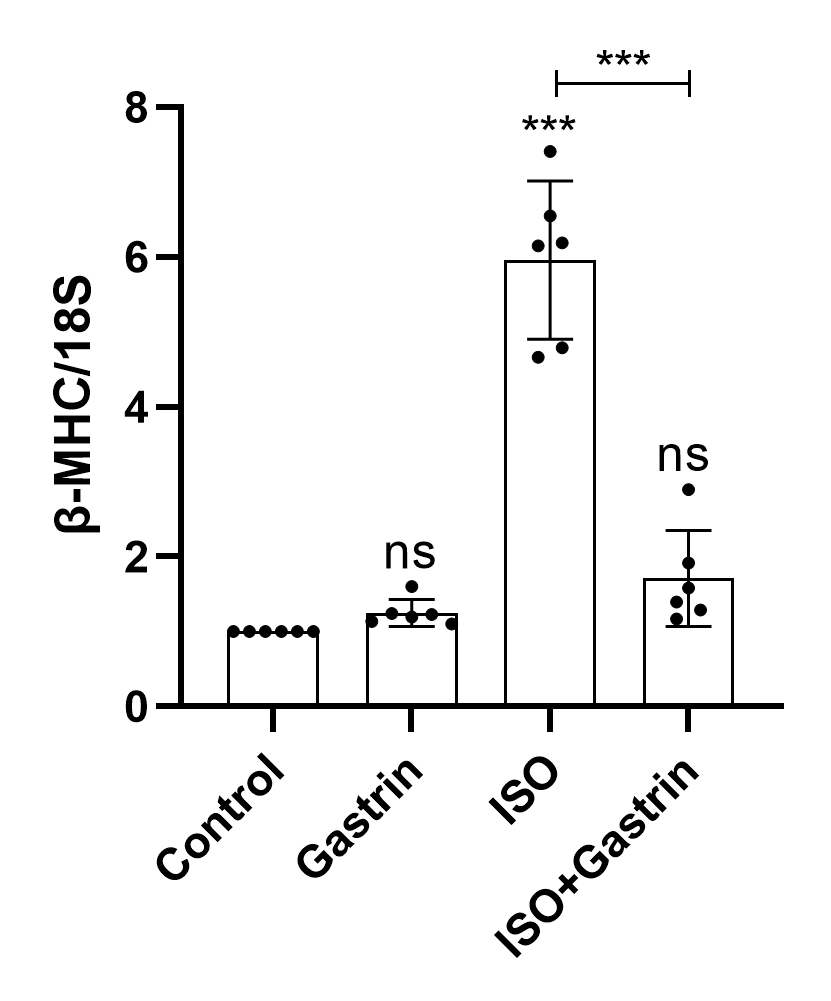


**Theβ-MHC expression of the heart tissues**

**Fig 2.(I)Expression of ANP in the hearts of mice across Control, ISO, ISO+Gastrin and ISO+Gastrin+CI-988 groups**

Table 23 Quantitative analysis of ANP expression in mouse hearts using the 2^-ΔΔCT^ method

| ANP/18S  (2^-ΔΔCT^) | Control | ISO | ISO+Gastrin | ISO+Gastrin+CI988 |
| --- | --- | --- | --- | --- |
| Group1 | 1 | 3.89 | 1.09 | 3.3 |
| Group2 | 1 | 3.21 | 1.08 | 3 |
| Group3 | 1 | 5.34 | 1.8 | 4.56 |
| Group4 | 1 | 5.49 | 1.82 | 2.22 |
| Group5 | 1 | 5.47 | 1.26 | 3.85 |
| Group6 | 1 | 5.1 | 2.05 | 4.38 |

Table24 The comparison of ANP across each group

|  | groups | ANP(2^-ΔΔCT^)  Mean±SD | F-value | P-value |
| --- | --- | --- | --- | --- |
| ANOVA |  |  | 38.913 | ＜0.001 |
| Multiple comparisons | Control | 1 |  | ＜0.001 |
|  | ISO | 4.75±0.97 |  |  |
|  | Control | 1 |  | 0.574 |
|  | ISO+gatrin | 1.52±0.42 |  |  |
|  | Control | 1 |  | ＜0.001 |
|  | ISO+gatrin+CI988 | 3.55±0.88 |  |  |
|  | ISO | 4.75±0.97 |  | ＜0.001 |
|  | ISO+gastrin | 1.52±0.42 |  |  |
|  | ISO+gastrin | 1.52±0.42 |  | ＜0.001 |
|  | ISO+gatrin+CI988 | 3.55±0.88 |  |  |


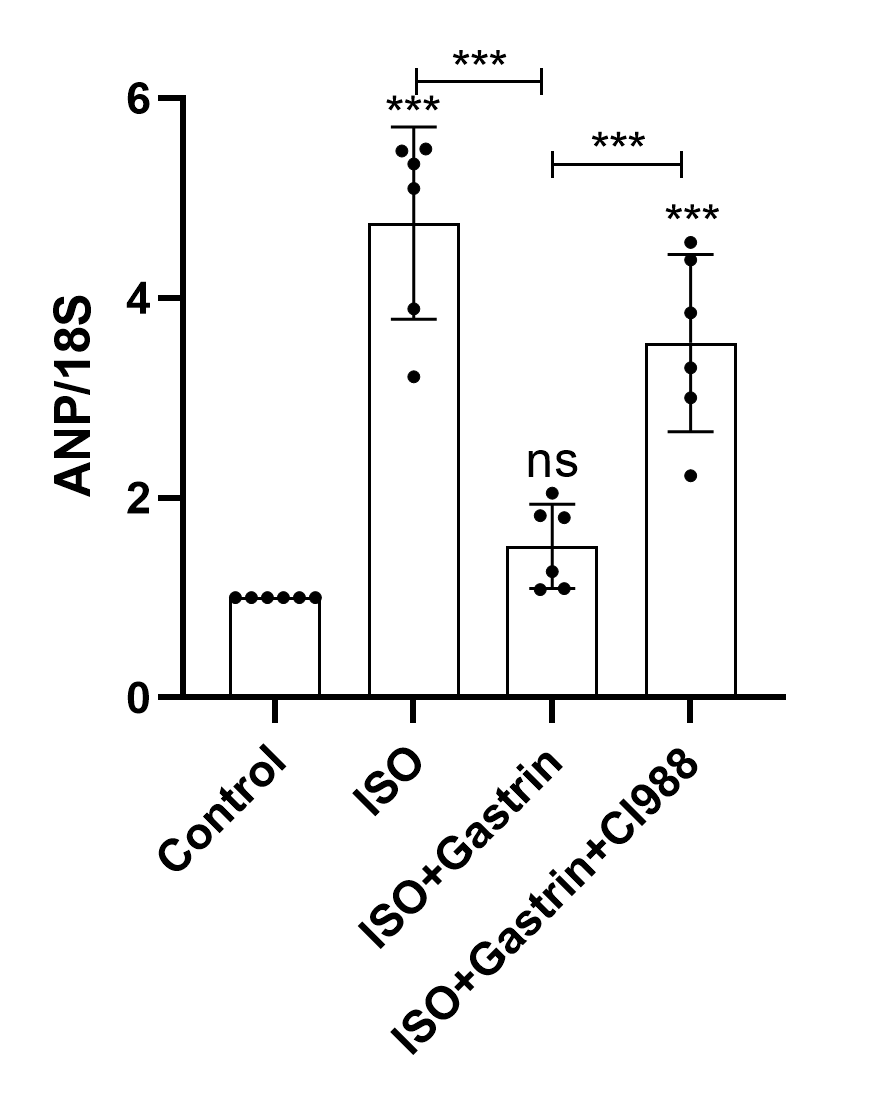


**The ANP expression of the heart tissues**

**Fig 2.(J)Expression of BNP in the hearts of mice across Control, ISO, ISO+Gastrin and ISO+Gastrin+CI-988 groups**

Table 25 Quantitative analysis of BNP expression in mouse hearts using the 2^-ΔΔCT^ method

| BNP/18S  (2^-ΔΔCT^) | Control | ISO | ISO+Gastrin | ISO+Gastrin+CI988 |
| --- | --- | --- | --- | --- |
| Group1 | 1 | 4.4 | 1.67 | 3.51 |
| Group2 | 1 | 4.45 | 1.37 | 4.09 |
| Group3 | 1 | 5.32 | 1.17 | 2.96 |
| Group4 | 1 | 5.39 | 1.35 | 4.03 |
| Group5 | 1 | 6.59 | 1.81 | 2.9 |
| Group6 | 1 | 4.98 | 1.36 | 3.04 |

Table26 The comparison of BNP across each group

|  | groups | BNP(2^-ΔΔCT^)  Mean±SD | F-value | P-value |
| --- | --- | --- | --- | --- |
| ANOVA |  |  | 89.894 | ＜0.001 |
| Multiple comparisons | Control | 1 |  | ＜0.001 |
|  | ISO | 5.19±0.80 |  |  |
|  | Control | 1 |  | 0.414 |
|  | ISO+gatrin | 1.445±0.24 |  |  |
|  | Control | 1 |  | ＜0.001 |
|  | ISO+gatrin+CI988 | 3.42±0.54 |  |  |
|  | ISO | 5.19±0.80 |  | ＜0.001 |
|  | ISO+gastrin | 1.445±0.24 |  |  |
|  | ISO+gastrin | 1.445±0.24 |  | ＜0.001 |
|  | ISO+gatrin+CI988 | 3.42±0.54 |  |  |


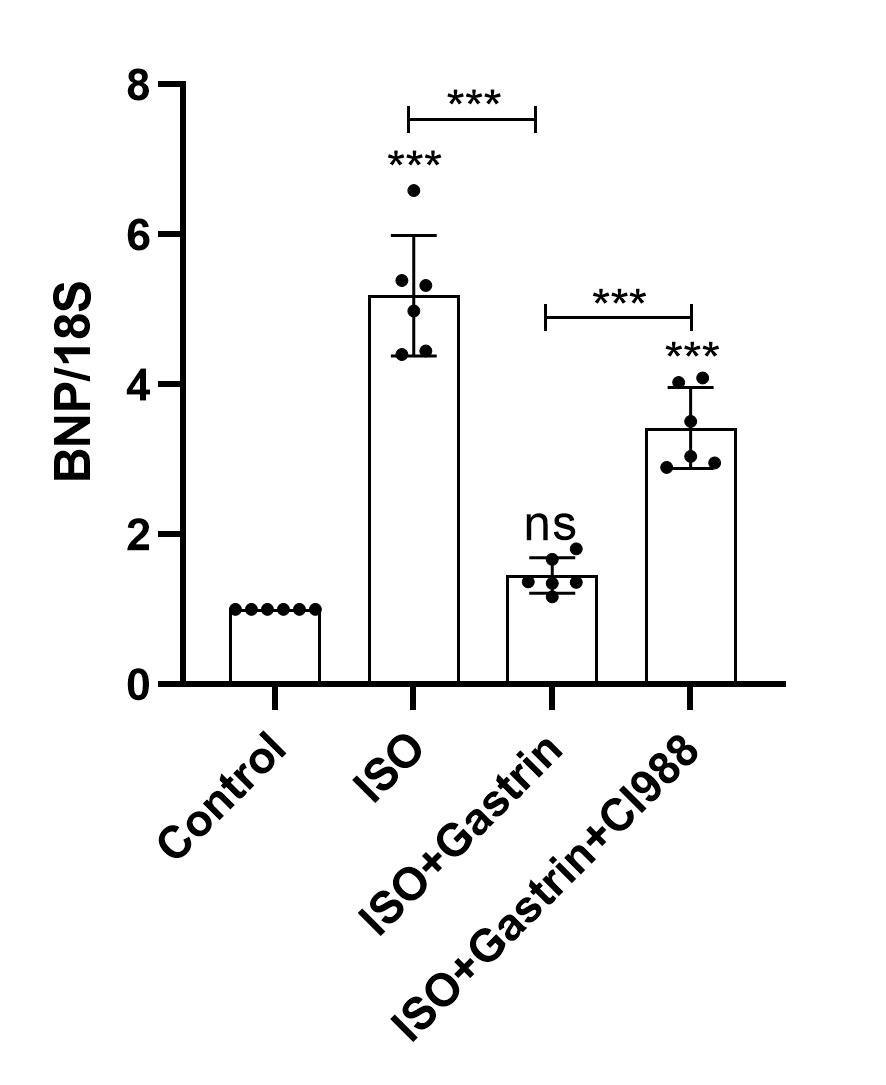


**The BNP expression of the heart tissues**

**Fig 2.(K)Expression of β-MHC in the hearts of mice across Control, ISO, ISO+Gastrin and ISO+Gastrin+CI-988 groups**

Table 27 Quantitative analysis of β-MHC expression in mouse hearts using the 2^-ΔΔCT^ method

| β-MHC/18S  (2^-ΔΔCT^) | Control | ISO | ISO+Gastrin | ISO+Gastrin+CI988 |
| --- | --- | --- | --- | --- |
| Group1 | 1 | 6.22 | 2.45 | 5.11 |
| Group2 | 1 | 5.14 | 1.65 | 3.37 |
| Group3 | 1 | 7.73 | 1.92 | 5.08 |
| Group4 | 1 | 5.15 | 1.55 | 3.68 |
| Group5 | 1 | 5.11 | 1.64 | 2.89 |
| Group6 | 1 | 5.39 | 1.69 | 2.76 |

Table28 The comparison of β-MHC across each group

|  | groups | β-MHC(2^-ΔΔCT^)  Mean±SD | F-value | P-value |
| --- | --- | --- | --- | --- |
| ANOVA |  |  | 48.298 | ＜0.001 |
| Multiple comparisons | Control | 1 |  | ＜0.001 |
|  | ISO | 5.79±1.04 |  |  |
|  | Control | 1 |  | 0.269 |
|  | ISO+gatrin | 1.82±0.33 |  |  |
|  | Control | 1 |  | ＜0.001 |
|  | ISO+gatrin+CI988 | 3.81±1.04 |  |  |
|  | ISO | 5.79±1.04 |  | ＜0.001 |
|  | ISO+gastrin | 1.82±0.33 |  |  |
|  | ISO+gastrin | 1.82±0.33 |  | ＜0.001 |
|  | ISO+gatrin+CI988 | 3.81±1.04 |  |  |


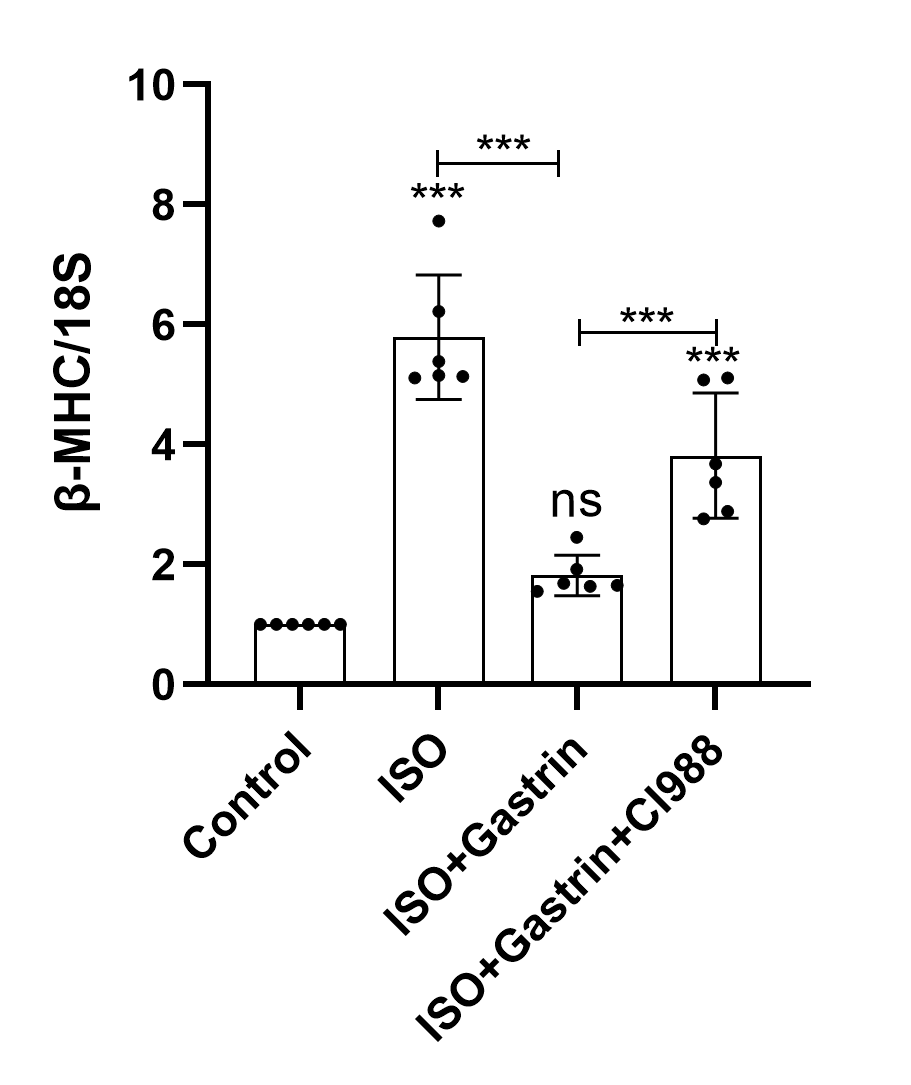


**The β-MHC expression of the heart tissues**

**Fig 3.(C)The relative fibrotic area as demonstrated by Masson's trichrome staining**

Following a comprehensive scanning of the tissue sections, the collagen content across the entire tissue area was quantitatively analyzed, and intergroup comparisons of collagen proportions were performed.

Table29 The proportion of collagen in tissue samples

| (%) | Control | Gastrin | ISO | ISO+Gastrin | ISO+Gastrin+CI988 |
| --- | --- | --- | --- | --- | --- |
| 1 | 2.8218 | 4.1009 | 7.4685 | 8.1501 | 10.0056 |
| 2 | 3.2563 | 3.3474 | 21.9285 | 4.3023 | 9.6849 |
| 3 | 2.5307 | 3.6282 | 13.8763 | 8.4536 | 10.9784 |
| 4 | 3.3795 | 3.3585 | 7.0353 | 5.4023 | 12.6491 |
| 5 | 2.8249 | 2.9887 | 23.898 | 5.5864 | 10.9338 |
| 6 | 4.4932 | 2.9015 | 14.9087 | 4.1355 | 14.0359 |
| 7 | 2.6907 | 2.7151 | 21.8492 | 3.9802 | 8.838 |

Table30 The comparison of relative fibrotic areas in each group

|  | groups | relative fibrotic areas(%)  Mean±SD | F-value | P-value |
| --- | --- | --- | --- | --- |
| ANOVA |  |  | 19.059 | ＜0.001 |
| Multiple comparisons | Control | 3.14±0.67 |  | 1.00 |
|  | Gastrin | 3.29±0.47 |  |  |
|  | Control | 3.14±0.67 |  | ＜0.001 |
|  | ISO | 15.85±6.96 |  |  |
|  | Control | 3.14±0.67 |  | 0.607 |
|  | ISO+gatrin | 5.71±1.87 |  |  |
|  | Control | 3.14±0.67 |  | ＜0.001 |
|  | ISO+gatrin+CI988 | 11.02±1.79 |  |  |
|  | ISO | 15.85±6.96 |  | ＜0.001 |
|  | ISO+gastrin | 5.71±1.87 |  |  |
|  | ISO+gastrin | 5.71±1.87 |  | 0.043 |
|  | ISO+gastrin+CI988 | 11.02±1.79 |  |  |

**
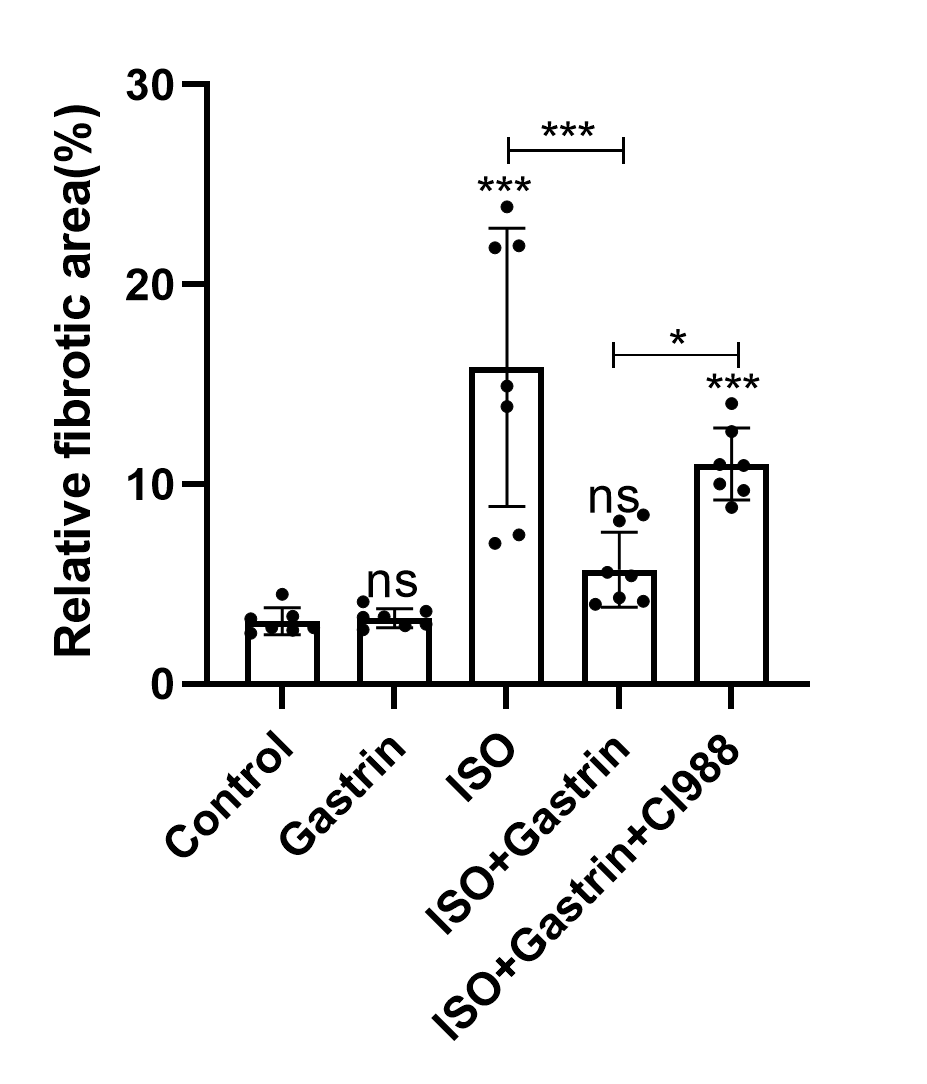
**

**The relative fibrotic areas of the five groups**

**Fig 3.(D) Quantitative Analysis of Type I Collagen Area**

Under a 20x magnification, 10 random microscopic fields were selected from different regions of each tissue section to calculate the collagen area percentage. The mean collagen area percentage across the 10 fields was then determined for each section to represent the collagen content of that section. Subsequently, collagen percentages from seven samples per group were compared and statistically analyzed.

Table31 The percentage of Collagen I of the hearts

| (%) | Control | Gastrin | ISO | ISO+Gastrin | ISO+Gastrin+CI988 |
| --- | --- | --- | --- | --- | --- |
| 1 | 4.61 | 3.47 | 8.95 | 8.81 | 10.62 |
| 2 | 4.75 | 2.62 | 13.85 | 4.93 | 11.9 |
| 3 | 3.18 | 3.64 | 9.61 | 9.15 | 11.43 |
| 4 | 4.7 | 2.5 | 16.73 | 7.98 | 10.9 |
| 5 | 3.99 | 3.12 | 14.01 | 3.56 | 8.86 |
| 6 | 3.33 | 2.68 | 8.05 | 3.14 | 9.56 |
| 7 | 4.34 | 3.59 | 9.89 | 6.76 | 9.39 |

Table32 The comparison of Collagen I percentage across each group

|  | groups | Collagen I percentage  (%)  Mean±SD | F-value | P-value |
| --- | --- | --- | --- | --- |
| ANOVA |  |  | 26.38 | ＜0.001 |
| Multiple compari-sons | Control | 4.13±0.65 |  | 0.851 |
|  | Gastrin | 3.09±0.49 |  |  |
|  | Control | 4.13±0.65 |  | ＜0.001 |
|  | ISO | 11.58±3.26 |  |  |
|  | Control | 4.13±0.65 |  | 0.233 |
|  | ISO+gatrin | 6.22±2.48 |  |  |
|  | Control | 4.13±0.65 |  | ＜0.001 |
|  | ISO+gatrin+CI988 | 10.38±1.13 |  |  |
|  | ISO | 11.58±3.26 |  | ＜0.001 |
|  | ISO+gastrin | 6.22±2.48 |  |  |
|  | ISO+gastrin | 6.22±2.48 |  | 0.004 |
|  | ISO+gastrin+CI988 | 10.38±1.13 |  |  |


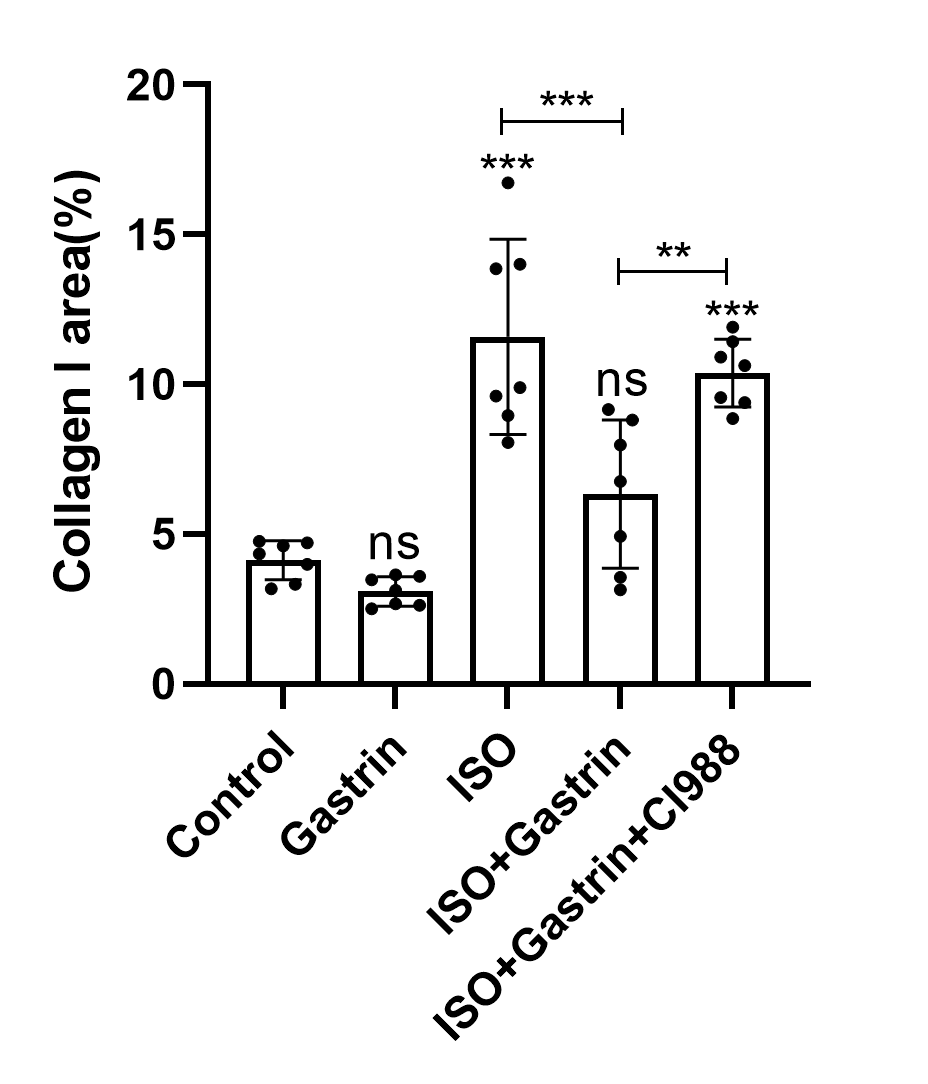


**The percentage of Collagen I of five groups**

**Fig 3.(E)Quantitative Analysis of Type III Collagen Area**

The calculation method is consistent with that of Collagen I.

Table33 The percentage of Collagen III of the hearts

| (%) | Control | Gastrin | ISO | ISO+Gastrin | ISO+Gastrin+CI988 |
| --- | --- | --- | --- | --- | --- |
| 1 | 11.14 | 13.43 | 19.73 | 15.34 | 20.77 |
| 2 | 13.63 | 11.12 | 16.21 | 14.45 | 18.64 |
| 3 | 13.58 | 11.67 | 17.15 | 14.05 | 18.35 |
| 4 | 13.31 | 12.71 | 22.43 | 13.07 | 14.16 |
| 5 | 11.85 | 13.43 | 20.97 | 11.79 | 20.45 |
| 6 | 8.75 | 13.45 | 16.71 | 13.39 | 20.01 |
| 7 | 13.4 | 11.22 | 16.07 | 15.55 | 16.16 |

Table34 The comparison of Collagen III percentage across each group

|  | groups | Collagen III percentage  (%)  Mean±SD | F-value | P-value |
| --- | --- | --- | --- | --- |
| ANOVA |  |  | 13.150 | ＜0.001 |
| Multiple comparisons | Control | 12.24±1.81 |  | 1.000 |
|  | Gastrin | 12.43±1.07 |  |  |
|  | Control | 12.24±1.81 |  | ＜0.001 |
|  | ISO | 18.47±2.56 |  |  |
|  | Control | 12.24±1.81 |  | 0.474 |
|  | ISO+gatrin | 13.95±1.32 |  |  |
|  | Control | 12.24±1.81 |  | ＜0.001 |
|  | ISO+gatrin+CI988 | 18.36±2.43 |  |  |
|  | ISO | 18.47±2.56 |  | 0.001 |
|  | ISO+gastrin | 13.95±1.32 |  |  |
|  | ISO+gastrin | 13.95±1.32 |  | 0.002 |
|  | ISO+gastrin+CI988 | 18.36±2.43 |  |  |

**
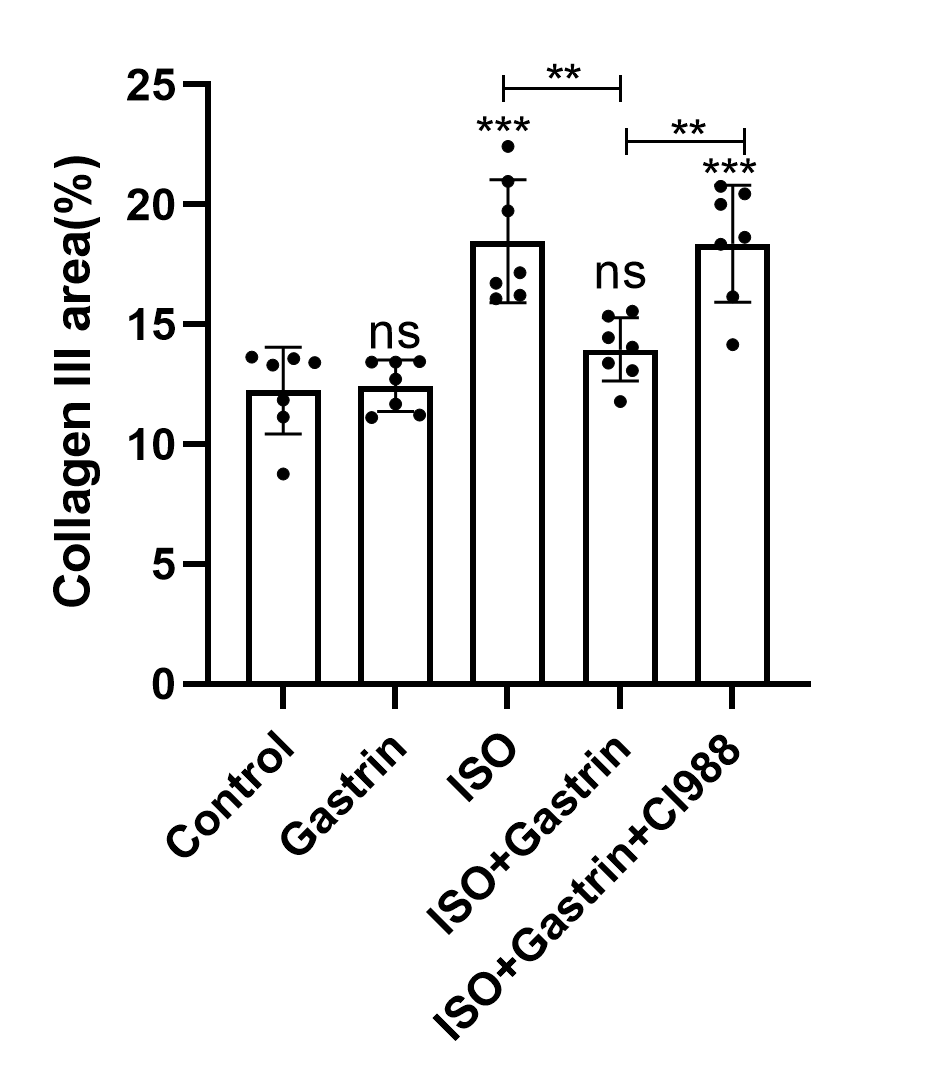
**

**The percentage of Collagen III of five groups**

**Fig 4.(B)The expression level of collagen I in myocardial tissues of mice in the Control, Gastrin, ISO, and ISO+Gastrin groups**

Table 35 Quantitative analysis of collagen I expression in mouse hearts using the 2^-ΔΔCT^ method

| Collagen I /18S  (2^-ΔΔCT^) | Control | Gastrin | ISO | ISO+Gastrin |
| --- | --- | --- | --- | --- |
| Group1 | 1 | 1.92 | 5.36 | 2.65 |
| Group2 | 1 | 1.14 | 6.16 | 2.54 |
| Group3 | 1 | 2.06 | 5.22 | 2.35 |
| Group4 | 1 | 1.47 | 4.43 | 1.46 |
| Group5 | 1 | 1.01 | 6.92 | 2.14 |
| Group6 | 1 | 1.03 | 3.51 | 1.22 |

Table36 The comparison of collagen I across each group

|  | groups | Collagen I(2^-ΔΔCT^)  Mean±SD | F-value | P-value |
| --- | --- | --- | --- | --- |
| ANOVA |  |  | 44.267 | ＜0.001 |
| Multiple comparisons | Control | 1 |  | 0.715 |
|  | Gastrin | 1.44±0.46 |  |  |
|  | Control | 1 |  | ＜0.001 |
|  | ISO | 5.27±1.21 |  |  |
|  | Control | 1 |  | 0.078 |
|  | ISO+gatrin | 2.06±0.59 |  |  |
|  | ISO | 5.27±1.21 |  | ＜0.001 |
|  | ISO+gastrin | 2.06±0.59 |  |  |


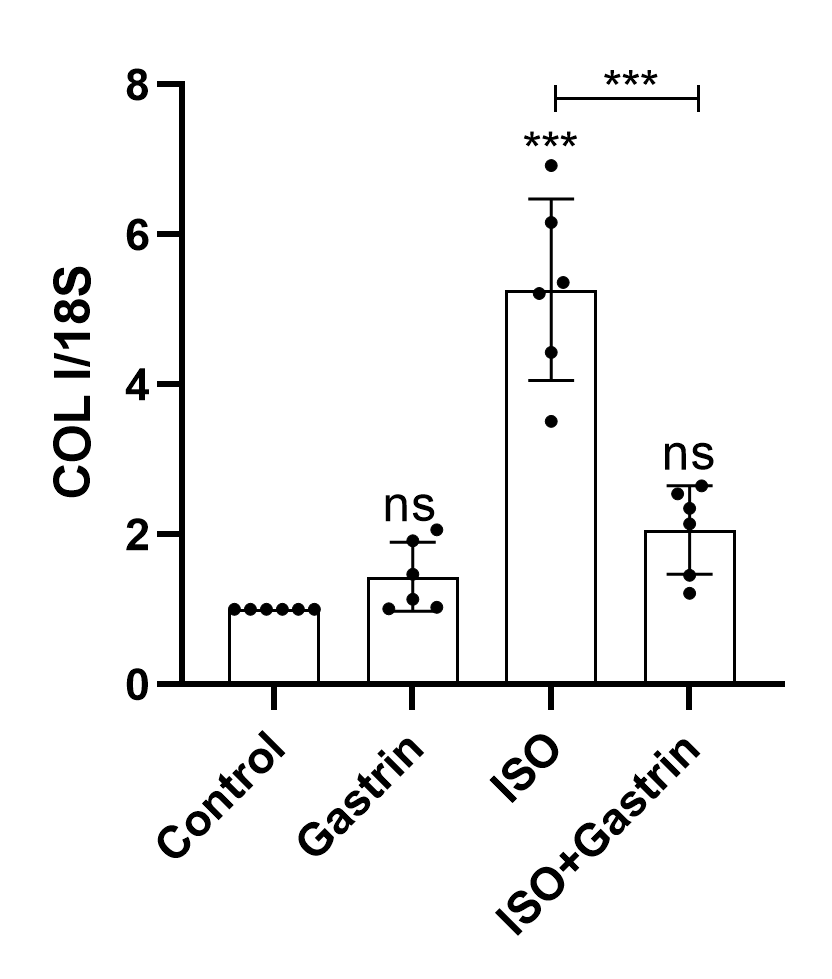


**The Collagen I expression of the heart tissues**

**Fig 4.(C)The expression level of collagen III in myocardial tissues of mice in the Control, Gastrin, ISO, and ISO+Gastrin groups**

Table 37 Quantitative analysis of collagen III expression in mouse hearts using the 2^-ΔΔCT^ method

| Collagen III /18S  (2^-ΔΔCT^) | Control | Gastrin | ISO | ISO+Gastrin |
| --- | --- | --- | --- | --- |
| Group1 | 1 | 1.02 | 3.34 | 1.38 |
| Group2 | 1 | 1.13 | 3.65 | 1.67 |
| Group3 | 1 | 1.49 | 3.99 | 1.44 |
| Group4 | 1 | 1.06 | 3.08 | 1.82 |
| Group5 | 1 | 1.68 | 4.82 | 1.95 |
| Group6 | 1 | 1.08 | 3.2 | 1.23 |

Table38 The comparison of collagen III across each group

|  | groups | Collagen III(2^-ΔΔCT^)  Mean±SD | F-value | P-value |
| --- | --- | --- | --- | --- |
| ANOVA |  |  | 63.290 | ＜0.001 |
| Multiple comparisons | Control | 1 |  | 0.689 |
|  | Gastrin | 1.24±0.27 |  |  |
|  | Control | 1 |  | ＜0.001 |
|  | ISO | 3.68±0.65 |  |  |
|  | Control | 1 |  | 0.067 |
|  | ISO+gatrin | 1.58±0.28 |  |  |
|  | ISO | 3.68±0.65 |  | ＜0.001 |
|  | ISO+gastrin | 1.58±0.28 |  |  |


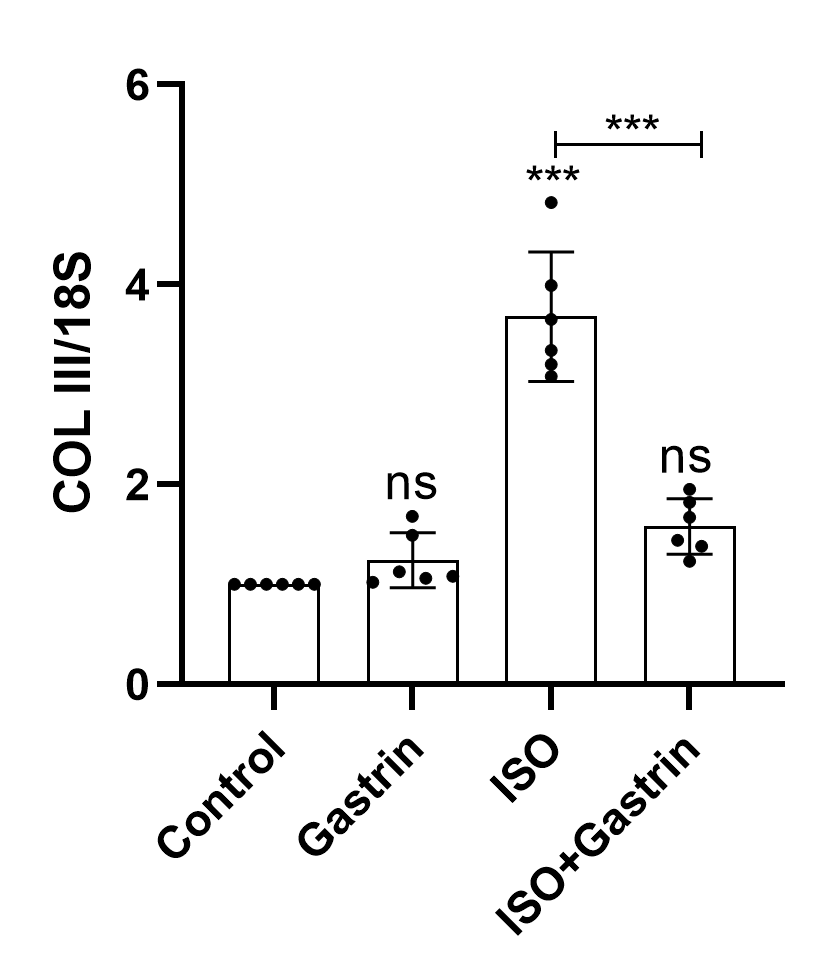


**The Collagen III expression of the heart tissues**

**Fig 4.(D)The expression level of α-SMA in myocardial tissues of mice in the Control, Gastrin, ISO, and ISO+Gastrin groups**

Table 39 Quantitative analysis of α-SMA expression in mouse hearts using the 2^-ΔΔCT^ method

| α-SMA/18S  (2^-ΔΔCT^) | Control | Gastrin | ISO | ISO+Gastrin |
| --- | --- | --- | --- | --- |
| Group1 | 1 | 1.21 | 3.82 | 1.77 |
| Group2 | 1 | 1.2 | 3.81 | 1.33 |
| Group3 | 1 | 1.78 | 5.1 | 1.76 |
| Group4 | 1 | 1.34 | 5.58 | 1.92 |
| Group5 | 1 | 1.44 | 5.58 | 1.99 |
| Group6 | 1 | 1.25 | 4.5 | 1.36 |

Table40 The comparison of α-SMA across each group

|  | groups | α-SMA(2^-ΔΔCT^)  Mean±SD | F-value | P-value |
| --- | --- | --- | --- | --- |
| ANOVA |  |  | 89.425 | ＜0.001 |
| Multiple comparisons | Control | 1 |  | 0.485 |
|  | Gastrin | 1.37±0.22 |  |  |
|  | Control | 1 |  | ＜0.001 |
|  | ISO | 4.73±0.81 |  |  |
|  | Control | 1 |  | 0.062 |
|  | ISO+gatrin | 1.69±0.28 |  |  |
|  | ISO | 4.73±0.81 |  | ＜0.001 |
|  | ISO+gastrin | 1.69±0.28 |  |  |


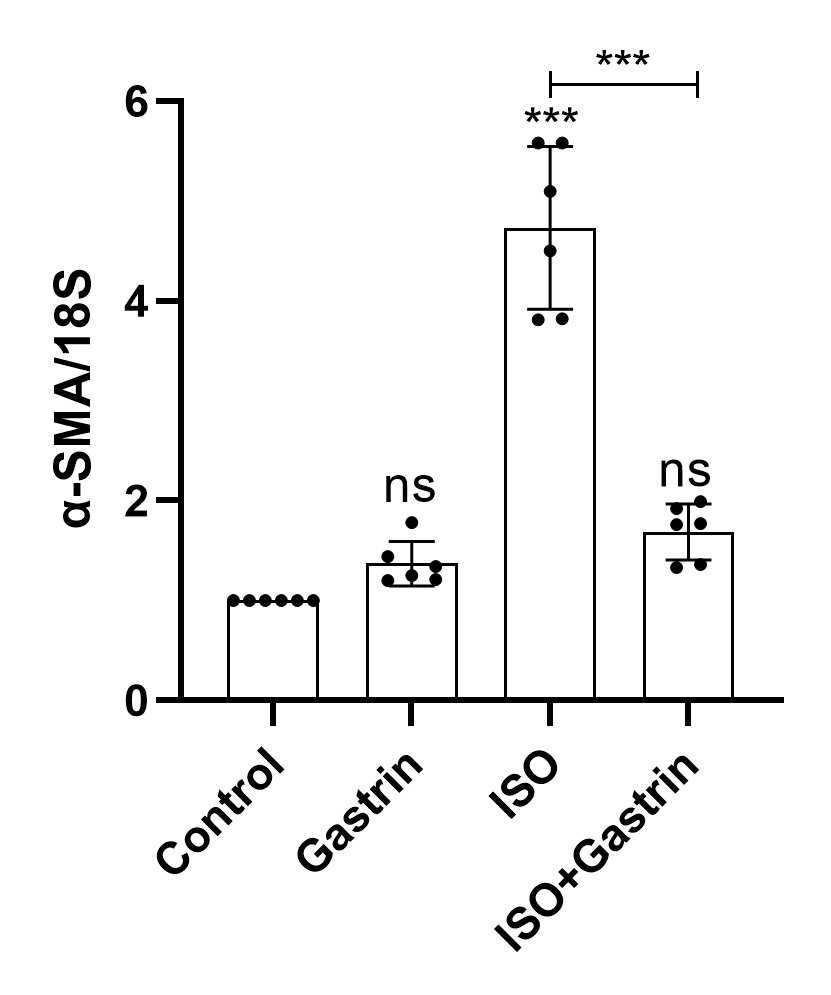


**The α-SMA expression of the heart tissues**

**Fig 4. (E)The expression level of collagen I in myocardial tissues of mice in the Control, ISO, ISO+Gastrin and ISO+Gastrin+CI-988 groups**

Table 41 Quantitative analysis of Collagen I expression in mouse hearts using the 2^-ΔΔCT^ method

| Collagen I /18S(2^-ΔΔCT^) | Control | ISO | ISO+Gastrin | ISO+Gastrin+CI988 |
| --- | --- | --- | --- | --- |
| Group1 | 1 | 3.35 | 1.15 | 2.79 |
| Group2 | 1 | 5.25 | 1.58 | 2.92 |
| Group3 | 1 | 4.04 | 1.38 | 3.14 |
| Group4 | 1 | 3.86 | 2.05 | 3.68 |
| Group5 | 1 | 3.75 | 1.4 | 3.04 |
| Group6 | 1 | 5.65 | 2.12 | 4.61 |

Table42 The comparison of ANP across each group

|  | groups | Collagen I(2^-ΔΔCT^)  Mean±SD | F-value | P-value |
| --- | --- | --- | --- | --- |
| ANOVA |  |  | 38.726 | ＜0.001 |
| Multiple comparisons | Control | 1 |  | ＜0.001 |
|  | ISO | 4.32±0.92 |  |  |
|  | Control | 1 |  | 0.326 |
|  | ISO+gatrin | 1.61±0.39 |  |  |
|  | Control | 1 |  | ＜0.001 |
|  | ISO+gatrin+CI988 | 3.36±0.68 |  |  |
|  | ISO | 4.32±0.92 |  | ＜0.001 |
|  | ISO+gastrin | 1.61±0.39 |  |  |
|  | ISO+gastrin | 1.61±0.39 |  | ＜0.001 |
|  | ISO+gatrin+CI988 | 3.36±0.68 |  |  |


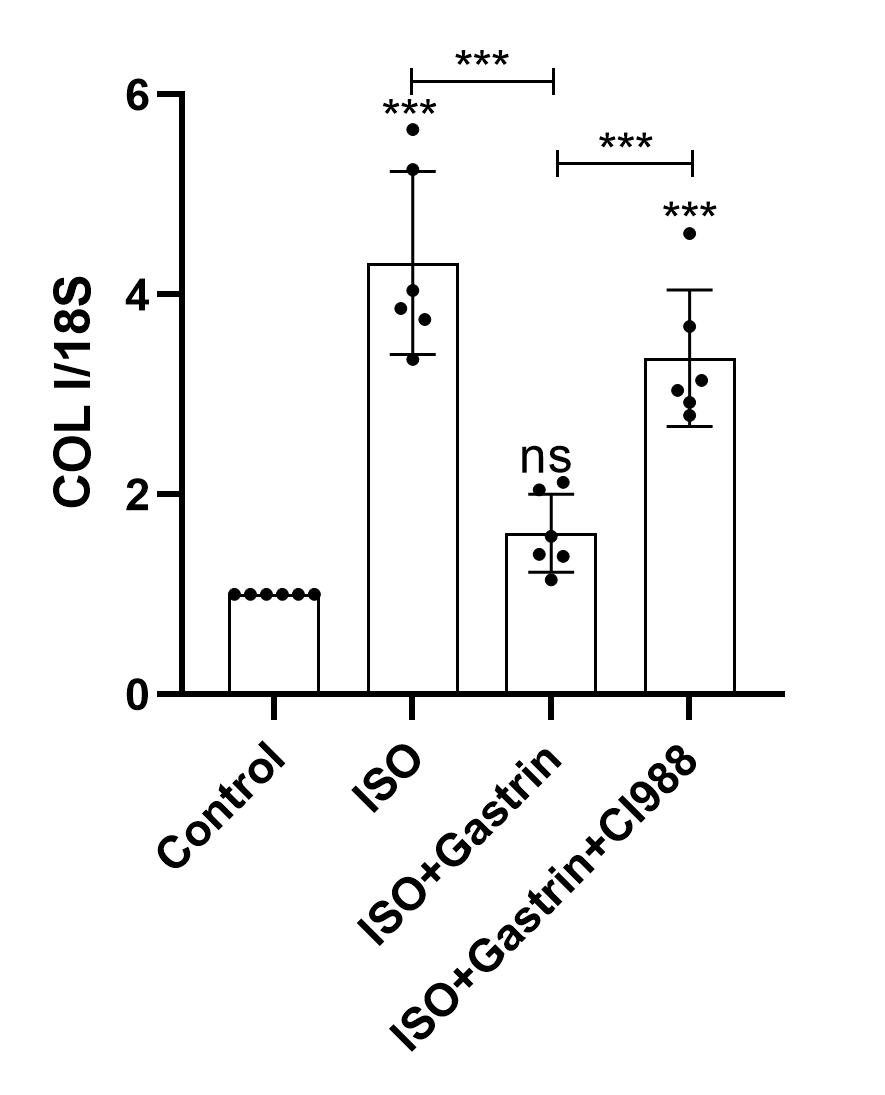


**The Collagen I expression of the heart tissues**

**Fig 4. (F)The expression level of collagen III in myocardial tissues of mice in the Control, ISO, ISO+Gastrin and ISO+Gastrin+CI-988 groups**

Table 43 Quantitative analysis of Collagen III expression in mouse hearts using the 2^-ΔΔCT^ method

| CollagenIII /18S(2^-ΔΔCT^) | Control | ISO | ISO+Gastrin | ISO+Gastrin+CI988 |
| --- | --- | --- | --- | --- |
| Group1 | 1 | 4.38 | 1.04 | 3.19 |
| Group2 | 1 | 6.46 | 1.32 | 4.7 |
| Group3 | 1 | 4.21 | 1.23 | 3.12 |
| Group4 | 1 | 5.67 | 1.04 | 3.28 |
| Group5 | 1 | 5.26 | 1.14 | 3.35 |
| Group6 | 1 | 4.94 | 1.27 | 3.54 |

Table44 The comparison of Collagen III across each group

|  | groups | Collagen III  (2^-ΔΔCT^)  Mean±SD | F-value | P-value |
| --- | --- | --- | --- | --- |
| ANOVA |  |  | 89.489 | ＜0.001 |
| Multiple comparisons | Control | 1 |  | ＜0.001 |
|  | ISO | 5.15±0.84 |  |  |
|  | Control | 1 |  | 0.935 |
|  | ISO+gatrin | 1.17±0.12 |  |  |
|  | Control | 1 |  | ＜0.001 |
|  | ISO+gatrin+CI988 | 3.53±0.59 |  |  |
|  | ISO | 5.15±0.84 |  | ＜0.001 |
|  | ISO+gastrin | 1.17±0.12 |  |  |
|  | ISO+gastrin | 1.17±0.12 |  | ＜0.001 |
|  | ISO+gatrin+CI988 | 3.53±0.59 |  |  |


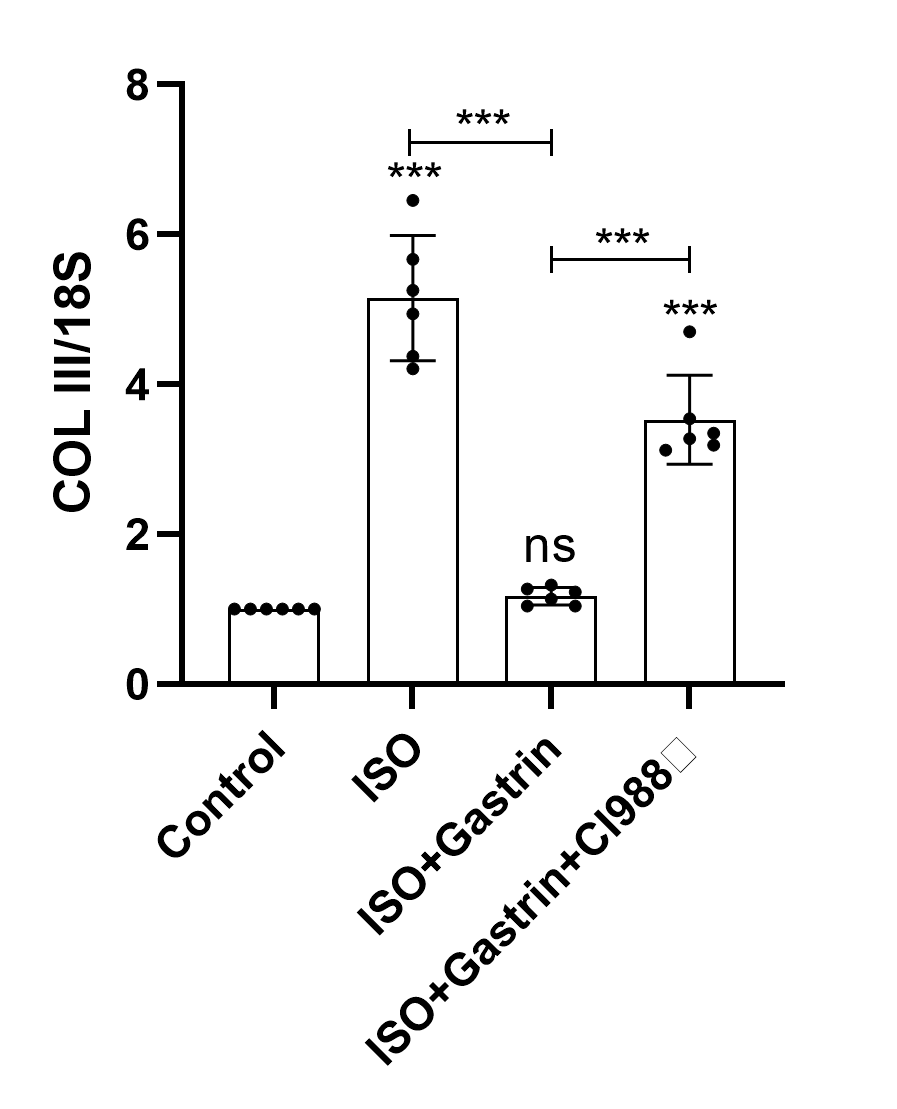


**The Collagen III expression of the heart tissues**

**Fig 4. (G)The expression level of α-SMA in myocardial tissues of mice in the Control, ISO, ISO+Gastrin, and ISO+Gastrin+CI-988 groups**

Table 45 Quantitative analysis of α-SMA expression in mouse hearts using the 2^-ΔΔCT^ method

| α-SMA /18S  (2^-ΔΔCT^) | Control | ISO | ISO+Gastrin | ISO+Gastrin+CI988 |
| --- | --- | --- | --- | --- |
| Group1 | 1 | 4.25 | 1.71 | 3.84 |
| Group2 | 1 | 5.59 | 1.86 | 4.29 |
| Group3 | 1 | 5.74 | 1.37 | 4.37 |
| Group4 | 1 | 4.83 | 1.5 | 4.44 |
| Group5 | 1 | 5.1 | 2.12 | 4.91 |
| Group6 | 1 | 6.66 | 1.85 | 4.36 |

Table46 The comparison of α-SMA across each group

|  | groups | α-SMA(2^-ΔΔCT^)  Mean±SD | F-value | P-value |
| --- | --- | --- | --- | --- |
| ANOVA |  |  | 117.159 | ＜0.001 |
| Multiple comparisons | Control | 1 |  | ＜0.001 |
|  | ISO | 5.36±0.83 |  |  |
|  | Control | 1 |  | 0.061 |
|  | ISO+gatrin | 1.73±0.27 |  |  |
|  | Control | 1 |  | ＜0.001 |
|  | ISO+gatrin+CI988 | 4.37±0.34 |  |  |
|  | ISO | 5.36±0.83 |  | ＜0.001 |
|  | ISO+gastrin | 1.73±0.27 |  |  |
|  | ISO+gastrin | 1.73±0.27 |  | ＜0.001 |
|  | ISO+gatrin+CI988 | 4.37±0.34 |  |  |

**
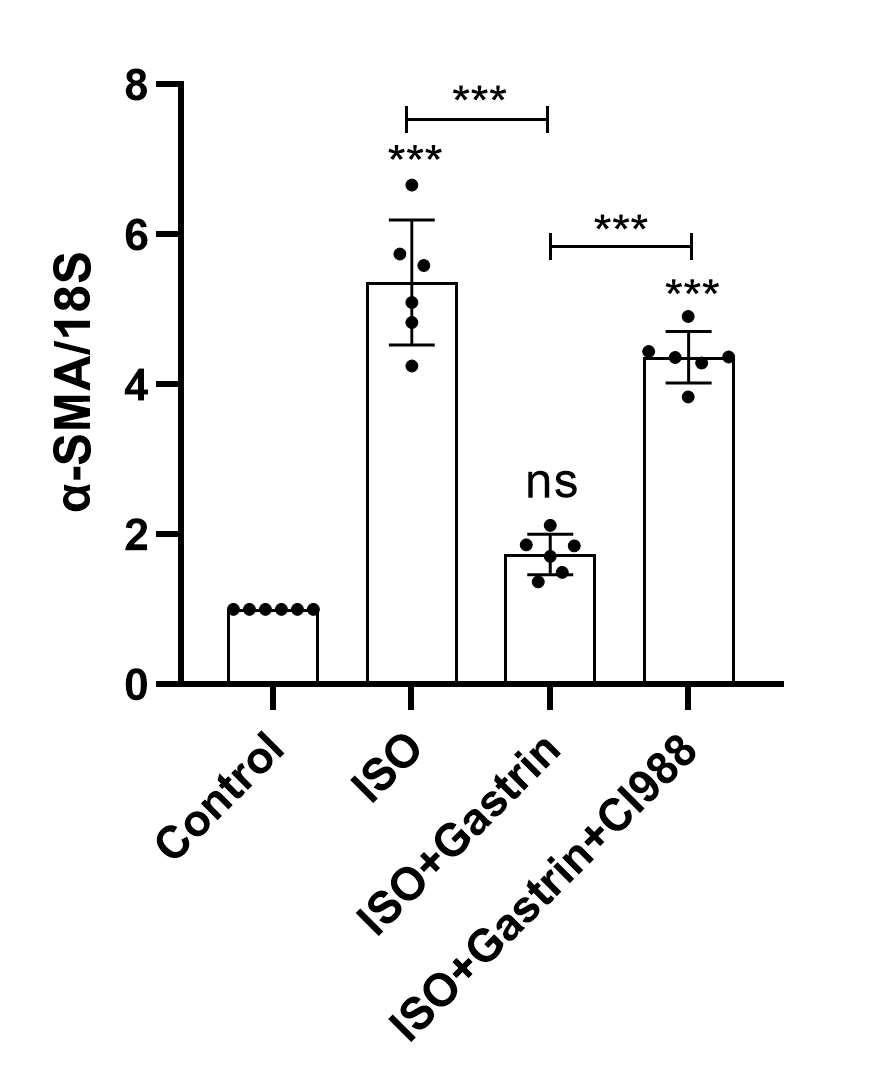
**

**The α-SMA expression of the heart tissues**

**Fig 5.(B)The level of p-JAK2/JAK2 in myocardial tissues of mice in the Control, Gastrin, ISO, and ISO+Gastrin groups**

Western blot analysis was performed on animal tissues in two separate batches. The Western blot results from one batch are presented in Figure 5(A), while the WB bands corresponding to p-JAK2 and total JAK2 from the second batch are shown below.

Fig S1 The WB bands of p-JAK2 and JAK2 from the second batch


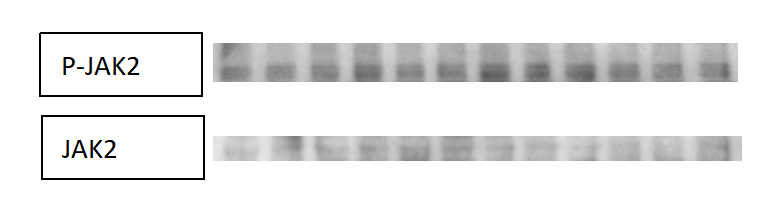


Table 47 The grayscale intensity of P-JAK2/JAK2 in Fig 5A

| groups | The grayscale intensity of JAK2 | The grayscale intensity of P-JAK2 | P-JAK2/JAK2 |
| --- | --- | --- | --- |
| Control | 2,365,972.00 | 3,065,845.00 | 1.295807812 |
|  | 3,536,747.00 | 3,568,190.00 | 1.008890373 |
|  | 3,520,534.00 | 3,768,809.00 | 1.070521972 |
| Gastrin | 3,927,091.00 | 4,373,997.00 | 1.113800775 |
|  | 4,408,459.00 | 4,610,364.00 | 1.045799451 |
|  | 4,694,151.00 | 4,790,134.00 | 1.020447361 |
| ISO | 3,444,086.00 | 6,033,264.00 | 1.751775072 |
|  | 3,152,486.00 | 5,685,640.00 | 1.803541713 |
|  | 2,555,394.00 | 4,565,523.00 | 1.786621946 |
| ISO+Gastrin | 3,130,886.00 | 4,736,381.00 | 1.512792545 |
|  | 2,750,102.00 | 4,123,461.00 | 1.49938475 |
|  | 4,254,382.00 | 4,412,774.00 | 1.037230319 |

Table 48 The comparison of P-JAK2/JAK2 across each group in Fig 5A

|  | groups | P-JAK2/JAK2  Mean±SD | F-value | P-value |
| --- | --- | --- | --- | --- |
| ANOVA |  |  | 12.842 | 0.002 |
| Multiple comparisons | Control | 1.13±0.15 |  | 0.955 |
|  | Gastrin | 1.06±0.48 |  |  |
|  | Control | 1.13±0.15 |  | 0.004 |
|  | ISO | 1.78±0.26 |  |  |
|  | Control | 1.13±0.15 |  | 0.362 |
|  | ISO+gatrin | 1.35±0.27 |  |  |
|  | ISO | 1.78±0.26 |  | 0.041 |
|  | ISO+gastrin | 1.35±0.27 |  |  |

Table 49 The grayscale intensity of P-JAK2/JAK2 of the second batch

| groups | The grayscale intensity of JAK2 | The grayscale intensity of P-JAK2 | P-JAK2/JAK2 |
| --- | --- | --- | --- |
| Control | 2,540,802.00 | 4,527,317.00 | 1.781845653 |
|  | 2,331,976.00 | 4,030,938.00 | 1.72855038 |
|  | 2,673,751.00 | 4,344,262.00 | 1.624781814 |
| Gastrin | 2,579,770.00 | 4,521,601.00 | 1.752714777 |
|  | 2,086,557.00 | 4,196,311.00 | 2.011117357 |
|  | 2,003,864.00 | 4,242,480.00 | 2.117149667 |
| ISO | 2,275,858.00 | 6,155,368.00 | 2.70463623 |
|  | 2,279,930.00 | 5,693,687.00 | 2.497307812 |
|  | 2,446,372.00 | 5,760,259.00 | 2.354612872 |
| ISO+Gastrin | 2,098,980.00 | 4,368,632.00 | 2.081311875 |
|  | 2,410,441.00 | 4,408,835.00 | 1.829057421 |
|  | 3,601,953.00 | 4,566,360.00 | 1.267745581 |

Table 50 The comparison of P-JAK2/JAK2 in the second batch

|  | groups | P-JAK2/JAK2  Mean±SD | F-value | P-value |
| --- | --- | --- | --- | --- |
| ANOVA |  |  | 6.949 | 0.013 |
| Multiple comparisons | Control | 1.71±0.08 |  | 0.628 |
|  | Gastrin | 1.96±0.19 |  |  |
|  | Control | 1.71±0.08 |  | 0.017 |
|  | ISO | 2.52±0.18 |  |  |
|  | Control | 1.71±0.08 |  | 1.000 |
|  | ISO+gatrin | 1.73±0.42 |  |  |
|  | ISO | 2.52±0.18 |  | 0.019 |
|  | ISO+gastrin | 1.73±0.42 |  |  |


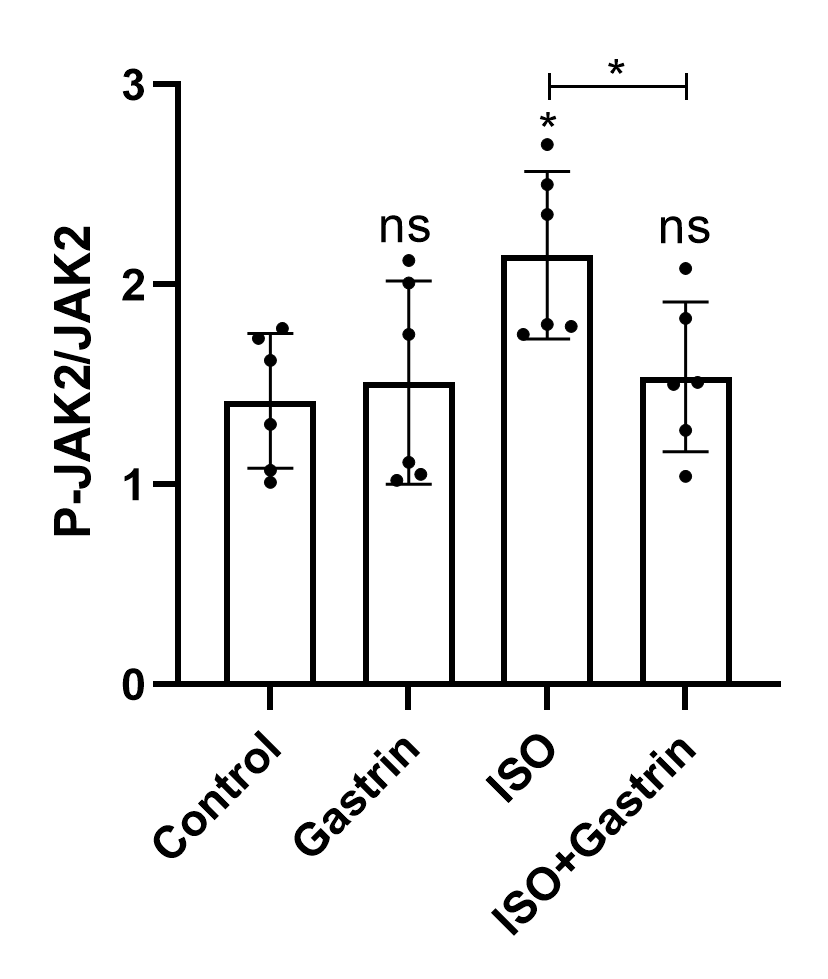


**The level of p-JAK2/JAK2**

**Fig 5.(C)The level of p-STAT3/STAT3 in myocardial tissues of mice in the Control, Gastrin, ISO, and ISO+Gastrin groups**

The P-STAT3 and STAT3 protein bands derived from the first batch of animal tissues are presented in Figure 5(A), while those from the second batch are shown below.

Fig S2 The WB bands of P-STAT3 and STAT3 from the second batch


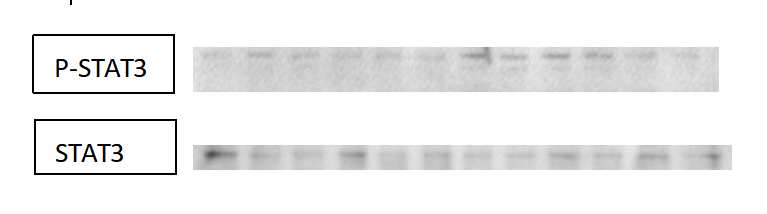


Table 51 The grayscale intensity of P-STAT3/STAT3 in Fig 5(A)

| groups | The grayscale intensity of STAT3 | The grayscale intensity of P-STAT3 | P-STAT3/STAT3 |
| --- | --- | --- | --- |
| Control | 1,565,126.00 | 2,290,960.00 | 1.463754356 |
|  | 1,576,158.00 | 3,783,891.00 | 2.400705386 |
|  | 1,642,912.00 | 3,125,805.00 | 1.902600383 |
| Gastrin | 1,415,539.00 | 4,350,901.00 | 2.693157516 |
|  | 2,207,658.00 | 5,160,117.00 | 2.337371549 |
|  | 1,986,813.00 | 4,347,534.00 | 2.188194863 |
| ISO | 1,408,602.00 | 6,067,889.00 | 4.307738453 |
|  | 1,955,549.00 | 5,843,028.00 | 2.987922062 |
|  | 1,942,801.00 | 7,596,442.00 | 3.910046371 |
| ISO+Gastrin | 2,128,713.00 | 4,518,116.00 | 2.122463667 |
|  | 1,659,536.00 | 3,110,233.00 | 1.874158198 |
|  | 1,926,839.00 | 2,841,518.00 | 1.474704425 |

Table 52 The grayscale intensity of P-STAT3/STAT3 of the second batch

| groups | The grayscale intensity of STAT3 | The grayscale intensity of P-STAT3 | P-STAT3/STAT3 |
| --- | --- | --- | --- |
| Control | 197,051.00 | 335,398.00 | 1.702087277 |
|  | 139,512.00 | 422,578.00 | 3.028972418 |
|  | 113,307.00 | 454,882.00 | 4.014597509 |
| Gastrin | 199,347.00 | 445,328.00 | 2.233933794 |
|  | 113,626.00 | 438,854.00 | 3.862267439 |
|  | 123,173.00 | 368,532.00 | 2.99198688 |
| ISO | 131,881.00 | 748,175.00 | 5.673106816 |
|  | 101,895.00 | 568,320.00 | 5.577506256 |
|  | 110,448.00 | 588,722.00 | 5.330309286 |
| ISO+Gastrin | 122,580.00 | 482,750.00 | 3.938244412 |
|  | 184,241.00 | 378,358.00 | 2.053603704 |
|  | 198,390.00 | 251,125.00 | 1.265814809 |

Table 53 The comparison of P-STAT3/STAT3 across each group

|  | groups | P-STAT3/STAT3Mean±SD | F-value | P-value |
| --- | --- | --- | --- | --- |
| ANOVA |  |  | 10.900 | 0.003 |
| Multiple comparisons | Control | 1.92±0.47 |  | 0.597 |
|  | Gastrin | 2.41±0.26 |  |  |
|  | Control | 1.92±0.47 |  | 0.003 |
|  | ISO | 3.73±0.68 |  |  |
|  | Control | 1.92±0.47 |  | 0.993 |
|  | ISO+gatrin | 1.82±0.33 |  |  |
|  | ISO | 3.73±0.68 |  | 0.004 |
|  | ISO+gastrin | 1.82±0.33 |  |  |


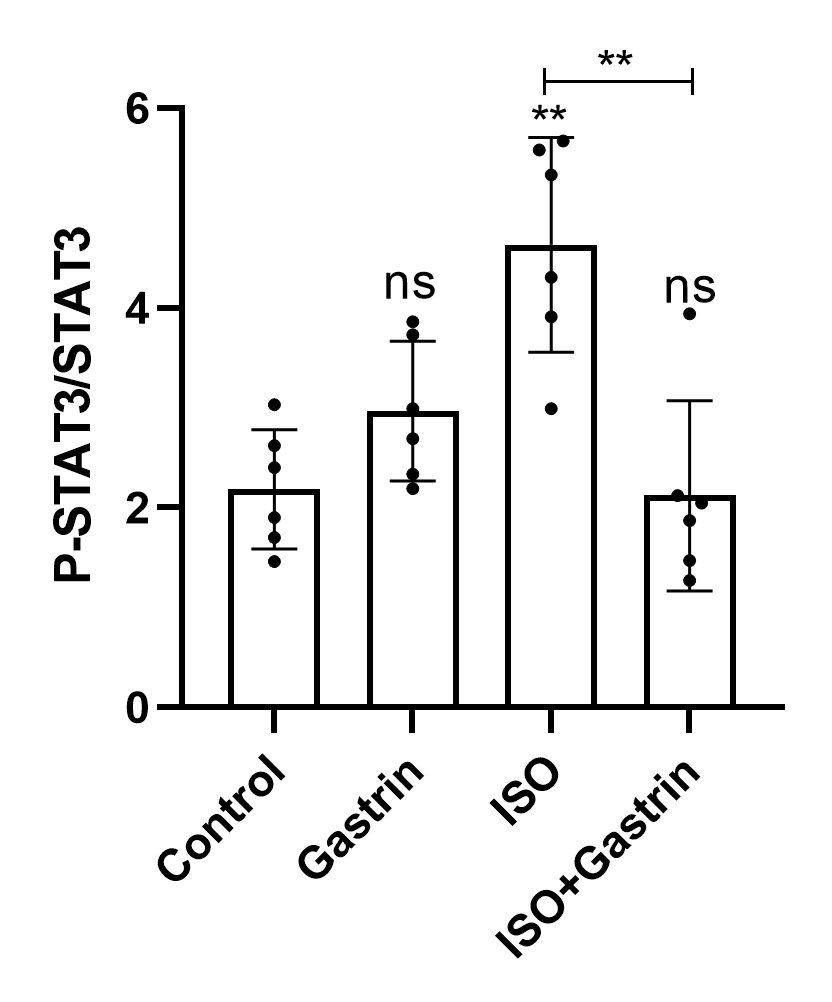


**The level of p-STAT3/STAT3**

**Fig 5. (D)The level of p-ERK/ERK in myocardial tissues of mice in the Control, Gastrin, ISO, and ISO+Gastrin groups**

The P-ERK and ERK protein bands derived from the first batch of animal tissues are presented in Figure 5(A), while those from the second batch are shown below.

Fig S3 The WB bands of P-ERK and ERK from the second batch


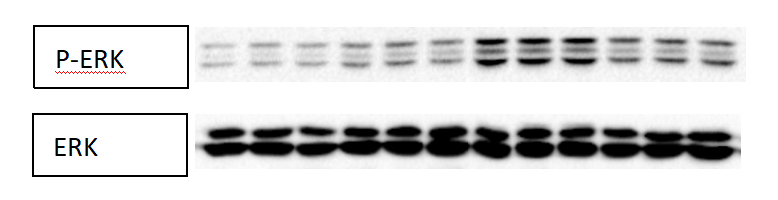


Table 54 The grayscale intensity of P-ERK/ERK in Fig 5(A)

| groups | The grayscale intensity of ERK | The grayscale intensity of P-ERK | P-ERK/ERK |
| --- | --- | --- | --- |
| Control | 28,279,512.00 | 14,908,520.00 | 0.527184486 |
|  | 26,195,916.00 | 12,074,235.00 | 0.460920511 |
|  | 24,712,475.00 | 12,473,824.00 | 0.504758184 |
| Gastrin | 22,701,900.00 | 12,022,991.00 | 0.529602853 |
|  | 25,415,754.00 | 12,616,530.00 | 0.496405891 |
|  | 27,797,923.00 | 17,252,365.00 | 0.620635038 |
| ISO | 23,181,071.00 | 19,145,860.00 | 0.825926464 |
|  | 22,267,942.00 | 19,405,218.00 | 0.871441914 |
|  | 27,020,964.00 | 19,777,867.00 | 0.731945278 |
| ISO+Gastrin | 28,018,944.00 | 14,349,355.00 | 0.512130471 |
|  | 24,800,322.00 | 10,392,461.00 | 0.419045406 |
|  | 22,484,073.00 | 15,665,155.00 | 0.696722298 |

Table 55 The grayscale intensity of P-ERK/ERK of second batch

| groups | The grayscale intensity of ERK | The grayscale intensity of P-ERK | P-ERK/ERK |
| --- | --- | --- | --- |
| Control | 23,841,786.00 | 7,336,600.00 | 0.307720235 |
|  | 25,382,935.00 | 10,394,380.00 | 0.409502684 |
|  | 21,958,298.00 | 9,850,430.00 | 0.448597154 |
| Gastrin | 25,024,514.00 | 15,160,910.00 | 0.605842335 |
|  | 25,432,976.00 | 18,570,700.00 | 0.73018195 |
|  | 28,887,820.00 | 18,048,160.00 | 0.624767116 |
| ISO | 25,947,419.00 | 43,395,800.00 | 1.672451507 |
|  | 26,203,313.00 | 43,827,680.00 | 1.672600713 |
|  | 26,585,660.00 | 42,551,220.00 | 1.600532768 |
| ISO+Gastrin | 24,945,878.00 | 24,498,600.00 | 0.982070064 |
|  | 26,384,846.00 | 25,520,470.00 | 0.96723968 |
|  | 28,379,655.00 | 25,165,080.00 | 0.886729596 |

Table 56 The comparison of P-ERK/ERK across each group

|  | groups | 1. ERK/ERK 2. Mean±SD | F-value | P-value |
| --- | --- | --- | --- | --- |
| ANOVA |  |  | 9.731 | ＜0.001 |
| Multiple comparisons | Control | 0.44±0.08 |  | 0.736 |
|  | Gastrin | 0.60±0.08 |  |  |
|  | Control | 0.44±0.08 |  | ＜0.001 |
|  | ISO | 1.23±0.46 |  |  |
|  | Control | 0.44±0.08 |  | 0.238 |
|  | ISO+gatrin | 0.74±0.24 |  |  |
|  | ISO | 1.23±0.46 |  | 0.024 |
|  | ISO+gastrin | 0.74±0.24 |  |  |


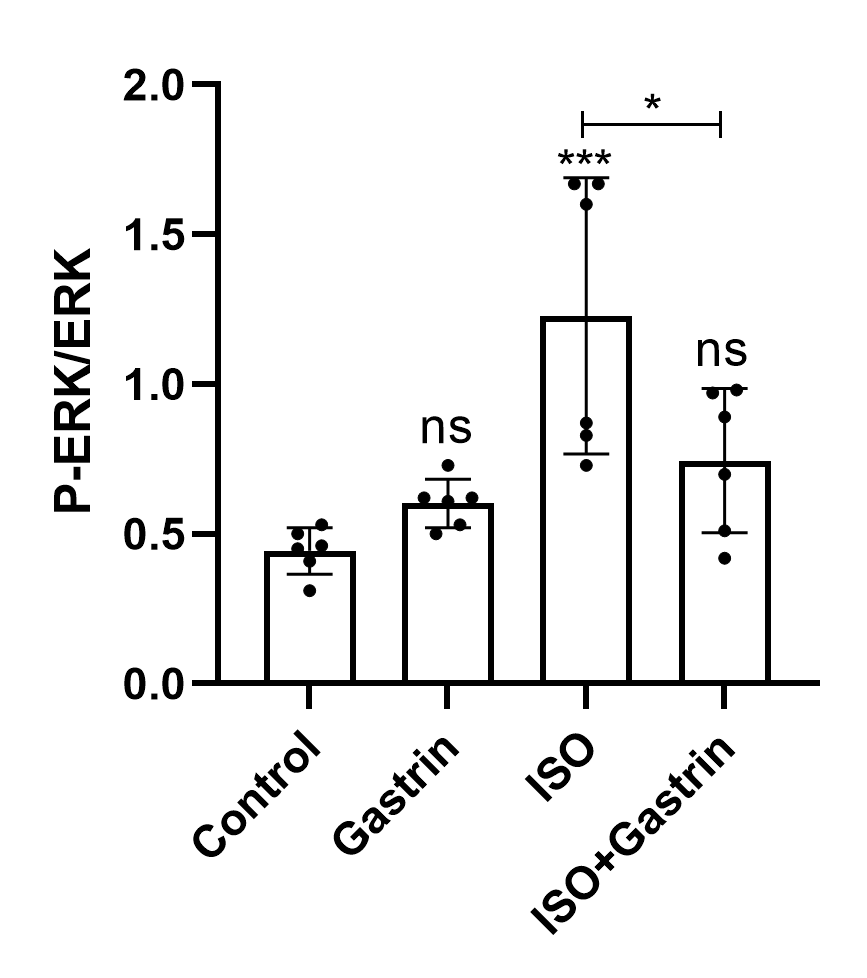


**The level of p-ERK/ERK**

**Fig 5.(F)The level of p-JAK2/JAK2 in myocardial tissues of mice in the Control, ISO, and ISO+Gastrin and ISO+Gastrin+CI988 groups**

The P-JAK2 and JAK2 protein bands derived from the first batch of animal tissues are presented in Figure 5(E), while those from the second batch are shown below.

Fig S4 The WB bands of P-JAK2 and JAK2 from the second batch


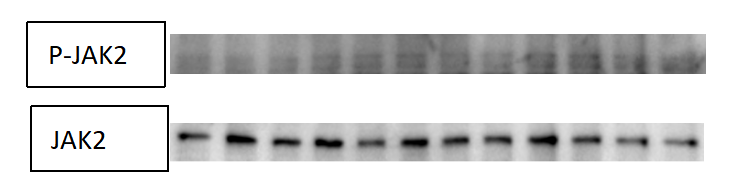


Table 57 The grayscale intensity of P-JAK2/JAK2 in Fig 5(E)

| groups | The grayscale intensity of JAK2 | The grayscale intensity of P-JAK2 | P-JAK2/JAK2 |
| --- | --- | --- | --- |
| Control | 5,883,277.00 | 670,114.00 | 0.113901487 |
|  | 7,557,136.00 | 942,770.00 | 0.124752287 |
|  | 5,888,476.00 | 1,437,126.00 | 0.244057376 |
| ISO | 3,325,747.00 | 2,420,827.00 | 0.727904738 |
|  | 3,338,542.00 | 1,752,823.00 | 0.525026494 |
|  | 3,094,922.00 | 2,293,592.00 | 0.741082328 |
| ISO+Gastrin | 5,076,372.00 | 1,038,565.00 | 0.20458804 |
|  | 4,186,824.00 | 798,189.00 | 0.190643075 |
|  | 6,461,169.00 | 1,103,655.00 | 0.170813517 |
| ISO+Gastrin+CI988 | 2,906,804.00 | 1,357,282.00 | 0.466932755 |
|  | 2,192,812.00 | 1,124,056.00 | 0.51260938 |
|  | 2,898,512.00 | 1,106,124.00 | 0.381617878 |

Table 58 The comparison of P-JAK2/JAK2 across each group of Fig 5(E)

|  | groups | P-JAK2/JAK2  Mean±SD | F-value | P-value |
| --- | --- | --- | --- | --- |
| ANOVA |  |  | 27.716 | ＜0.001 |
| Multiple comparisons | Control | 0.16±0.07 |  | ＜0.001 |
|  | ISO | 0.66±0.12 |  |  |
|  | Control | 0.16±0.07 |  | 0.971 |
|  | ISO+gatrin | 0.19±0.02 |  |  |
|  | Control | 0.16±0.07 |  | 0.008 |
|  | ISO+gastrin+CI988 | 0.45±0.06 |  |  |
|  | ISO | 0.66±0.12 |  | ＜0.001 |
|  | ISO+gastrin | 0.19±0.02 |  |  |
|  | ISO+gastrin | 0.19±0.02 |  | 0.014 |
|  | ISO+gastrin+CI988 | 0.45±0.06 |  |  |

Table 59 The grayscale intensity of P-JAK2/JAK2 of the second batch

| groups | The grayscale intensity of JAK2 | The grayscale intensity of P-JAK2 | P-JAK2/JAK2 |
| --- | --- | --- | --- |
| Control | 3,446,519.00 | 4,394,212.00 | 1.274971065 |
|  | 4,827,624.00 | 3,616,111.00 | 0.7490457 |
|  | 3,497,536.00 | 3,867,416.00 | 1.105754451 |
| ISO | 4,110,289.00 | 5,940,997.00 | 1.445396419 |
|  | 3,306,877.00 | 5,926,609.00 | 1.79220727 |
|  | 4,235,312.00 | 6,429,455.00 | 1.518059354 |
| ISO+Gastrin | 4,007,319.00 | 4,508,057.00 | 1.124955862 |
|  | 3,614,297.00 | 4,129,409.00 | 1.142520662 |
|  | 4,969,529.00 | 4,519,586.00 | 0.909459629 |
| ISO+Gastrin+CI988 | 3,523,715.00 | 5,500,345.00 | 1.560950588 |
|  | 2,970,355.00 | 5,280,464.00 | 1.777721518 |
|  | 2,956,176.00 | 5,674,794.00 | 1.919640103 |

Table 60 The comparison of P-JAK2/JAK2 across each group of the second batch

|  | groups | P-JAK2/JAK2  Mean±SD | F-value | P-value |
| --- | --- | --- | --- | --- |
| ANOVA |  |  | 10.217 | 0.004 |
| Multiple comparisons | Control | 1.04±0.27 |  | 0.039 |
|  | ISO | 1.58±0.18 |  |  |
|  | Control | 1.04±0.27 |  | 1.000 |
|  | ISO+gatrin | 1.06±0.13 |  |  |
|  | Control | 1.04±0.27 |  | 0.01 |
|  | ISO+gastrin+CI988 | 1.75±0.18 |  |  |
|  | ISO | 1.58±0.18 |  | 0.045 |
|  | ISO+gastrin | 1.06±0.13 |  |  |
|  | ISO+gastrin | 1.06±0.13 |  | 0.011 |
|  | ISO+gastrin+CI988 | 1.75±0.18 |  |  |


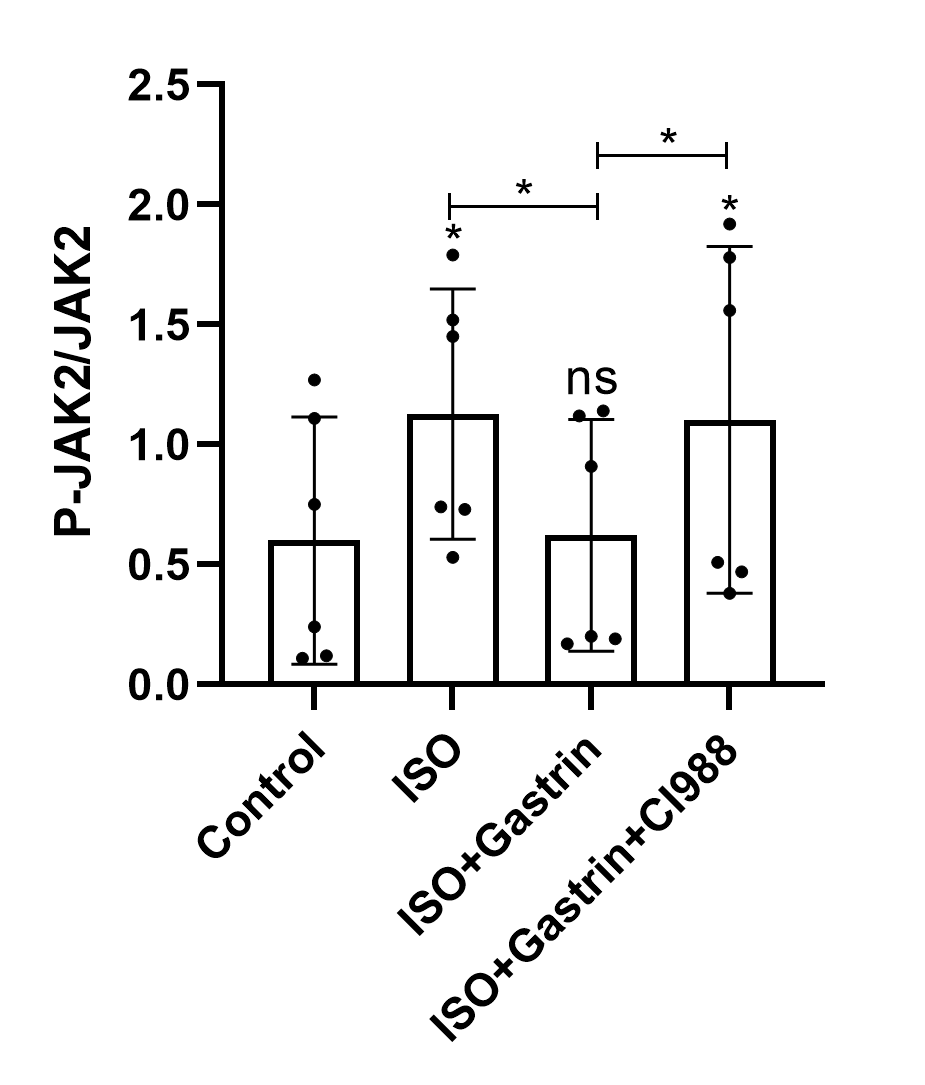


**The level of p-JAK2/JAK2**

**Fig 5.(G)The level of P-STAT3/STAT3 in myocardial tissues of mice in the Control, ISO, and ISO+Gastrin and ISO+Gastrin+CI988 groups**

The P-STAT3 and STAT3 protein bands derived from the first batch of animal tissues are presented in Figure 5(E), while those from the second batch are shown below.

Fig S5 The WB bands of P-STAT3 and STAT3 from the second batch


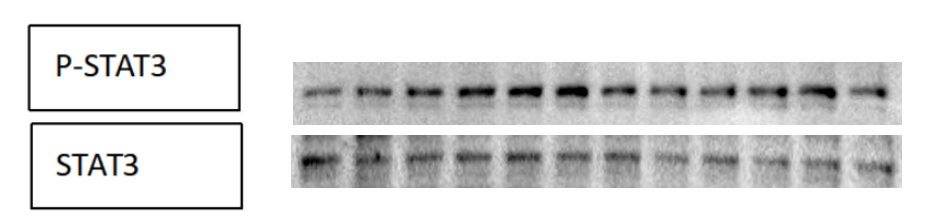


Table 61 The grayscale intensity of P-STAT3/STAT3 in Fig 5(E)

| groups | The grayscale intensity of STAT3 | The grayscale intensity of P-STAT3 | P-STAT3/STAT3 |
| --- | --- | --- | --- |
| Control | 2,892,061.00 | 3,632,566.00 | 1.256047504 |
|  | 3,317,210.00 | 4,495,008.00 | 1.35505681 |
|  | 3,319,049.00 | 4,615,429.00 | 1.390587786 |
| ISO | 3,885,620.00 | 7,199,433.00 | 1.852840216 |
|  | 3,509,806.00 | 5,991,133.00 | 1.706969844 |
|  | 3,328,521.00 | 5,535,686.00 | 1.663106827 |
| ISO+Gastrin | 3,058,521.00 | 3,168,731.00 | 1.036033756 |
|  | 2,488,740.00 | 3,665,128.00 | 1.472684169 |
|  | 3,014,268.00 | 4,473,689.00 | 1.48417095 |
| ISO+Gastrin+CI988 | 3,261,436.00 | 6,329,864.00 | 1.94082116 |
|  | 2,898,568.00 | 5,092,137.00 | 1.756776795 |
|  | 2,606,503.00 | 4,914,087.00 | 1.885317991 |

Table 62 The grayscale intensity of P-STAT3/STAT3 of the second batch

| groups | The grayscale intensity of STAT3 | The grayscale intensity of P-STAT3 | P-STAT3/STAT3 |
| --- | --- | --- | --- |
| Control | 3,183,380.00 | 5,262,359.42 | 1.653072965 |
|  | 4,565,771.00 | 3,980,543.00 | 0.871822744 |
|  | 3,199,555.00 | 3,785,192.00 | 1.183037016 |
| ISO | 1,838,439.00 | 4,743,033.00 | 2.579924055 |
|  | 2,818,197.00 | 5,804,655.00 | 2.059705194 |
|  | 2,669,692.00 | 7,949,300.00 | 2.977609402 |
| ISO+Gastrin | 2,897,320.00 | 4,628,841.00 | 1.597628498 |
|  | 3,288,969.00 | 4,322,267.00 | 1.314170793 |
|  | 3,804,383.00 | 3,581,665.00 | 0.941457524 |
| ISO+Gastrin+CI988 | 3,010,628.00 | 5,568,572.00 | 1.849638016 |
|  | 2,612,038.00 | 7,038,498.00 | 2.694638439 |
|  | 1,736,520.00 | 5,041,944.00 | 2.903475917 |

Table 63 The comparison of P-STAT3/STAT3 across each group

|  | groups | P-STAT3/STAT3  Mean±SD | F-value | P-value |
| --- | --- | --- | --- | --- |
| ANOVA |  |  | 8.957 | ＜0.001 |
| Multiple comparisons | Control | 1.28±0.26 |  | 0.008 |
|  | ISO | 2.14±0.53 |  |  |
|  | Control | 1.28±0.26 |  | 1.000 |
|  | ISO+gatrin | 1.31±0.26 |  |  |
|  | Control | 1.28±0.26 |  | 0.006 |
|  | ISO+gastrin+CI988 | 2.17±0.49 |  |  |
|  | ISO | 2.14±0.53 |  | 0.01 |
|  | ISO+gastrin | 1.31±0.26 |  |  |
|  | ISO+gastrin | 1.31±0.26 |  | 0.007 |
|  | ISO+gastrin+CI988 | 2.17±0.49 |  |  |


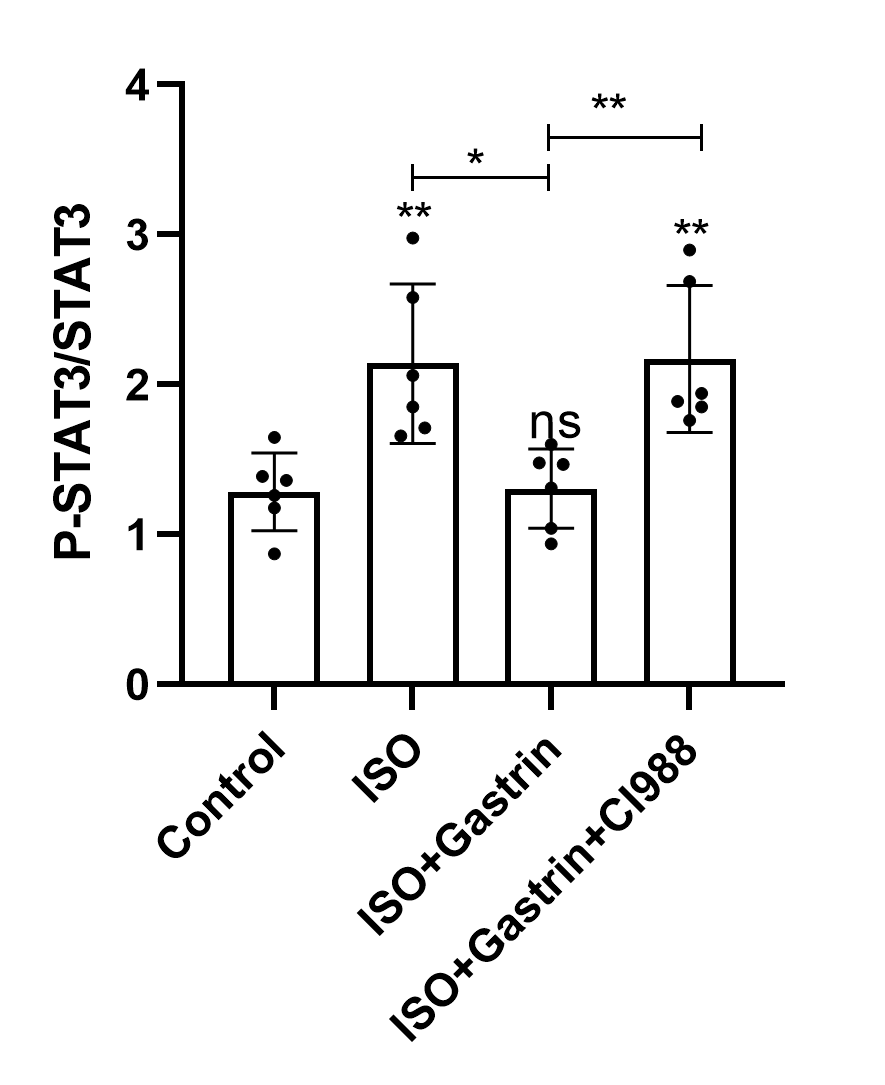


**The level of P-STAT3/STAT3**

**Fig 5.(H)The level of p-ERK/ERK in myocardial tissues of mice in the Control, ISO, and ISO+Gastrin and ISO+Gastrin+CI988 groups**

The P-ERK and ERK protein bands derived from the first batch of animal tissues are presented in Figure 5(E), while those from the second batch are shown below.

Fig S6 The WB bands of P-ERK and ERK from the second batch


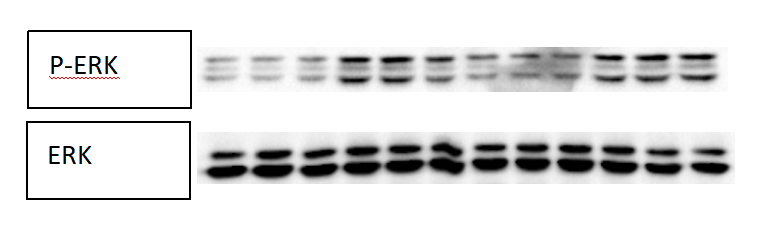


Table 64 The grayscale intensity of P-ERK/ERK in Fig5(E)

| groups | The grayscale intensity of ERK | The grayscale intensity of P-ERK | P-ERK/ERK |
| --- | --- | --- | --- |
| Control | 11,234,953.00 | 3,260,633.00 | 0.29022222 |
|  | 14,191,883.00 | 3,539,633.00 | 0.249412499 |
|  | 7,599,626.00 | 3,060,982.00 | 0.402780611 |
| ISO | 14,190,565.00 | 8,709,672.00 | 0.613764991 |
|  | 12,868,537.00 | 7,716,883.00 | 0.599670576 |
|  | 15,512,030.00 | 8,289,672.00 | 0.534402783 |
| ISO+Gastrin | 12,574,367.00 | 4,095,935.00 | 0.325736874 |
|  | 13,904,903.00 | 6,586,757.00 | 0.47370032 |
|  | 13,219,883.00 | 5,413,430.00 | 0.409491521 |
| ISO+Gastrin+CI988 | 12,144,334.00 | 6,211,224.00 | 0.51145036 |
|  | 13,904,687.00 | 8,710,209.00 | 0.626422515 |
|  | 11,516,307.00 | 7,033,872.00 | 0.610774965 |

Table 65 The grayscale intensity of P-ERK/ERK of the second batch

| groups | The grayscale intensity of ERK | The grayscale intensity of P-ERK | P-ERK/ERK*6 |
| --- | --- | --- | --- |
| Control | 18,998,237.00 | 1,205,969.57 | 0.380867837 |
|  | 22,070,784.00 | 1,161,767.14 | 0.315829418 |
|  | 19,921,382.00 | 1,388,985.00 | 0.418339953 |
| ISO | 22,145,638.00 | 3,205,670.86 | 0.868524318 |
|  | 22,137,403.00 | 3,089,385.29 | 0.837330003 |
|  | 22,139,295.00 | 2,945,870.43 | 0.798364292 |
| ISO+Gastrin | 19,009,831.00 | 1,441,007.00 | 0.454819509 |
|  | 20,011,077.00 | 1,428,469.57 | 0.428303655 |
|  | 21,599,663.00 | 1,662,776.29 | 0.461889508 |
| ISO+Gastrin+CI988 | 19,851,797.00 | 2,660,337.29 | 0.804059386 |
|  | 17,146,338.00 | 3,147,328.43 | 1.101341323 |
|  | 17,113,183.00 | 3,186,385.71 | 1.11716881 |

Table 66 The comparison of P-ERK/ERK across each group

|  | groups | P-ERK/ERK  Mean±SD | F-value | P-value |
| --- | --- | --- | --- | --- |
| ANOVA |  |  | 11.872 | ＜0.001 |
| Multiple comparisons | Control | 0.31±0.08 |  | 0.003 |
|  | ISO | 0.58±0.04 |  |  |
|  | Control | 0.31±0.08 |  | 0.792 |
|  | ISO+gatrin | 0.40±0.07 |  |  |
|  | Control | 0.31±0.08 |  | ＜0.001 |
|  | ISO+gastrin+CI988 | 0.58±0.06 |  |  |
|  | ISO | 0.58±0.04 |  | 0.023 |
|  | ISO+gastrin | 0.40±0.07 |  |  |
|  | ISO+gastrin | 0.40±0.07 |  | 0.003 |
|  | ISO+gastrin+CI988 | 0.58±0.06 |  |  |


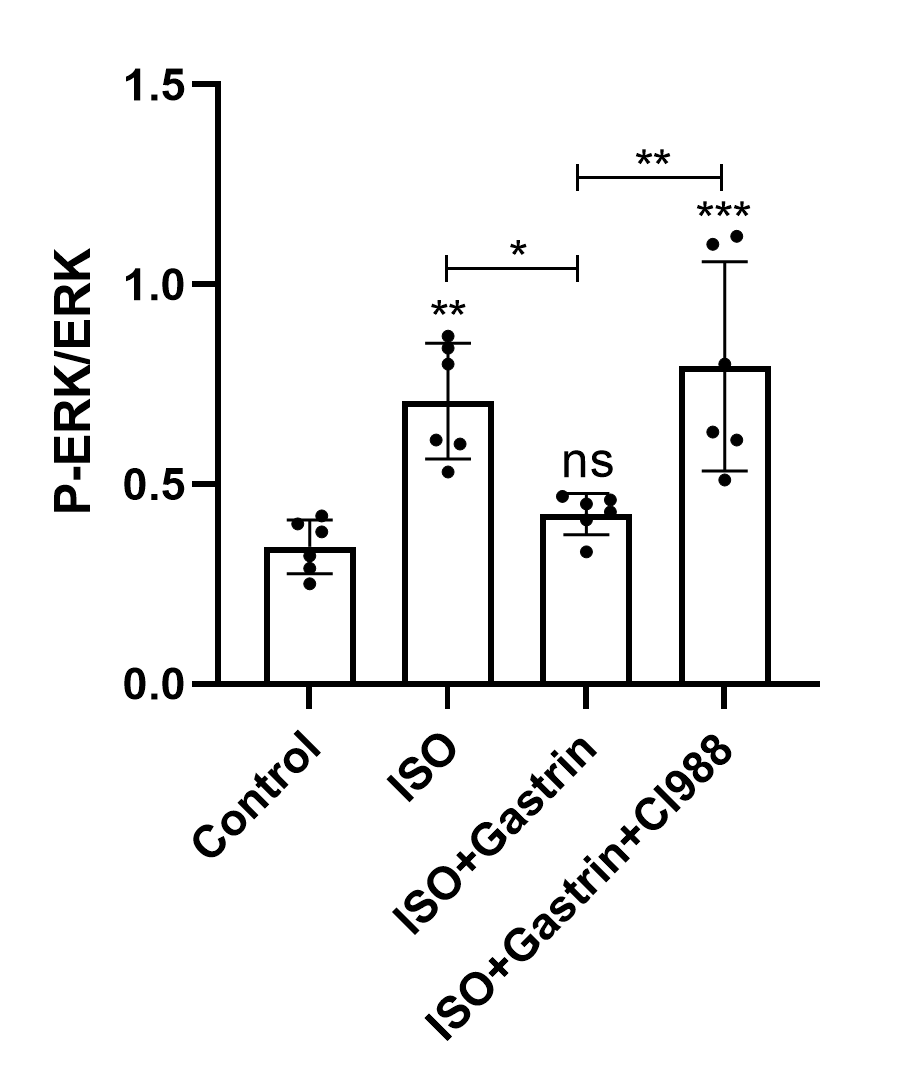


**The level of p-ERK/ERK**

**Fig 6.(B)Cardiomyocyte surface areas in H9c2 myocardial cells**

Cell crawling cultures were maintained through three serial subpassages. During each subpassage, cells were assigned to the following experimental groups: Control, Gastrin, ISO, ISO+gastrin and ISO+gastrin+CI988 group, with corresponding treatments administered as per protocol. Subsequently, cytoskeletal fluorescence staining was performed, and images were captured at 400×magnification. Ten representative fields of view were selected per sample. The average cell size in each field was quantified using ImageJ software, and the mean value across the ten fields was calculated to determine the overall average cardiomyocyte surface areas per sample.

Table 67 The cardiomyocyte surface areas of each group

| (um^2^) | Control | Gastrin | ISO | ISO+Gastrin | ISO+Gastrin+CI988 |
| --- | --- | --- | --- | --- | --- |
| 1 | 854.73 | 948.59 | 2031.10 | 1235.10 | 1732.97 |
| 2 | 830.58 | 994.30 | 2128.71 | 1250.04 | 1847.14 |
| 3 | 1110.50 | 799.94 | 1939.59 | 1196.60 | 1786.50 |

Table 68 The comparison of H9c2 cells surface areas across each group

|  | groups | surface areas across(um^2^)  Mean±SD | F-value | P-value |
| --- | --- | --- | --- | --- |
| ANOVA |  |  | 81.942 | ＜0.001 |
| Multiple comparisons | Control | 931.94±155.11 |  | 0.999 |
|  | Gastrin | 914.28±101.62 |  |  |
|  | Control | 931.94±155.11 |  | ＜0.001 |
|  | ISO | 2033.13±94.58 |  |  |
|  | Control | 931.94±155.11 |  | 0.026 |
|  | ISO+gatrin | 1227.25±27.57 |  |  |
|  | Control | 931.94±155.11 |  | ＜0.001 |
|  | ISO+gatrin+CI988 | 1788.87±57.12 |  |  |
|  | ISO | 2033.13±94.58 |  | ＜0.001 |
|  | ISO+gastrin | 1227.25±27.57 |  |  |
|  | ISO+gastrin | 1227.25±27.57 |  | ＜0.001 |
|  | ISO+gastrin+CI988 | 1788.87±57.12 |  |  |


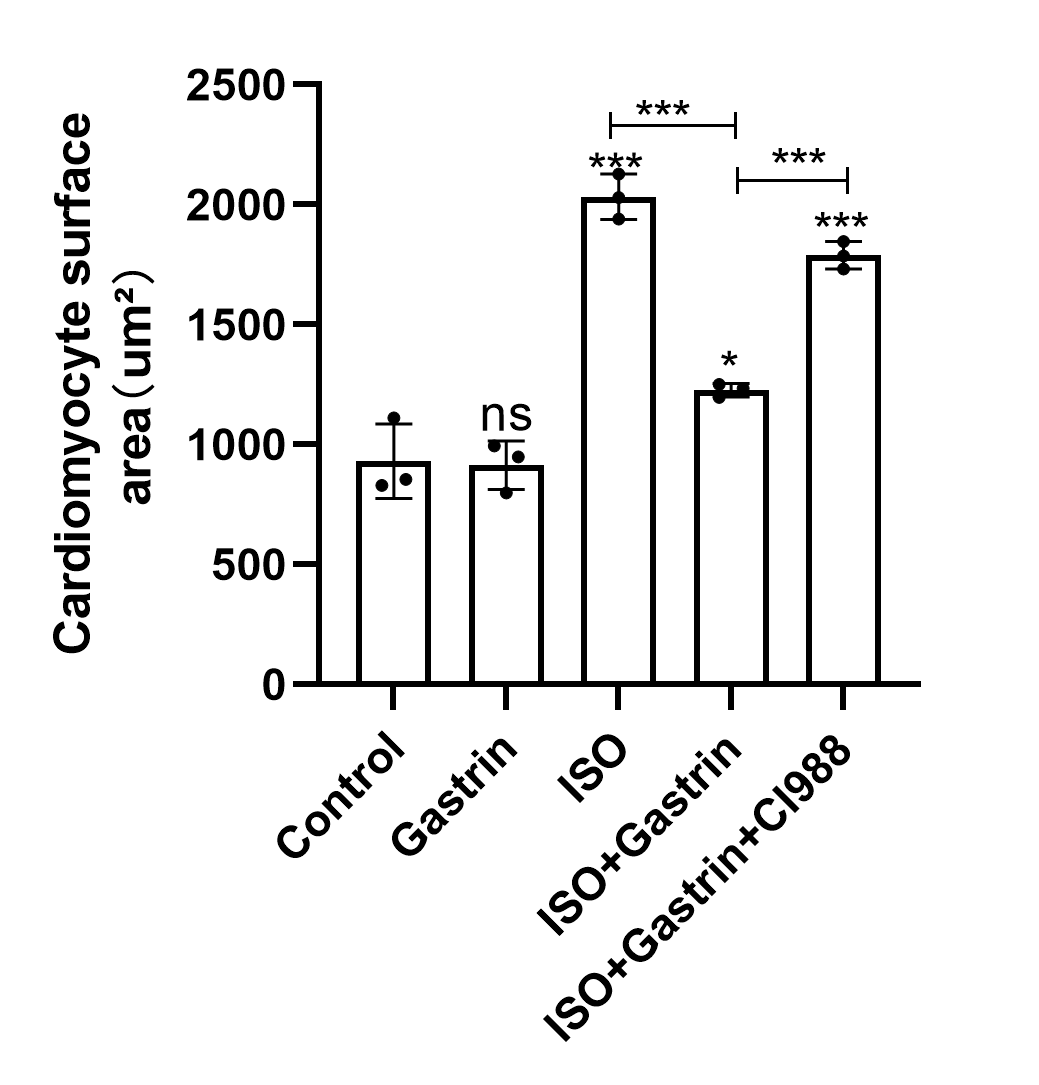


**The** **cardiomyocyte surface areas of five group**

**Fig 6.(C)The expression of ANP of H9c2 myocardial cells in control, gastrin, ISO, and ISO+gastrin groups**

Table 69 Quantitative analysis of ANP expression in H9c2 cells using the 2^-ΔΔCT^ method

| ANP/18S  (2^-ΔΔCT^) | Control | Gastrin | ISO | ISO+Gastrin |
| --- | --- | --- | --- | --- |
| Experiment1 | 1 | 1.02 | 4.58 | 1.74 |
| Experiment2 | 1 | 1.46 | 3.15 | 1.27 |
| Experiment3 | 1 | 1.52 | 2.7 | 1.57 |

Table70 The comparison of ANP across each group

|  | groups | ANP(2^-ΔΔCT^)  Mean±SD | F-value | P-value |
| --- | --- | --- | --- | --- |
| ANOVA |  |  | 13.731 | 0.002 |
| Multiple comparisons | Control | 1 |  | 0.859 |
|  | Gastrin | 1.33±0.28 |  |  |
|  | Control | 1 |  | 0.002 |
|  | ISO | 3.48±0.98 |  |  |
|  | Control | 1 |  | 0.062 |
|  | ISO+gatrin | 1.53±0.24 |  |  |
|  | ISO | 3.48±0.98 |  | 0.008 |
|  | ISO+gastrin | 1.53±0.24 |  |  |


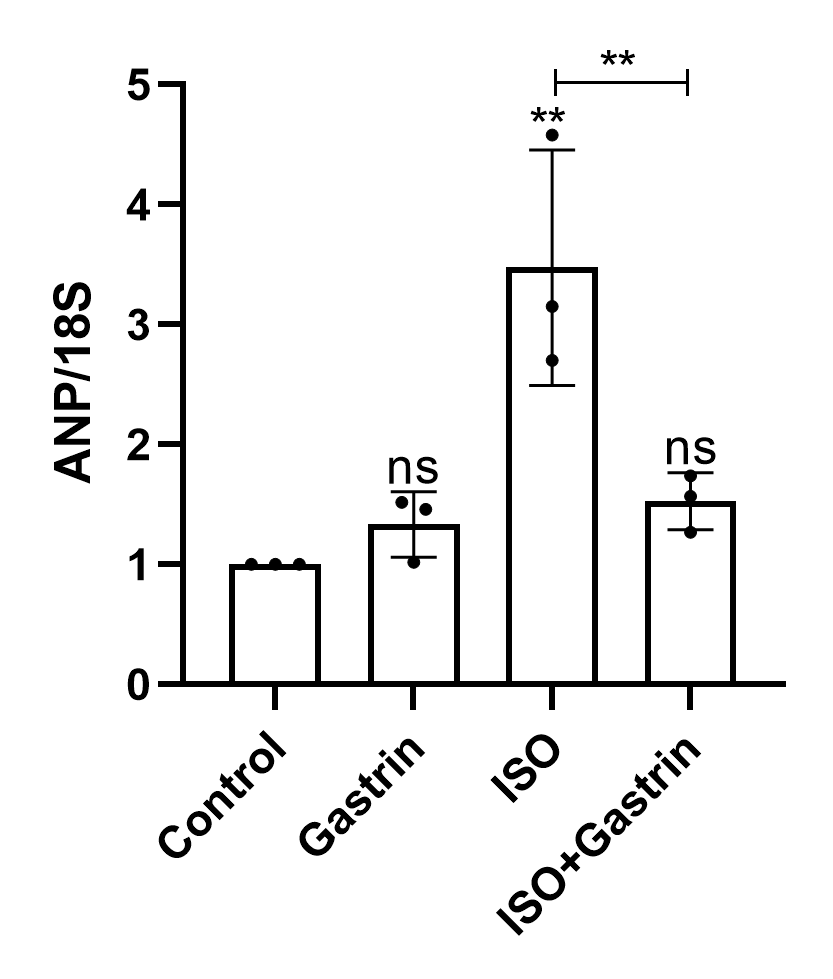


**The ANP expression of H9c2 cells**

**Fig 6.(D)The expression of BNP of H9c2 myocardial cells in control, gastrin, ISO, and ISO+gastrin groups**

Table 71 Quantitative analysis of BNP expression in H9c2 cells using the 2^-ΔΔCT^ method

| BNP/18S  (2^-ΔΔCT^) | Control | Gastrin | ISO | ISO+Gastrin |
| --- | --- | --- | --- | --- |
| Experiment1 | 1 | 1.24 | 4.93 | 1.03 |
| Experiment2 | 1 | 1.31 | 5.11 | 2.01 |
| Experiment3 | 1 | 0.98 | 2.72 | 1.26 |

Table72 The comparison of BNP across each group

|  | groups | BNP(2^-ΔΔCT^)  Mean±SD | F-value | P-value |
| --- | --- | --- | --- | --- |
| ANOVA |  |  | 13.800 | 0.002 |
| Multiple comparisons | Control | 1 |  | 0.990 |
|  | Gastrin | 1.17±0.17 |  |  |
|  | Control | 1 |  | 0.002 |
|  | ISO | 4.25±1.33 |  |  |
|  | Control | 1 |  | 0.877 |
|  | ISO+gatrin | 1.43±0.51 |  |  |
|  | ISO | 4.25±1.33 |  | 0.006 |
|  | ISO+gastrin | 1.43±0.51 |  |  |


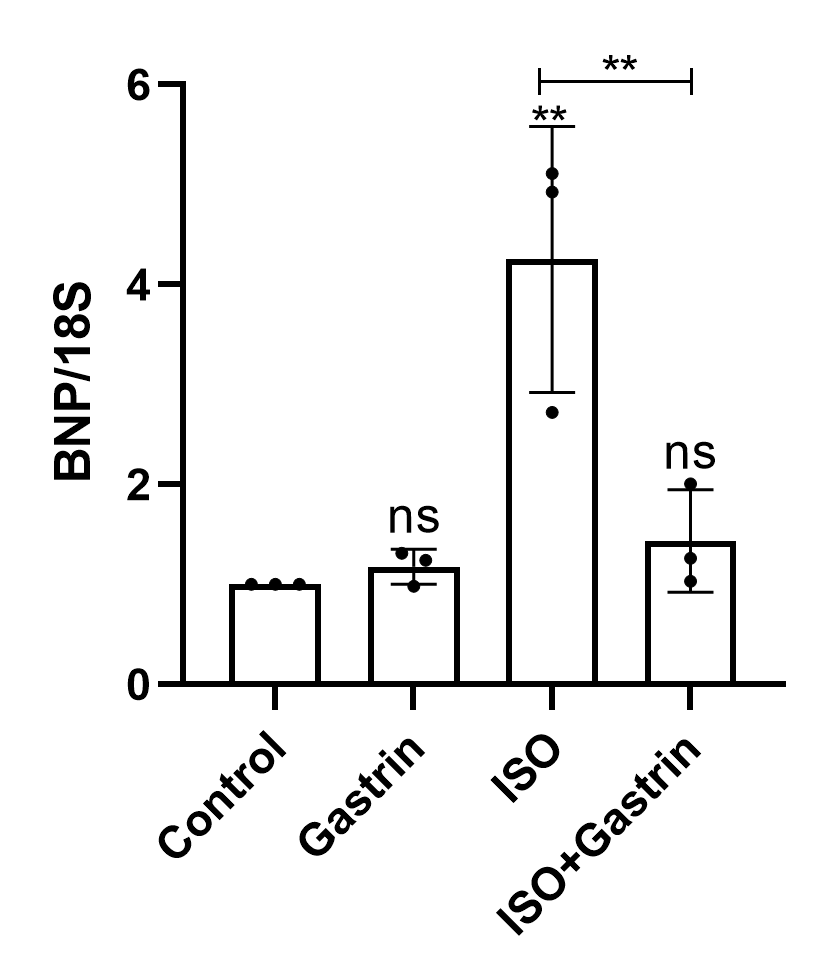


**The BNP expression of H9c2 cells**

**Fig 6.(E)The expression of ANP of H9c2 myocardial cells in control, gastrin, ISO+gastrin, and ISO+gastrin+ CI-988 groups**

Table 73 Quantitative analysis of ANP expression in H9c2 cells using the 2^-ΔΔCT^ method

| ANP/18S  (2^-ΔΔCT^) | Control | ISO | ISO+Gastrin | ISO+Gastrin+CI988 |
| --- | --- | --- | --- | --- |
| Experiment1 | 1 | 3.54 | 1.79 | 2.99 |
| Experiment2 | 1 | 3.17 | 1.66 | 2.71 |
| Experiment3 | 1 | 3.56 | 1.21 | 2.24 |

Table74 The comparison of ANP across each group

|  | groups | ANP(2^-ΔΔCT^)  Mean±SD | F-value | P-value |
| --- | --- | --- | --- | --- |
| ANOVA |  |  | 50.126 | ＜0.001 |
| Multiple comparisons | Control | 1 |  | ＜0.001 |
|  | ISO | 3.43±0.22 |  |  |
|  | Control | 1 |  | 0.127 |
|  | ISO+gatrin | 1.55±0.30 |  |  |
|  | Control | 1 |  | ＜0.001 |
|  | ISO+gastrin+CI988 | 2.65±0.38 |  |  |
|  | ISO | 3.43±0.22 |  | ＜0.001 |
|  | ISO+gatrin | 1.55±0.30 |  |  |
|  | ISO+gatrin | 1.55±0.30 |  | 0.004 |
|  | ISO+gastrin+CI988 | 2.65±0.38 |  |  |


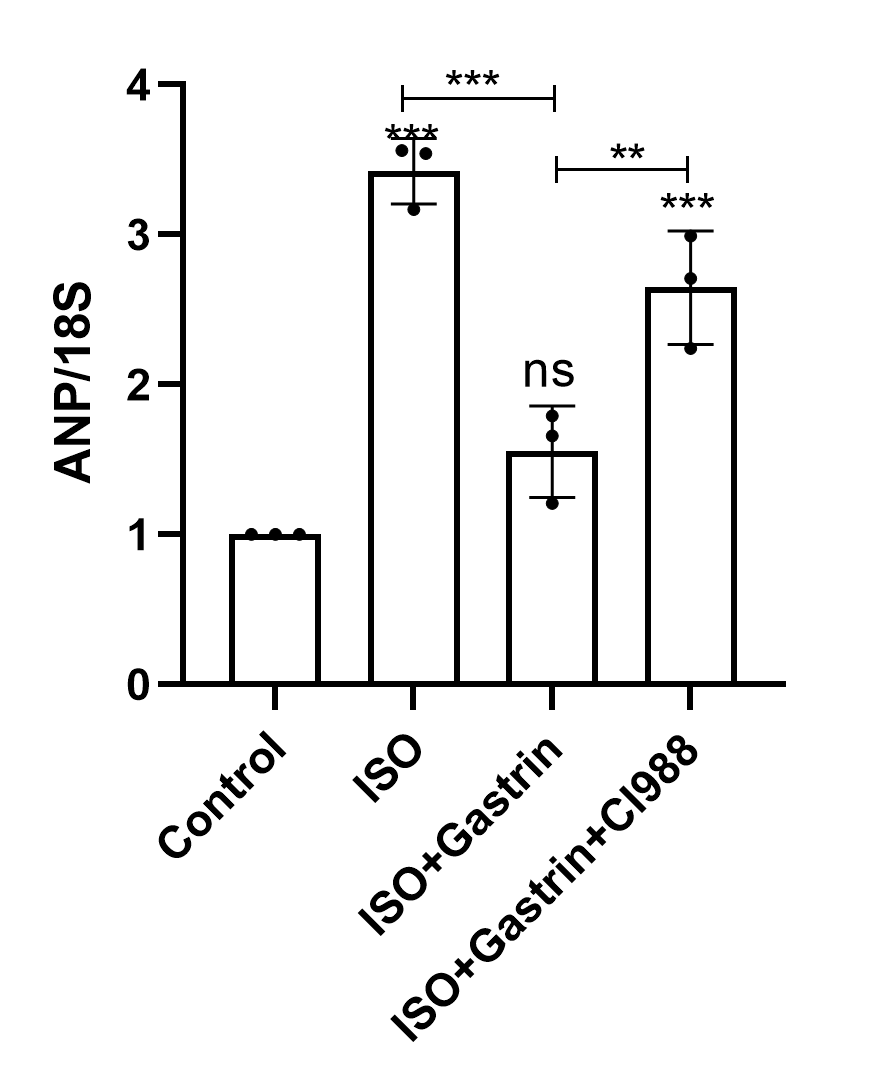


**The ANP expression of H9c2 cells**

**Fig 6.(F)The expression of BNP of H9c2 myocardial cells in control, gastrin, ISO+gastrin, and ISO+gastrin+ CI-988 groups**

Table 75 Quantitative analysis of BNP expression in H9c2 cells using the 2^-ΔΔCT^ method

| BNP/18S  (2^-ΔΔCT^) | Control | ISO | ISO+Gastrin | ISO+Gastrin+CI988 |
| --- | --- | --- | --- | --- |
| Experiment1 | 1 | 3.15 | 1.08 | 2.95 |
| Experiment2 | 1 | 5.2 | 2.17 | 4.21 |
| Experiment3 | 1 | 4.55 | 1.27 | 3.52 |

Table76 The comparison of BNP across each group

|  | groups | BNP(2^-ΔΔCT^)  Mean±SD | F-value | P-value |
| --- | --- | --- | --- | --- |
| ANOVA |  |  | 16.600 | ＜0.001 |
| Multiple comparisons | Control | 1 |  | 0.001 |
|  | ISO | 4.30±1.04 |  |  |
|  | Control | 1 |  | 0.797 |
|  | ISO+gatrin | 1.51±0.58 |  |  |
|  | Control | 1 |  | 0.007 |
|  | ISO+gastrin+CI988 | 3.56±0.63 |  |  |
|  | ISO | 4.30±1.04 |  | 0.004 |
|  | ISO+gatrin | 1.51±0.58 |  |  |
|  | ISO+gatrin | 1.51±0.58 |  | 0.024 |
|  | ISO+gastrin+CI988 | 3.56±0.63 |  |  |


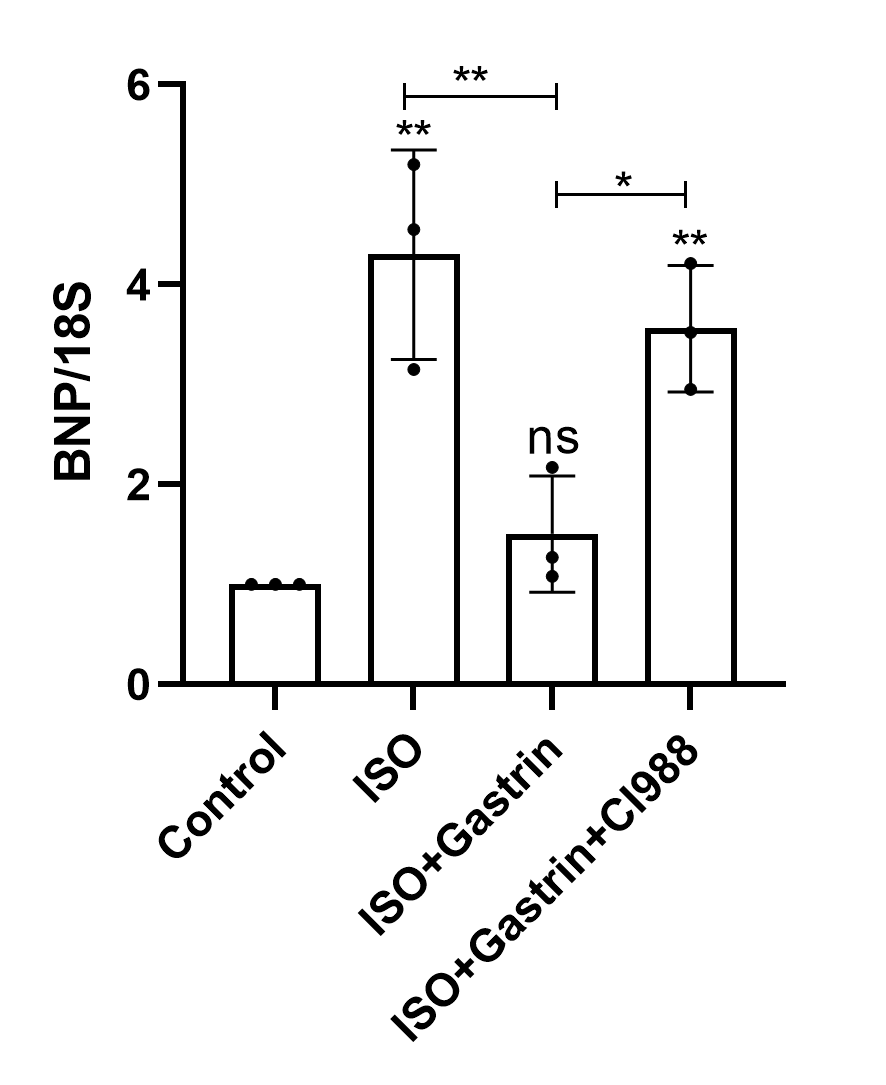


**The BNP expression of H9c2 cells**

**Fig 7.(B)The level of p-JAK2/JAK2 of H9c2 myocardial cells in the Control,Gastrin, ISO, and ISO+Gastrin groups**

Table 77 The grayscale intensity of P-JAK2/JAK2

| groups | The grayscale intensity of JAK2 | The grayscale intensity of P-JAK2 | P-JAK2/JAK2 |
| --- | --- | --- | --- |
| Control | 1,081,232.00 | 905,273.00 | 0.837260643 |
|  | 1,040,584.00 | 983,156.00 | 0.94481176 |
|  | 928,896.00 | 1,561,825.00 | 1.681377678 |
| Gastrin | 677,112.00 | 1,418,791.00 | 2.095356455 |
|  | 716,888.00 | 1,889,968.00 | 2.636350448 |
|  | 616,633.00 | 1,880,797.00 | 3.0501076 |
| ISO | 813,314.00 | 4,783,389.00 | 5.881355786 |
|  | 783,077.00 | 4,563,559.00 | 5.827727031 |
|  | 864,421.00 | 4,285,529.00 | 4.957687284 |
| ISO+Gastrin | 729,790.00 | 3,279,014.00 | 4.493092533 |
|  | 792,840.00 | 1,973,027.00 | 2.488556329 |
|  | 1,027,852.00 | 754,005.00 | 0.733573511 |

Table 78 The comparison of P-JAK2/JAK2 across each group

|  | groups | P-JAK2/JAK2  Mean±SD | F-value | P-value |
| --- | --- | --- | --- | --- |
| ANOVA |  |  | 9.681 | 0.005 |
| Multiple comparisons | Control | 1.15±0.46 |  | 0.378 |
|  | Gastrin | 2.59±0.48 |  |  |
|  | Control | 1.15±0.46 |  | 0.004 |
|  | ISO | 5.56±0.52 |  |  |
|  | Control | 1.15±0.46 |  | 0.390 |
|  | ISO+gatrin | 2.57±1.88 |  |  |
|  | ISO | 5.56±0.52 |  | 0.031 |
|  | ISO+gastrin | 2.57±1.88 |  |  |


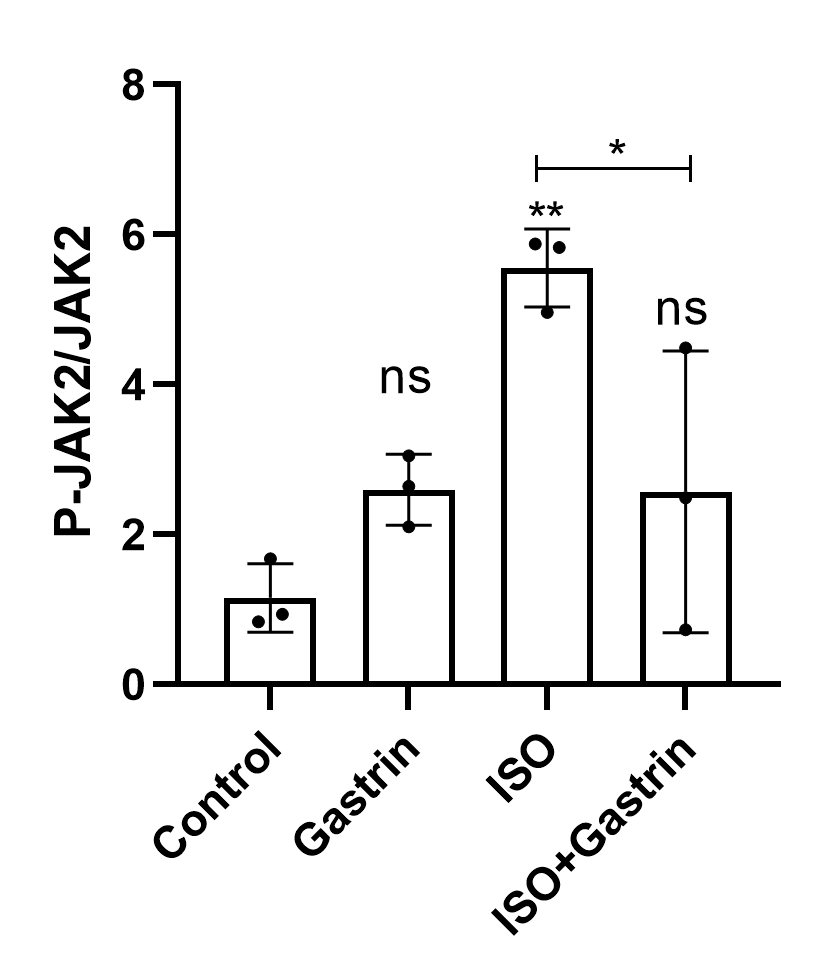


**The level of p-JAK2/JAK2 of H9c2 cells**

**Fig 7.(C)The level of p-STAT3/ STAT3 of H9c2 myocardial cells in the Control,Gastrin, ISO, and ISO+Gastrin groups**

Table 79 The grayscale intensity of P-STAT3/STAT3

| groups | The grayscale intensity of STAT3 | The grayscale intensity of P-STAT3 | P-STAT3/STAT3 |
| --- | --- | --- | --- |
| Control | 3,948,831.00 | 1,561,376.00 | 0.395402082 |
|  | 6,821,366.00 | 1,728,752.00 | 0.253431937 |
|  | 6,122,683.00 | 1,688,634.00 | 0.275799678 |
| Gastrin | 5,009,080.00 | 1,907,924.00 | 0.380893098 |
|  | 5,352,864.00 | 2,641,915.00 | 0.493551676 |
|  | 5,724,657.00 | 2,860,320.00 | 0.499649149 |
| ISO | 5,146,154.00 | 3,387,970.00 | 0.658349906 |
|  | 6,019,342.00 | 3,255,064.00 | 0.540767413 |
|  | 5,633,738.00 | 2,978,416.00 | 0.528674922 |
| ISO+Gastrin | 5,873,106.00 | 2,112,612.00 | 0.359709496 |
|  | 5,484,422.00 | 402,528.00 | 0.07339479 |
|  | 4,081,817.00 | 1,357,420.00 | 0.33255288 |

Table 80 The comparison of P-STAT3/STAT3 across each group

|  | groups | P-STAT3/STAT3Mean±SD | F-value | P-value |
| --- | --- | --- | --- | --- |
| ANOVA |  |  | 6.306 | 0.017 |
| Multiple comparisons | Control | 1.35±0.20 |  | 0.329 |
|  | Gastrin | 1.42±0.29 |  |  |
|  | Control | 1.35±0.20 |  | 0.046 |
|  | ISO | 2.35±0.41 |  |  |
|  | Control | 1.35±0.20 |  | 0.914 |
|  | ISO+gatrin | 1.77±0.17 |  |  |
|  | ISO | 2.35±0.41 |  | 0.019 |
|  | ISO+gastrin | 1.77±0.17 |  |  |


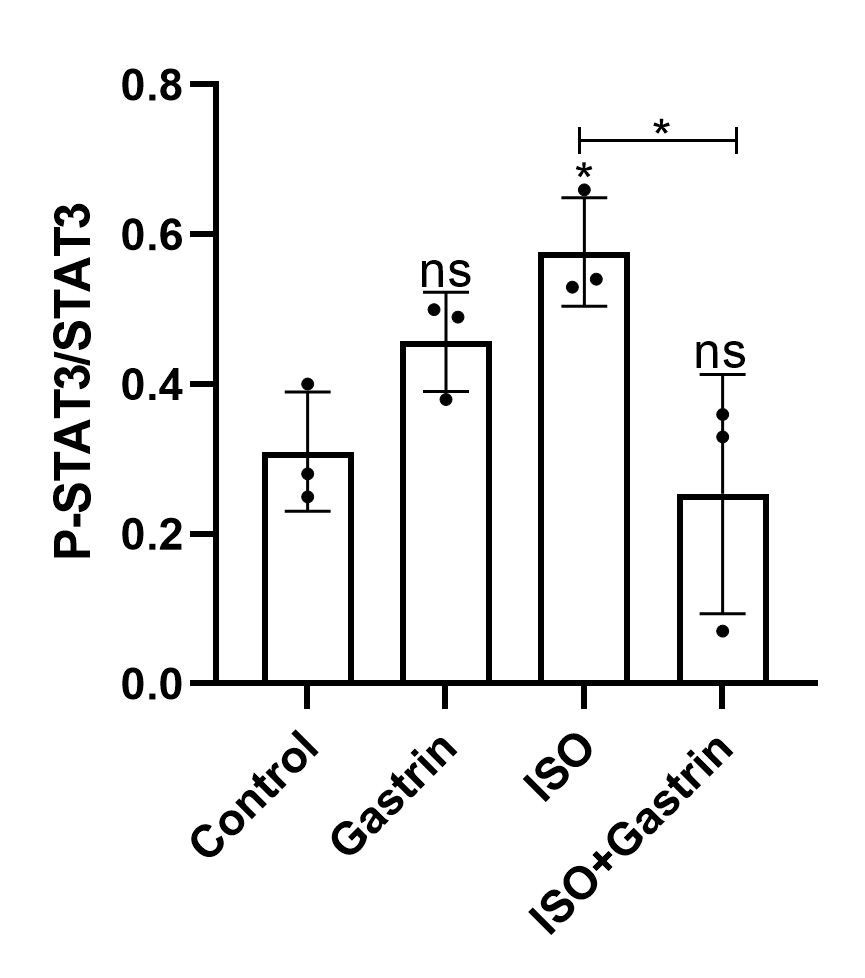


**The level of p-STAT3/ STAT3 of H9c2 cells**

**Fig 7.(D)The level of p-ERK/ERK of H9c2 myocardial cells in the Control,Gastrin, ISO, and ISO+Gastrin groups**

Table 81 The grayscale intensity of P-ERK/ERK

| groups | The grayscale intensity of ERK | The grayscale intensity of P-ERK | P-ERK/ERK |
| --- | --- | --- | --- |
| Control | 17,587,630.00 | 9,993,455.00 | 0.568209304 |
|  | 18,333,477.00 | 11,076,787.00 | 0.604183647 |
|  | 17,624,272.00 | 10,260,914.00 | 0.582203566 |
| Gastrin | 16,312,719.00 | 10,315,843.00 | 0.632380353 |
|  | 17,842,891.00 | 12,013,728.00 | 0.673306136 |
|  | 15,132,538.00 | 11,945,080.00 | 0.789363952 |
| ISO | 14,706,289.00 | 15,290,849.00 | 1.039748981 |
|  | 15,830,779.00 | 15,177,299.00 | 0.95872092 |
|  | 17,197,556.00 | 14,047,735.00 | 0.816844847 |
| ISO+Gastrin | 17,200,282.00 | 13,463,819.00 | 0.782767341 |
|  | 18,583,963.00 | 13,805,724.00 | 0.742883743 |
|  | 19,588,970.00 | 12,403,793.00 | 0.63320292 |

Table 82 The comparison of P-ERK/ERK across each group

|  | groups | 1. ERK/ERK 2. Mean±SD | F-value | P-value |
| --- | --- | --- | --- | --- |
| ANOVA |  |  | 10.20 | 0.004 |
| Multiple comparisons | Control | 0.58±0.02 |  | 0.368 |
|  | Gastrin | 0.70±0.08 |  |  |
|  | Control | 0.58±0.02 |  | 0.003 |
|  | ISO | 0.94±0.11 |  |  |
|  | Control | 0.58±0.02 |  | 0.244 |
|  | ISO+gatrin | 0.72±0.08 |  |  |
|  | ISO | 0.94±0.11 |  | 0.041 |
|  | ISO+gastrin | 0.72±0.08 |  |  |


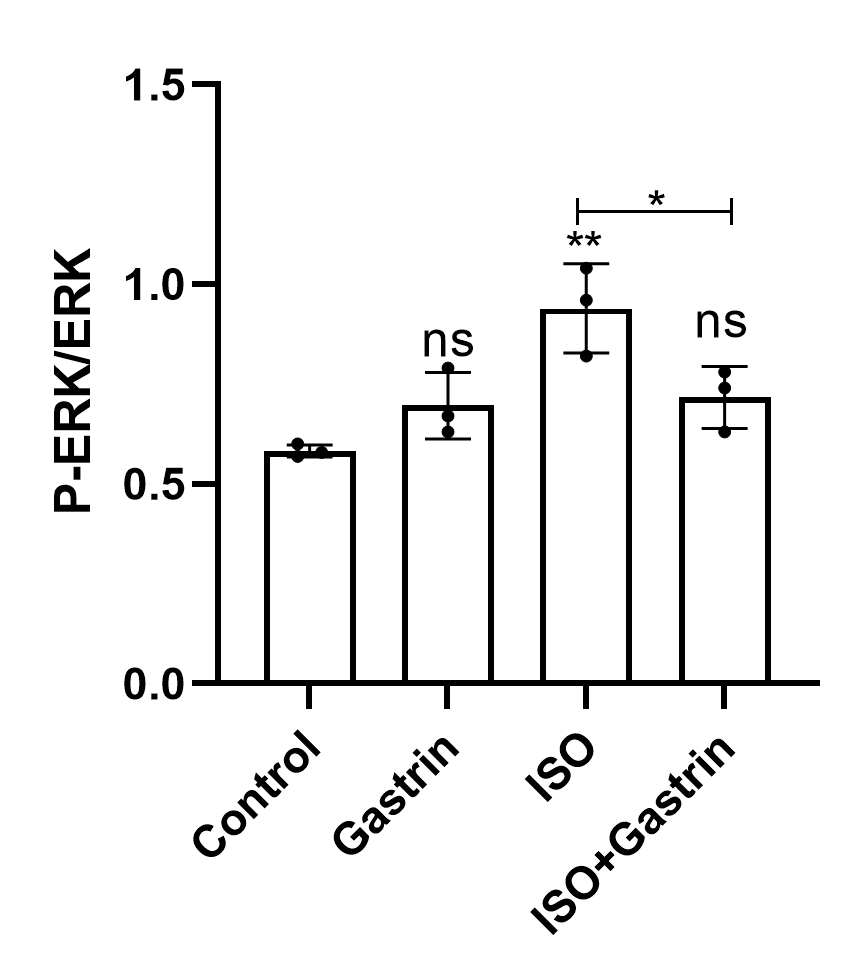


**The level of p-ERK/ERK of H9c2 cells**

**Fig 7.(F)The level of p-JAK2/ JAK2 of H9c2 myocardial cells in the Control, ISO, ISO+ Gastrin, and ISO+Gastrin+CI-988 groups**

Table 83 The grayscale intensity of P-JAK2/JAK2

| groups | The grayscale intensity of JAK2 | The grayscale intensity of P-JAK2 | P-JAK2/JAK2 |
| --- | --- | --- | --- |
| Control | 4,439,938.00 | 2,689,728.00 | 0.605803054 |
|  | 4,586,168.00 | 2,963,413.00 | 0.646163202 |
|  | 5,872,400.00 | 3,965,792.00 | 0.675327294 |
| ISO | 3,521,784.00 | 4,861,336.00 | 1.380361771 |
|  | 4,142,386.00 | 4,492,248.00 | 1.084459053 |
|  | 6,501,387.00 | 6,425,352.00 | 0.988304803 |
| ISO+Gastrin | 6,830,468.00 | 3,308,822.00 | 0.48442098 |
|  | 6,257,851.00 | 2,910,746.00 | 0.465135076 |
|  | 5,436,592.00 | 2,496,573.00 | 0.459216546 |
| ISO+Gastrin+CI988 | 4,985,458.00 | 3,259,993.00 | 0.653900404 |
|  | 4,708,902.00 | 4,273,380.00 | 0.907510923 |
|  | 4,702,916.00 | 4,228,479.00 | 0.899118547 |

Table 84 The comparison of P-JAK2/JAK2 across each group

|  | groups | P-JAK2/JAK2  Mean±SD | F-value | P-value |
| --- | --- | --- | --- | --- |
| ANOVA |  |  | 15.915 | ＜0.001 |
| Multiple comparisons | Control | 0.64±0.03 |  | 0.005 |
|  | ISO | 1.15±0.20 |  |  |
|  | Control | 0.64±0.03 |  | 0.395 |
|  | ISO+gatrin | 0.47±0.01 |  |  |
|  | Control | 0.64±0.03 |  | 0.373 |
|  | ISO+gastrin+CI988 | 0.82±0.14 |  |  |
|  | ISO | 1.15±0.20 |  | ＜0.001 |
|  | ISO+gastrin | 0.47±0.01 |  |  |
|  | ISO+gastrin | 0.47±0.01 |  | 0.038 |
|  | ISO+gastrin+CI988 | 0.82±0.14 |  |  |


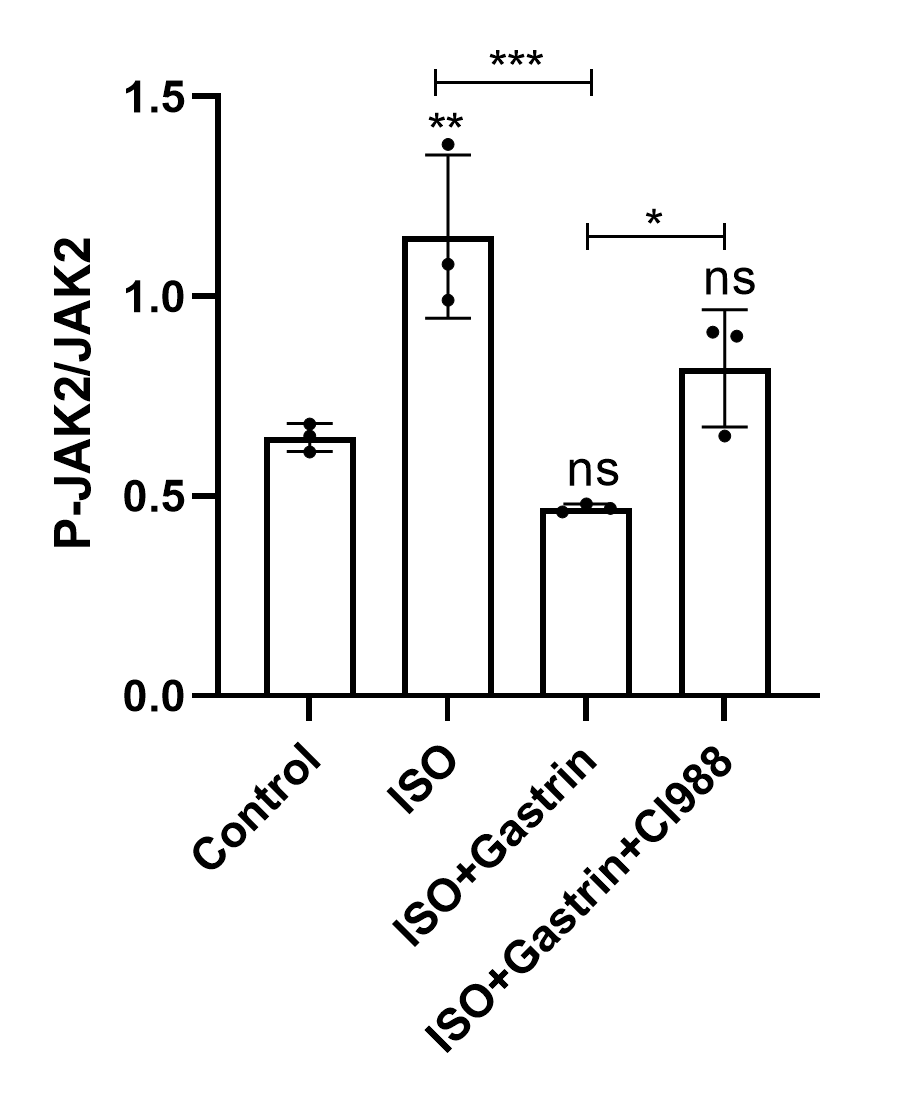


**The level of p-JAK2/ JAK2 of H9c2 cells**

**Fig 7.(G)The level of p-STAT3/ STAT3 of H9c2 myocardial cells in the Control, ISO, ISO+ Gastrin, and ISO+Gastrin+CI-988 groups**

Table 85 The grayscale intensity of P-STAT3/STAT3

| groups | The grayscale intensity of STAT3 | The grayscale intensity of P-STAT3 | P-STAT3/STAT3 |
| --- | --- | --- | --- |
| Control | 1,943,919.00 | 1,142,625.00 | 0.587794553 |
|  | 3,054,682.00 | 1,007,484.00 | 0.329816328 |
|  | 2,380,100.00 | 1,345,349.00 | 0.565248939 |
| ISO | 1,341,452.00 | 1,186,073.00 | 0.884171033 |
|  | 1,658,444.00 | 1,365,237.00 | 0.823203557 |
|  | 1,932,760.00 | 1,375,742.00 | 0.711801776 |
| ISO+Gastrin | 3,439,782.00 | 1,211,426.00 | 0.352181039 |
|  | 2,058,952.00 | 1,094,999.00 | 0.531823471 |
|  | 2,601,166.00 | 734,107.00 | 0.28222228 |
| ISO+Gastrin+CI988 | 1,193,156.00 | 932,750.00 | 0.781750249 |
|  | 1,150,051.00 | 957,263.00 | 0.832365695 |
|  | 1,692,572.00 | 1,180,560.00 | 0.6974947 |

Table 86 The comparison of P-STAT3/STAT3 across each group

|  | groups | P-STAT3/STAT3  Mean±SD | F-value | P-value |
| --- | --- | --- | --- | --- |
| ANOVA |  |  | 10.276 | 0.004 |
| Multiple comparisons | Control | 0.49±0.14 |  | 0.036 |
|  | ISO | 0.81±0.09 |  |  |
|  | Control | 0.49±0.14 |  | 0.663 |
|  | ISO+gatrin | 0.39±0.13 |  |  |
|  | Control | 0.49±0.14 |  | 0.062 |
|  | ISO+gastrin+CI988 | 0.77±0.07 |  |  |
|  | ISO | 0.81±0.09 |  | 0.008 |
|  | ISO+gastrin | 0.39±0.13 |  |  |
|  | ISO+gastrin | 0.39±0.13 |  | 0.013 |
|  | ISO+gastrin+CI988 | 0.77±0.07 |  |  |


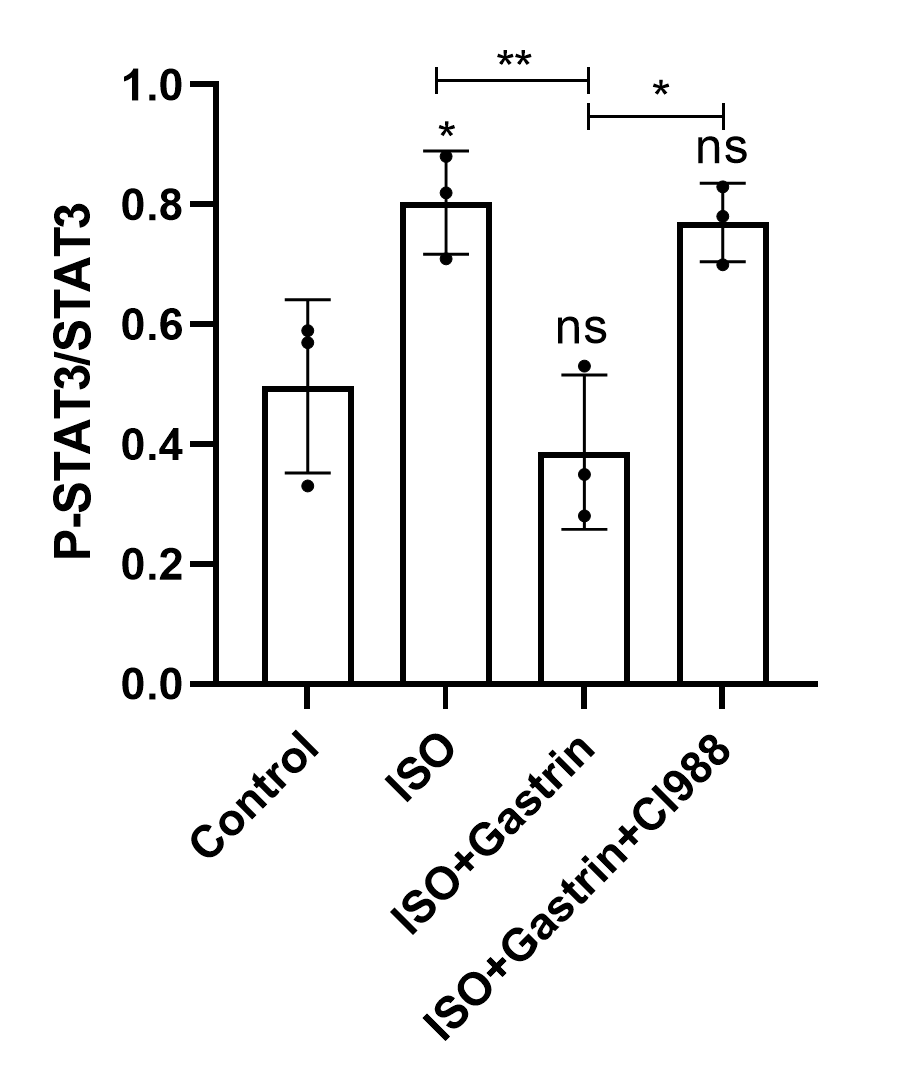


**The level of p-STAT3/ STAT3 of H9c2 cells**

**Fig 7.(H)The level of p-ERK/ERK of H9c2 myocardial cells in the Control, ISO, ISO+ Gastrin, and ISO+Gastrin+CI-988 groups**

Table 87 The grayscale intensity of P-ERK/ERK

| groups | The grayscale intensity of ERK | The grayscale intensity of P-ERK | P-ERK/ERK |
| --- | --- | --- | --- |
| Control | 22,044,402.00 | 4,857,327.00 | 0.220342879 |
|  | 22,764,787.00 | 4,437,457.00 | 0.194926357 |
|  | 22,920,087.00 | 6,040,525.00 | 0.263547211 |
| ISO | 22,371,580.00 | 16,946,896.00 | 0.757518959 |
|  | 20,984,135.00 | 16,824,643.00 | 0.801779201 |
|  | 20,758,966.00 | 16,896,805.00 | 0.81395215 |
| ISO+Gastrin | 21,583,085.00 | 3,232,433.00 | 0.149766959 |
|  | 22,085,083.00 | 3,261,788.00 | 0.147691906 |
|  | 20,030,539.00 | 4,008,319.00 | 0.200110391 |
| ISO+Gastrin+CI988 | 21,101,395.00 | 16,040,377.00 | 0.760157184 |
|  | 21,633,338.00 | 17,174,535.00 | 0.793892048 |
|  | 22,285,367.00 | 18,072,845.00 | 0.810973631 |

Table 88 The comparison of P-ERK/ERK across each group

|  | groups | P-ERK/ERK  Mean±SD | F-value | P-value |
| --- | --- | --- | --- | --- |
| ANOVA |  |  | 389.784 | ＜0.001 |
| Multiple comparisons | Control | 0.23±0.03 |  | ＜0.001 |
|  | ISO | 0.79±0.03 |  |  |
|  | Control | 0.23±0.03 |  | 0.143 |
|  | ISO+gatrin | 0.17±0.03 |  |  |
|  | Control | 0.23±0.03 |  | ＜0.001 |
|  | ISO+gastrin+CI988 | 0.78±0.03 |  |  |
|  | ISO | 0.79±0.03 |  | ＜0.001 |
|  | ISO+gastrin | 0.17±0.03 |  |  |
|  | ISO+gastrin | 0.17±0.03 |  | ＜0.001 |
|  | ISO+gastrin+CI988 | 0.78±0.03 |  |  |


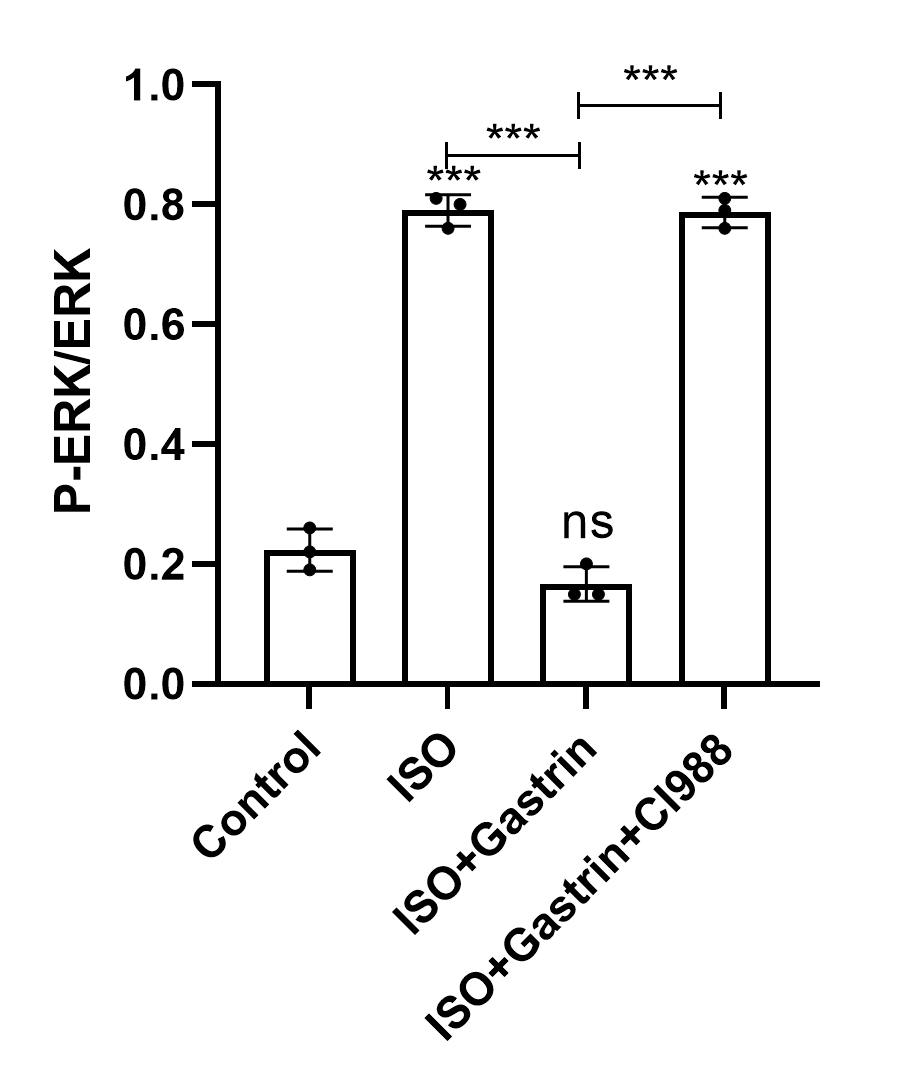


**The level of p-ERK/ERK of H9c2 cells**

**Fig 7.(I)The expression of ANP after the addition of ERK agonist mSIRK in H9c2 myocardial cells**

Table 89Quantitative analysis of ANP expression in H9c2 cells using the 2^-ΔΔCT^ method

| ANP/18S  (2^-ΔΔCT^) | Control | ISO | ISO+Gastrin | ISO+Gastrin+mSIRK |
| --- | --- | --- | --- | --- |
| Experiment1 | 1 | 3.28 | 1.1 | 2.42 |
| Experiment2 | 1 | 2.53 | 1 | 1.91 |
| Experiment3 | 1 | 3.27 | 1.55 | 3.29 |

Table 90 The comparison of ANP across each group

|  | groups | ANP(2^-ΔΔCT^)  Mean±SD | F-value | P-value |
| --- | --- | --- | --- | --- |
| ANOVA |  |  | 15.546 | 0.001 |
| Multiple comparisons | Control | 1 |  | 0.002 |
|  | ISO | 3.03±0.43 |  |  |
|  | Control | 1 |  | 0.924 |
|  | ISO+gatrin | 1.22±0.29 |  |  |
|  | Control | 1 |  | 0.011 |
|  | ISO+gastrin+mSIRK | 2.54±0.70 |  |  |
|  | ISO | 3.03±0.43 |  | 0.004 |
|  | ISO+gatrin | 1.22±0.29 |  |  |
|  | ISO+gatrin | 1.22±0.29 |  | 0.024 |
|  | ISO+gastrin+mSIRK | 2.54±0.70 |  |  |


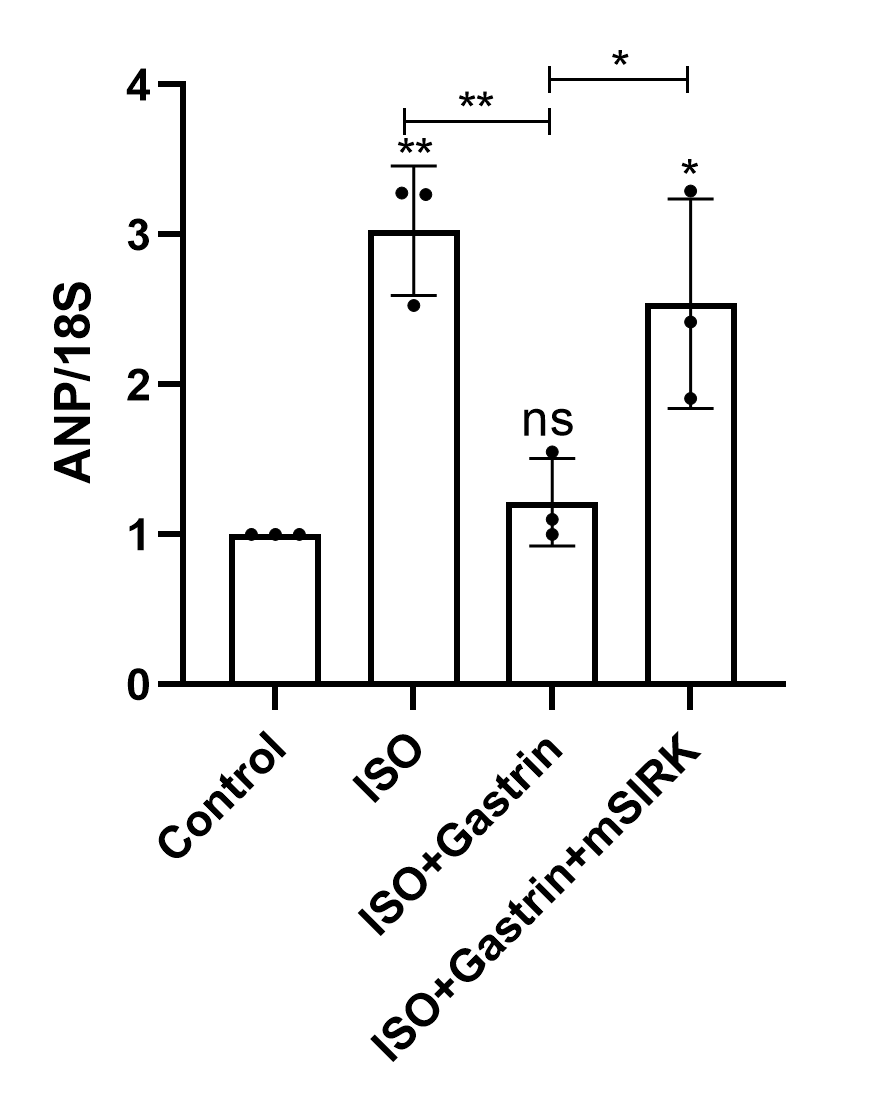


**The expression of ANP in H9c2 cells**

**Fig 7.(J)The expression of BNP after the addition of ERK agonist mSIRK in H9c2 myocardial cells**

Table 91 Quantitative analysis of BNP expression in H9c2 cells using the 2^-ΔΔCT^ method

| BNP/18S  (2^-ΔΔCT^) | Control | ISO | ISO+Gastrin | ISO+Gastrin+mSIRK |
| --- | --- | --- | --- | --- |
| Experiment1 | 1 | 3.55 | 1.84 | 3.79 |
| Experiment2 | 1 | 3.23 | 1.23 | 2.82 |
| Experiment3 | 1 | 3.48 | 1.53 | 2.96 |

Table 92 The comparison of BNP across each group

|  | groups | BNP(2^-ΔΔCT^)  Mean±SD | F-value | P-value |
| --- | --- | --- | --- | --- |
| ANOVA |  |  | 43.661 | ＜0.001 |
| Multiple comparisons | Control | 1 |  | ＜0.001 |
|  | ISO | 3.42±1.67 |  |  |
|  | Control | 1 |  | 0.237 |
|  | ISO+gatrin | 1.54±0.30 |  |  |
|  | Control | 1 |  | ＜0.001 |
|  | ISO+gastrin+mSIRK | 3.19±0.53 |  |  |
|  | ISO | 3.42±1.67 |  | ＜0.001 |
|  | ISO+gatrin | 1.54±0.30 |  |  |
|  | ISO+gatrin | 1.54±0.30 |  | ＜0.001 |
|  | ISO+gastrin+mSIRK | 3.19±0.53 |  |  |

**
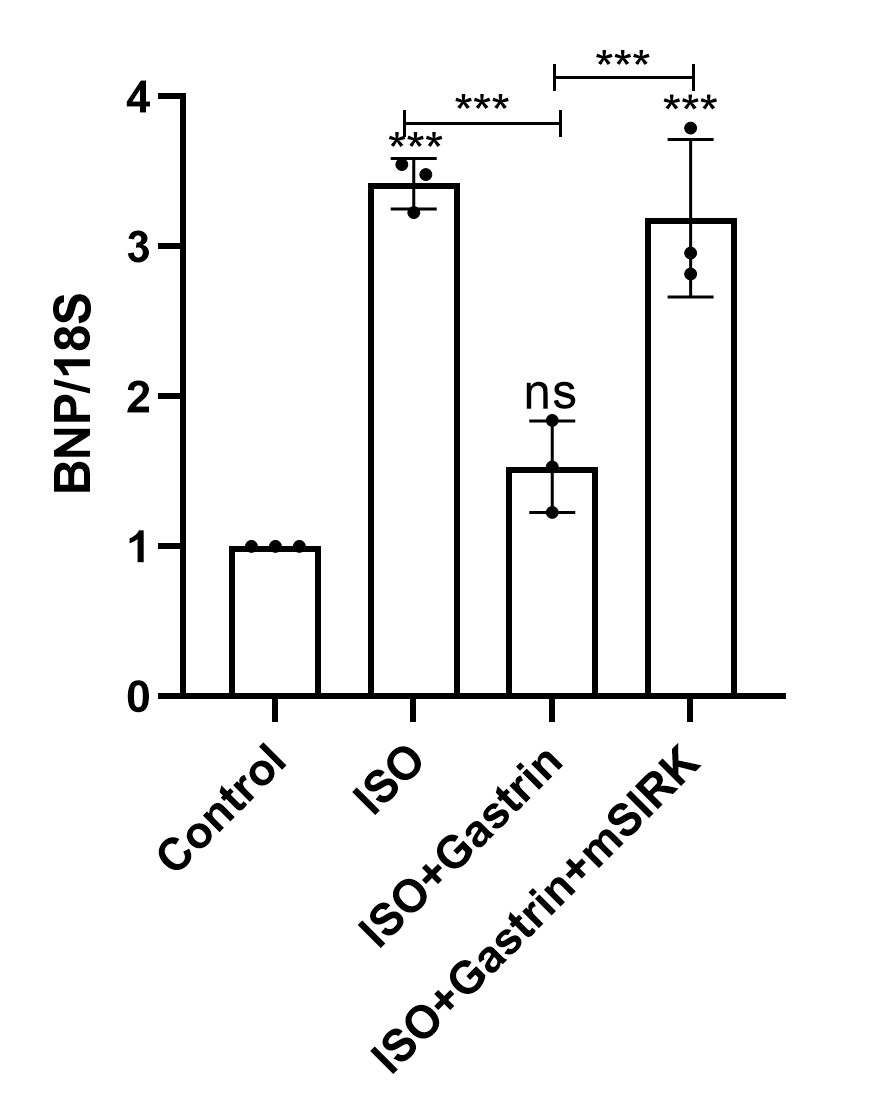
**

**The expression of ANP in H9c2 cells**

**Fig 7. (L)The level of p-ERK/ERK after the addition of ERK agonist mSIRK in H9c2 myocardial cells**

Table 93 The grayscale intensity of P-ERK/ERK

| groups | The grayscale intensity of ERK | The grayscale intensity of P-ERK | P-ERK/ERK |
| --- | --- | --- | --- |
| Control | 22,918,880.00 | 13,530,654.00 | 0.590371519 |
|  | 22,236,123.00 | 13,133,765.00 | 0.590649953 |
|  | 21,616,839.00 | 16,092,456.00 | 0.744440758 |
| ISO | 18,528,668.00 | 17,374,957.00 | 0.937733732 |
|  | 18,009,717.00 | 19,094,667.00 | 1.060242479 |
|  | 18,007,226.00 | 19,431,786.00 | 1.079110464 |
| ISO+Gastrin | 18,434,507.00 | 15,833,235.00 | 0.858891155 |
|  | 18,375,568.00 | 14,406,668.00 | 0.784012119 |
|  | 20,298,391.00 | 14,534,107.00 | 0.716022615 |
| ISO+Gastrin+CI988 | 17,782,363.00 | 19,392,147.00 | 1.090527001 |
|  | 21,288,207.00 | 20,923,210.00 | 0.982854498 |
|  | 22,106,664.00 | 21,190,689.00 | 0.958565661 |

Table 94 The comparison of P-ERK/ERK across each group

|  | groups | P-ERK/ERK  Mean±SD | F-value | P-value |
| --- | --- | --- | --- | --- |
| ANOVA |  |  | 389.784 | ＜0.001 |
| Multiple comparisons | Control | 0.64±0.09 |  | 0.001 |
|  | ISO | 1.03±0.08 |  |  |
|  | Control | 0.64±0.09 |  | 0.179 |
|  | ISO+gatrin | 0.79±0.07 |  |  |
|  | Control | 0.64±0.09 |  | 0.002 |
|  | ISO+gastrin+mSIRK | 1.01±0.07 |  |  |
|  | ISO | 1.03±0.08 |  | 0.022 |
|  | ISO+gastrin | 0.79±0.07 |  |  |
|  | ISO+gastrin | 0.79±0.07 |  | 0.030 |
|  | ISO+gastrin+mSIRK | 1.01±0.07 |  |  |

**
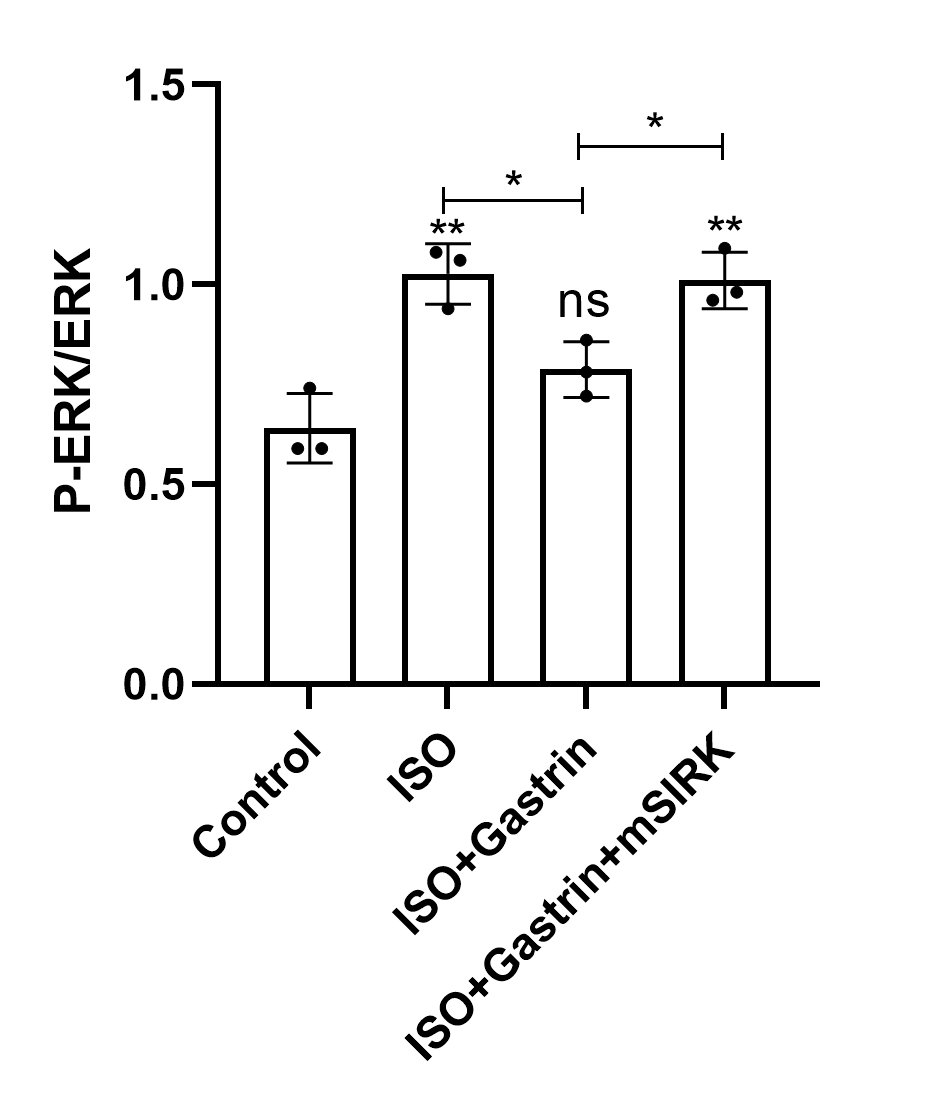
**

**The level of p-ERK/ERK**

**Fig 7.(M)The expression of ANP after the addition of STAT3 agonist colevelin in H9c2 myocardial cells**

Table95 Quantitative analysis of ANP expression in H9c2 cells using the 2^-ΔΔCT^ method

| ANP/18S  (2^-ΔΔCT^) | Control | ISO | ISO+Gastrin | ISO+Gastrin+Colevelin |
| --- | --- | --- | --- | --- |
| Experiment1 | 1 | 2.88 | 1.22 | 2.33 |
| Experiment2 | 1 | 3.39 | 1.24 | 2.69 |
| Experiment3 | 1 | 2.13 | 1.12 | 1.98 |

Table 96 The comparison of ANP across each group

|  | groups | ANP(2^-ΔΔCT^)  Mean±SD | F-value | P-value |
| --- | --- | --- | --- | --- |
| ANOVA |  |  | 16.995 | ＜0.001 |
| Multiple comparisons | Control | 1 |  | 0.001 |
|  | ISO | 2.8±0.64 |  |  |
|  | Control | 1 |  | 0.911 |
|  | ISO+gatrin | 1.2±0.06 |  |  |
|  | Control | 1 |  | 0.009 |
|  | ISO+gastrin+Colevelin | 2.33±0.36 |  |  |
|  | ISO | 2.8±0.64 |  | 0.003 |
|  | ISO+gatrin | 1.2±0.06 |  |  |
|  | ISO+gatrin | 1.2±0.06 |  | 0.022 |
|  | ISO+gastrin+Colevelin | 2.33±0.36 |  |  |


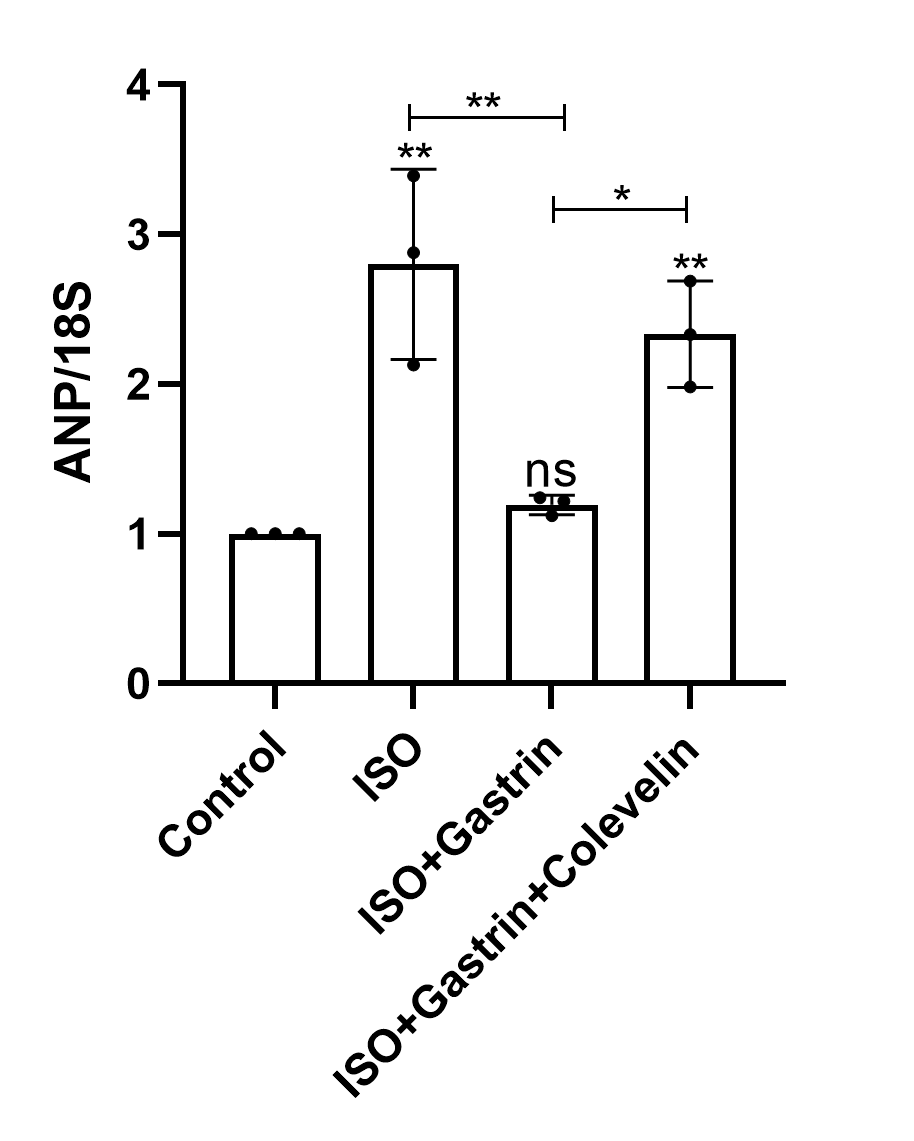


**The expression of ANP in H9c2 cells**

**Fig 7.(N)The expression of BNP after the addition of STAT3 agonist colevelin in H9c2 myocardial cells**

Table 97 Quantitative analysis of BNP expression in H9c2 cells using the 2^-ΔΔCT^ method

| BNP/18S  (2^-ΔΔCT^) | Control | ISO | ISO+Gastrin | ISO+Gastrin+Colevelin |
| --- | --- | --- | --- | --- |
| Experiment1 | 1 | 2.38 | 1.19 | 2.22 |
| Experiment2 | 1 | 3.77 | 1.48 | 2.89 |
| Experiment3 | 1 | 2.84 | 1.2 | 2.14 |

Table 98 The comparison of BNP across each group

|  | groups | BNP(2^-ΔΔCT^)  Mean±SD | F-value | P-value |
| --- | --- | --- | --- | --- |
| ANOVA |  |  | 15.354 | 0.001 |
| Multiple comparisons | Control | 1 |  | 0.002 |
|  | ISO | 3.0±0.7 |  |  |
|  | Control | 1 |  | 0.830 |
|  | ISO+gatrin | 1.29±0.16 |  |  |
|  | Control | 1 |  | 0.013 |
|  | ISO+gastrin+Colevelin | 2.42±0.41 |  |  |
|  | ISO | 4.30±1.04 |  | 0.004 |
|  | ISO+gatrin | 1.51±0.58 |  |  |
|  | ISO+gatrin | 1.51±0.58 |  | 0.042 |
|  | ISO+gastrin+Colevelin | 3.56±0.63 |  |  |


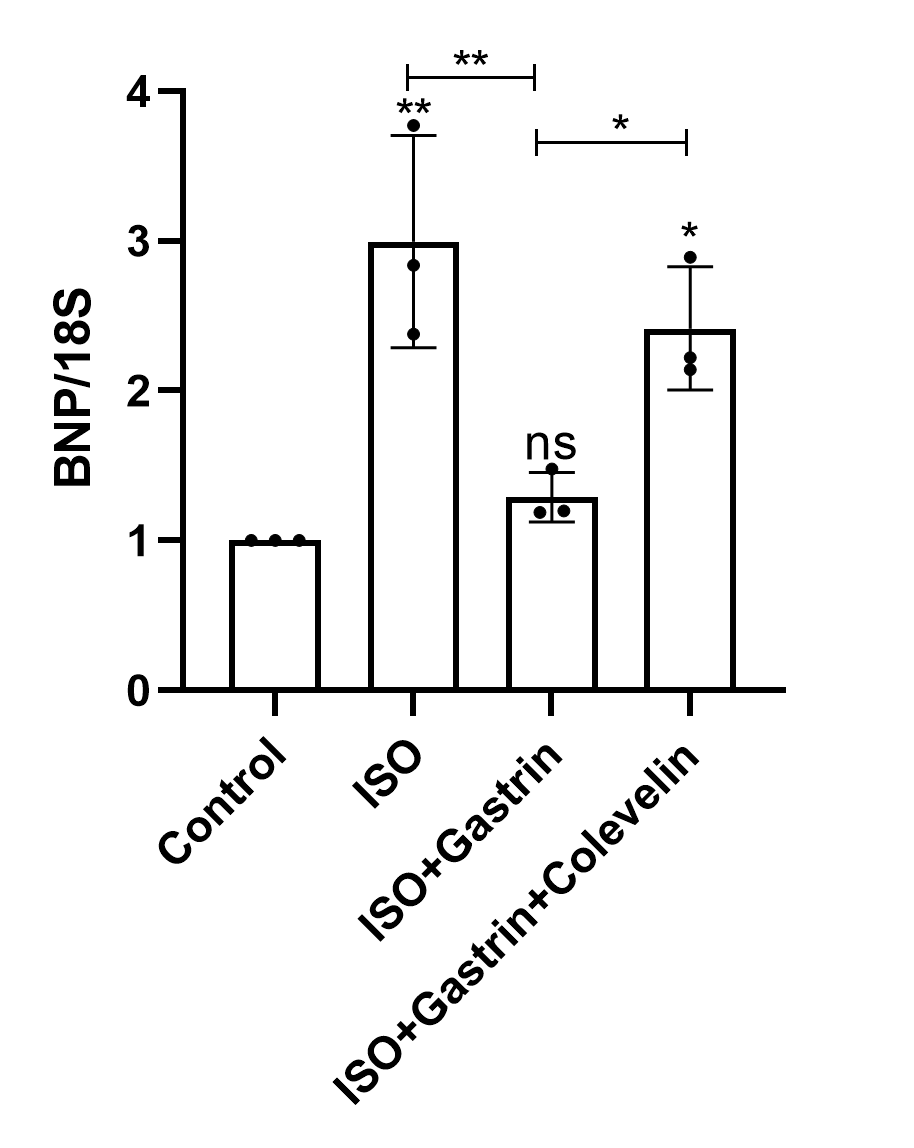


**The expression of BNP in H9c2 cells**

**Fig 7.(P)The level of of p-STAT3/STAT3 after the addition of STAT3 agonist colevelin in H9c2 myocardial cells**

Table 99 The grayscale intensity of P-STAT3/STAT3

| groups | The grayscale intensity of STAT3 | The grayscale intensity of P-STAT3 | P-STAT3/STAT3 |
| --- | --- | --- | --- |
| Control | 3,644,331.00 | 7,974,675.00 | 2.188241134 |
|  | 3,593,315.00 | 5,007,200.00 | 1.393476497 |
|  | 2,638,918.00 | 6,420,884.00 | 2.433150253 |
| ISO | 1,483,412.00 | 8,512,908.00 | 5.738734755 |
|  | 3,007,409.00 | 9,794,411.00 | 3.256760554 |
|  | 1,386,522.00 | 7,996,330.00 | 5.767185807 |
| ISO+Gastrin | 3,043,844.00 | 6,866,988.00 | 2.256024947 |
|  | 3,253,430.00 | 6,918,596.00 | 2.126554436 |
|  | 3,654,318.00 | 7,291,725.00 | 1.995372324 |
| ISO+Gastrin+Colevelin | 2,171,803.00 | 9,367,672.00 | 4.31331571 |
|  | 2,719,278.00 | 10,997,069.00 | 4.044113548 |
|  | 2,068,326.00 | 8,928,166.00 | 4.316614499 |

Table 100 The comparison of P-STAT3/STAT3 across each group

|  | groups | P-STAT3/STAT3  Mean±SD | F-value | P-value |
| --- | --- | --- | --- | --- |
| ANOVA |  |  | 10.830 | 0.003 |
| Multiple comparisons | Control | 2.00±0.54 |  | 0.008 |
|  | ISO | 4.92±1.44 |  |  |
|  | Control | 2.00±0.54 |  | 0.997 |
|  | ISO+gatrin | 2.13±0.13 |  |  |
|  | Control | 2.00±0.54 |  | 0.033 |
|  | ISO+gastrin+Colevelin | 4.22±0.16 |  |  |
|  | ISO | 4.92±1.44 |  | 0.010 |
|  | ISO+gastrin | 2.13±0.13 |  |  |
|  | ISO+gastrin | 2.13±0.13 |  | 0.043 |
|  | ISO+gastrin+Colevelin | 4.22±0.16 |  |  |


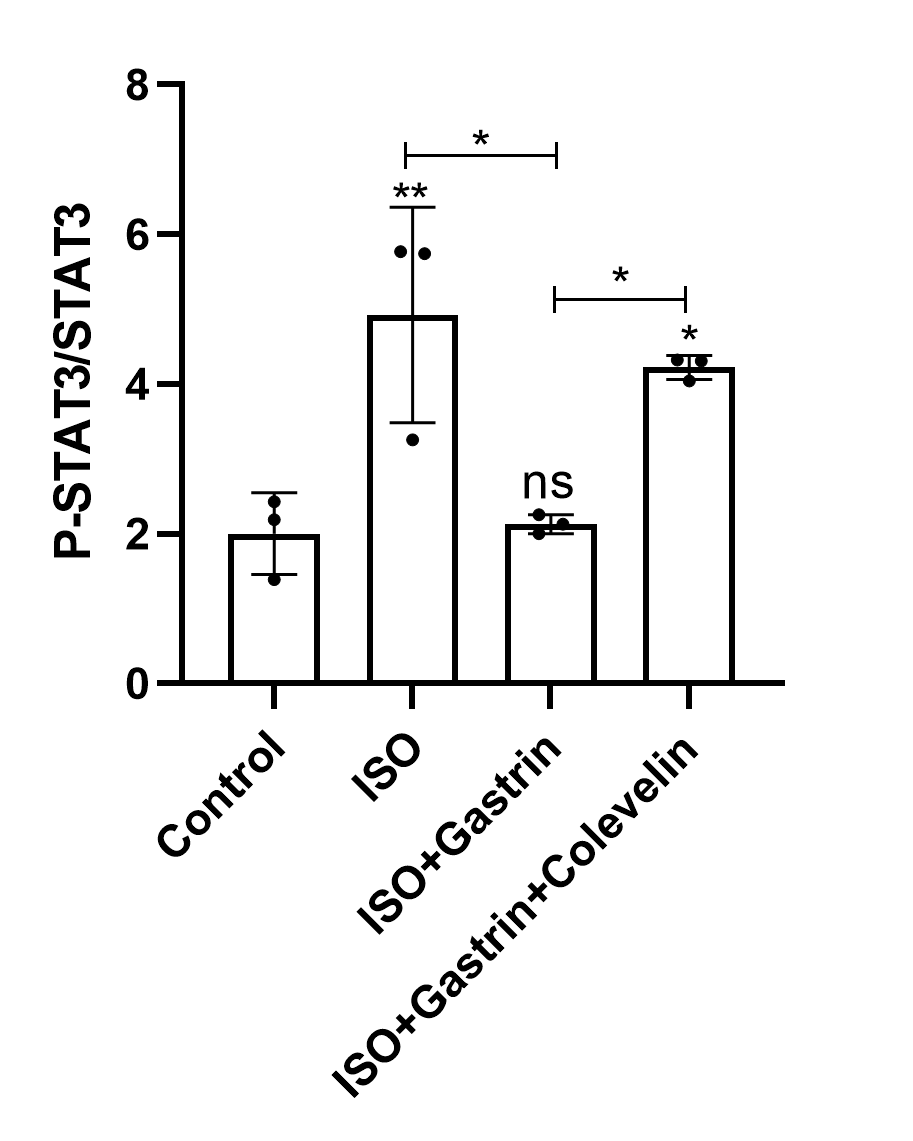


**The level of of p-STAT3/STAT3**

The original data of the PCR experiment mentioned above is in the supplementary ducument(**<supplement of supporting information.xlsx>**).
