# Supplementary figures and images for: Gastrin ameliorates heart failure and suppresses myocardial remodeling via the JAK2/STAT3 and ERK1/2 pathways
Source: PLoS One. 2026 Mar 5;21(3):e0343403. doi: 10.1371/journal.pone.0343403 (PMC12962455; doi:10.1371/journal.pone.0343403)

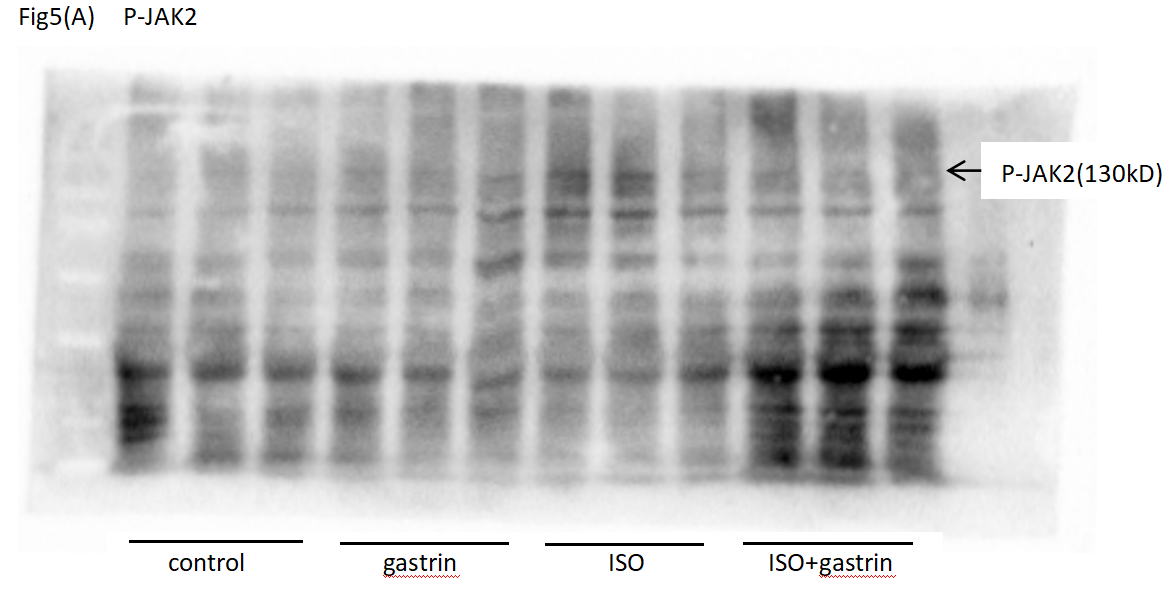


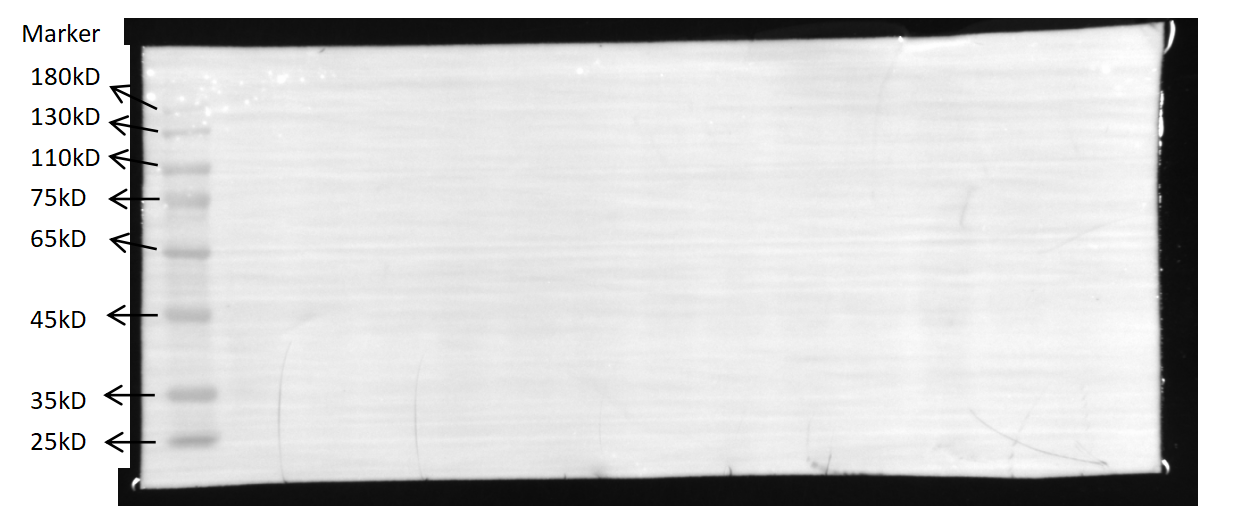


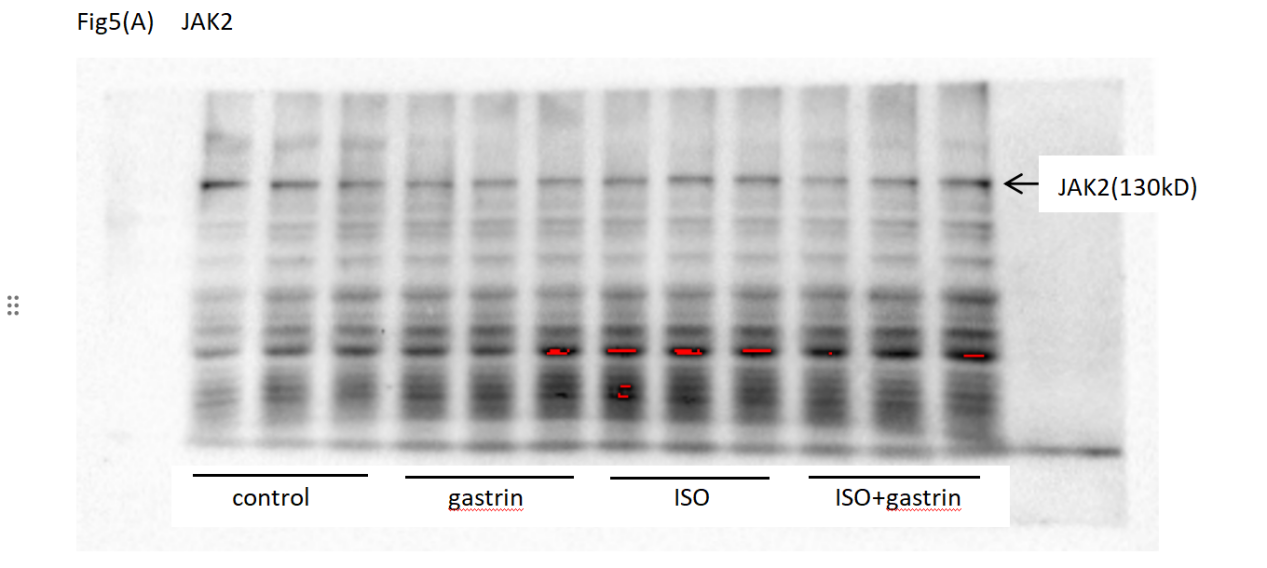


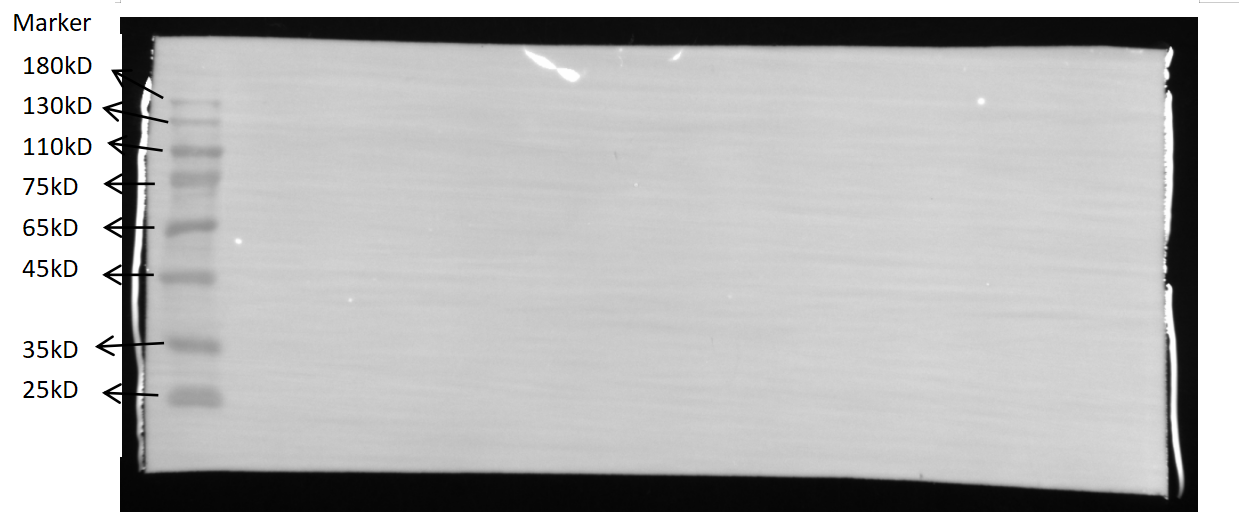


Fig5(A) P-STAT3


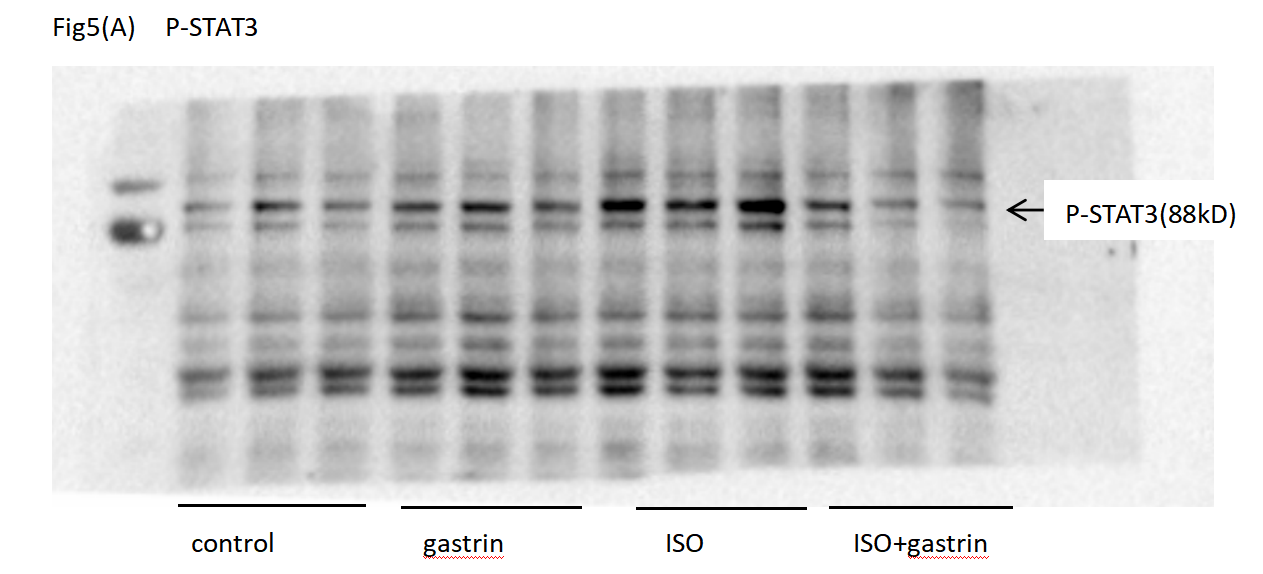


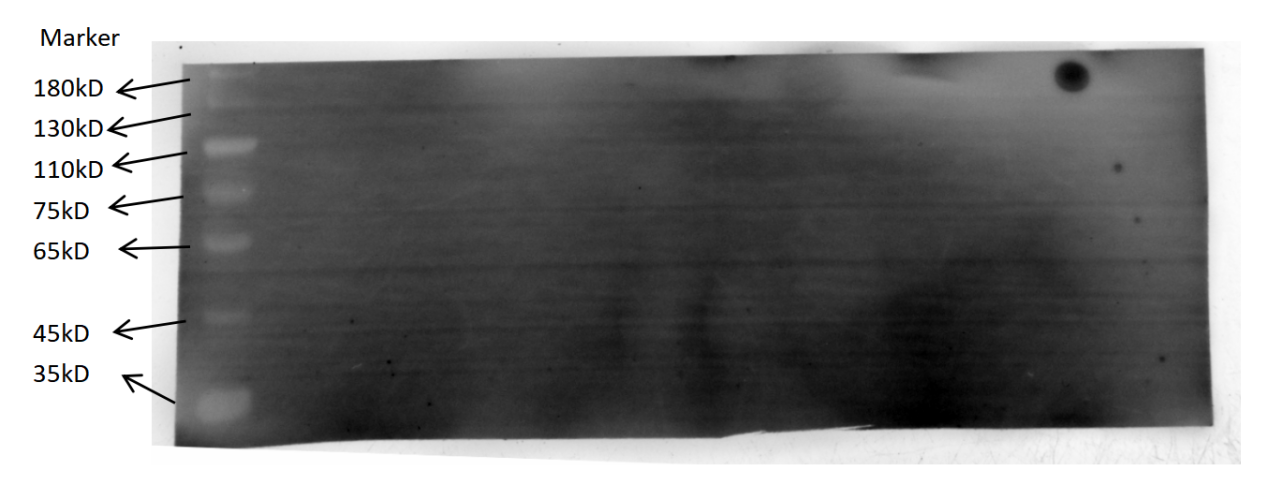


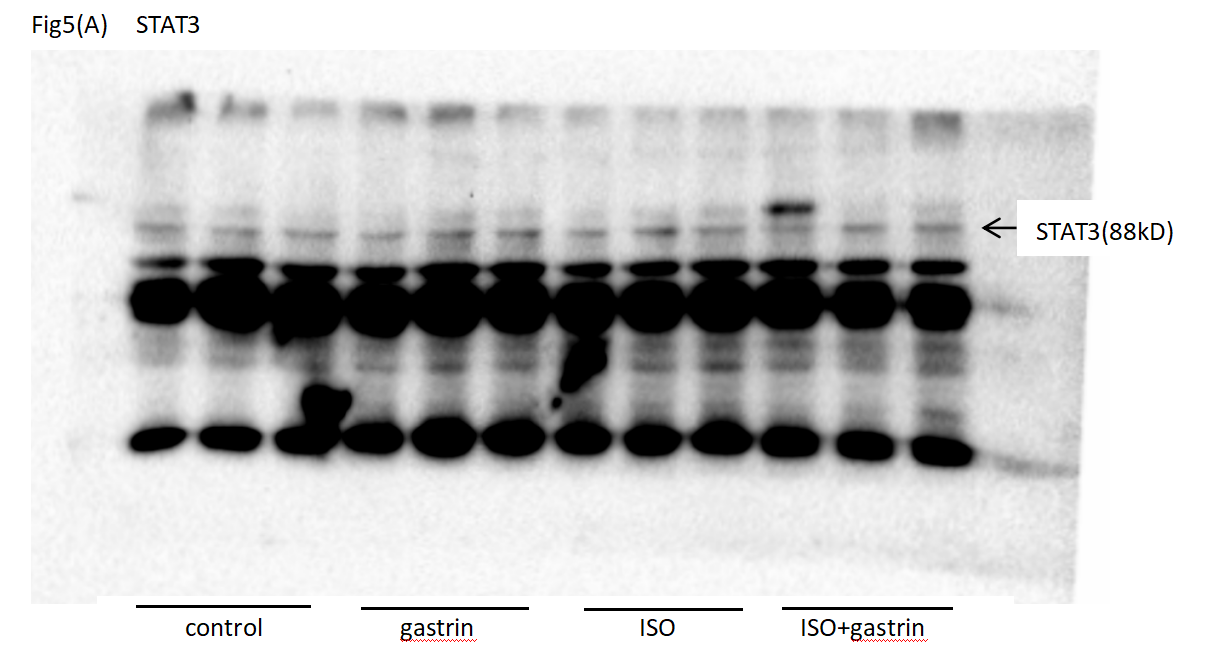


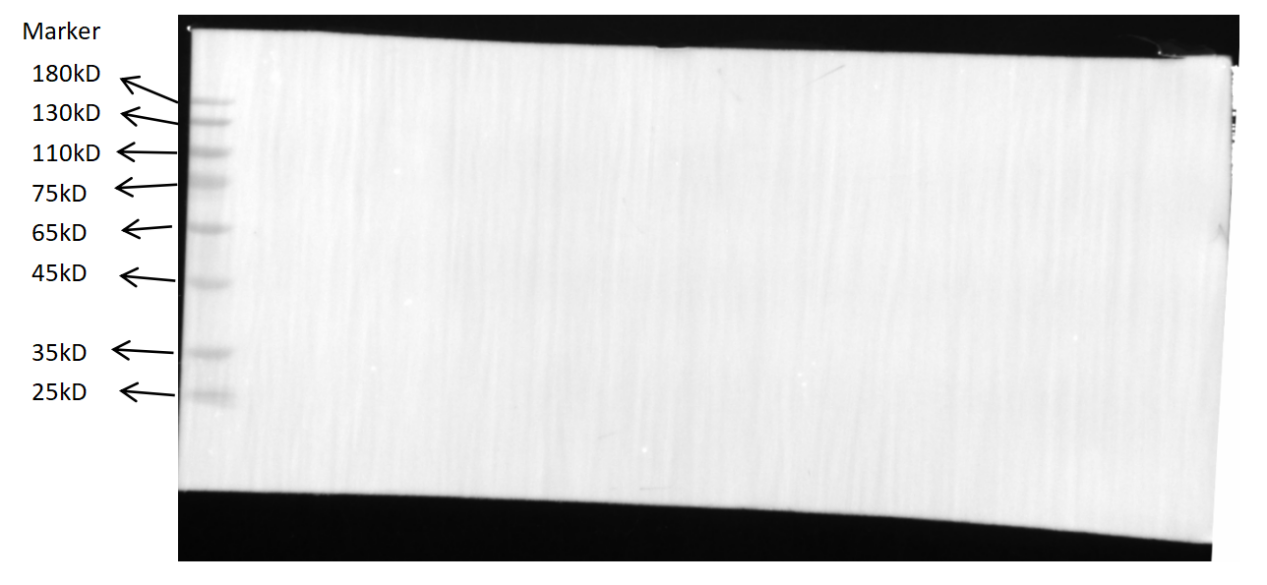


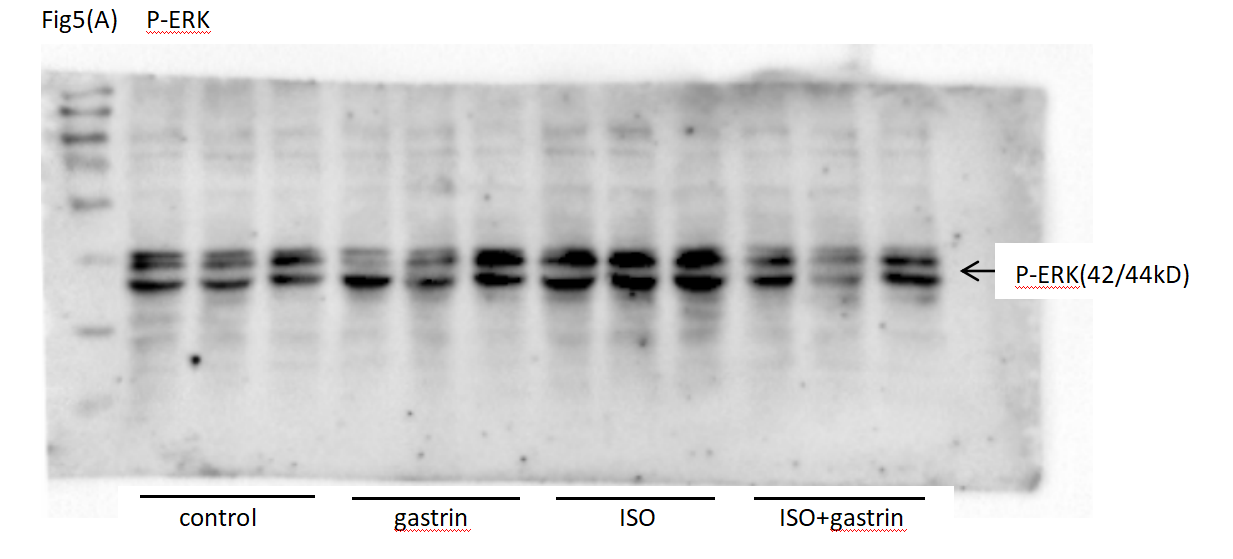


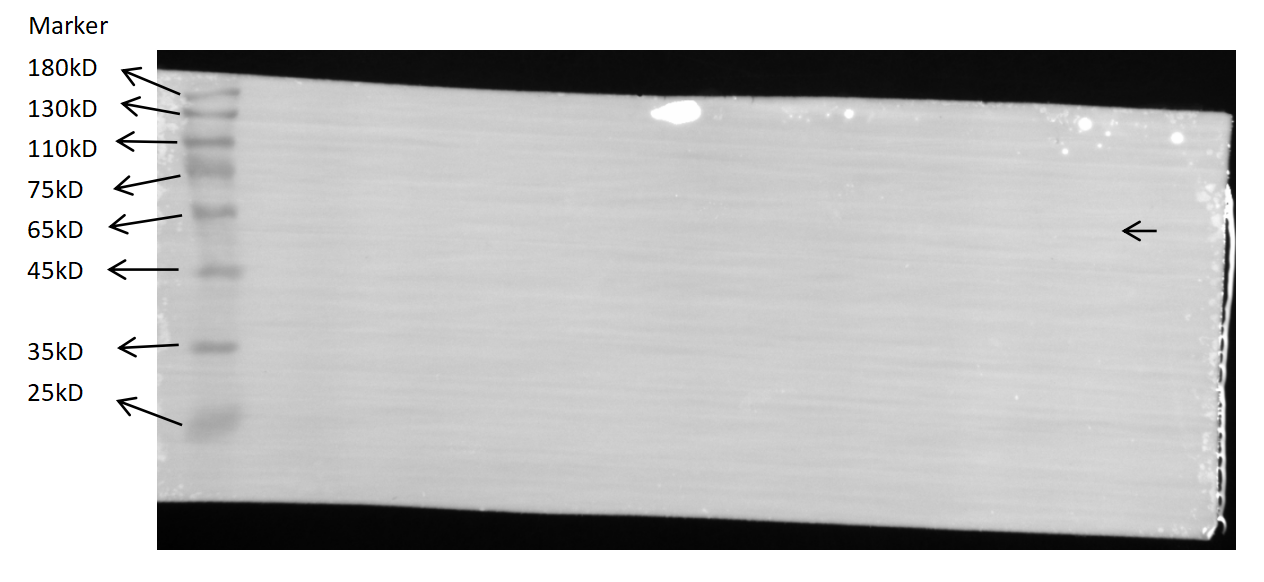


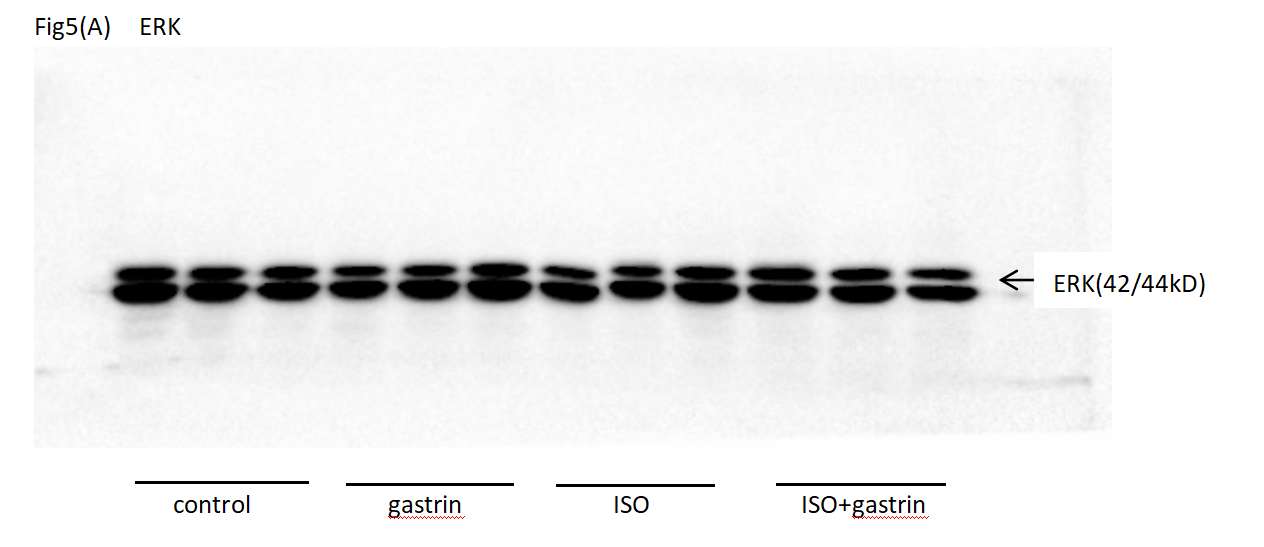


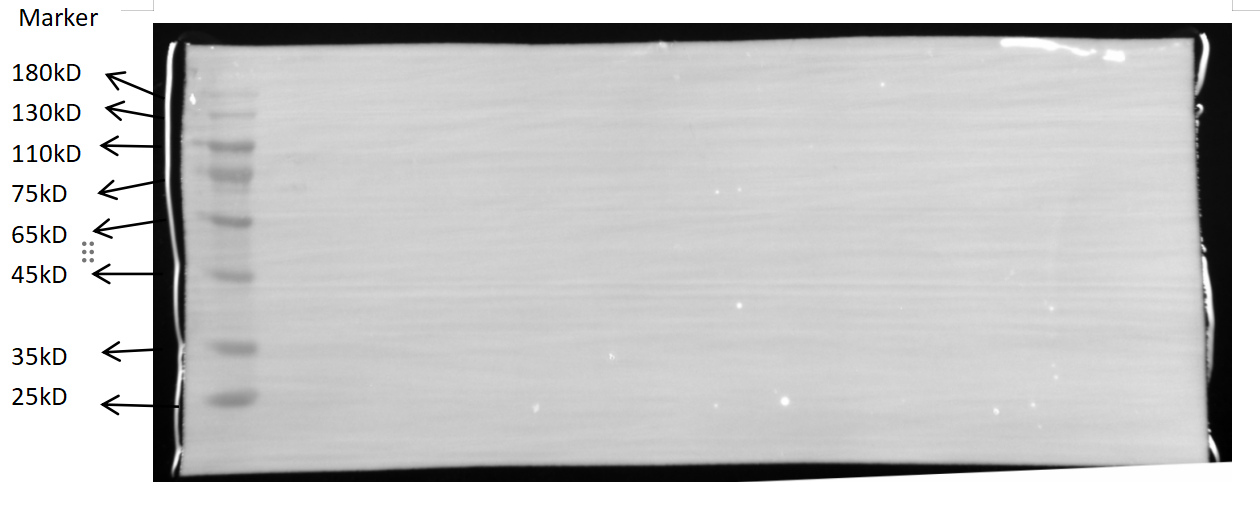


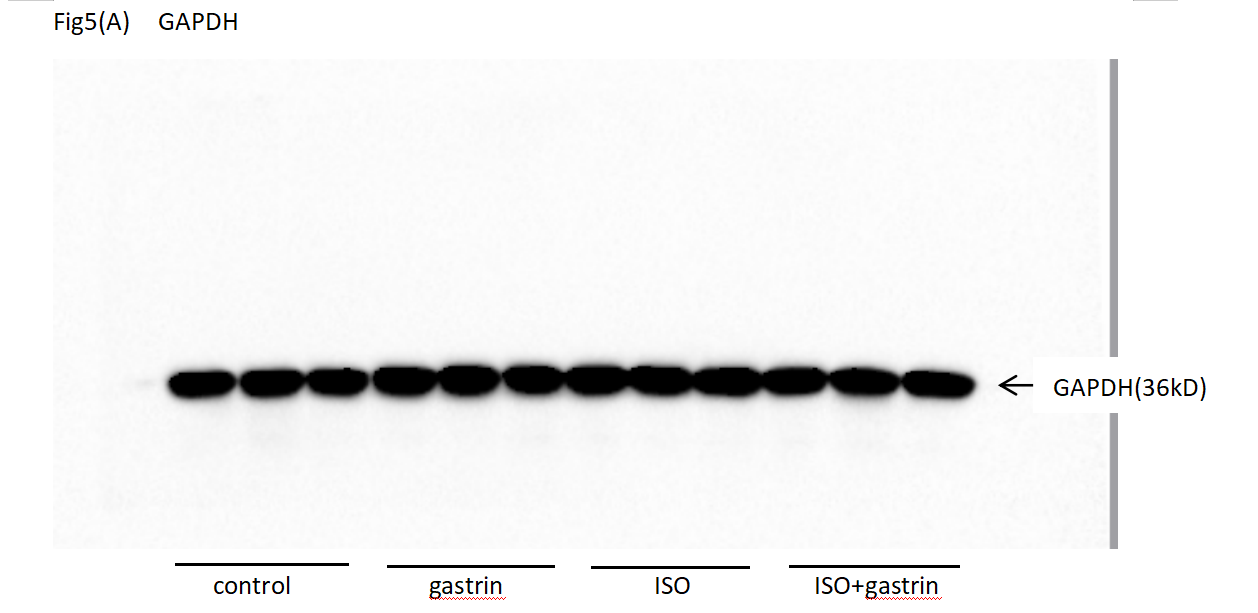


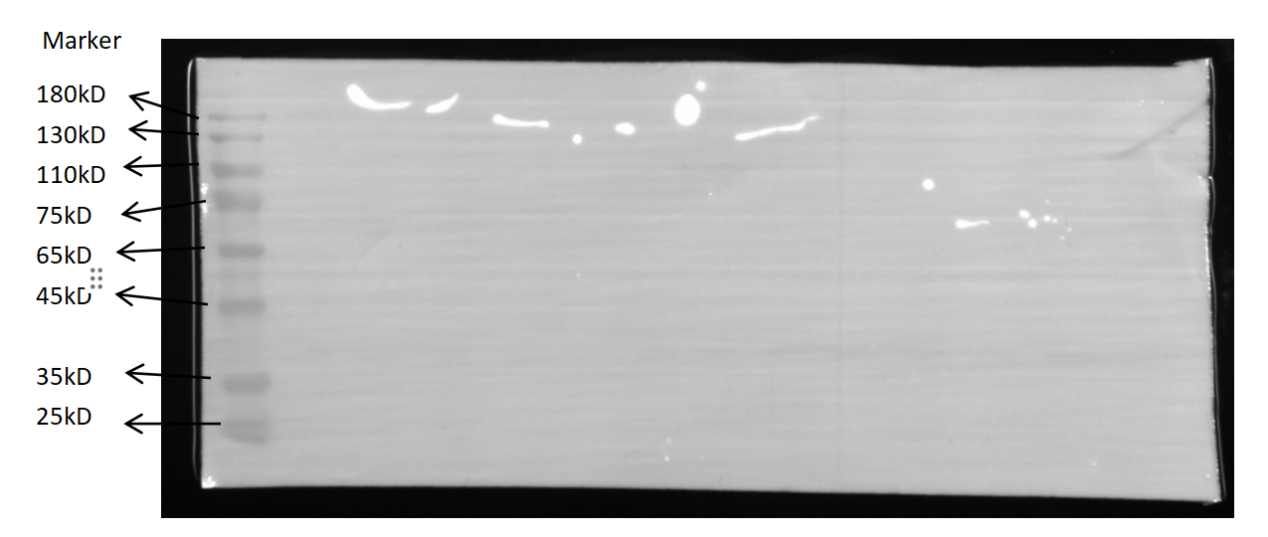


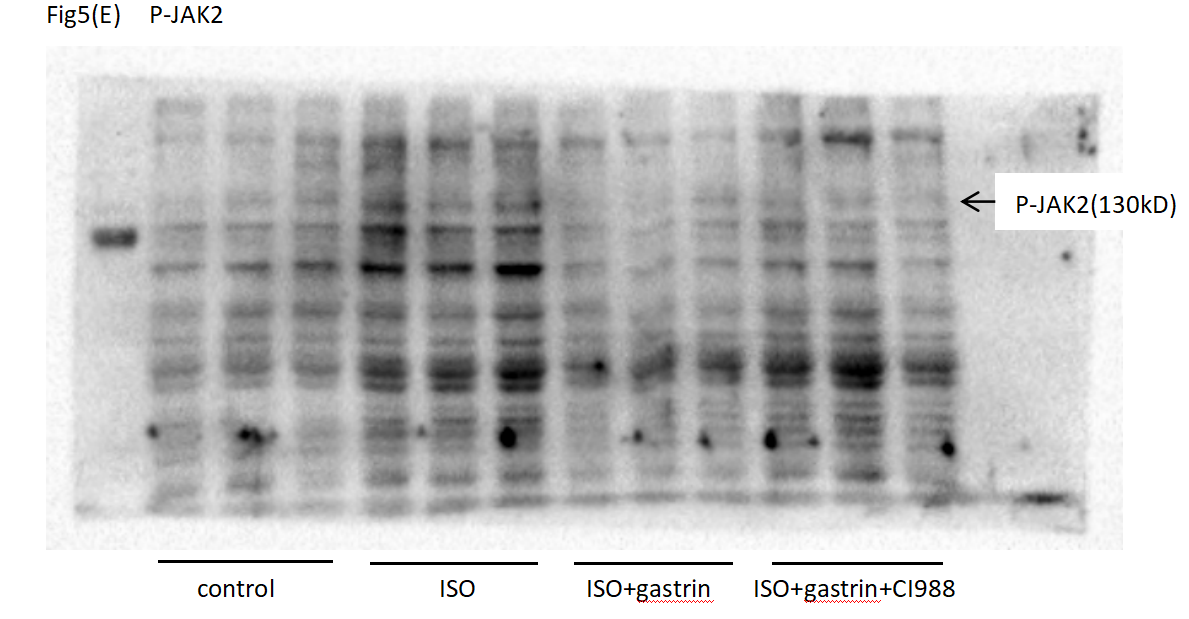


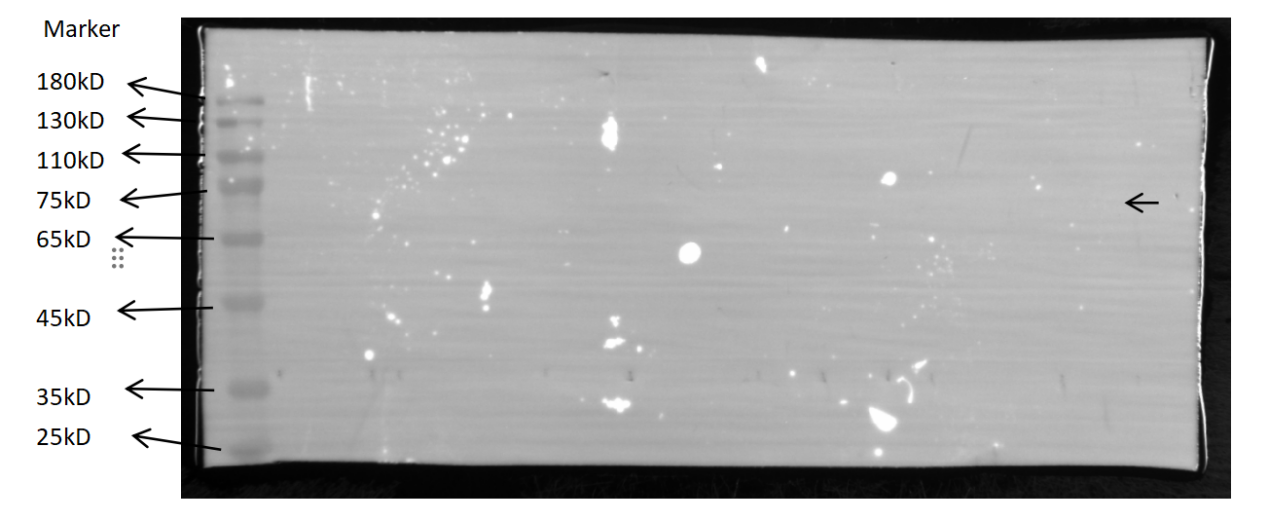


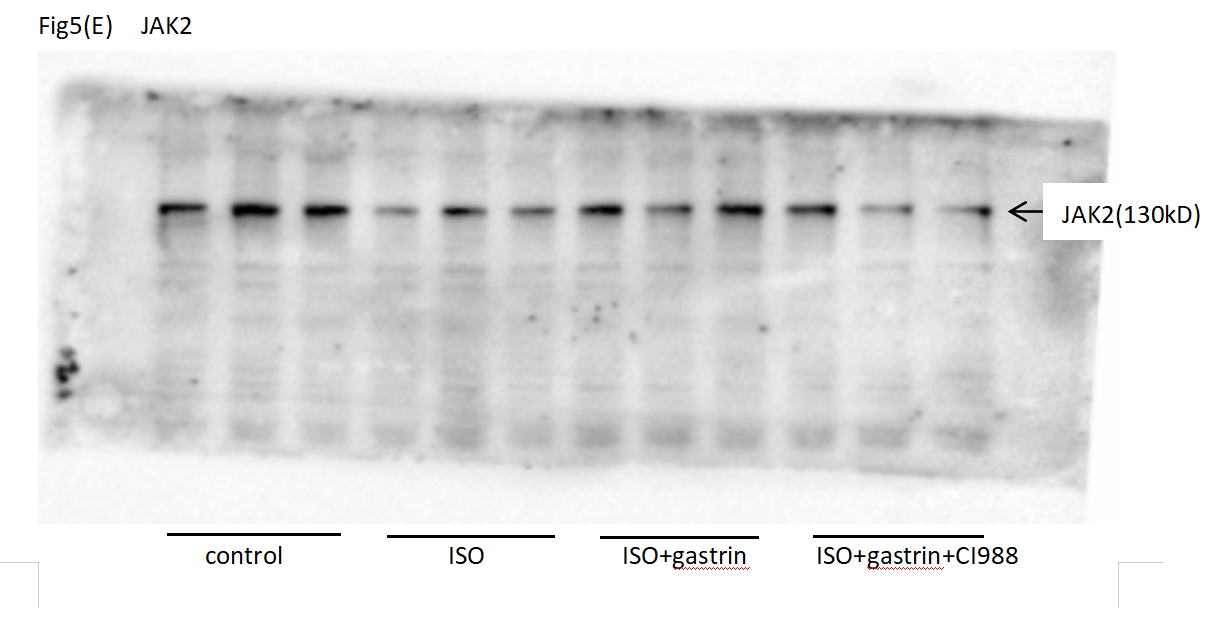


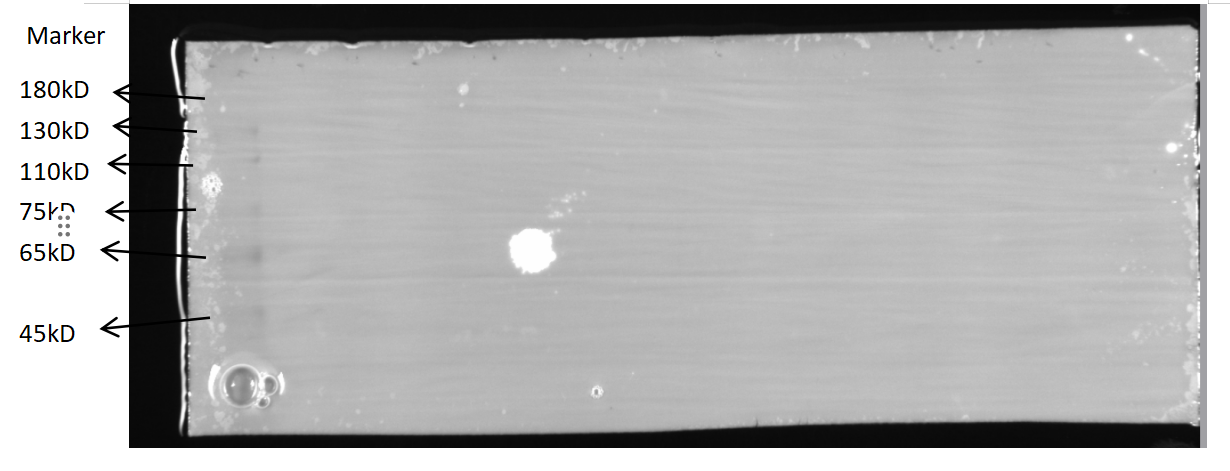


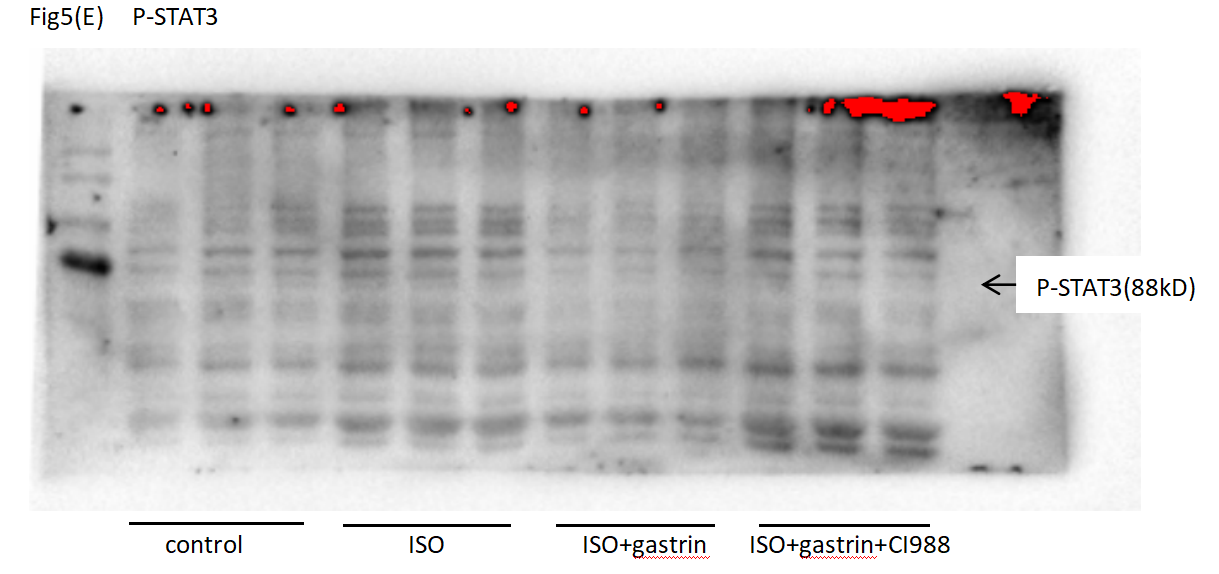


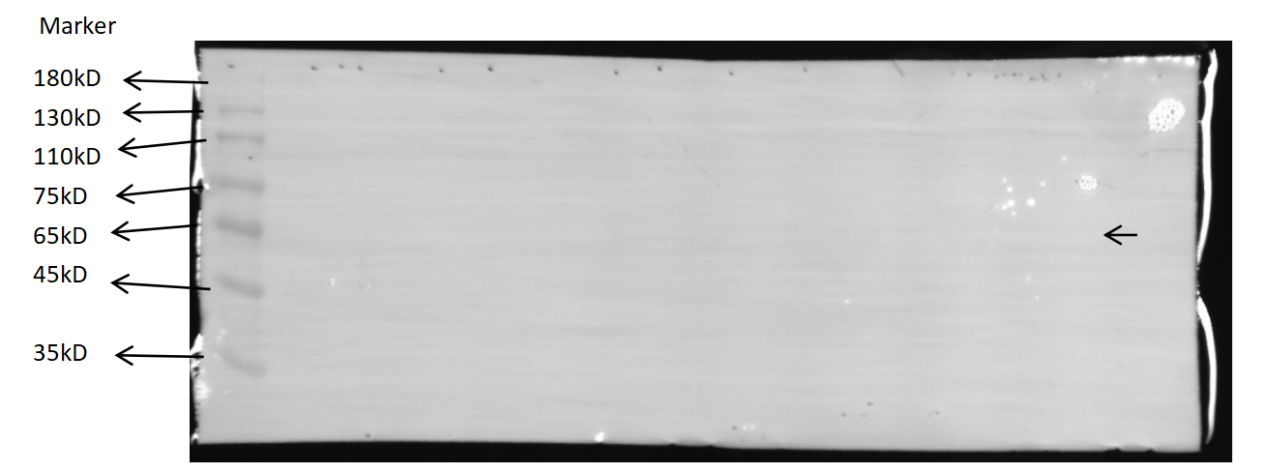


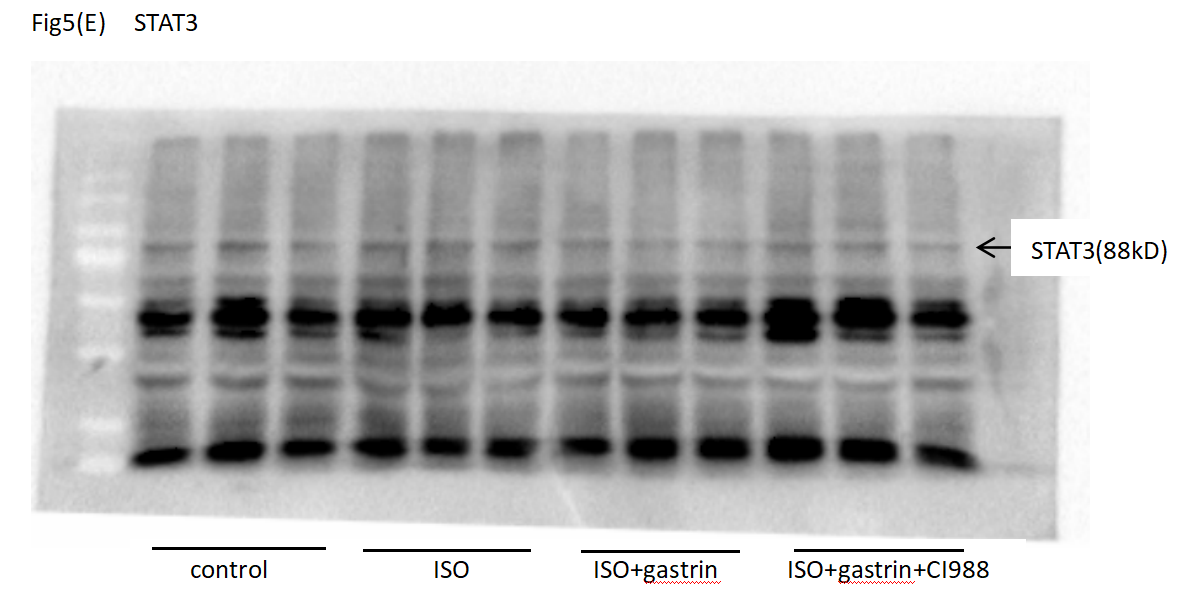


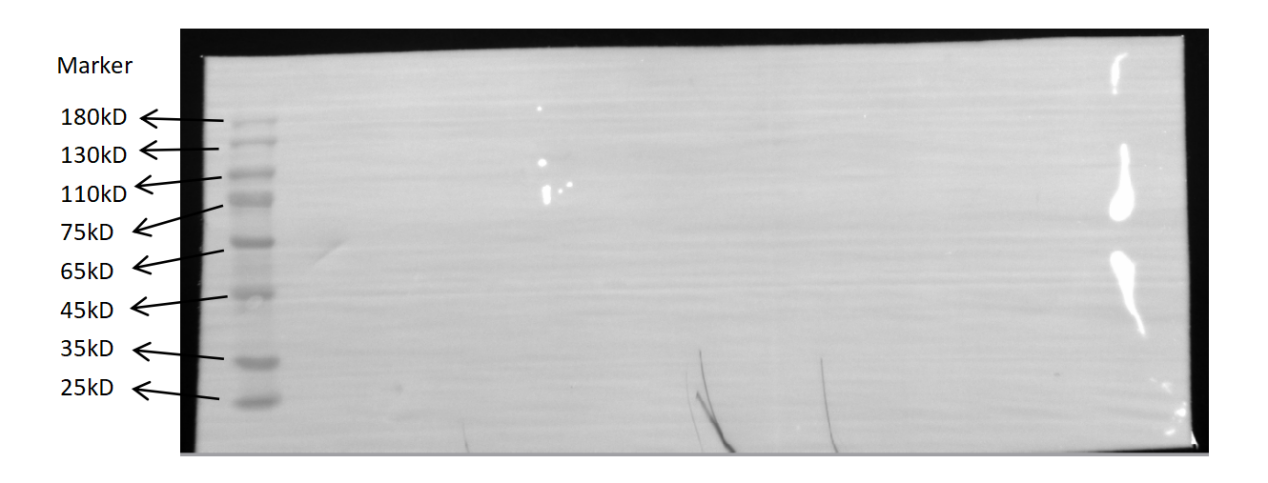


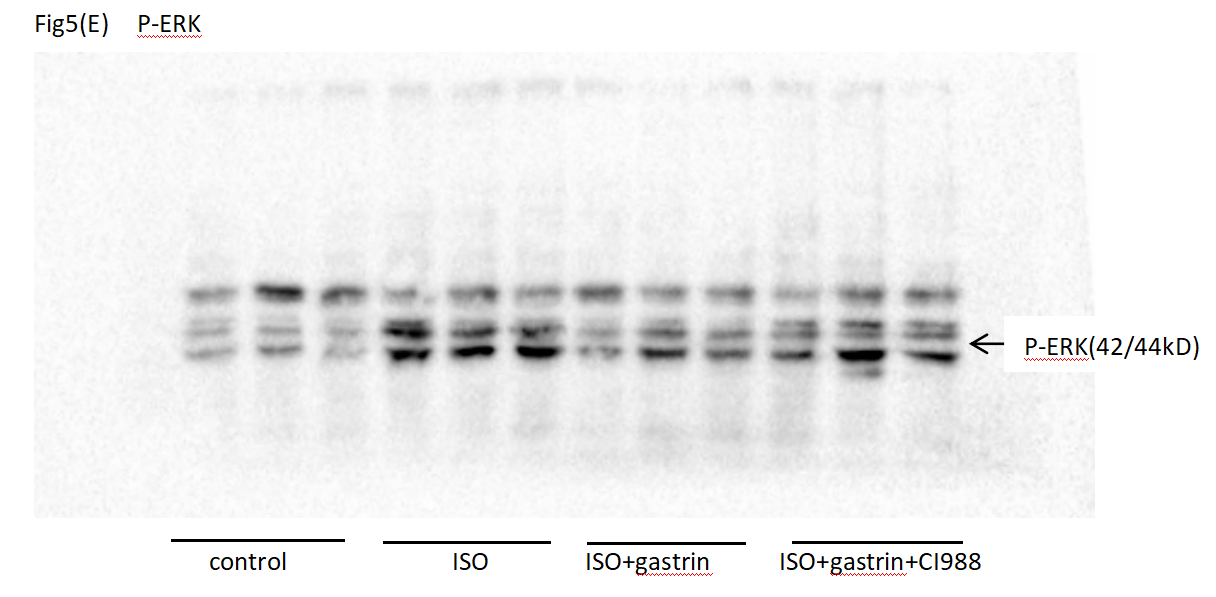


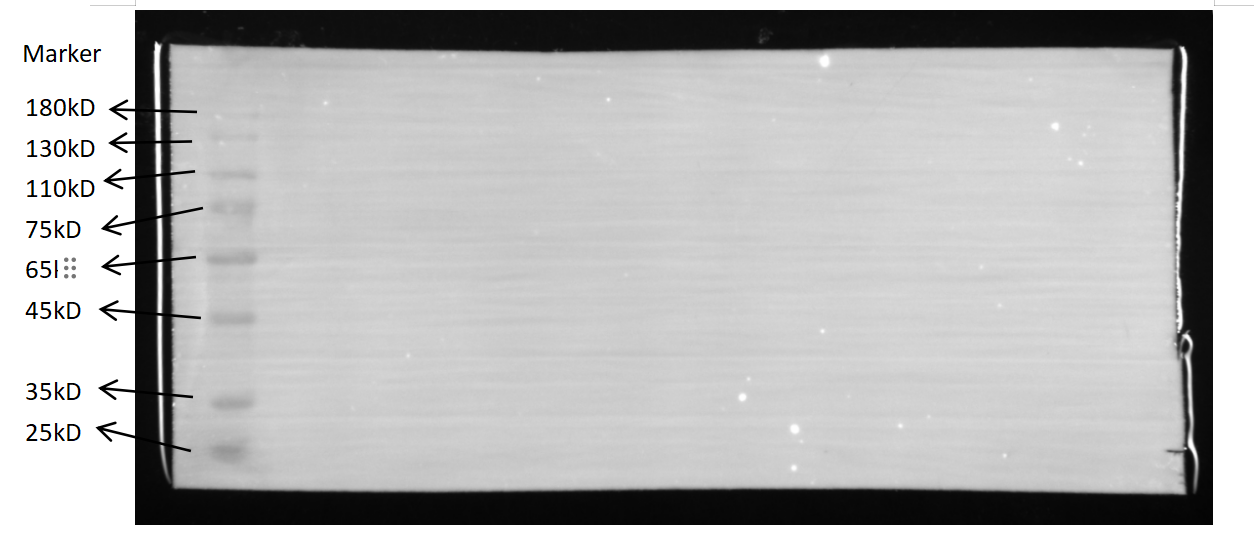


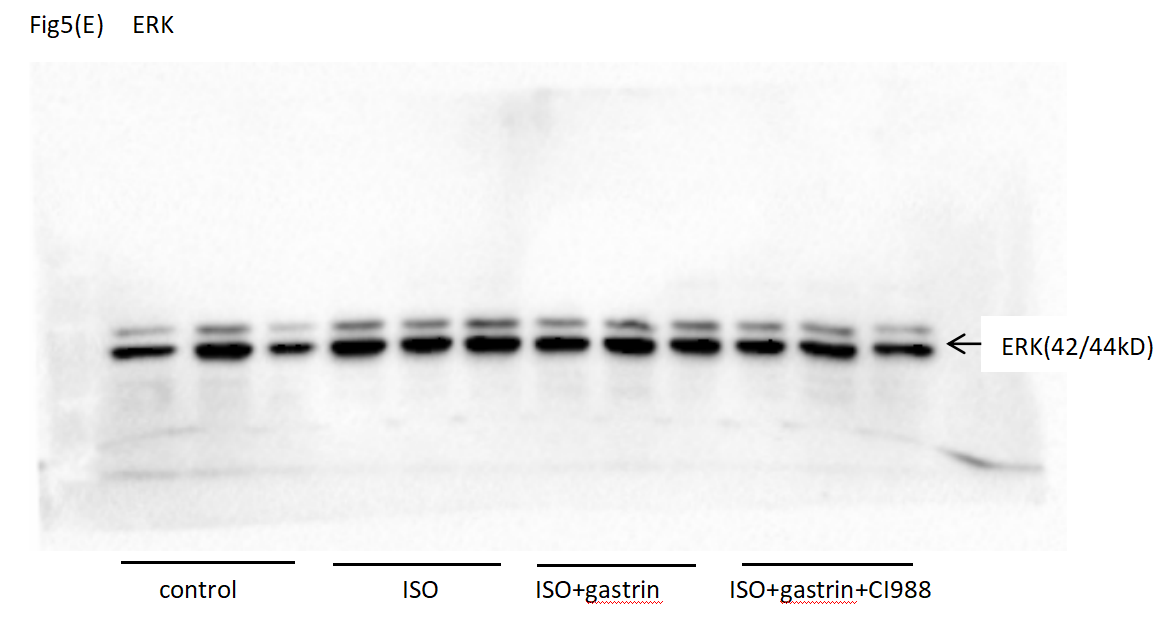


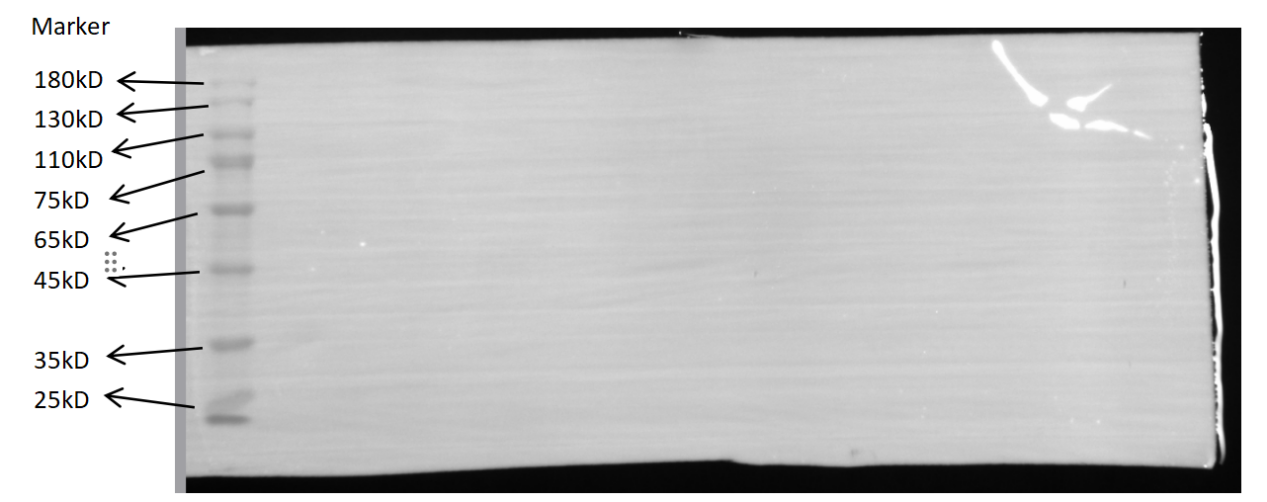


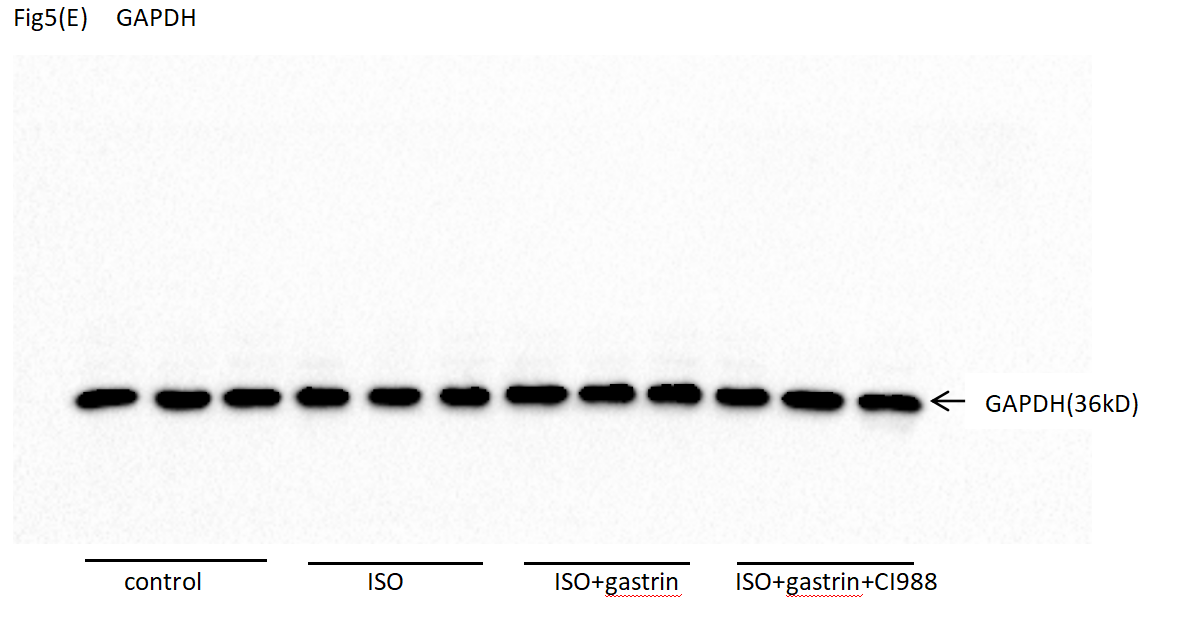


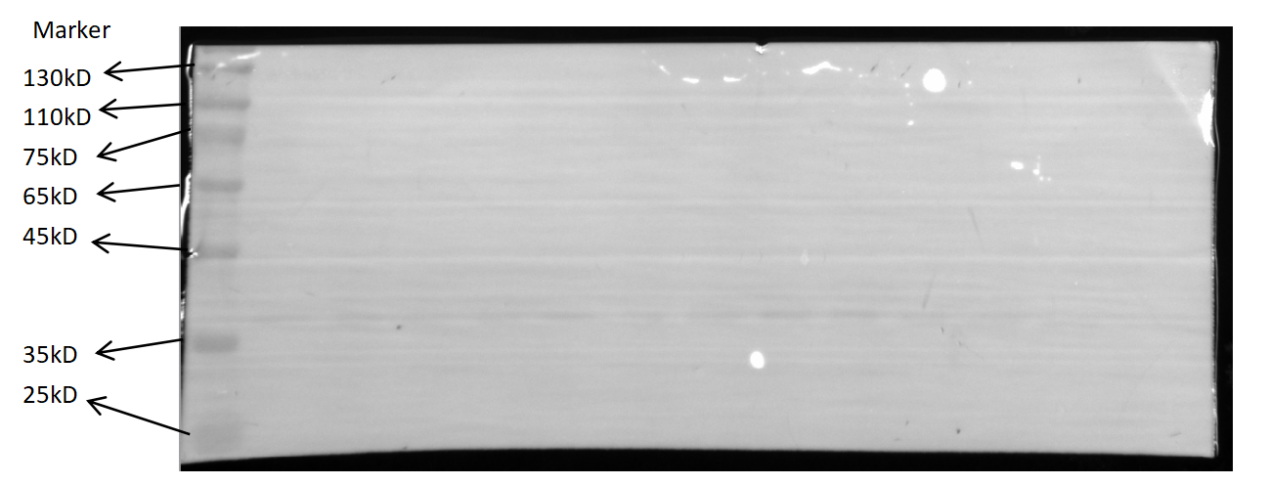


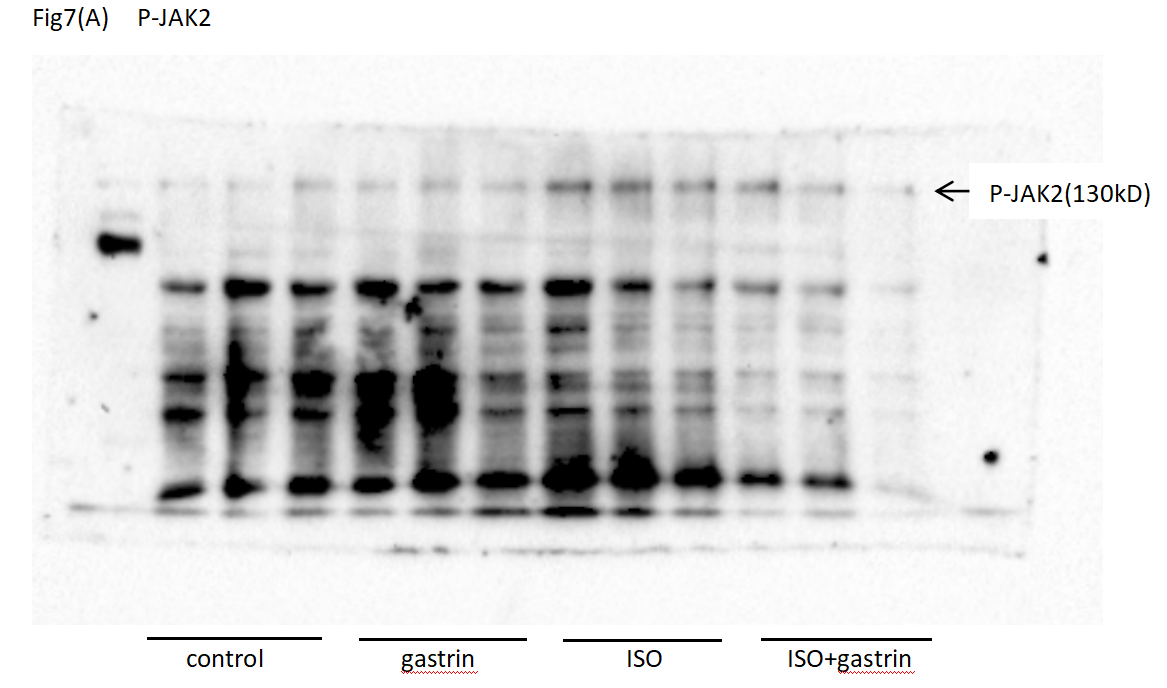


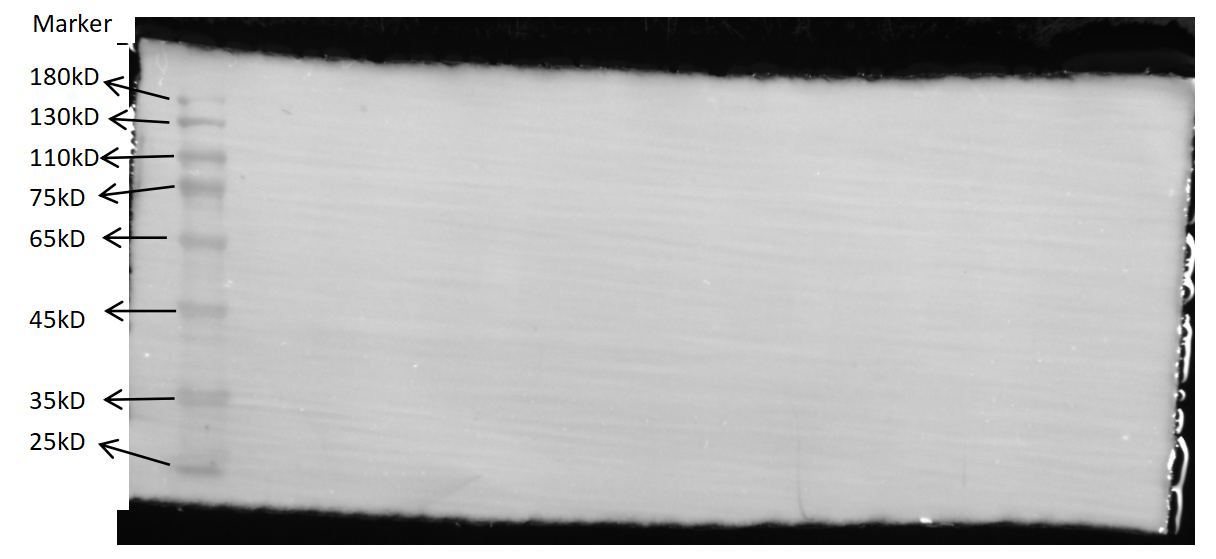


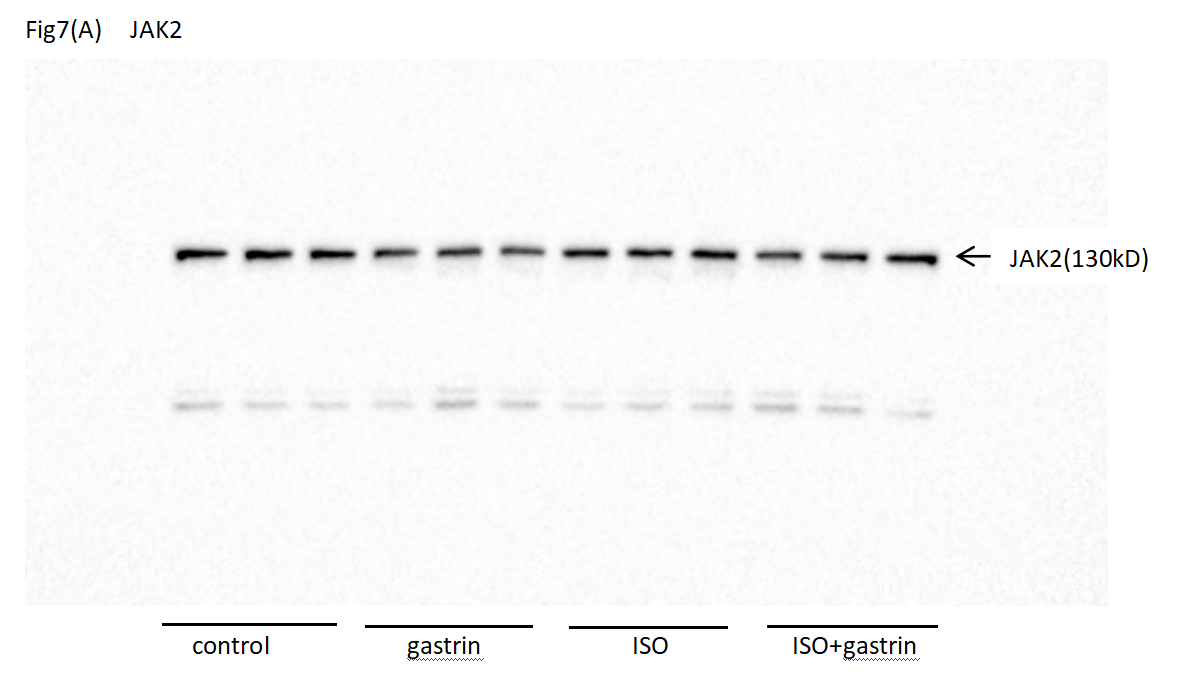


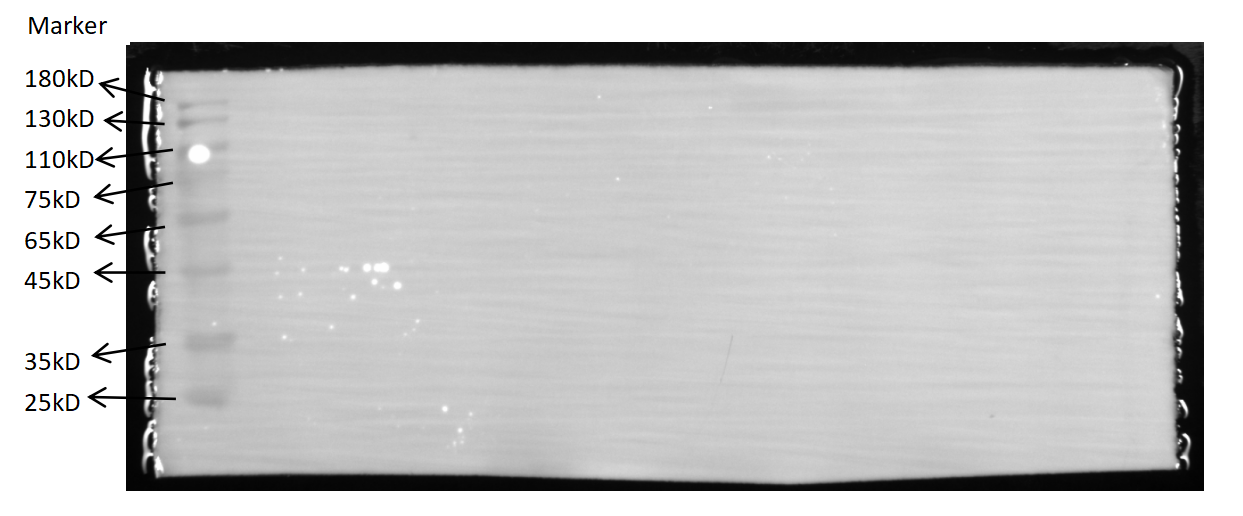


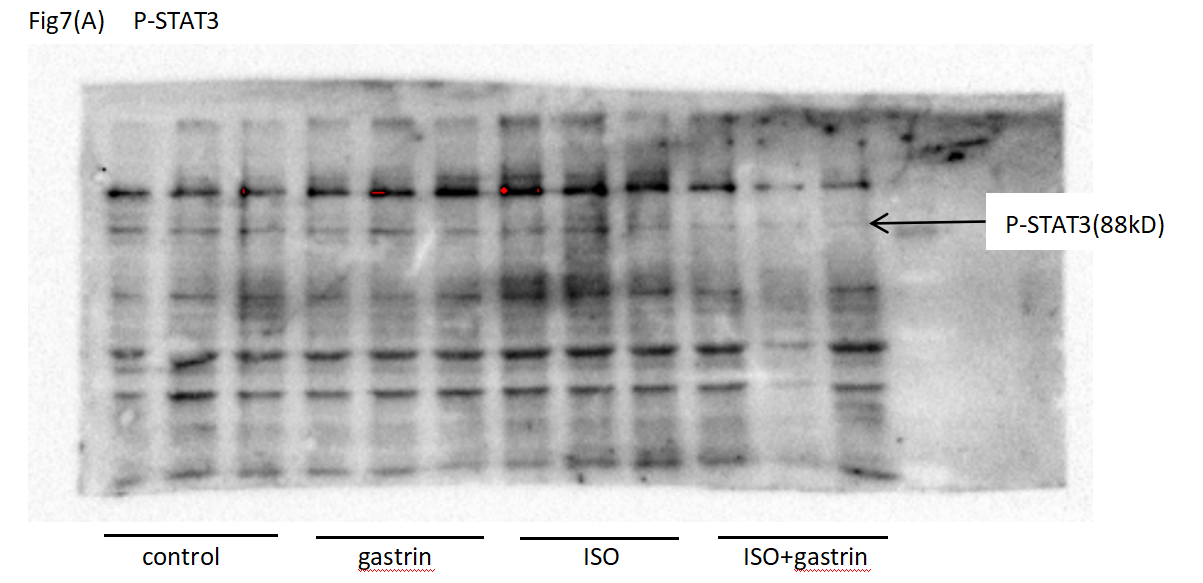


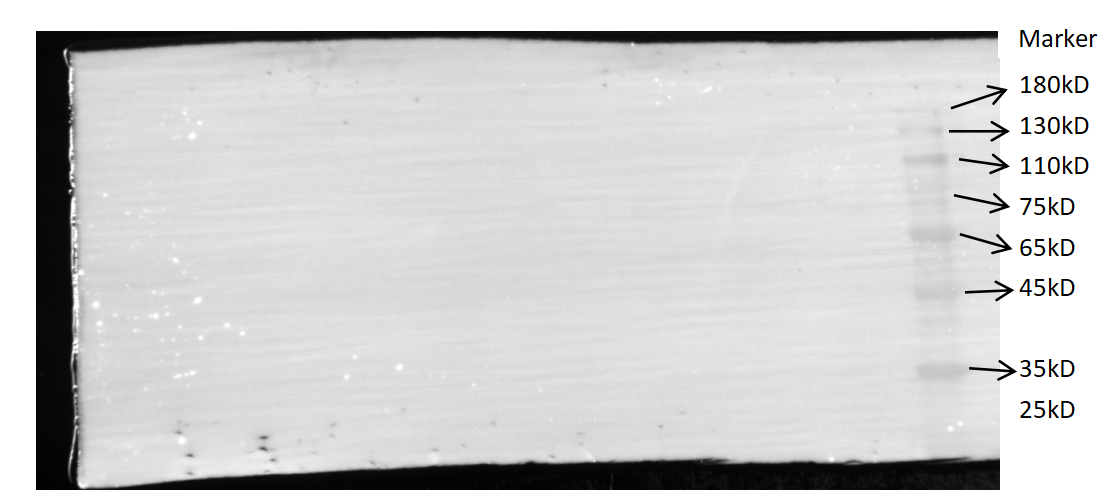


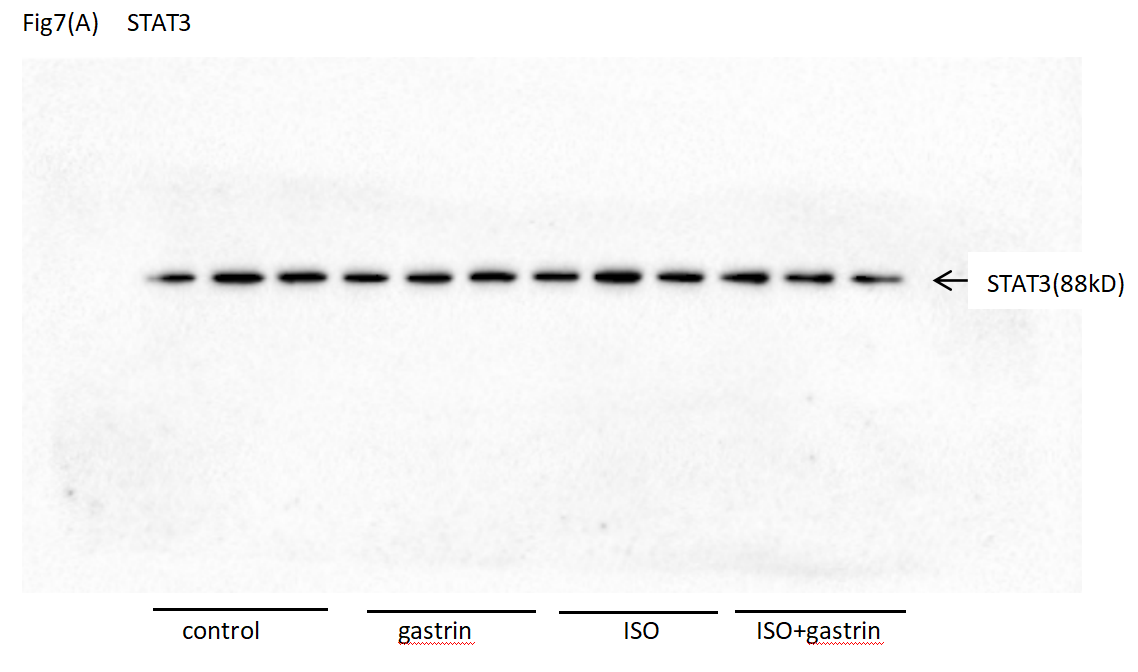


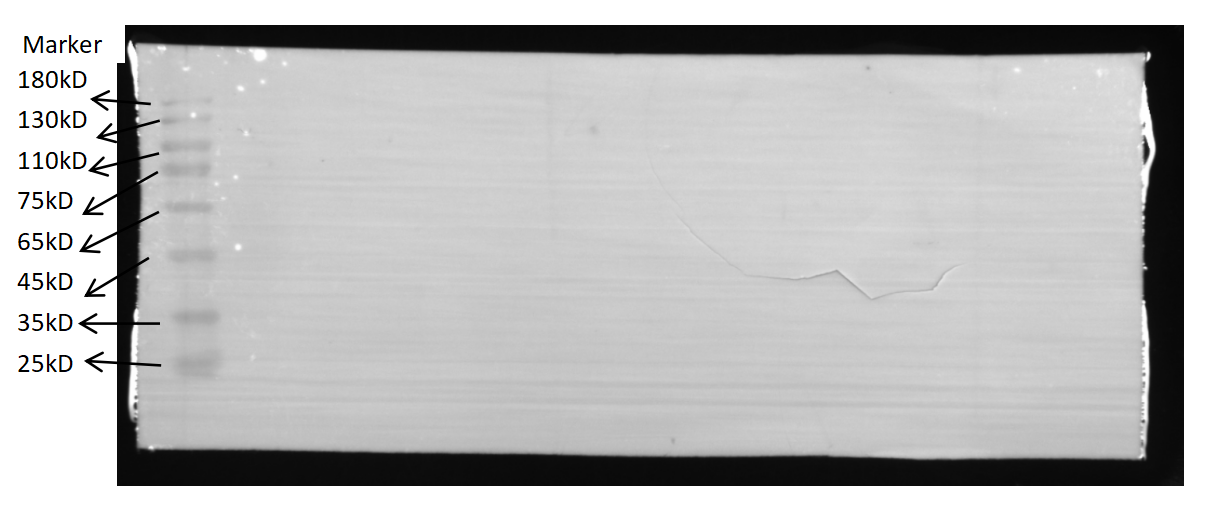


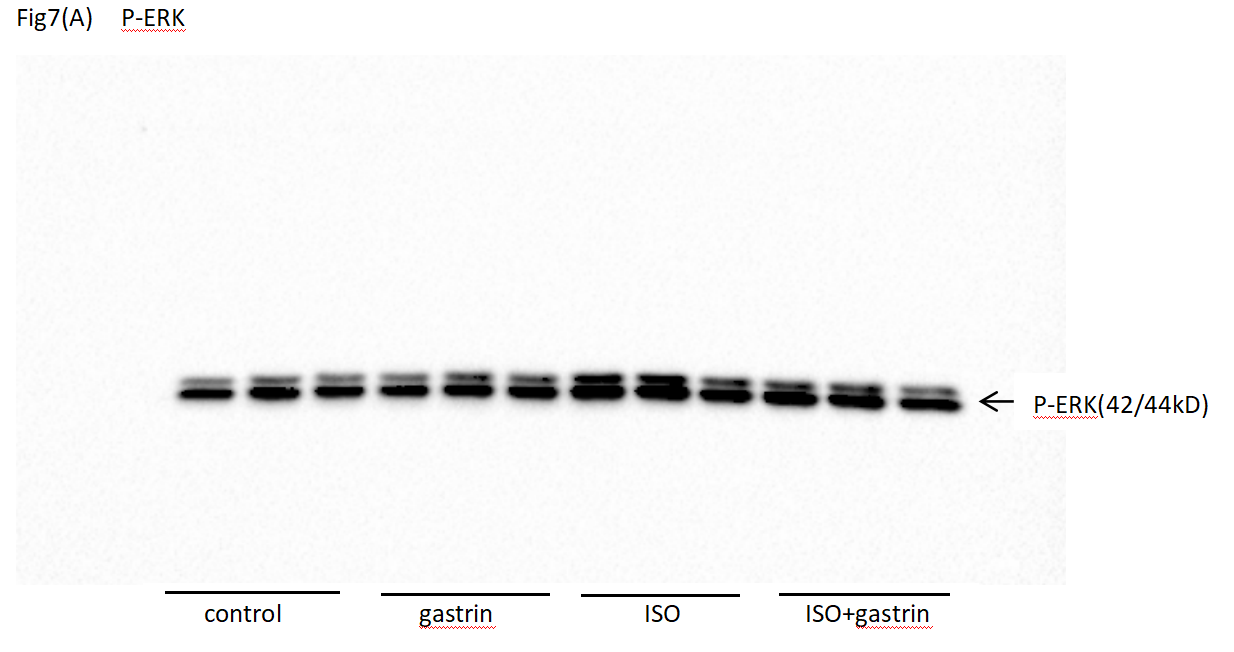


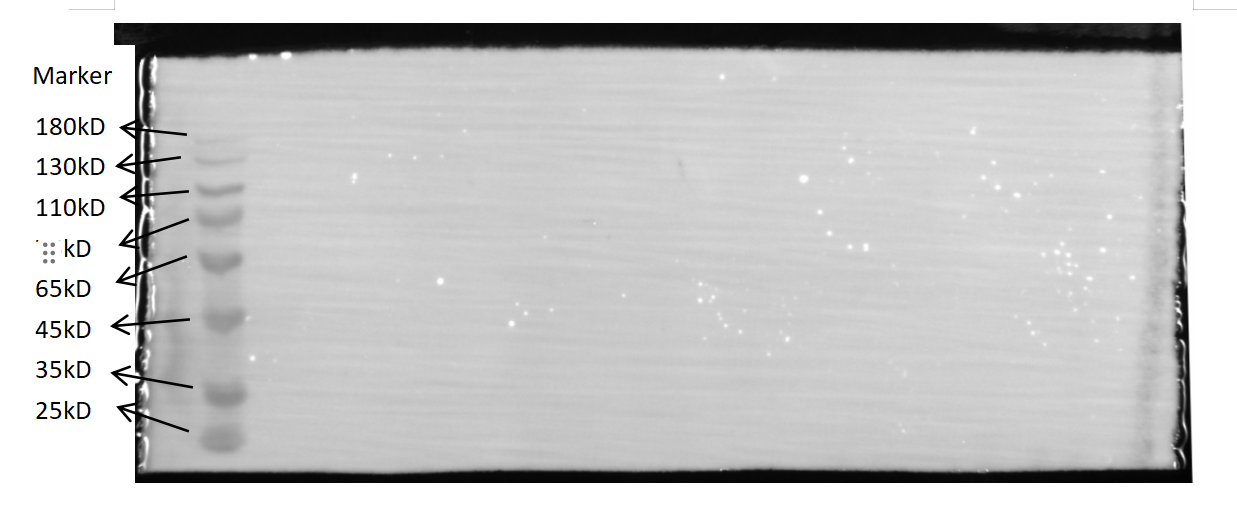


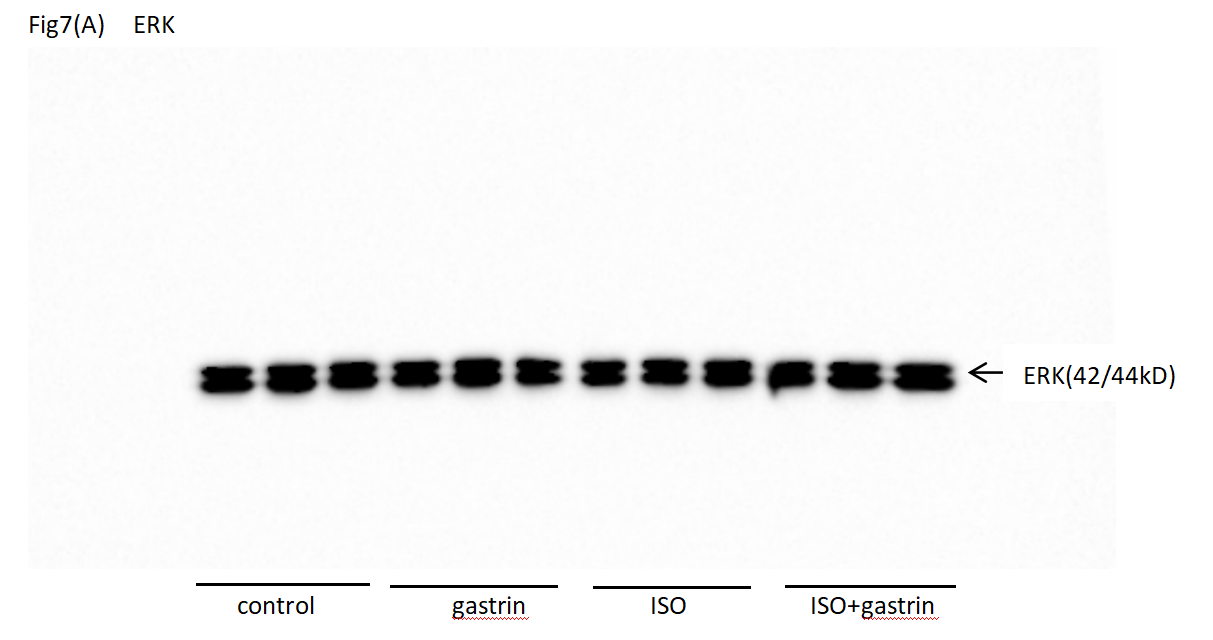


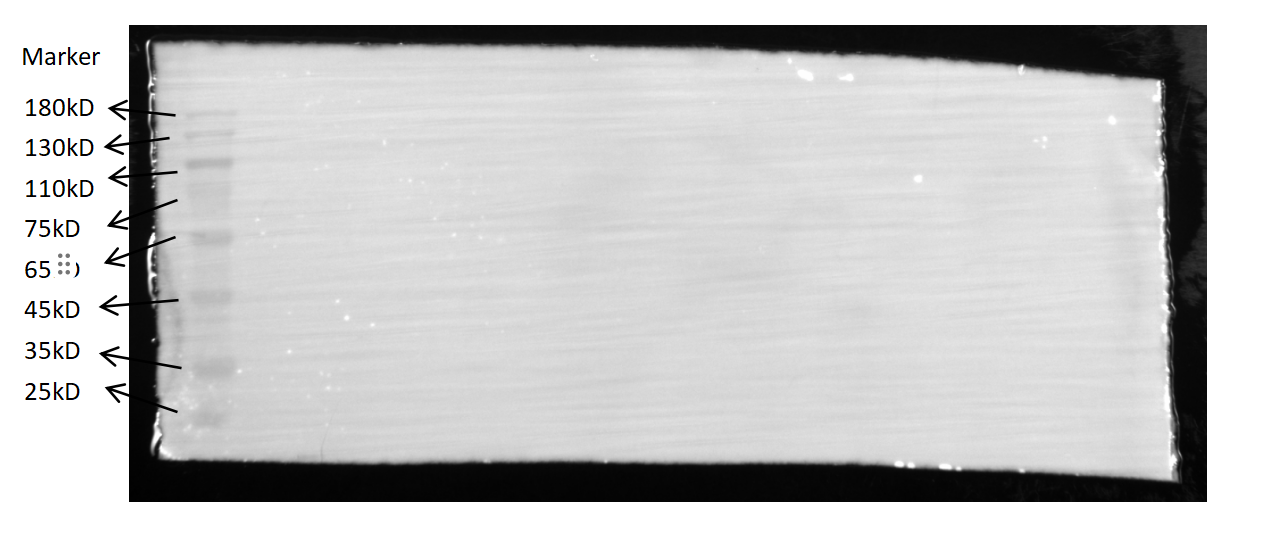


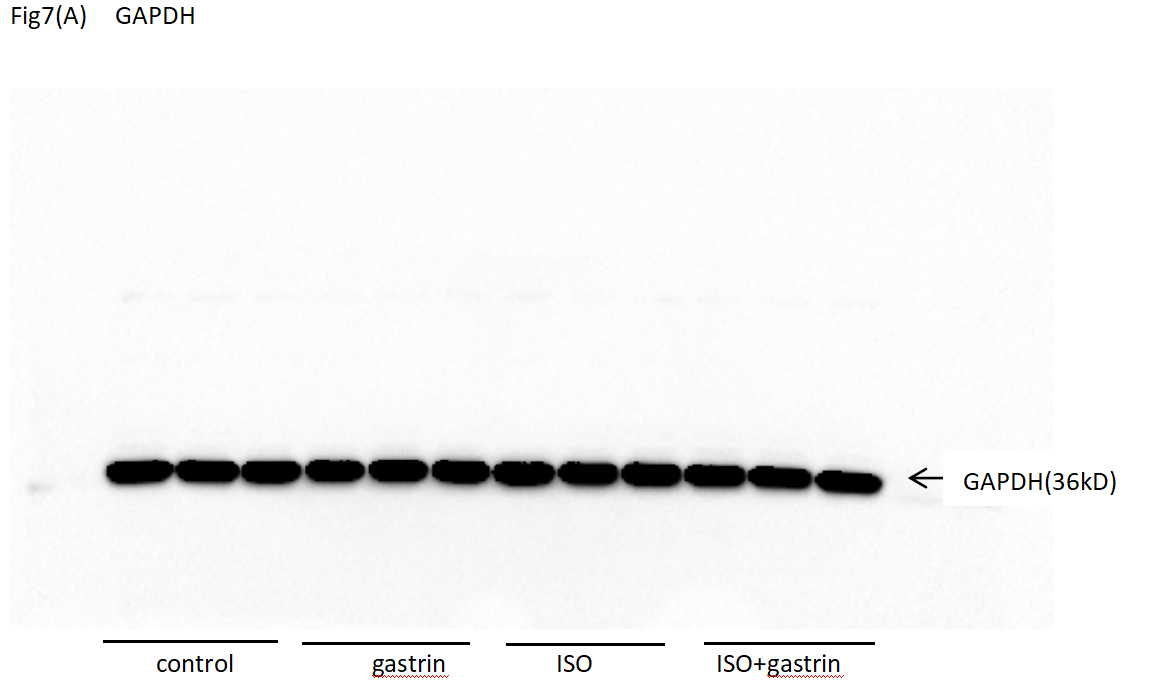


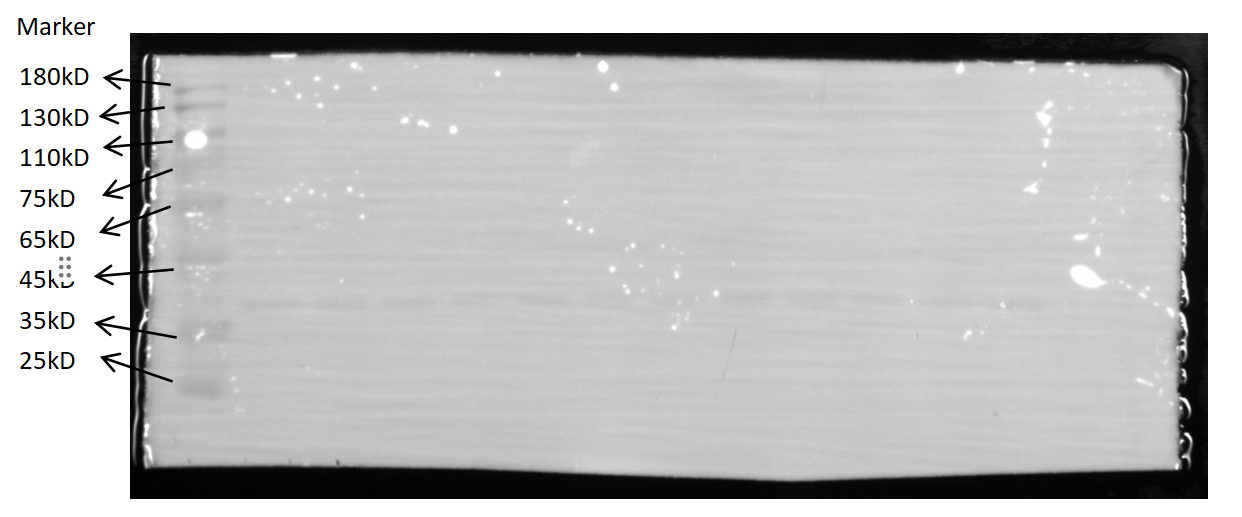


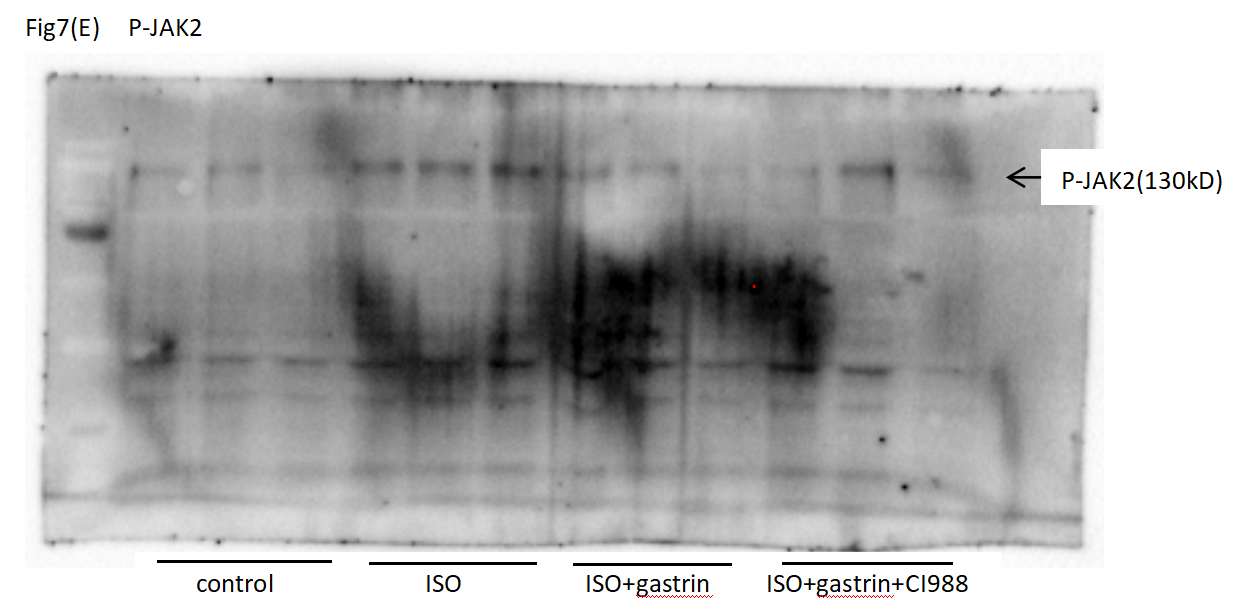


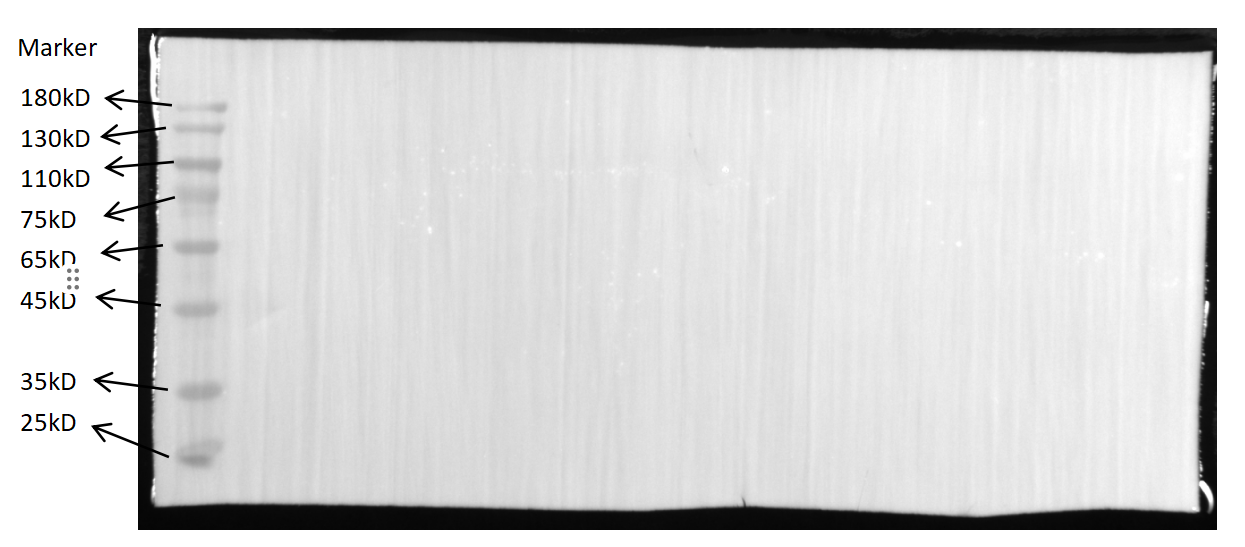


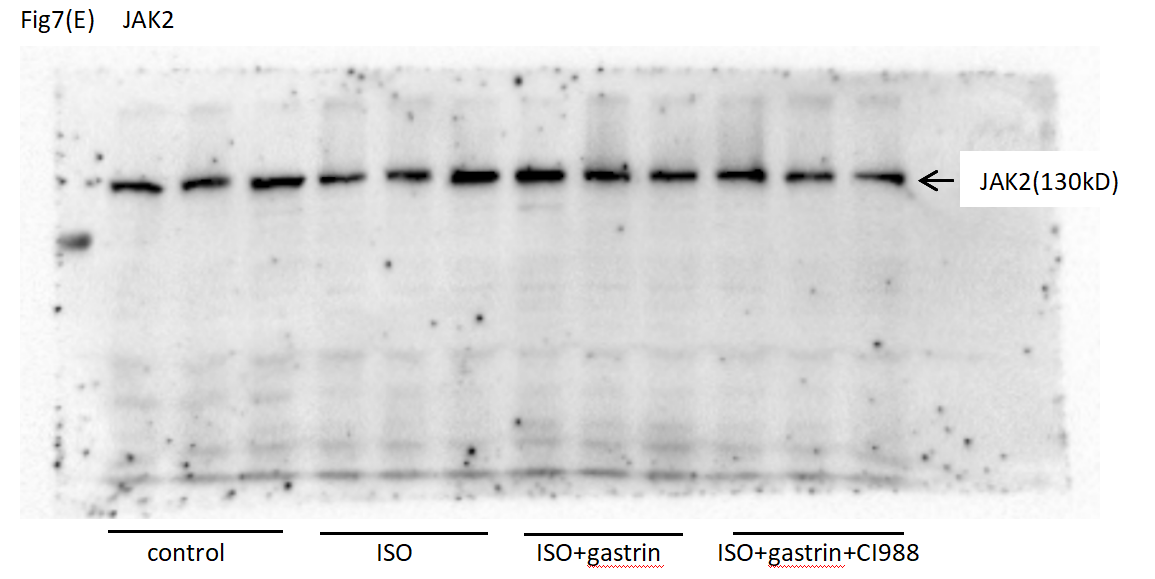


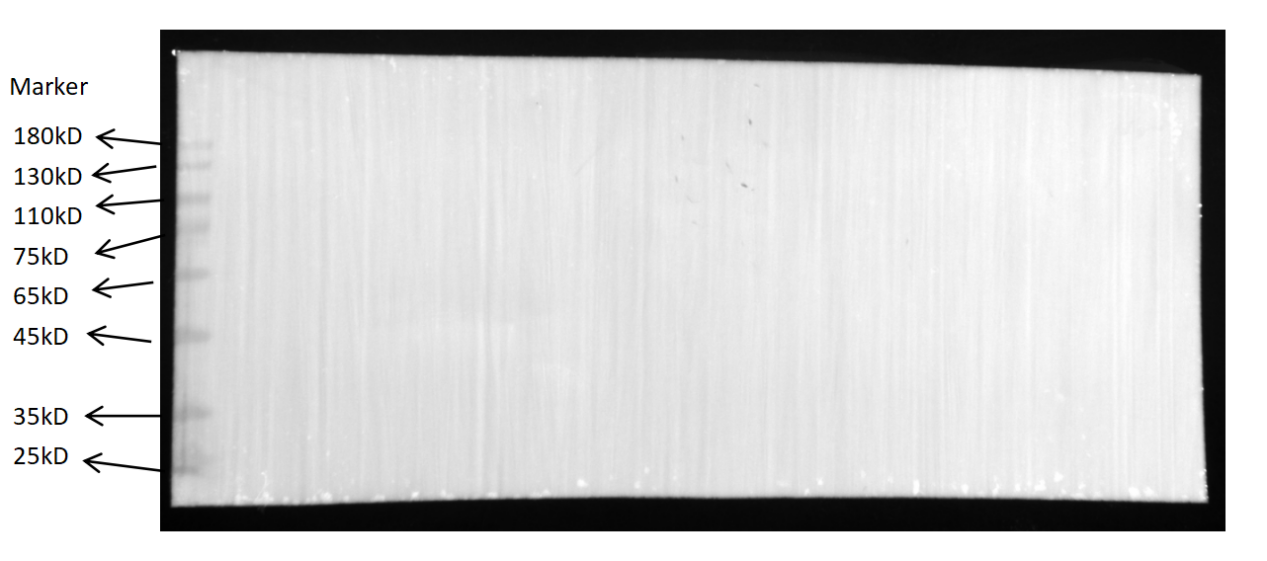


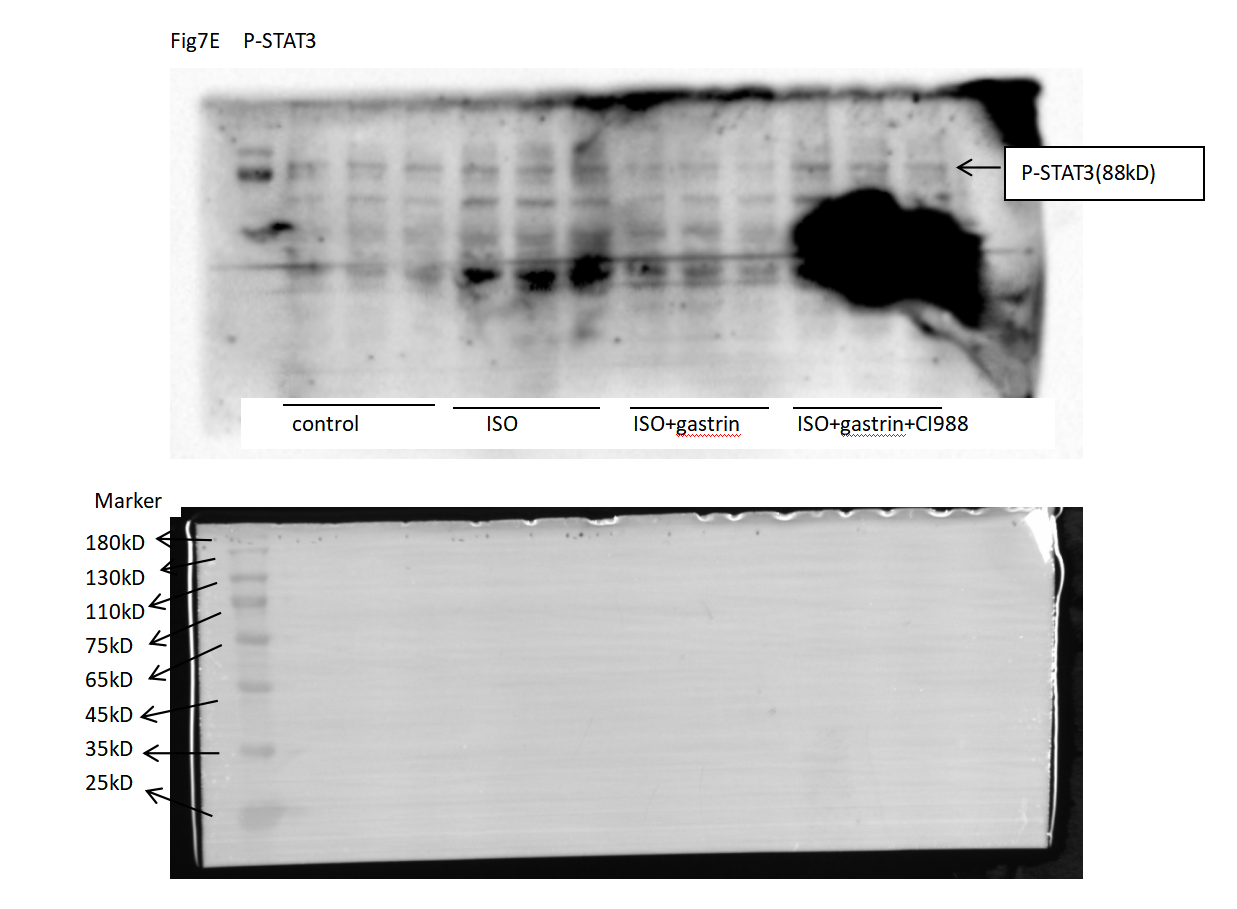


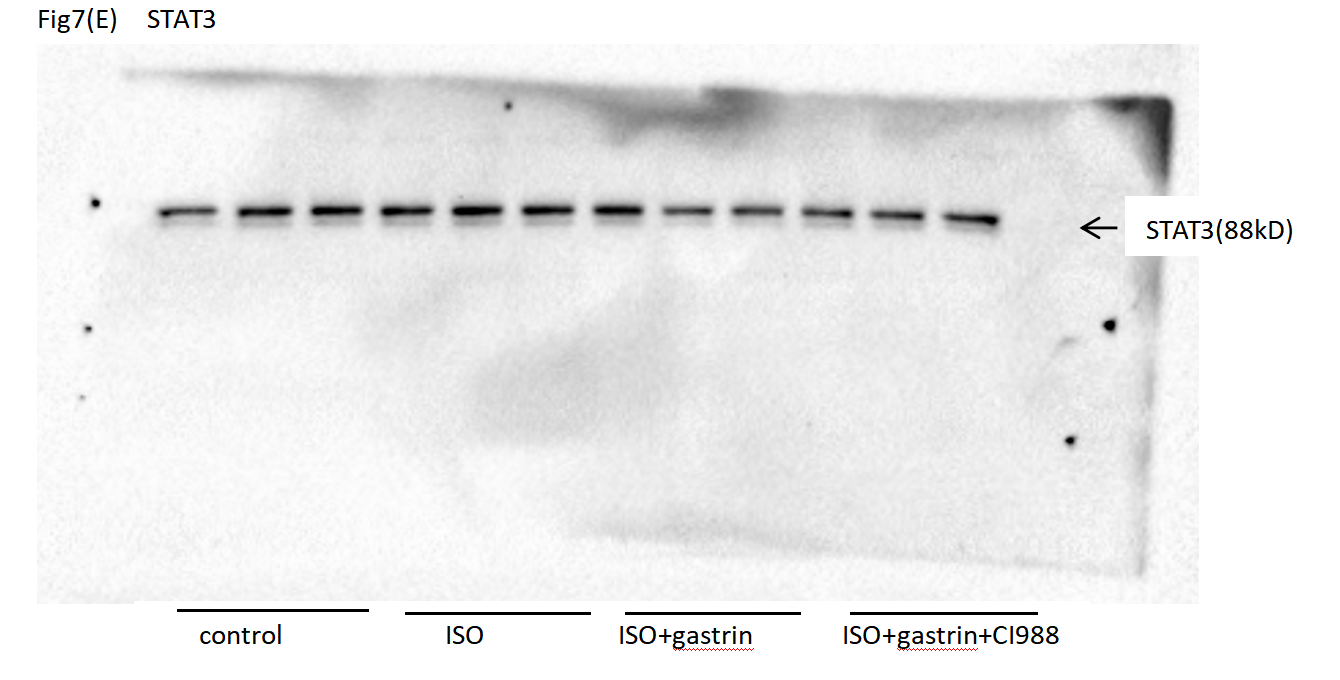


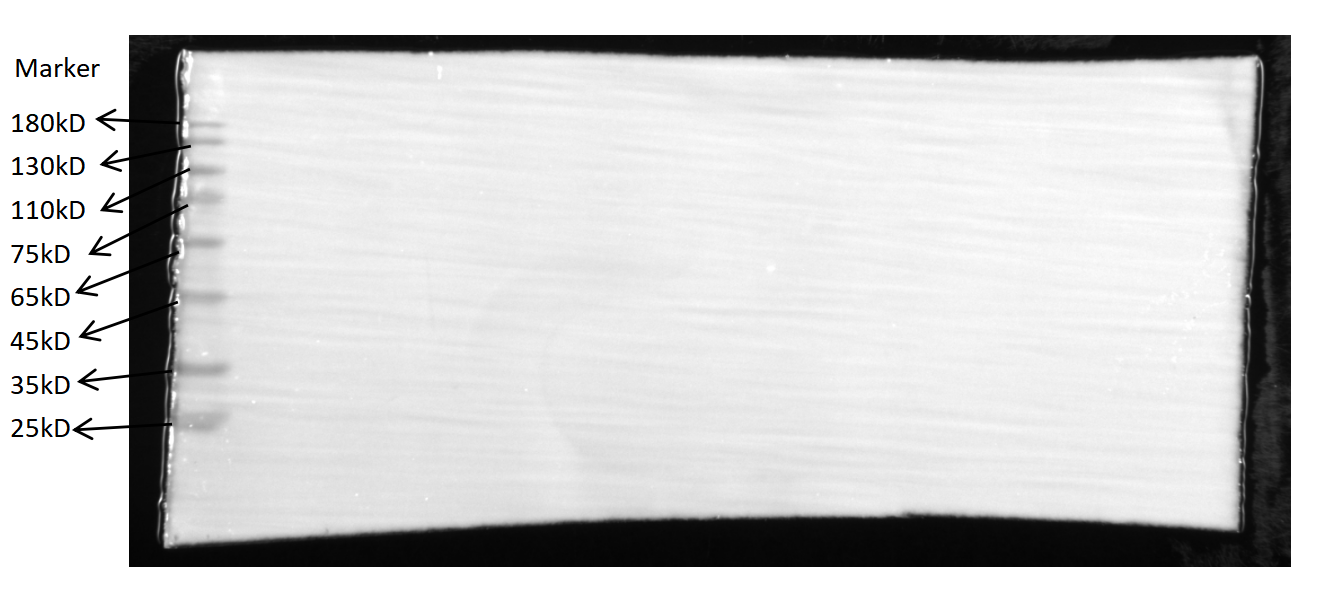


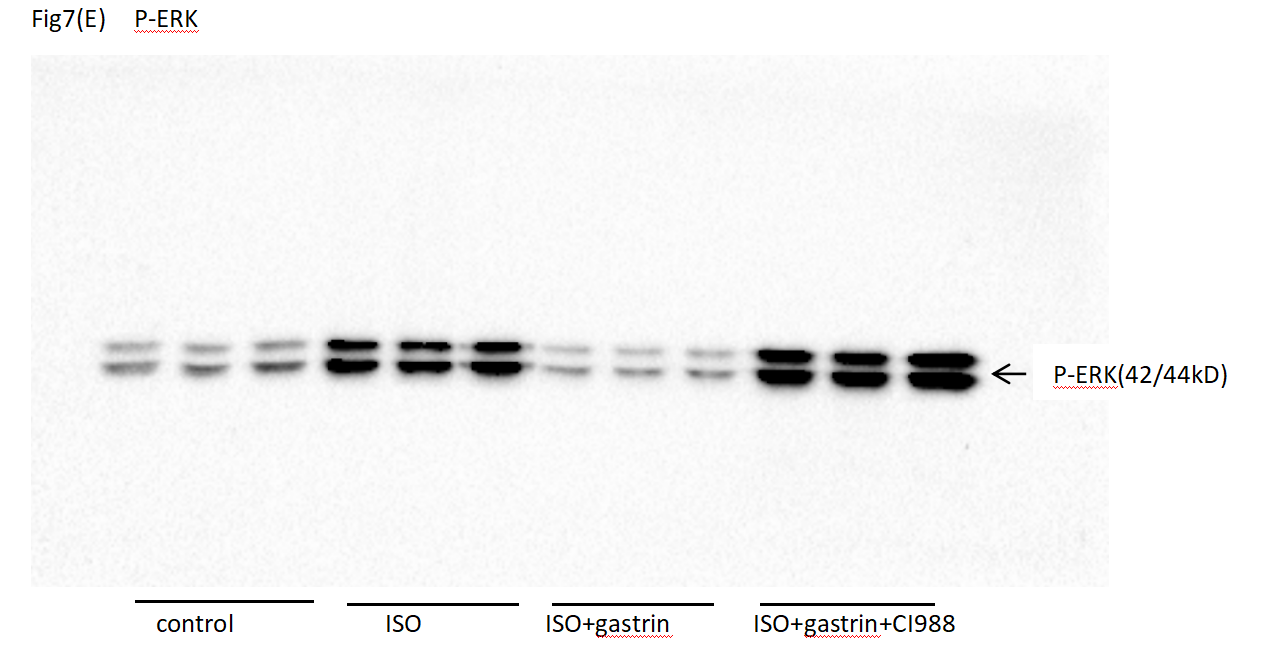


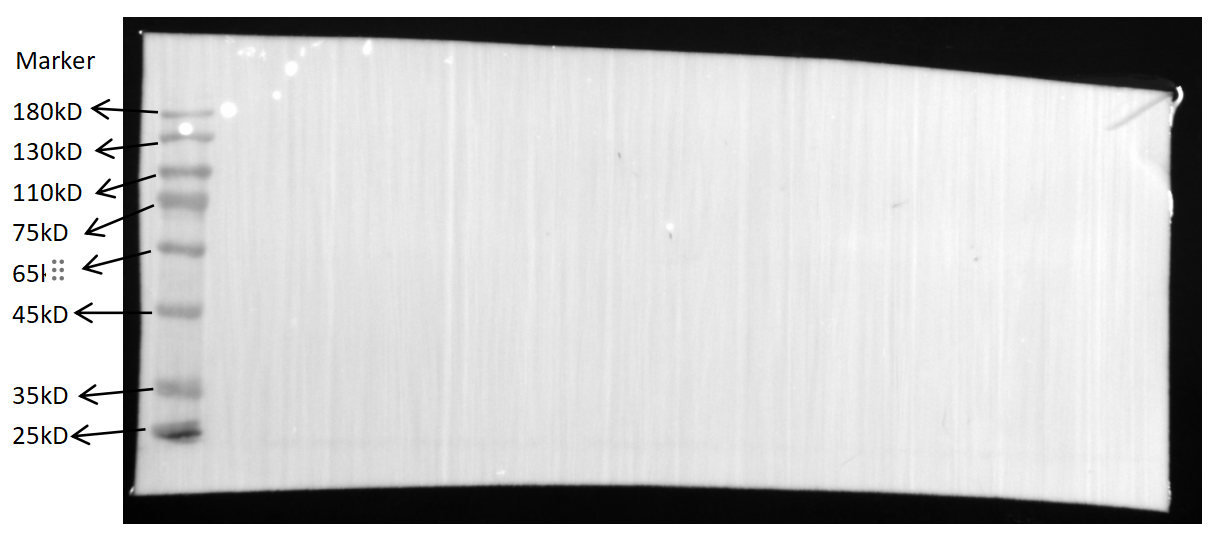


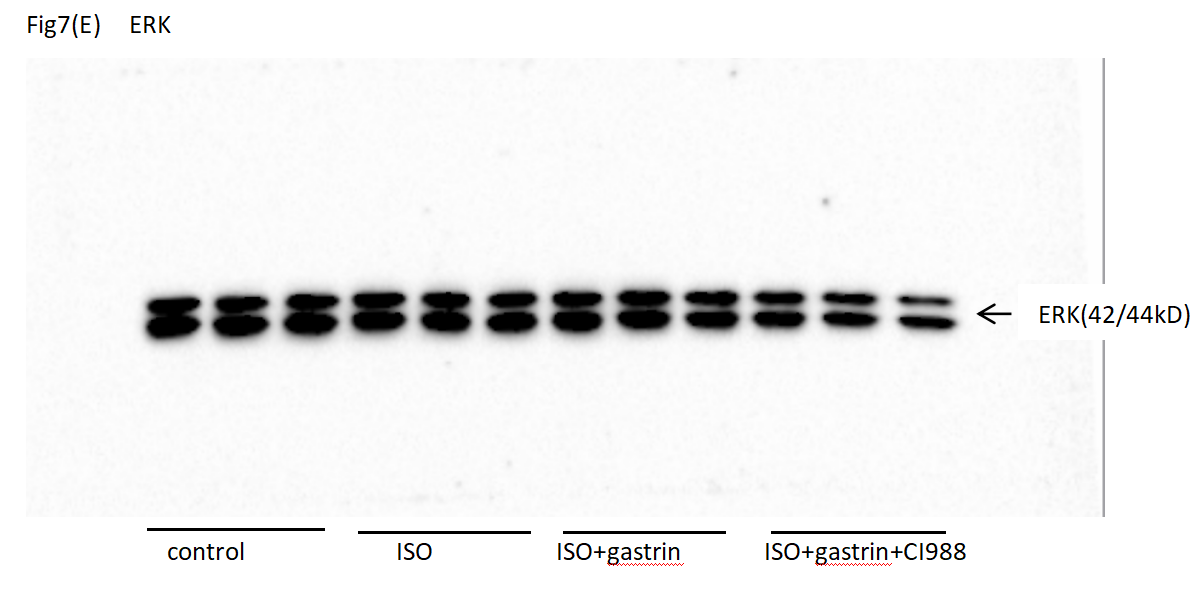


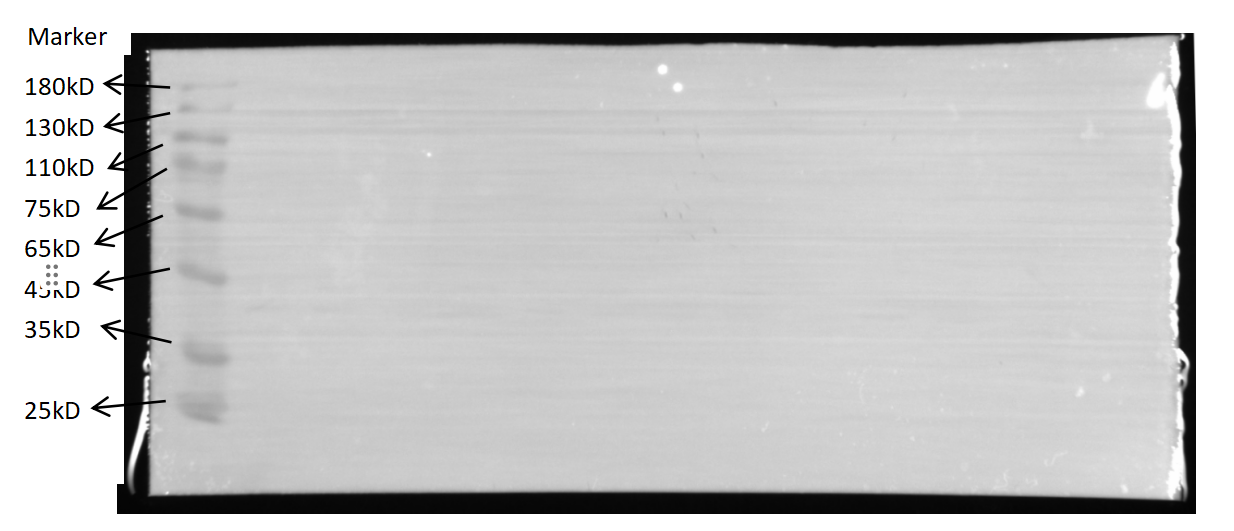


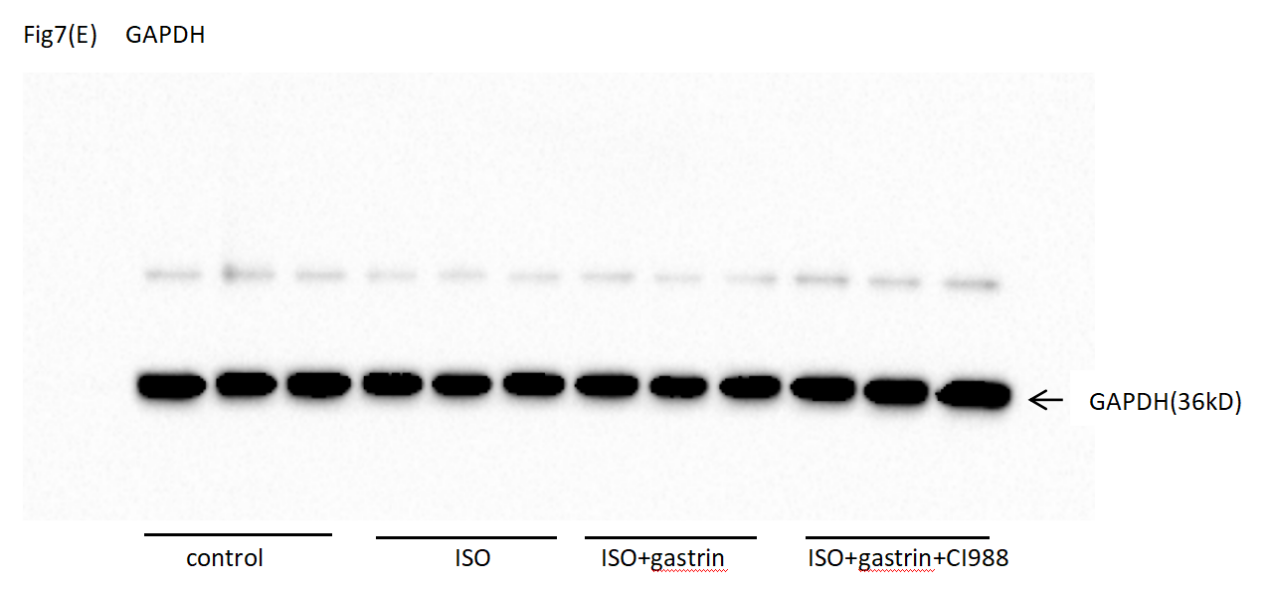


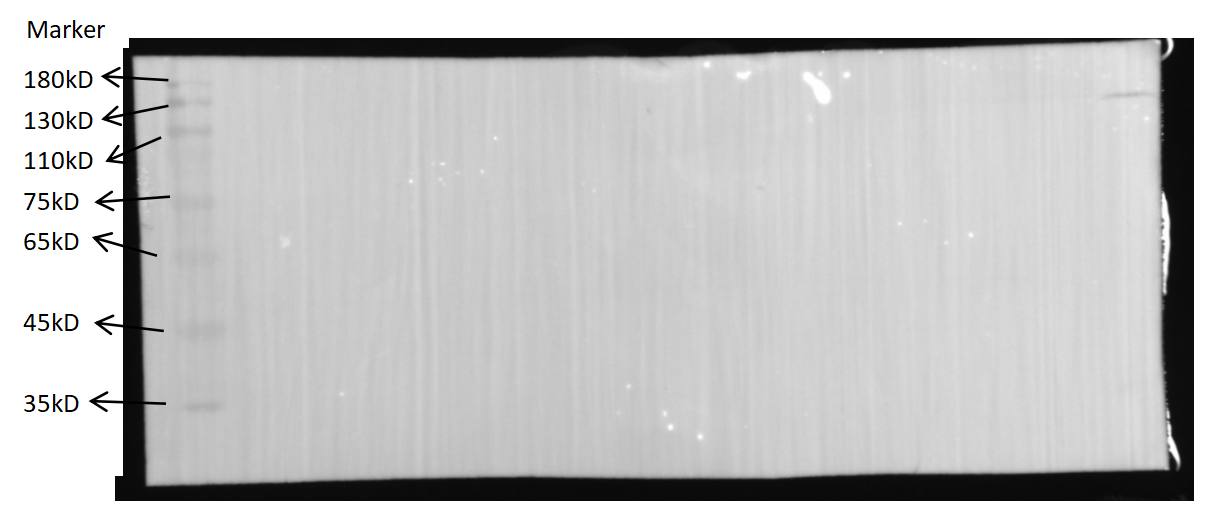


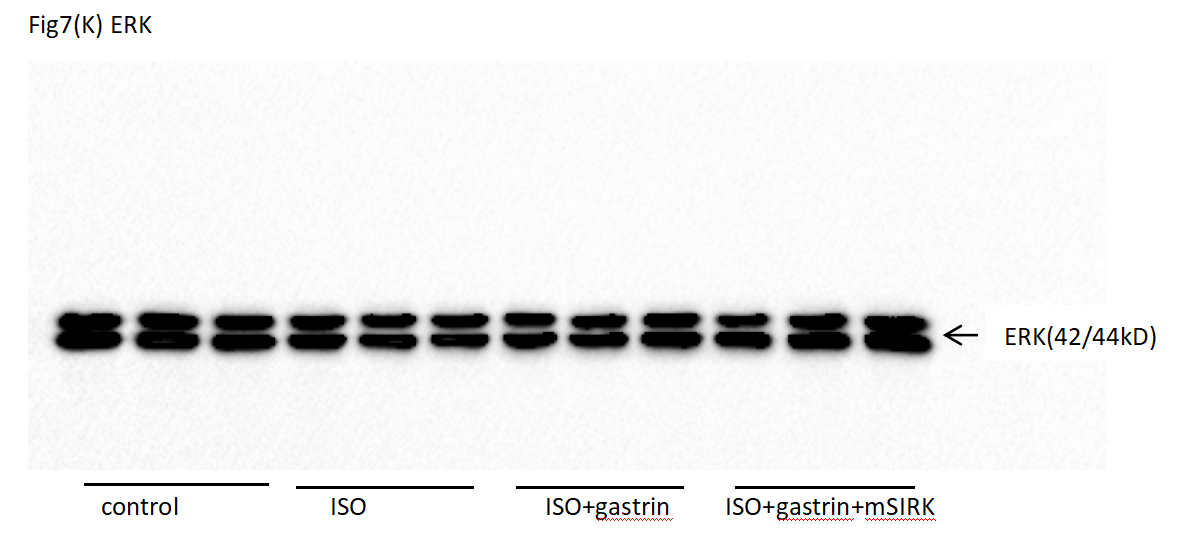


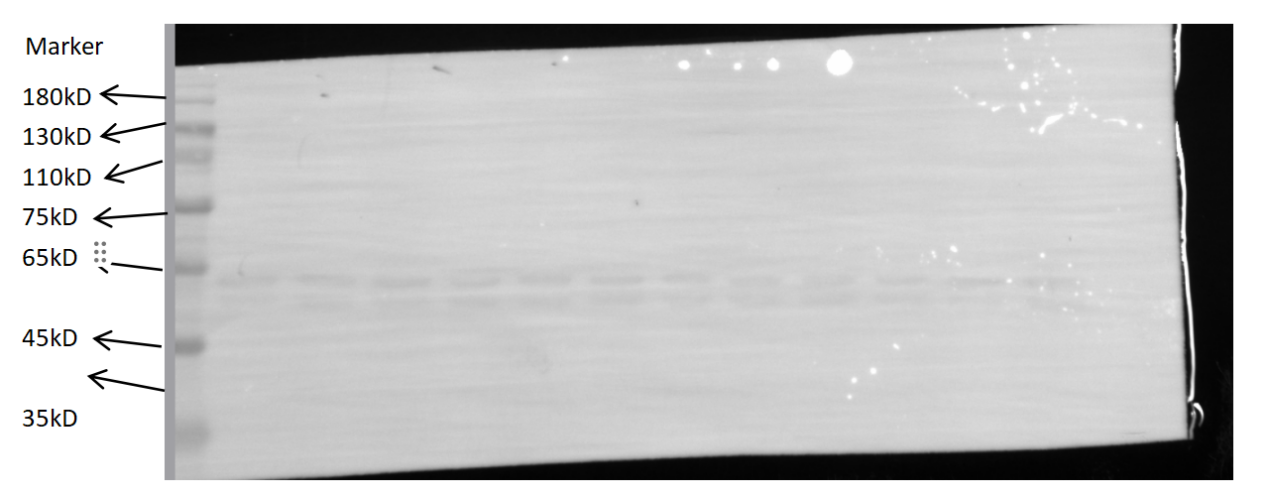


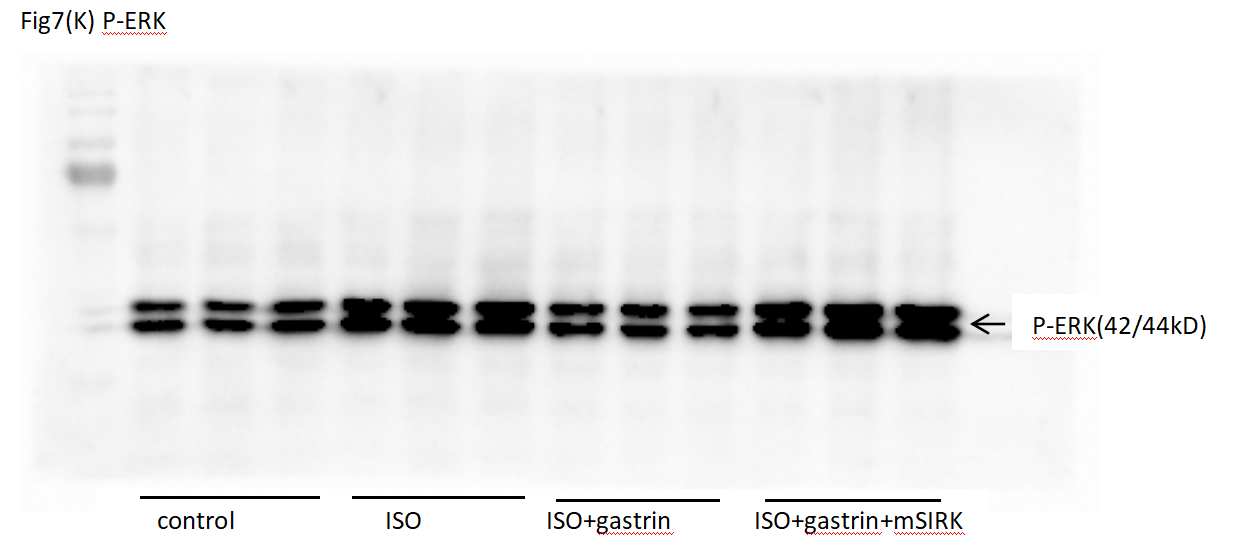


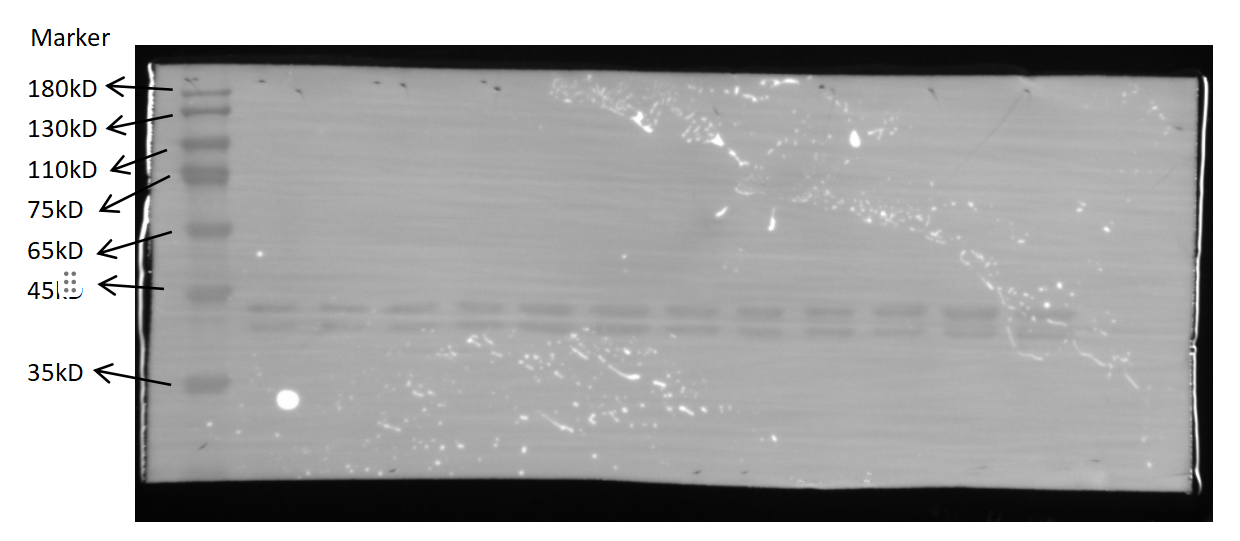


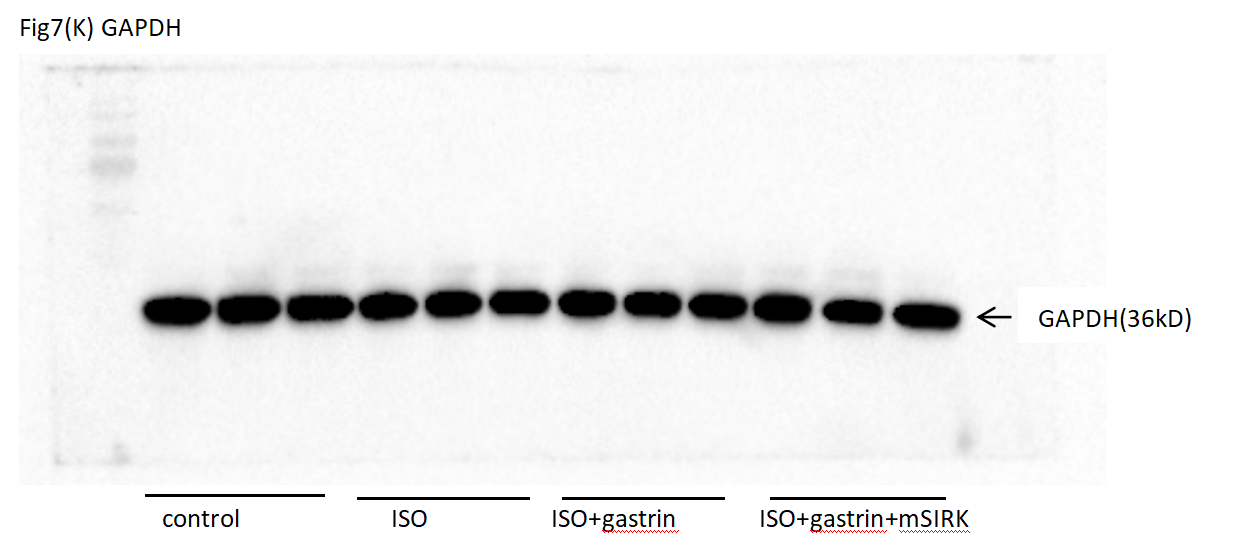


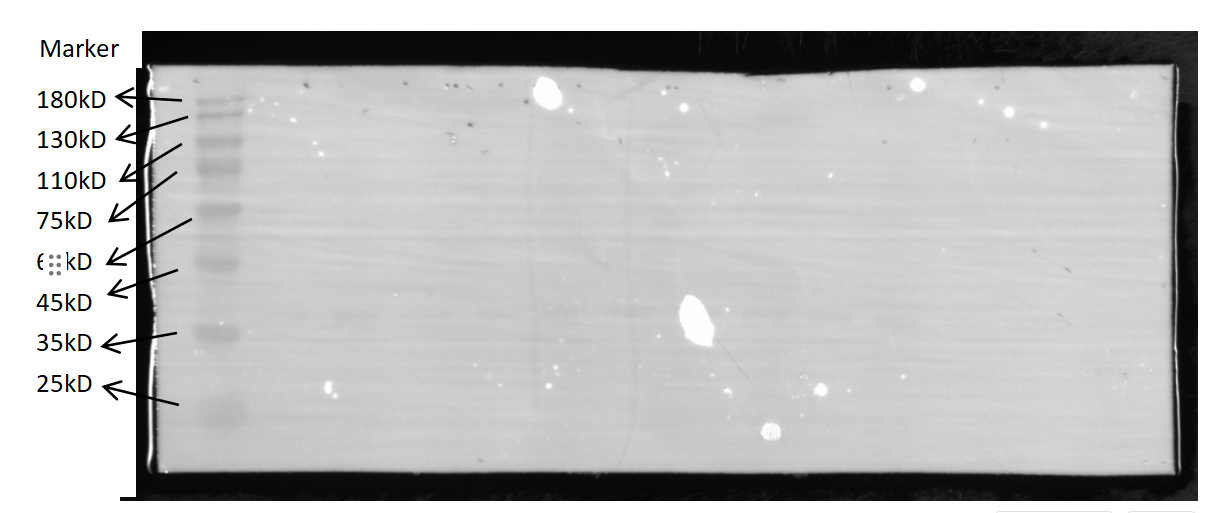


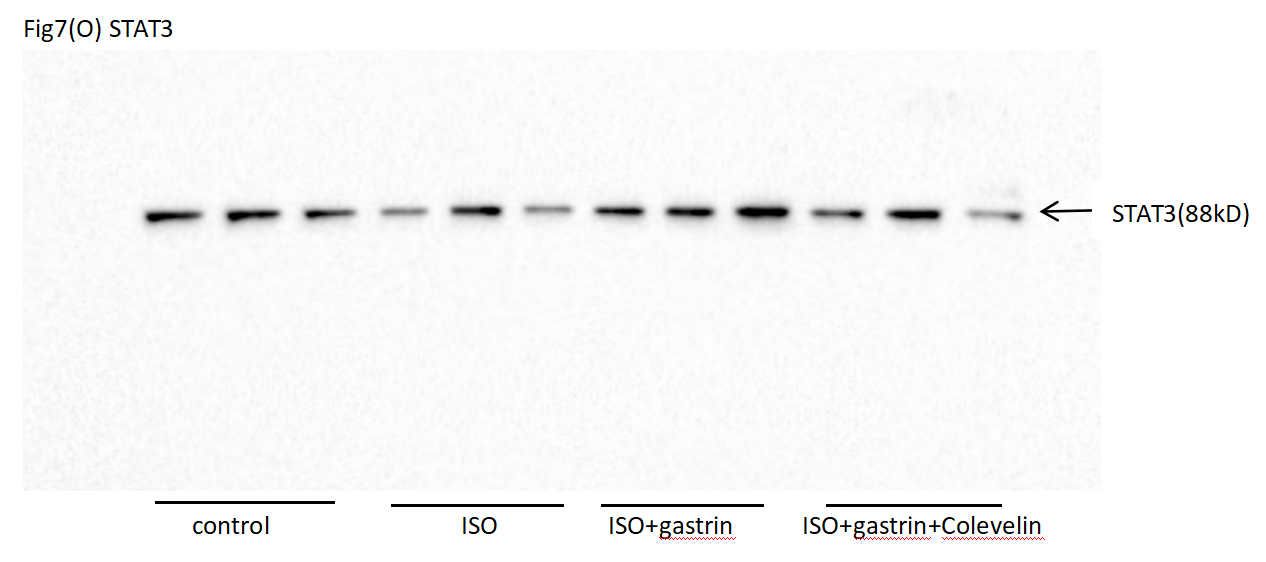


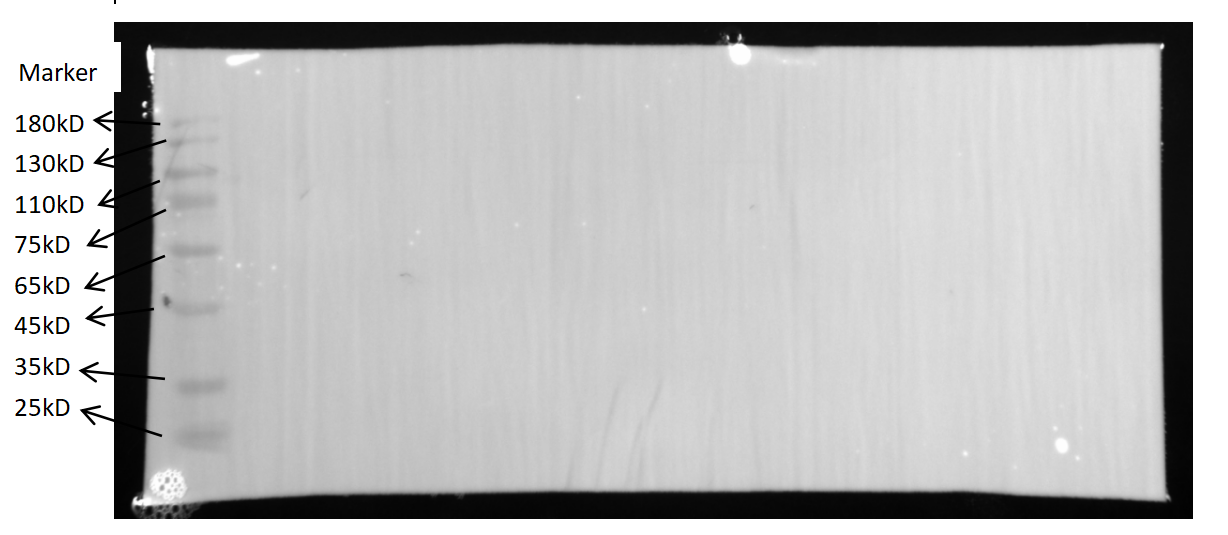


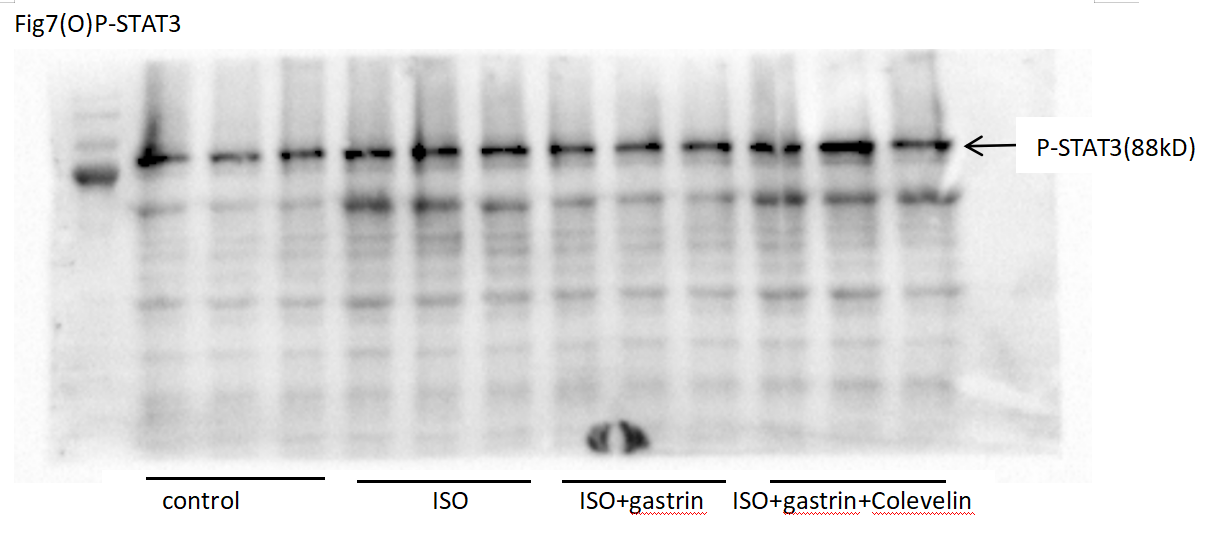


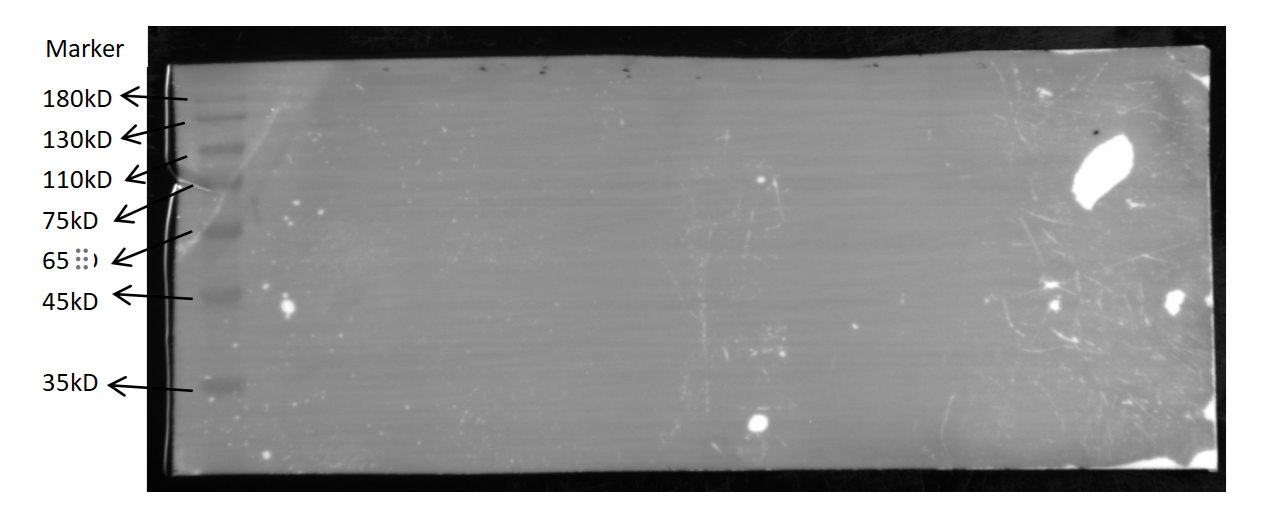


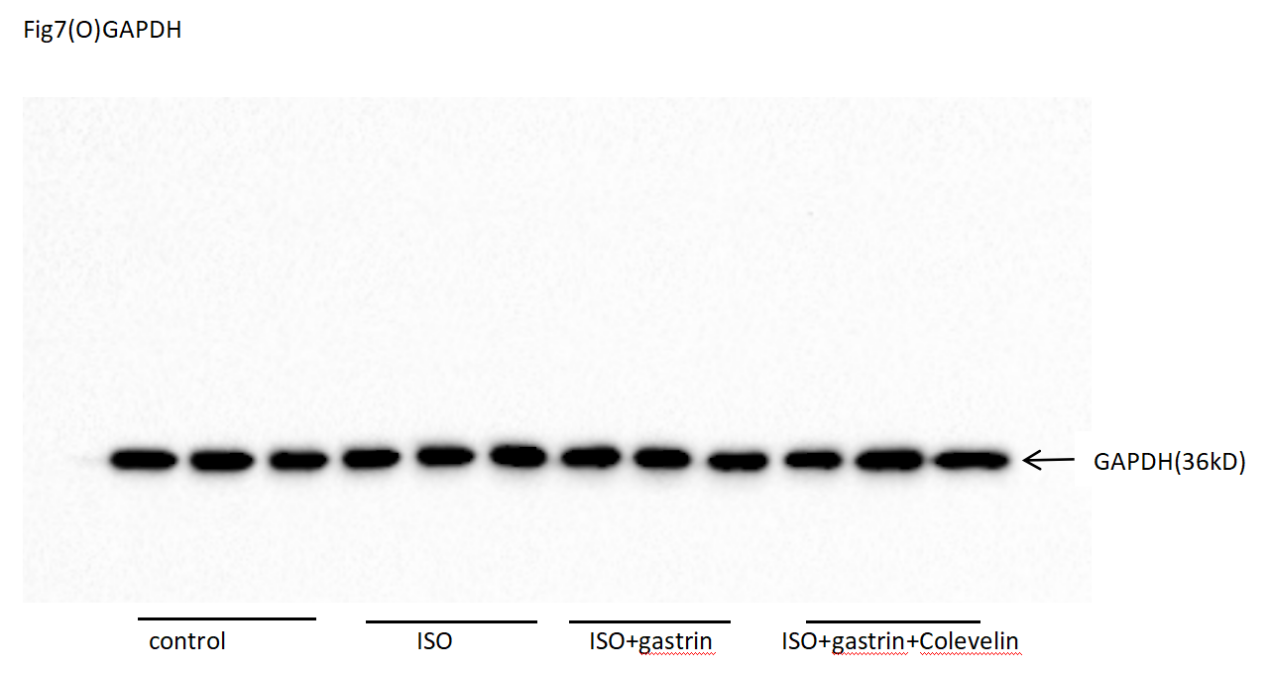


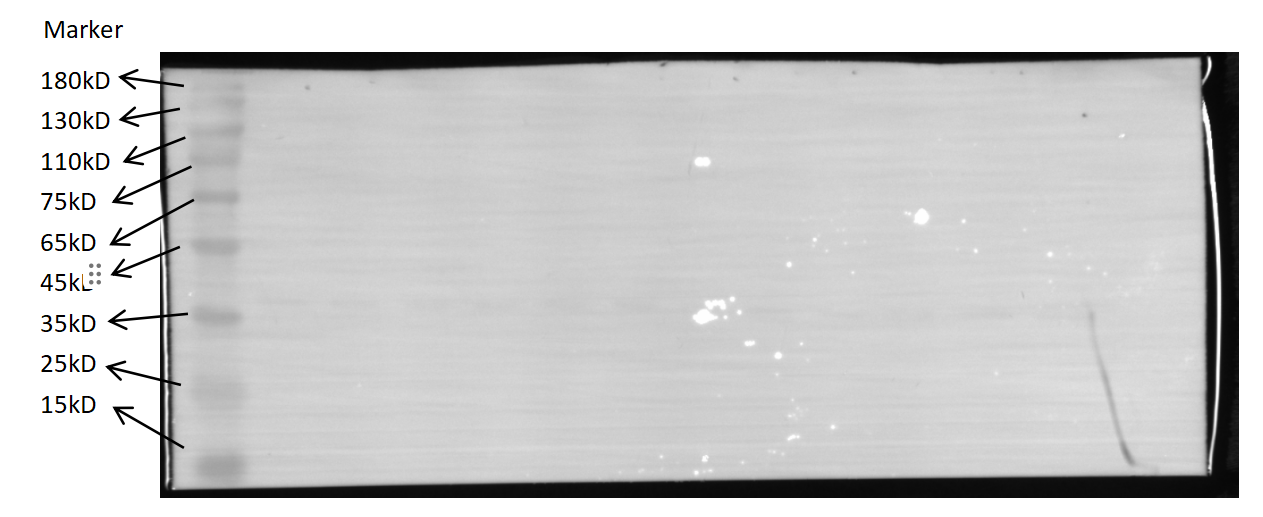


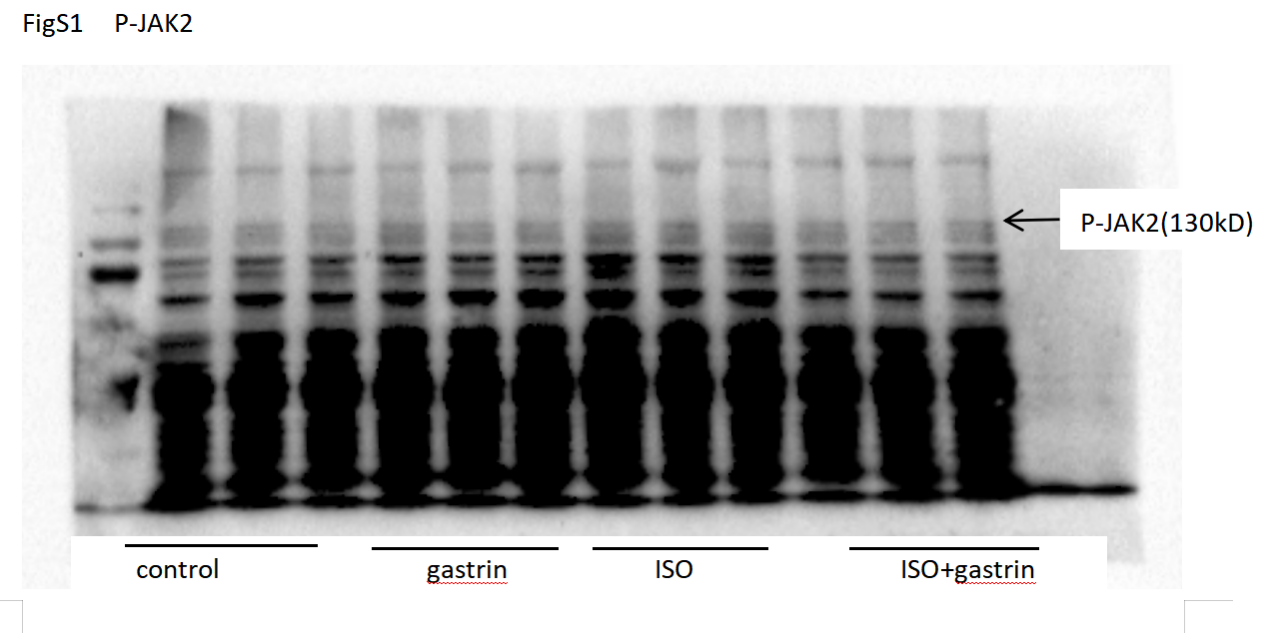


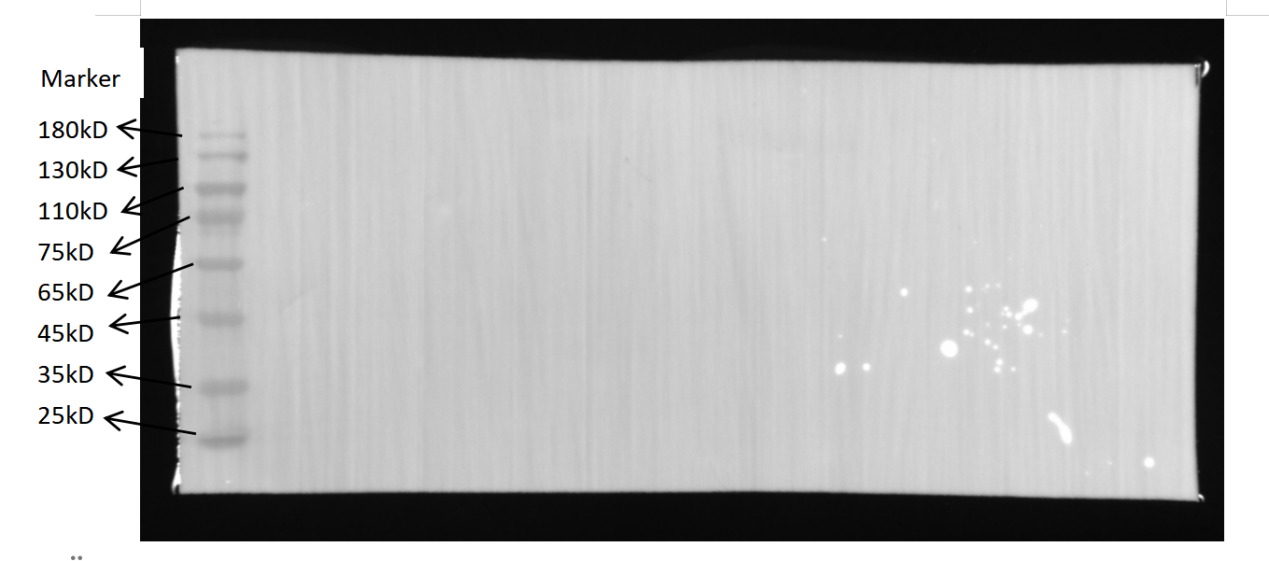


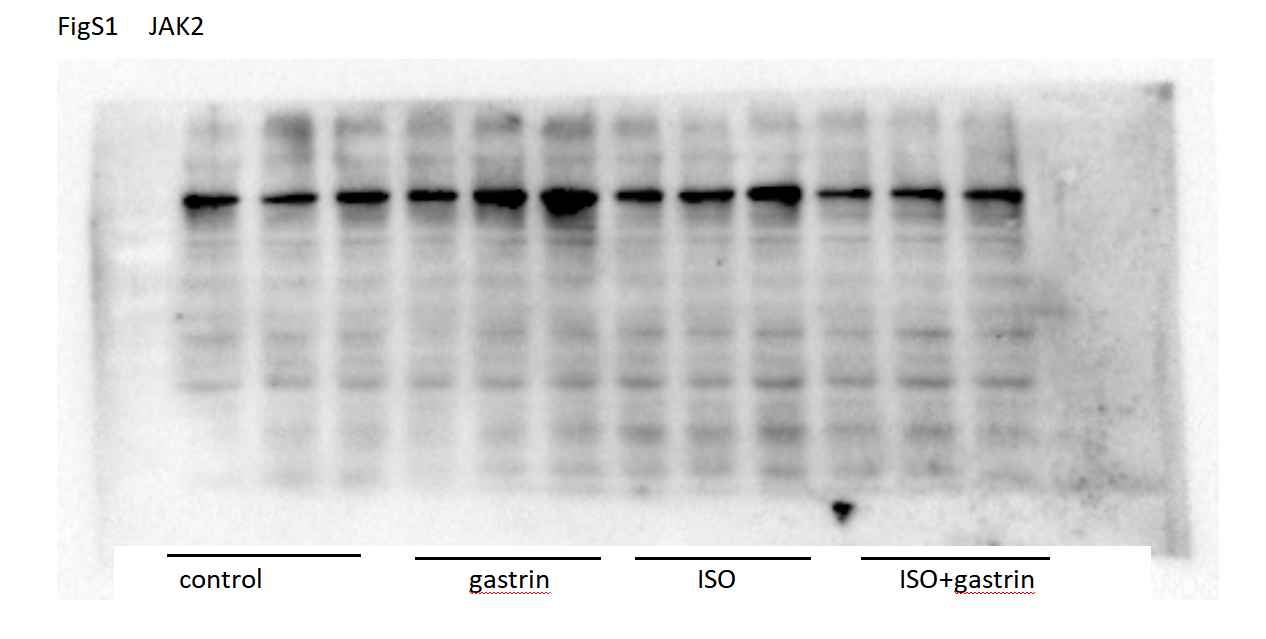


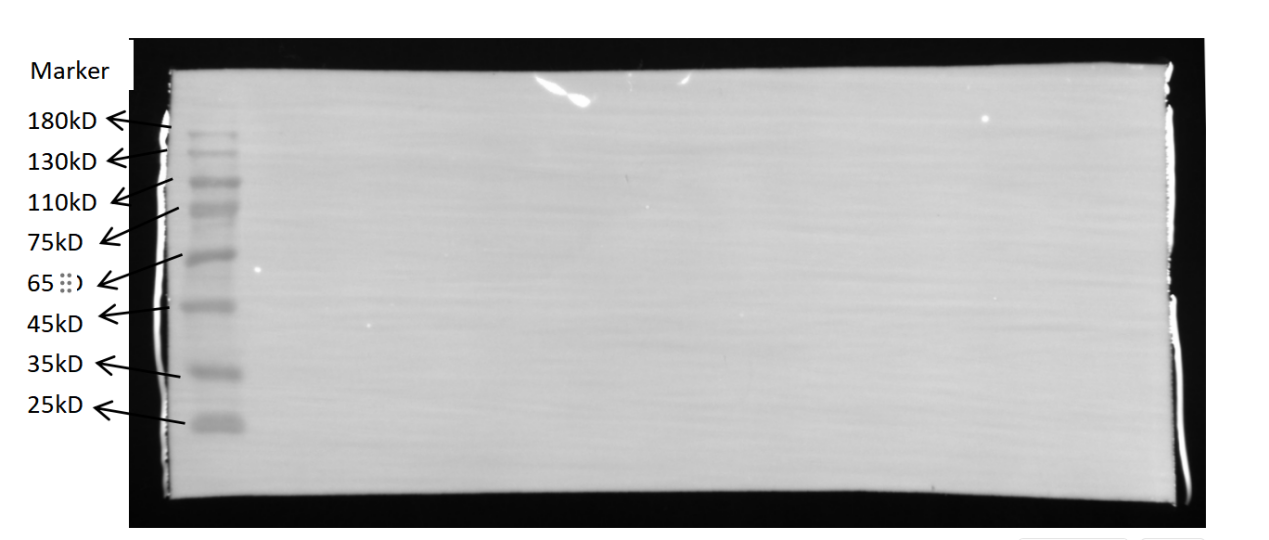


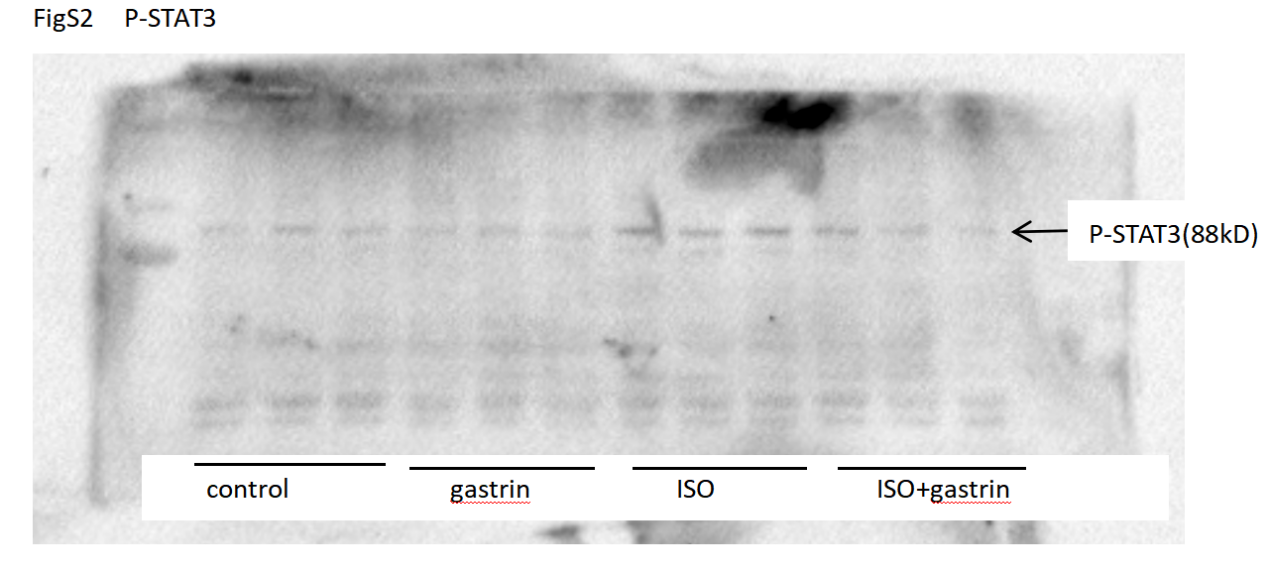


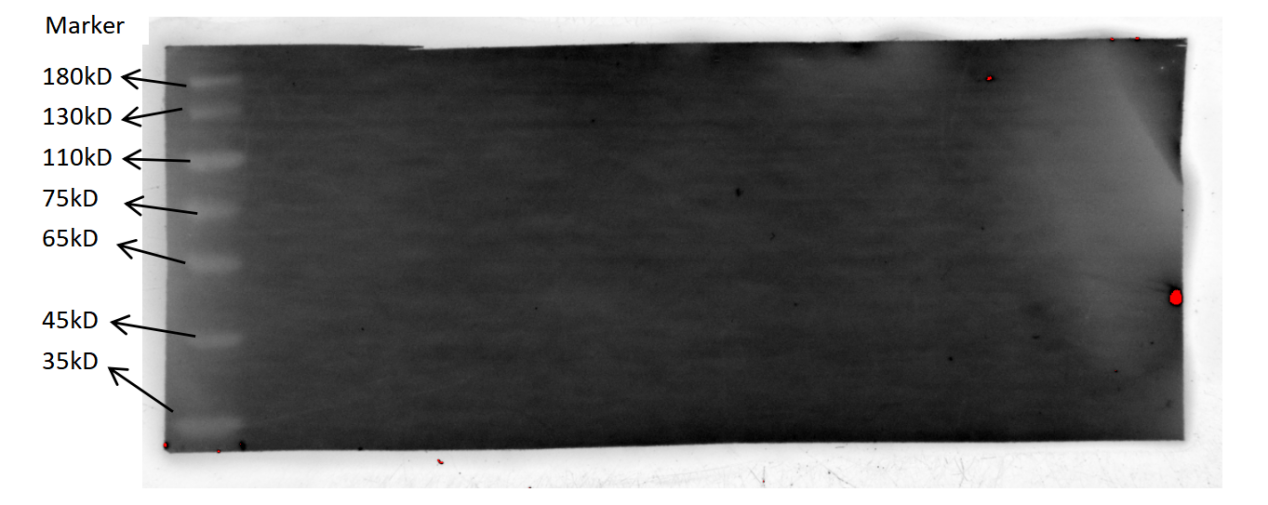


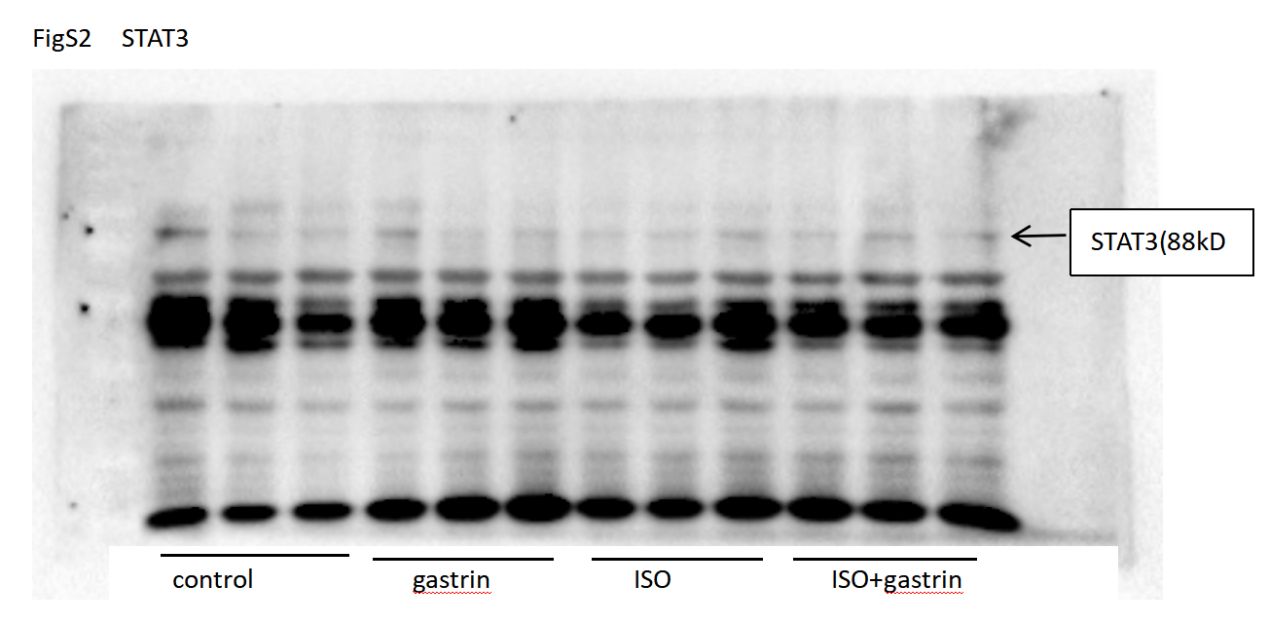


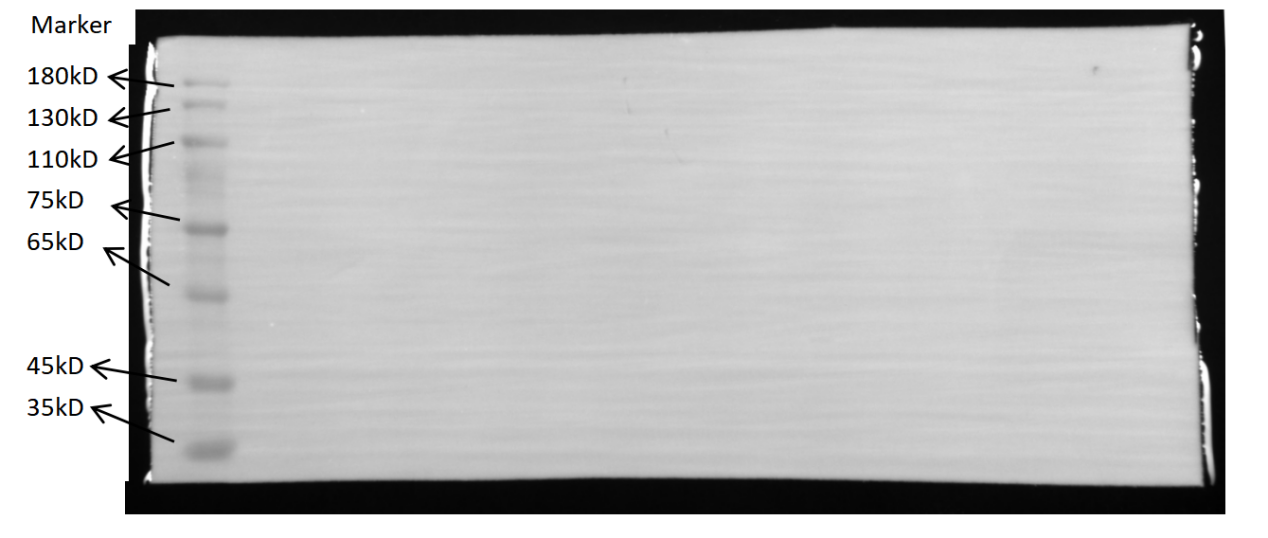


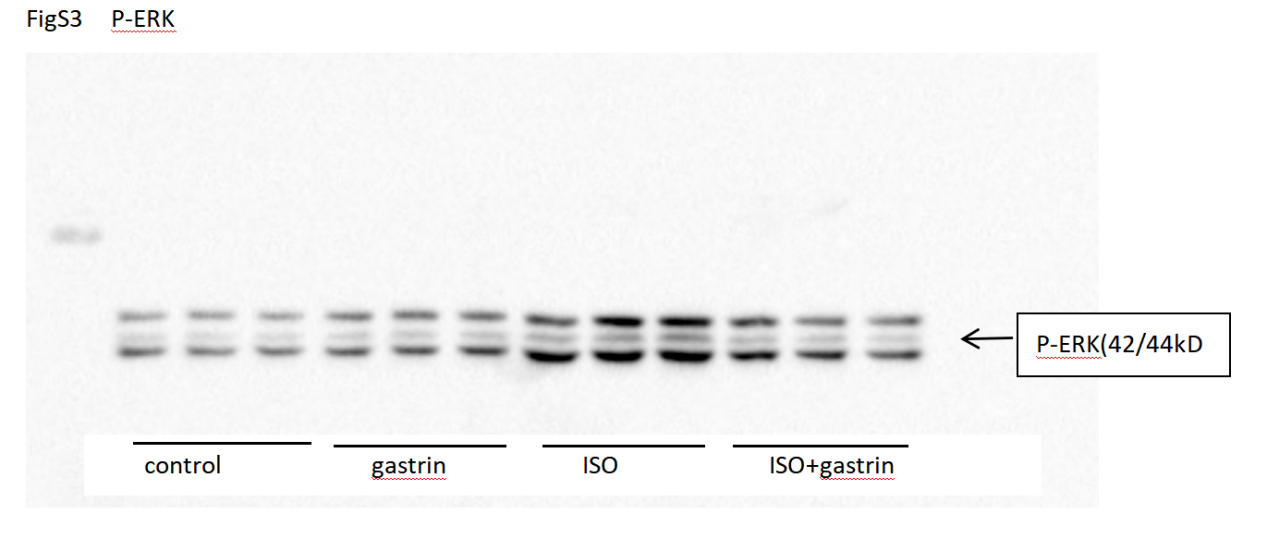


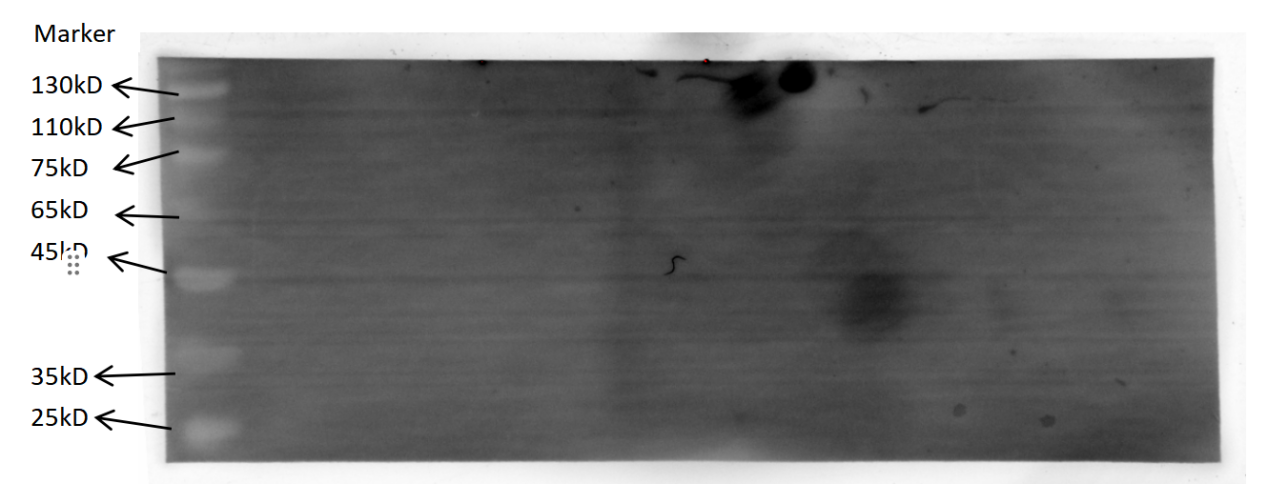


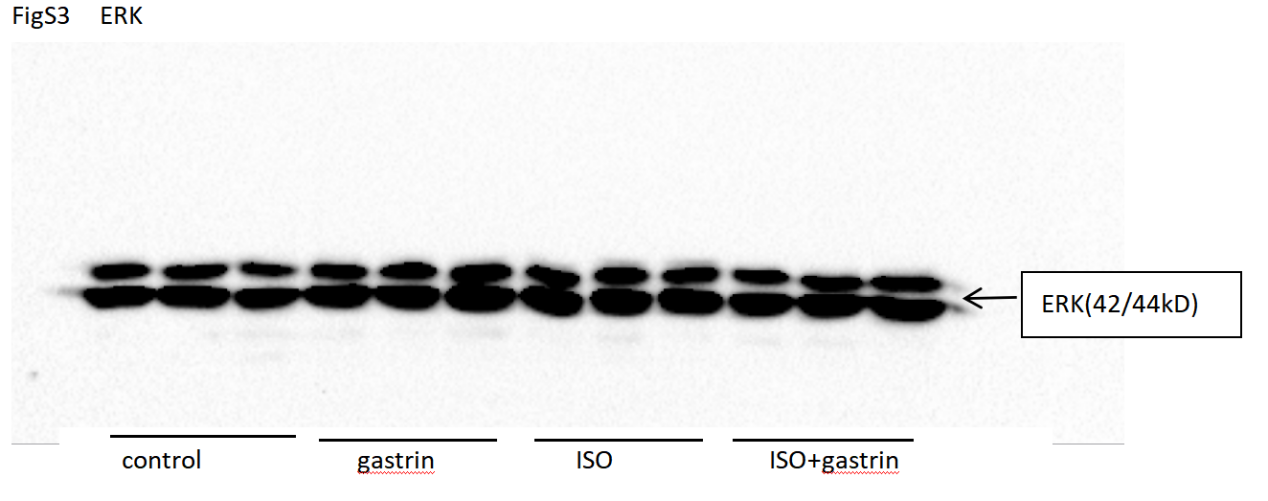


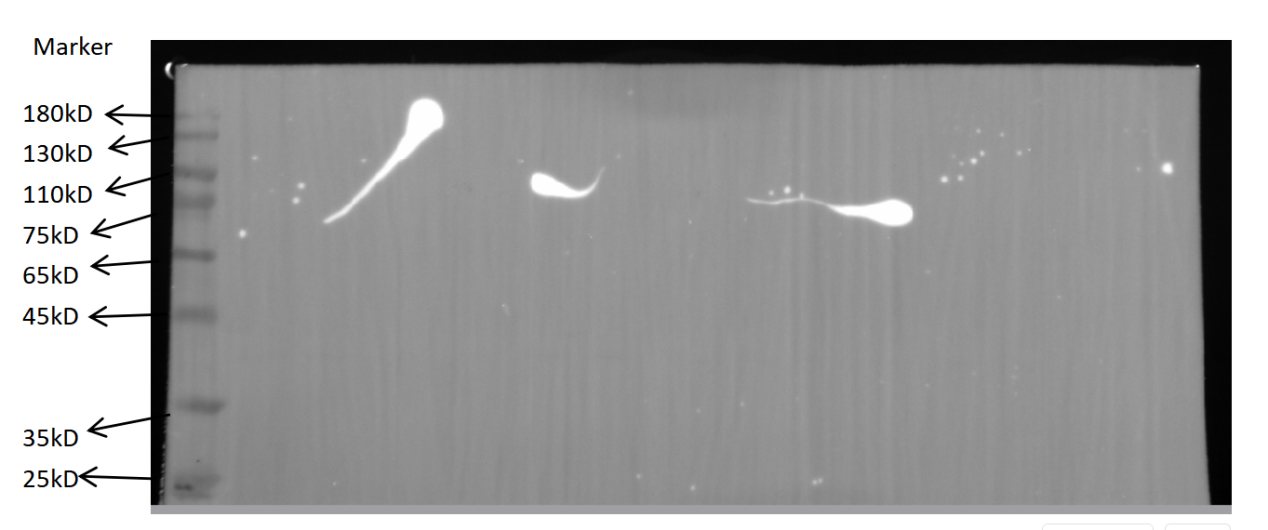


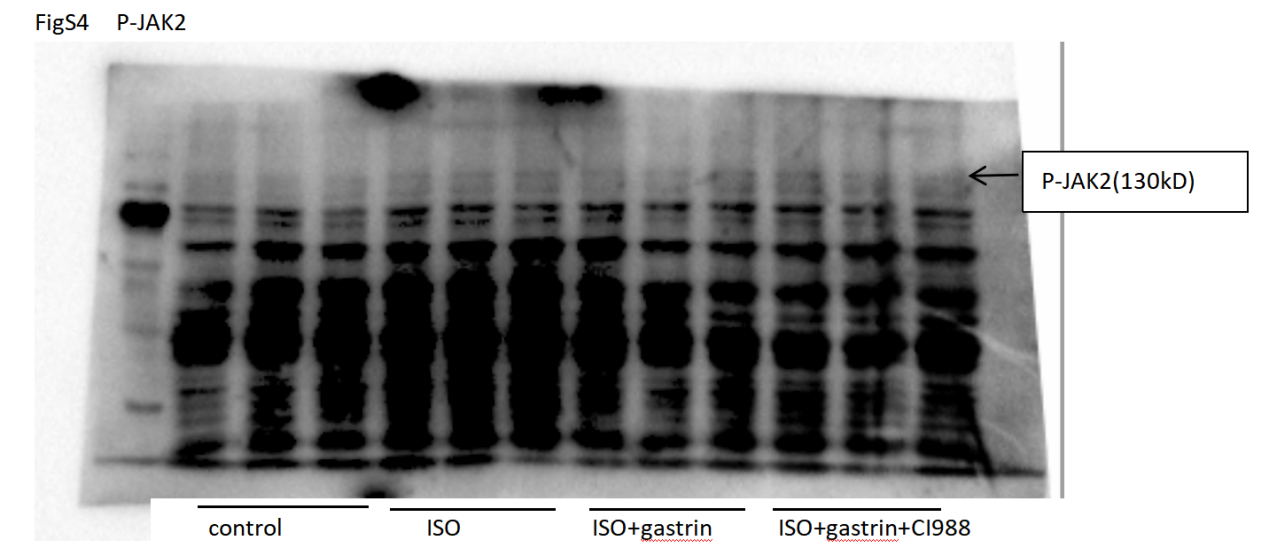


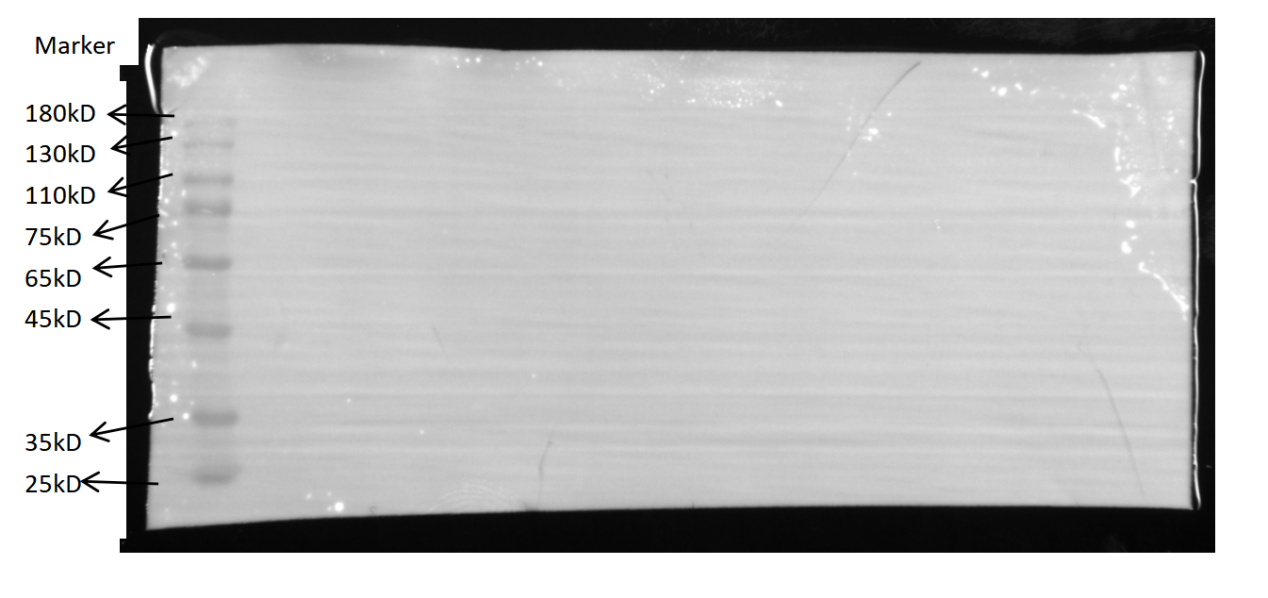


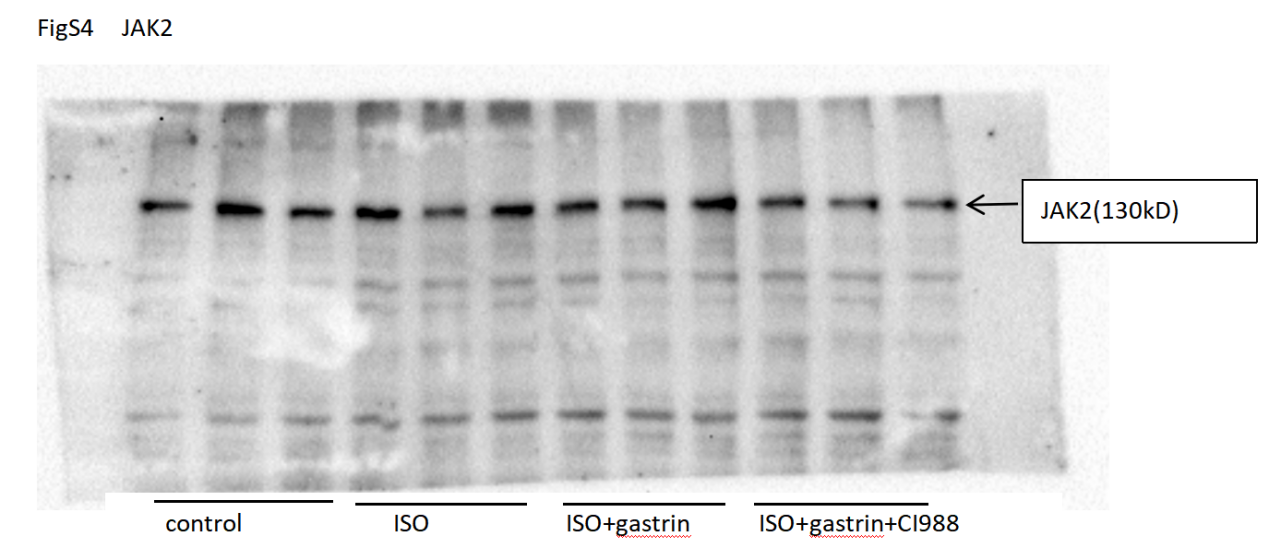


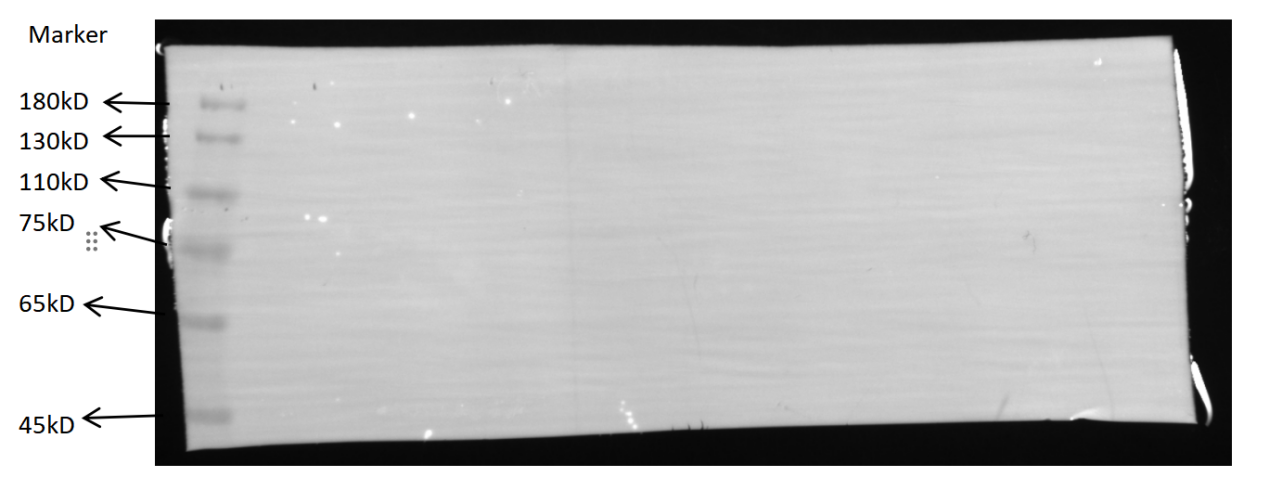


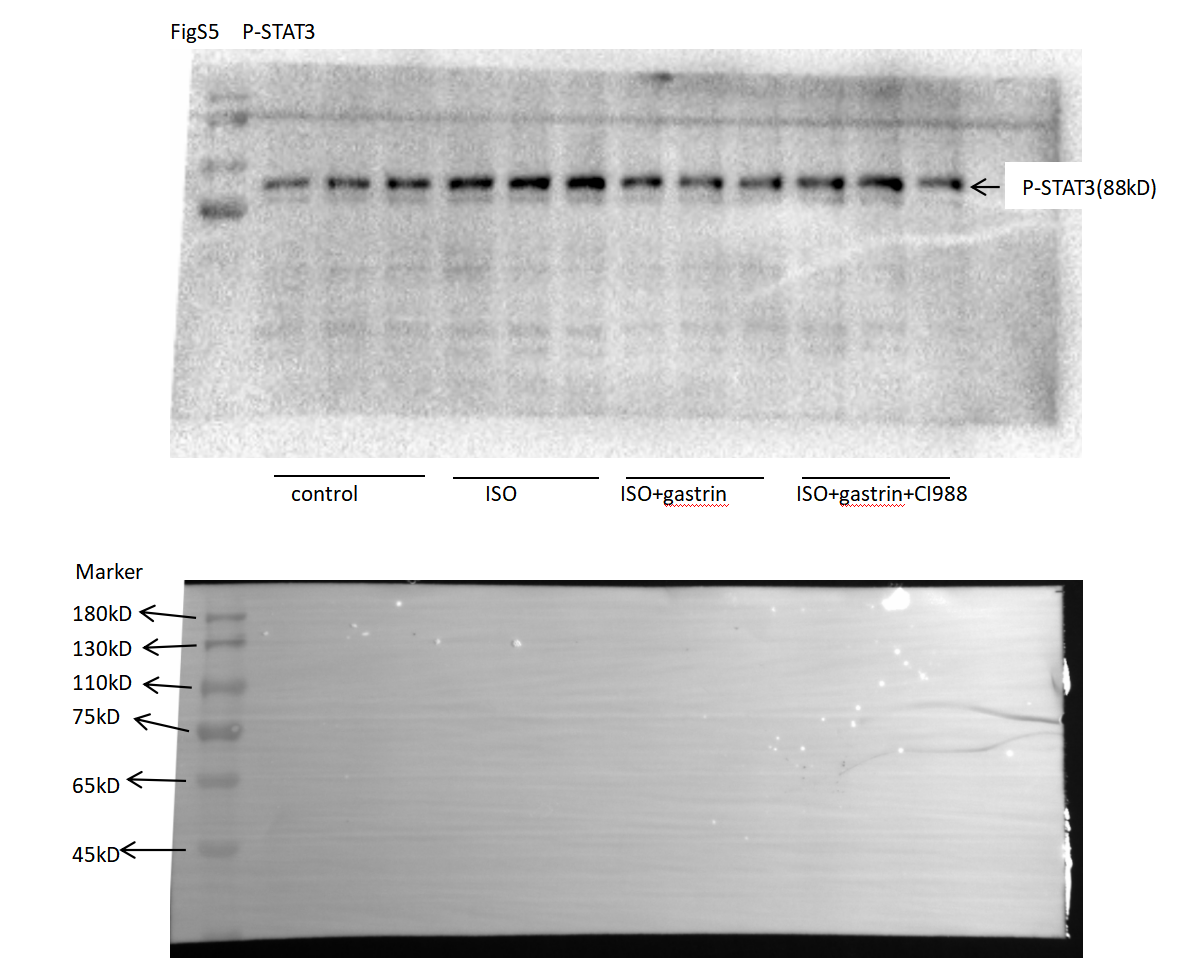


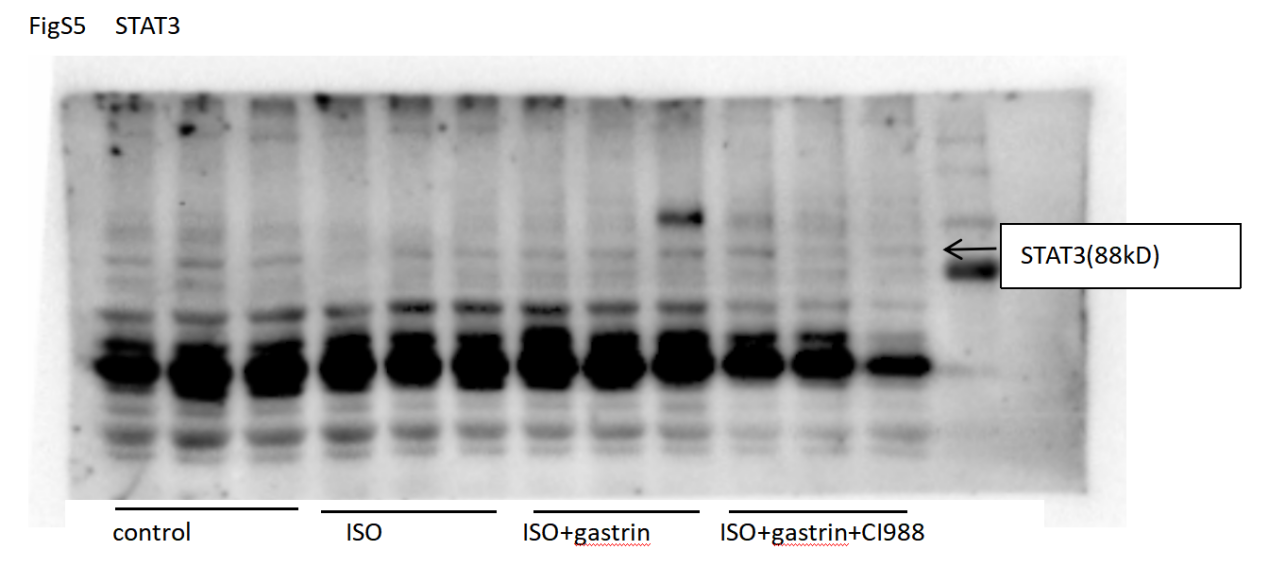


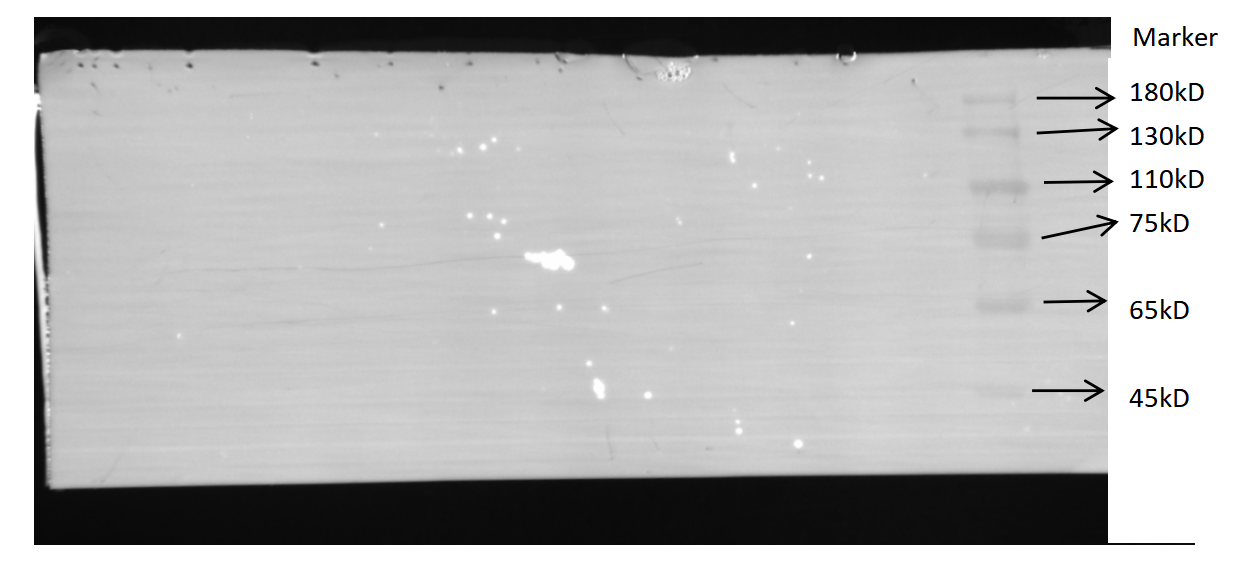


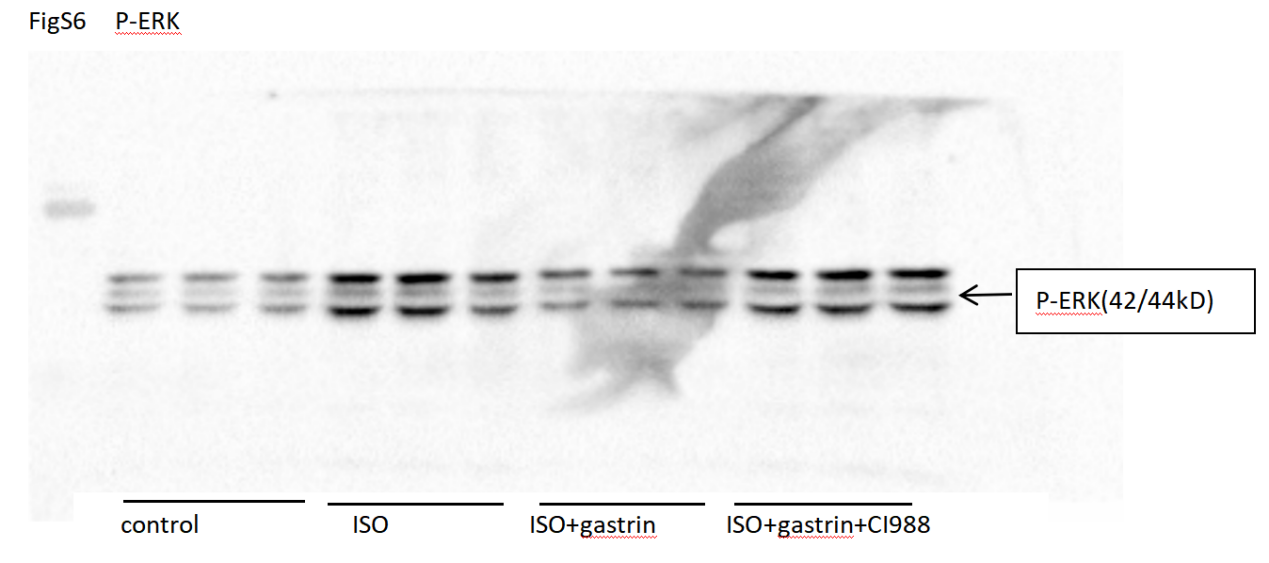


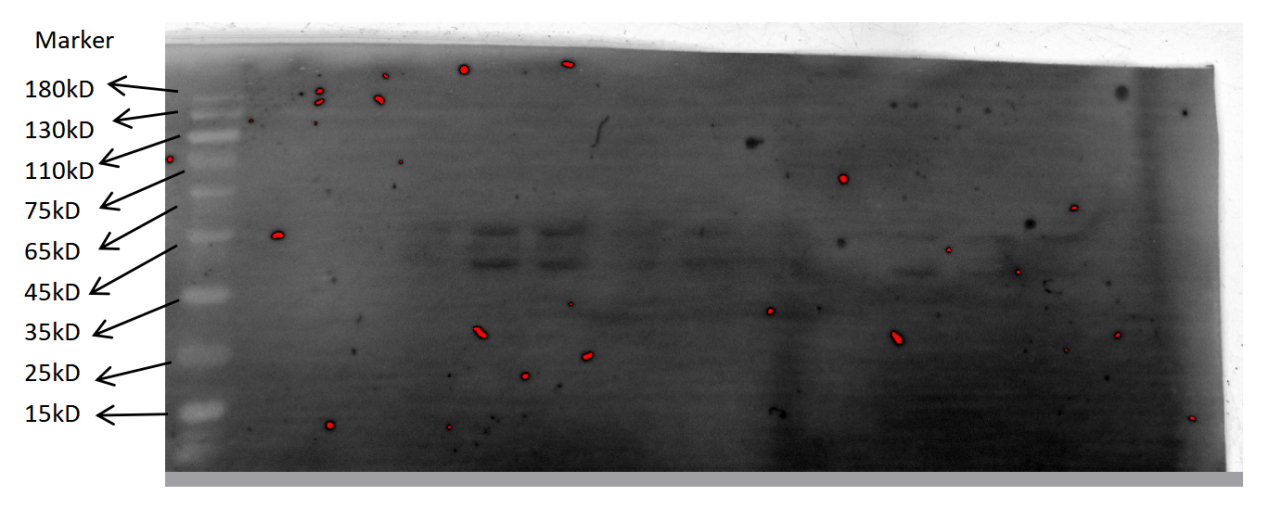


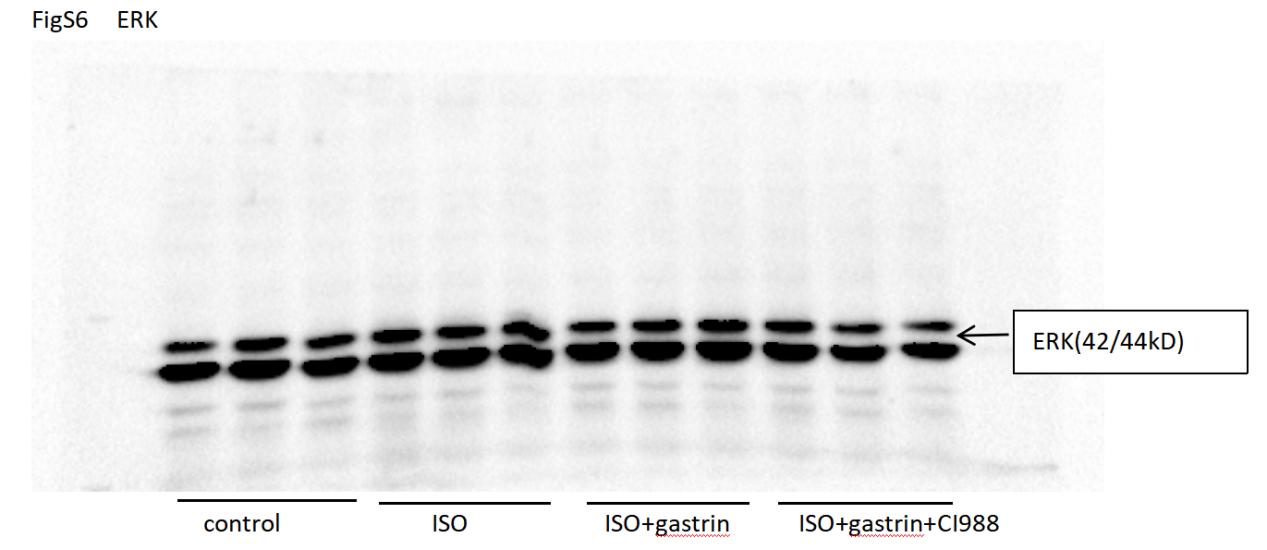


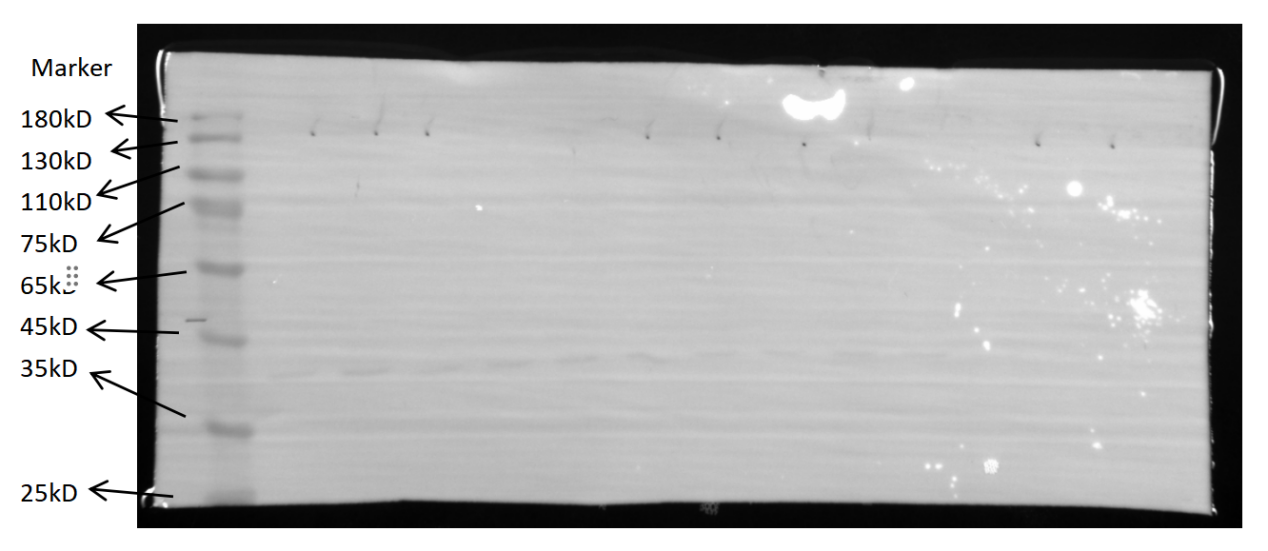

Supplement: S2 File — (DOCX) [file pone.0343403.s002.docx]
