# Supplementary material for: Gastrin ameliorates heart failure and suppresses myocardial remodeling via the JAK2/STAT3 and ERK1/2 pathways
Source: PLoS One. 2026 Mar 5;21(3):e0343403. doi: 10.1371/journal.pone.0343403 (PMC12962455; doi:10.1371/journal.pone.0343403)
Supplement: Attachment — Submitted filename: Prezado Editor PLOS ONE.pdf [file pone.0343403.s004.pdf]

Prezado Editor

Dear Editor,

First, I would like to apologize for not submitting my considerations by the first established deadline. Unfortunately, I had personal issues that prevented me from initially reviewing them critically, so I requested a longer review.

The manuscript is well-prepared, with scientific and methodological criteria and sufficient results to answer the proposed questions.

The references are adequate.

As I am not a native English speaker, I did not make any suggestions regarding spelling and agreement.

I suggest accepting the manuscript.

Yours sincerely

Dra. Natália Maria Maciel Guerra Silva
