# Supplementary material for: Gastrin ameliorates heart failure and suppresses myocardial remodeling via the JAK2/STAT3 and ERK1/2 pathways
Source: PLoS One. 2026 Mar 5;21(3):e0343403. doi: 10.1371/journal.pone.0343403 (PMC12962455; doi:10.1371/journal.pone.0343403)
Supplement: Attachment — Submitted filename: Response to Reviewers.docx [file pone.0343403.s005.docx]

**Response to editor:**

**Question1:**Please ensure that your manuscript meets PLOS ONE's style requirements, including those for file naming.

**Response:**The manuscript has been reformatted in accordance with the journal's submission guidelines.

**Question2:**Please confirm at this time whether or not your submission contains all raw data required to replicate the results of your study.

**Response:**All original data associated with the article have been compiled into the files titled "S1 file" and "Supplement to supporting Information" and have been submitted to the journal alongside this revised manuscript.

**Question3:**PLOS ONE now requires that authors provide the original uncropped and unadjusted images underlying all blot or gel results reported in a submission’s figures or Supporting Information files.

**Response:**All gel images presented in the manuscript have been saved in the file "S1_raw_images" in accordance with the journal's requirements.

**Question4:**When completing the data availability statement of the submission form, you indicated that you will make your data available on acceptance.

**Response:**I hereby acknowledge and accept the journal's data disclosure policy, which permits unrestricted and free access to all associated data.

**Question5:**PLOS requires an ORCID iD for the corresponding author in Editorial Manager on papers submitted after December 6th, 2016. Please ensure that you have an ORCID iD and that it is validated in Editorial Manager.

**Response:**The ORCID iD of the corresponding author BaoLi: 0009-0006 -4449-7743.

The ORCID record of the corresponding author BaoLi is <https://orcid.org/> 0009-0006-4449-7743.

**Question6:**Please include your full ethics statement in the ‘Methods’ section of your manuscript file. In your statement, please include the full name of the IRB or ethics committee who approved or waived your study, as well as whether or not you obtained informed written or verbal consent. If consent was waived for your study, please include this information in your statement as well.

**Response:**The information regarding the ethics committee and the corresponding ethics approval number has been included in the "Methods" section. As follows:

This study received ethical approval from the Ethics Committee of Shanxi Provincial Cardiovascular Hospital(registration number: 2023kjt100; date:2023.12.1).(Line 179-181,Page 9)

**Question7:**Please remove your figures from within your manuscript file, leaving only the individual TIFF/EPS image files, uploaded separately. These will be automatically included in the reviewers’ PDF.

**Response:**The figures in the manuscript have been uploaded separately in accordance with the journal's requirements, and the figure captions have been incorporated into the manuscript.

**Question8:**If the reviewer comments include a recommendation to cite specific previously published works, please review and evaluate these publications to determine whether they are relevant and should be cited. There is no requirement to cite these works unless the editor has indicated otherwise. 
**Response:**We gratefully acknowledge the editor for ensuring a fair and rigorous review process. We also appreciate the reviewers’ impartiality, as they did not request the inclusion of citations to their previously published works.

**Response To Additional Editor Comments:**
**Question1:**Please specify the purity of the studied compound within the Materials and Methods section.

**Response:**The purity of the compound under investigation has been documented in the manuscript.As follows:

Gastrin (MCE, China, purity: 99.83%) and CI-988 (Tocris Bioscience, UK, purity≥98%) were administered by intraperitoneal injection, while ISO (Sigma-Aldrich, USA, purity≥99.8%) was administered subcutaneously.(Line 166-169,Page 8)

**Question2:**Please ensure the inclusion of the registration number and the full date (day, month, and year) of the ethical approval for conducting animal experiments.
**Response:**The registration number and the full date (day, month, and year) of the ethical approval for conducting animal experiments have been contained in the manuscript.As follows:

This study received ethical approval from the Ethics Committee of Shanxi Provincial Cardiovascular Hospital(registration number: 2023kjt100; date:2023/12/1).(Line 178-181,Page 9)

**Question3:**Methods for animal anesthesia and euthanasia should be provided.
**Response:**Methods for animal anesthesia and euthanasia have been registered in the manuscript.As follows:

After two weeks of treatment, six mice per group were randomly selected and humanely euthanized by cervical dislocation, and their cardiac tissues were harvested for molecular analyses. (Line 172-174,Page 8)

**Response to reviewer #1:**

Thank you for kindly taking the time to review my manuscript. I am deeply grateful to the editor for the considerable effort and dedication invested in the editorial and review process. I sincerely appreciate the opportunity to contribute to the journal and am fully committed to incorporating any feedback provided. I welcome all suggestions and will promptly respond with thoughtful revisions.

**Response to reviewer #2:**

**Question1:**The manuscript is not paginated and no line number are indicated making reference to parts of text difficult for the reviewer.
**Response:** I sincerely apologize for failing to include line and page numbers in the initial submission of the manuscript, which may have hindered your review process. I have now properly numbered both pages and lines in accordance with the journal's requirements.

**Question2:**Abstract: The experimental design is not described making the understanding of results, difficult. What is CI-988? This is not explained.
**Response:** In accordance with your recommendations, the experimental objective and a concise experimental design have been incorporated into the abstract section, and the role of CI-988 has been clarified.As detailed below:

This article aims to investigate the effects of gastrin on myocardial remodeling and HF, as well as its potential signal transduction mechanisms.(Line 27-29,Page 2)

In vivo studies were initially conducted to investigate the relationship between gastrin and HF, as well as the effects of gastrin on myocardial remodeling and HF. (Line 30-32,Page 2)

Furthermore, the effect of gastrin on cardiomyocyte hypertrophy was investigated in vitro using H9C2 cells , with F-actin staining and qRT-PCR analysis of ANP and BNP employed. (Line 38-40,Page 2)

CI-988( a CCK2R inhibitor) (Line 51,Page 3)

**Question3:** Introduction:

A. The gastrin part should be introduced earlier in the introduction.

**Response:** Your insights are highly valuable and have been carefully considered. In accordance with your suggestion, the section on gastrin in the article has been revised and advanced accordingly.

B. A long section talks about signalling in HF which is, in my opinion, unnecessary. The effects of various molecules on signalling pathway should not be described if it is not related to study. Maybe it is, but since the main player of this study, gastrin, has not been introduced, it is difficult for the reader to follow the intent of the authors.

**Response:** This article primarily investigates the effects of gastrin on myocardial remodeling and heart failure, with a further exploration of the underlying signaling mechanisms. In the introduction, the discussion focuses on signaling pathways implicated in heart failure, specifically highlighting two pathways relevant to this study: the ERK pathway within the MAPK cascade and the JAK2/STAT3 pathway. We have incorporated the introduction of gastrin in accordance with your suggestion, followed by a detailed description of the pathways associated with heart failure, thereby elucidating gastrin's influence on heart failure through these mechanisms.

C. It is mentioned that gastrin and its receptors are widely expressed in cell lines, animal and human samples without mentionning tissues or organs making this sentence useless.

**Response:** We have deleted this sentence as per your suggestion. We mentioned later in the text that gastrin and its receptor are expressed in myocardial tissue. As follows:

Notably, both gastrin and its receptor, cholecystokinin receptor 2 (CCK2R), show abundant expression within cardiac tissues.(Line 114-115, Page 6)

D. The authors seems to imply that gastrin is secreted in pathological conditions. They also propose that gastrin has beneficial effects suggesting that this endogenous secretion is insufficient and an external imput is necessary.

**Response:** Under physiological conditions, gastrin is secreted in both humans and animals. However, under certain pathological conditions, gastrin secretion may be elevated. For example, as noted in this article, gastrin levels increase in mice with heart failure. Nevertheless, the primary focus of this study is to investigate the protective effects of exogenous gastrin administration on heart failure and myocardial remodeling in murine models.

E. Overall, the authors did not introduce gastrin in a way that links it to other roles than its traditional digestive roles.

**Response:** Based on your suggestions, this article expands upon the traditional understanding of gastrin by incorporating its newly recognized role in signal transduction. The details are as follows:

Historically recognized as a key regulator of gastrointestinal epithelial development and tumorigenesis, gastrin has, in recent decades, been the subject of extensive research. (Line 104-106, Page 5)

**Question4:** Animal experimentation:

A. I did not understand why half the animals were sacrificed after two weeks and another half after four weeks. I did not find an explanation for this elsewhere in the manuscript.

**Response:** Given that the medication duration for the ISO-induced mouse model of myocardial remodeling is 2 weeks, whereas the heart failure model requires a 4-week treatment period, molecular assessments related to myocardial remodeling should be conducted at the 2-week time point following model establishment.

B. References should be included for dosages.

**Response:** The reference literature listed after each drug concentration corresponds to the source from which the concentration value was derived. Specifically as follows:

The mice were then randomly assigned to five experimental groups (n =13mice/group): control, gastrin treatment (30 μg/kg/day) ^[15]^, ISO administration (10 mg/kg/day)^[29]^, ISO combined with gastrin, and ISO combined with gastrin plus CI-988 (300 μg/kg/day)^[15]^. Gastrin (MCE, China, purity: 99.83%) and CI-988 (Tocris Bioscience, UK, purity≥98%) were administered by intraperitoneal injection, while ISO (Sigma-Aldrich, USA, purity≥99.8%) was administered subcutaneously. (Line 162-169, Page 8)

C. A justification for studying only males should be included.

**Response:** To minimize gender-related variability(Line 158, Page 8)

**Question5:** Results.
**A.** Again, I was not certain looking at the figure if I was looking at 2 or 4-week groups.

**Response:** This experiment did not include a comparative analysis between mice subjected to 2-week and 4-week treatment durations. The treatment periods were established based on the distinct pathological timelines of ISO-induced cardiac changes: myocardial remodeling typically develops within approximately 2 weeks, whereas ISO-induced heart failure requires about 4 weeks for full manifestation. Consequently, myocardial tissue from mice treated for 2 weeks was used to assess indicators of myocardial remodeling, while tissue from mice treated for 4 weeks was utilized for the evaluation of heart failure markers.

B. Figure 1B seemed cropped on the left where control was written.

**Response:** Thank you sincerely for your thorough review of my manuscript. Figure 1B, which was previously obscured by IA, has been revised accordingly.

C. Starting in Figure 2, gene expression results were splitted into two. Ci-988 results being only in the Figures e to G.

**Response:** In this study, all molecular experiments were divided into two parts. One part included the control group, the ISO group, the gastrin group, and the ISO+gastrin group, with the primary objective of investigating the effect of gastrin on ISO-induced heart failure and myocardial cell hypertrophy. The other part consisted of the control group, the ISO group, the ISO+gastrin group, and the ISO+gastrin+CI988 group. CI988, a CCK2R antagonist, was used to determine whether gastrin mediates its biological effects via the CCK2 receptor.

D. Only 3 samples/group are illustrated, which is very low. The authors should justify this since it does not correspond to what they described in the Methods. The same is in Figures, 4, 5 and 6. The last figure is on cell culture studies.

**Response:** In gene expression experiments, three samples were selected from each group for data collection of the initial phase of the study. Subsequently, in accordance with your request, the remaining three tissue samples per group were also subjected to experimental analysis. All data have been collectively processed using appropriate statistical methods and the results have been re-visualized accordingly.The revised charts are presented in Figures 2, 3, and 5. Fig6 is on cell culture studies too.

**Question6:** Discussion.
The authors should refrain to describe their model as a HF model since they did not look at other manifestations of the syndrome such as lung congestion or lower exercise capacity.Many elements of the introduction should be in the discussion.

**Response:** This study incorporated animal experiments to investigate the effects of gastrin on myocardial remodeling and heart failure. In the myocardial remodeling component, ISO-induced modeling was conducted with a two-week administration period, whereas a four-week administration period was employed for the heart failure component. Furthermore, assessment of heart failure primarily involved the evaluation of left ventricular systolic function, specifically the ejection fraction (EF) value. Future research on heart failure will incorporate a more comprehensive evaluation of relevant clinical indicators, including pulmonary edema and reduced exercise tolerance, to enable a holistic assessment of disease severity and progression.

**Question7:** References: Correct #6, #9 and #32.

**Response:** We have corrected it as follows:

Qian JF, Liang SQ, Wang QY, Xu JC, Luo W, Huang WJ, et al. Isoproterenol induces MD2 activation by β-AR-cAMP-PKA-ROS signalling axis in cardiomyocytes and macrophages drives inflammatory heart failure. Acta pharmacologica Sinica 2024;45 (3):531-544.(Line686-688,Page 32)

Sun J, Hao W, Fillmore N, Ma H, Springer D, Yu ZX, et al. Human Relaxin-2 Fusion Protein Treatment Prevents and Reverses Isoproterenol-Induced Hypertrophy and Fibrosis in Mouse Heart. Journal of the American Heart Association 2019;8 (24):e013465.(Line694-696, Page 33)

Luo Y, Jiang N, May HI, Luo X, Ferdous A, Schiattarella GG, et al. Cooperative Binding of ETS2 and NFAT Links Erk1/2 and Calcineurin Signaling in the Pathogenesis of Cardiac Hypertrophy. Circulation 2021;144 (1):34-51.(Line756-758,Page 34)

**Response to reviewer #3:**

1. **Question1:** The rationale for using additional gastrin in this study is unclear. ISO treatment alone already increased serum gastrin levels, indicating that ISO leads to elevated endogenous gastrin content. Administering a gastrin antagonist should be expected to worsen myocardial hypertrophy and cardiac function, and such results would more directly support the benefits of gastrin treatment in ISO-treated mice.
   **Response:** The viewpoint “administering a gastrin antagonist should be expected to worsen myocardial hypertrophy and cardiac function”you have expressed is indeed highly reasonable. Your perspective seeks to elucidate the protective effect of gastrin through a counter-evidence approach, whereas my research will demonstrate this aforementioned effect through a positive and constructive framework. Although gastrin levels increased in the ISO-induced mouse model of heart failure, the elevation was limited in magnitude. Therefore, exogenous supplementation of gastrin is necessary to fully investigate its cardioprotective potential.
   **Question2:** Animal groups. In this study, gastrin treatment alone was used as another control group. The reviewer is curious why the authors did not include a CI-988–only treatment group.
   **Response:** Your suggestion is excellent and reflects the rigor of scientific research. We are also very grateful for this very constructive opinion you provided. This study mainly explores the effects of gastrin on myocardial remodeling and heart failure, and uses the CCK2R inhibitor CI-988 to demonstrate its biological effects. Our samples are still very well-preserved. We will further explore the impact of CI-988 alone on myocardial remodeling and heart failure in subsequent research, so as to more rigorously investigate the role of gastrin.
   **Question3:** Statistical analysis
   The authors stated that one-way ANOVA was used in this study. However, it is unclear whether all data passed tests for normality and equal variance. If not, what alternative statistical methods were applied?
   **Response:**All of our data meet the assumptions of normal distribution and homogeneity of variance.
   **Question4:** Results
   **A.** A major concern is the quality of the blots, particularly for phosphorylated proteins. In Figure 5A, the differences in p-JAK2 and p-STAT3 between groups are difficult to discern. Higher-quality images should be provided.

**Response:**We have re-conducted the experiments for the p-JAK2 and p-STAT3 bands as requested and obtained clearer and more interpretable results. The details are presented in Fig 5(A).
**B.** Figure 5E shows variability in GAPDH content between groups, particularly in the last five samples. It would be more appropriate to use total protein as a loading control.

**Response:**We sincerely apologize for the experimental inaccuracies that resulted in unsatisfactory internal reference bands in our previous submission. We have since repeated the experiment under controlled conditions, and the updated results confirm that the internal reference remains unaffected by the experimental treatments. The new band of GAPDH is presented in Fig 5(E).

**C.** In Figure 6, the authors need to specify the material used for this experiment.

**Response:** As specified in the figure caption, the research subject is the H9C2 cell line, and rhodamine-phalloidin was used as the reagent for cytoskeletal staining. The experimental materials presented in Figure 6 are explicitly described in the results section and corresponding figure captions. As detailed below:

To investigate cardiomyocyte size alterations, H9C2 cytoskeletal structures were visualized by staining F-actin with Rhodamine-phalloidin. (Line 498-499,Page 24)

Effect of gastrin on H9C2 cardiomyocyte hypertrophy. (A)Rhodamine- phalloidin staining of F-actin; (B)Cardiomyocyte surface areas; (C)(D) ANP, BNP in control, gastrin, ISO, and ISO+gastrin groups; (E)(F)ANP, BNP in control, ISO, ISO+gastrin, and ISO+gastrin+ CI-988 groups.(Line 514-519,Page 25)

E. Additionally, there are major quality differences between the Figure 6A and 6E panels for p-STAT3 and STAT3 images. The authors should explain these discrepancies.
   **Response:** In your query, you noted that "the STAT3 and p-STAT3 bands in 6A and 6E appear different"; however, the Western blot experiment is not included in Figure 6. It is likely that you are referring to panels 7A and 7E instead. During the experiment, variations in cell density may lead to differences in sample concentration. To account for this, the sample loading amount will be adjusted according to the measured concentration, with final loads ranging between 30 μg and 40 μg. As a result, slight variations in band intensity may be observed in the experimental results due to differences in loaded sample quantities, which could account for the differing gray values of STAT3 and p-STAT3 observed in lanes 7A and 7E.
   **Question5:** Discussion
   **A.** The authors state, “In this study, we established mouse models of ISO-induced MH and HF.” However, the ISO-induced heart failure model is already well established in the literature. What unique features does this model provide compared to previously reported studies?

**Response:** The expressions in the previous manuscript may have lacked sufficient precision. This study did not develop a novel heart failure model; instead, it adopted an established model of isoproterenol-induced myocardial remodeling and heart failure. Accordingly, the relevant sections of the manuscript have been revised for clarity and accuracy.Specifically as follows:

This study represents the first evidence demonstrating a significant cardioprotective effect of gastrin in ISO-induced heart failure models.(Line 554-555,Page 26)

B. However, after consuming a mixed meal, hypertensive adults exhibited significantly increased serum gastrin compared to normotensive controls. Thus, gastrin may potentially serve as a biomarker for various chronic diseases.” What do these statements mean?

**Response:** This passage aims to convey that gastrin levels are elevated in various chronic conditions and may serve as a potential biomarker for the prediction of chronic diseases.Certain portions of the original text were somewhat elaborate, potentially posing comprehension challenges for readers; accordingly, appropriate revisions have been implemented to enhance clarity and readability:

Gastrin-like peptides have been identified in the myocardium across various animal species.Our study demonstrated increased gastrin levels in mice with HF, consistent with findings from numerous prior studies reporting increased gastrin concentrations in various chronic conditions.

More importantly,[Tania](https://pubmed.ncbi.nlm.nih.gov/?term=Deis+T&cauthor_id=39875014) et al. found elevated gastrin levels in HF patients. Fang reported that plasma gastrin concentrations in patients with SMD were higher than those without sepsis. Similarly, chronic kidney disease has been linked to elevated serum gastrin levels. Jiang et al. found hypertensive adults exhibited significantly increased serum gastrin compared to normotensive controls after consuming a mixed meal.It is hypothesized that gastrin may serve as a biomarker for various chronic diseases.(Line 573-584,Page 27-28)

C. Please discuss the selectivity of CI-988 and its potential off-target effects. Since CI-988 is a CCK2R antagonist, clarification is needed on whether it might interact with other receptors or signaling pathways that could influence the observed cardiac outcomes.

**Response:**

**1.**Your consideration is highly reasonable. As noted in the text, CI-988 is a selective CCK2R inhibitor. As follows:

However, the combination of gastrin and CI-988 (a selective antagonist of cholecystokinin type-2 receptors) treatment diminishes the inhibitory effect of gastrin alone. (Line 602-604,Page 29)

2. Furthermore, given that CCK2R is widely distributed across multiple organs, long-term administration may lead to systemic adverse effects, which are described in the limitation section of discussion.As follows:

Finally,in this study, gastrin was administered systemically; therefore, its long-term therapeutic efficacy and potential extra-cardiac effects warrant more comprehensive preclinical assessment.(Line 650-653,Page 31)

**Response to reviewer #4:**

Thank you for reviewing my manuscript; I am deeply honored to receive your recognition. We will diligently and carefully revise the manuscript to meet the journal's requirements.

**Response to reviewer #5:**

**Question1:** It is not clear whether the gastrin levels are comparable in human heart failure as observed in the ISO-treated mice.

**Response:** Recent studies have demonstrated that gastrin levels are elevated in patients with heart failure. This finding has been incorporated into the manuscript.As follows:

[Tania](https://pubmed.ncbi.nlm.nih.gov/?term=Deis+T&cauthor_id=39875014) et al.found elevated gastrin levels in HF patients(Line 577-578,Page 28)

**Question2:** The ISO model is short term. What would be the effect of gastrin on chronic pressure overload or post-myocardial infraction remodeling? This should be explored or at least addressed in discussion section.

**Response:** Your proposal is highly commendable, and the content has already been addressed in the manuscript. As follows:

Fu et al. showed that gastrin enhances cardiac function after MI. (Line 586-587,Page 28)

As well as, our findings indicate gastrin effectively reduces myocardial fibrosis, consistent with its inhibitory effects on renal fibrosis in hypertensive nephropathy^[13]^ and cardiac fibrosis after MI. (Line 588-591,Page 28)
**Question3:** Recent studies on fibroblast-specific signaling (Hippo-YAP) in remodeling can be discussed in context of gastrin.

**Response:**

The present study primarily investigates the STAT3/JAK2 and ERK1/2 signaling pathways; however, it is possible that gastrin may also mediate biological effects through additional pathways. Future research should further explore these alternative signaling mechanisms as outlined in the manuscript.As follows:

Future studies should aim to validate these findings in additional models of sympathetic overactivation and further elucidate the underlying molecular mechanisms through which gastrin/CCK2R signaling modulates downstream pathways, including G protein-biased signaling, β-arrestin recruitment and Hippo-YAP signaling,etc.(Line 634-639, Page30)
**Question4:** STAT3 and ERK1/2 are known to be cardioprotective, whereas their inhibition appears to be protective in the manuscript. This could be context dependent regulation by gastrin, which should be discussed in detail.

**Response:** A detailed explanation of this issue has been provided in the manuscript.As follows:

When cardiac sympathetic overload occurs, the cellular signaling pathways(JAK2/STAT3 and ERK1/2) is activated, inducing myocardial hypertrophy as a compensatory mechanism to counteract myocardial stress induced by increased cardiac load. However, sustained activation of this pathway may lead to eccentric myocardial hypertrophy, ultimately contributing to the development of heart failure. Therefore, targeted inhibition of the hypertrophy signaling pathway may exert cardioprotective effects.(Line 607-614, Page29)
**Question5:** While the authors have used CI-988, the manuscript does not provide any evidence on CCK2R localization or expression levels in fibroblasts or cardiomyocytes.

**Response:**In the introduction, we mentioned the expression of CCK2R in cardiac tissue, as follows:

Notably, both gastrin and its receptor, cholecystokinin receptor 2 (CCK2R), show abundant expression within cardiac tissues. (Line 114-115,Page 6)

**Question6:**Discussion should address the limitation of only using cardiomyocytes and not cardiac fibroblasts for fibrosis experiments.

**Response:**The manuscript has been revised in accordance with your requirements.As follows:

This study has several limitations. First, the compelling anti-fibrotic effects of gastrin observed in vivo were not further dissected in an in vitro setting using cardiac fibroblasts. While our data from whole-heart tissue analyses strongly suggest the involvement of the CCK2R/JAK2/STAT3 and CCK2R/ERK1/2 pathway, future studies utilizing isolated fibroblast cultures are necessary to confirm the direct cellular target of gastrin and to rule out potential indirect effects mediated by other cell types in the cardiac microenvironment. (Line 640-647,Page 31)
**Minor Comments:
Question1:** State in the methods, whether histological quantifications were blinded.

**Response:** All histological staining procedures were conducted in a blinded manner.(Line 206-207,Page 10)
**Question2:** In abstract and discussion, the terms “hypertrophy reversal” and “functional recovery” should be used with clear distinction as these are not same.

**Response:**This study comprises two components of in vivo experimental research: the first investigates the effect of gastrin on improving cardiac function, and the second examines gastrin's role in ameliorating myocardial remodeling, specifically focusing on myocardial hypertrophy and myocardial fibrosis.
**Question3:**The figures appear hazy; authors are suggested to upload good quality images according to the journal’s requirements.

**Response:**Thank you for reviewing my manuscript. I have resubmitted the figures in accordance with the journal's requirements.
