# Supplementary material for: Gastrin ameliorates heart failure and suppresses myocardial remodeling via the JAK2/STAT3 and ERK1/2 pathways
Source: PLoS One. 2026 Mar 5;21(3):e0343403. doi: 10.1371/journal.pone.0343403 (PMC12962455; doi:10.1371/journal.pone.0343403)
Supplement: Attachment — Submitted filename: Response to reviewers 2026.1.27.doc [file pone.0343403.s006.doc]

**Response to reviewer #3:**

1. **Question1:** The rationale for using additional gastrin in this study is unclear. ISO treatment alone already increased serum gastrin levels, indicating that ISO leads to elevated endogenous gastrin content. Administering a gastrin antagonist should be expected to worsen myocardial hypertrophy and cardiac function, and such results would more directly support the benefits of gastrin treatment in ISO-treated mice.

**Response:**

Thank you for the reviewer’s insightful comments. It is indeed an important question. Administering a gastrin antagonist should be expected to worsen myocardial hypertrophy and cardiac function, and such results would more directly support the benefits of gastrin treatment in ISO-treated mice. However, because we are unable to obtain a gastrin antagonist. Therefore, in line with other studies, we used exogenous supplementation of gastrin to investigate the role of gastrin in ISO-induced cardiac hypertrophy. It has been addressed in the first paragraph of “Result” section of the revised manuscript(shown in red text).(Line 369-372,Page 18)

**Question2:** Animal groups. In this study, gastrin treatment alone was used as another control group. The reviewer is curious why the authors did not include a CI-988–only treatment group.

**Response:**

Thank you for your crucial comments. It is indeed an important question. We didn’t establish a CI-988-only treatment group as the control group in vivo. This is a limitation of this study. We have added it as a limitation in the paragraph 7 of the “Discussion” section of the revised paper (shown in red text). (Line 676-680, Page 32)

In the future, we will investigate the effect of CI-988-only treatment on ISO and/or TAC-induced cardiac remodeling in vivo.

**Question3:** Statistical analysis

The authors stated that one-way ANOVA was used in this study. However, it is unclear whether all data passed tests for normality and equal variance. If not, what alternative statistical methods were applied?

**Response:**

Thank you for your important and professional questions about statistical analysis. In the revived manuscript, we performed statistical analysis for all the data again. And a similar result was obtained for the data after all data passing tests for normality and equal variance. We have revised the manuscript accordingly, shown in red text in every Figure legend and in the “Statistical Analysis” section of the “Materials and Methods” section.

**Question4 (1):** Results: A major concern is the quality of the blots, particularly for phosphorylated proteins. In Figure 5A, the differences in p-JAK2 and p-STAT3 between groups are difficult to discern. Higher-quality images should be provided.

**Response:**

Thank you for the valuable comments. We test the p-JAK2 and p-STAT3 protein expression using western blot again, and provided a higher-quality images, as presented in Fig 5(A) in the revised manuscript.
**Question4 (2):** Figure 5E shows variability in GAPDH content between groups, particularly in the last five samples. It would be more appropriate to use total protein as a loading control.

**Response:**

Thank you for your valuable suggestions. We repeated the experiment under controlled conditions, and the updated results confirm that the internal reference remains unaffected by the experimental treatments. The new band of GAPDH is presented in Fig 5(E) in the revised manuscript.

**Question4(3):** In Figure 6, the authors need to specify the material used for this experiment.

**Response:**

Thank you for your valuable suggestions. We first apologize to you for our negligence. Here, we make a clear explanation for the figure 6.

(Figure 6A) Rhodamine- phalloidin staining of F-actin(400×), DAPI stained the nuclei. (Figure6 B) Quantification analysis of cardiomyocyte surface areas; (Figure6 C-D) RT-PCR analysis of ANP and BNP mRNA in control, gastrin, ISO, and ISO+gastrin groups; (Figure6 E-F) RT-PCR analysis of ANP and BNP mRNA in control, ISO, ISO+gastrin, and ISO+gastrin+ CI-988 groups. In the revised manuscript, we have specified the material in Figure 6 (shown in red text) (Line 529-533, Page 25-26).

**Question4(4):** Additionally, there are major quality differences between the Figure 7A and 7E panels for p-STAT3 and STAT3 images. The authors should explain these discrepancies.

**Response:**

Thank you for the positive comment. We test the STAT and p-STAT protein expression of Fig 7(E) using west blot. The new images have been presented in the Fig 7(E) of the revised manuscript.

**Question5: Discussion**

**Question5(1):** The authors state, “In this study, we established mouse models of ISO-induced MH and HF.” However, the ISO-induced heart failure model is already well established in the literature. What unique features does this model provide compared to previously reported studies?

**Response:**

Thank you for the positive comment. We apologize to you for our inaccurate description. Here we make an explanation. In fact, we aimed to describe that we successfully replicated the ISO-induced myocardial hypertrophy model, as previous study. In the revised manuscript, we have made a modification for the methods as shown (Line 573-575, Page 27)

**Question5(2):** However, after consuming a mixed meal, hypertensive adults exhibited significantly increased serum gastrin compared to normotensive controls. Thus, gastrin may potentially serve as a biomarker for various chronic diseases.” What do these statements mean?

**Response:** Thank you for the positive comment. In the revised manuscript, we have made a clear modification of the Discussion (shown in red text). (Line 595-609, Page 28-29)

**Question5(3):** Please discuss the selectivity of CI-988 and its potential off-target effects. Since CI-988 is a CCK2R antagonist, clarification is needed on whether it might interact with other receptors or signaling pathways that could influence the observed cardiac outcomes.

**Response:**

Thank you for the positive comment. In the revised manuscript, we made a clarification on whether CCK2R might interact with other downstream signaling pathways that could influence the observed cardiac outcomes. (shown in red text). (Line 641-654, Page 30-31)
